# Optimized Xanthenium Photocages with Fused Ring Systems for Photoactivated Chemotherapy and G Protein-Coupled Receptor Photopharmacology

**DOI:** 10.1021/jacs.6c02825

**Published:** 2026-04-08

**Authors:** Tibor Á. Molnár, Eszter Kozma, Anna Benedikti, Márk Holczer, Attila Kormos, Zsombor Gonda, Ákos Balde, Selina Pühringer, Verena Handl, Christian Bayer, Waltraud Huber, Nassim Ghaffari-Tabrizi-Wizsy, Linda Waldherr, Krisztina Németh, Márton Bojtár

**Affiliations:** † MTA—HUN-REN TTK Lendület “Momentum” Chemical Biology Research Group, Institute of Organic Chemistry, 280964 HUN-REN Research Centre for Natural Sciences. Magyar Tudósok Krt. 2, H-1117 Budapest, Hungary; ‡ Hevesy György PhD School of Chemistry, Eötvös Loránd University, Pázmány Péter Sétány 1/A, 1117 Budapest, Hungary; § Gottfried Schatz Research Center—Division of Medical Physics and Biophysics, Medical University of Graz, 8010 Graz, Austria; ∥ Otto Loewi Research Center—Division of Immunology, Research Unit CAM Lab, Medical University of Graz, 8010 Graz, Austria; ⊥ BioTechMed-Graz, Austria, Auenbruggerplatz 30, 8036 Graz, Austria

## Abstract

Photocages are light-responsive chemical protecting groups that enable the controlled release of bioactive molecules. As the need for precise spatiotemporal manipulation in increasingly complex systems continues to grow, the development of novel photocage scaffolds remains essential. Herein, we show how the structural and electronic properties of xanthenium photocages determine their ground state stability. Based on rational design, we introduce their next generation built on the julolidine auxochrome, resulting in ultra efficient, green to red light activatable photocages, **X590** and **X600H**. **X590** was applied in the light-controlled delivery of a potent tubulin inhibitor, monomethyl auristatin E (MMAE) and a topoisomerase inhibitor (SN38) with low light doses above 600 nm. The light activatable prodrugs possess high photoindices both in 2D cell cultures and 3D tumor spheroids and show antitumor effects in the chorioallantoic membrane (CAM) assay in live chicken embryos. The versatility of this new scaffold was demonstrated using a caged agonist for the light activation of serotonin 2C G protein-coupled receptor. Overall, these photocages should find wide applications in photopharmacology and the detailed studies presented herein could contribute to the clinical translation of photoactivated chemotherapy.

## Graphical abstract

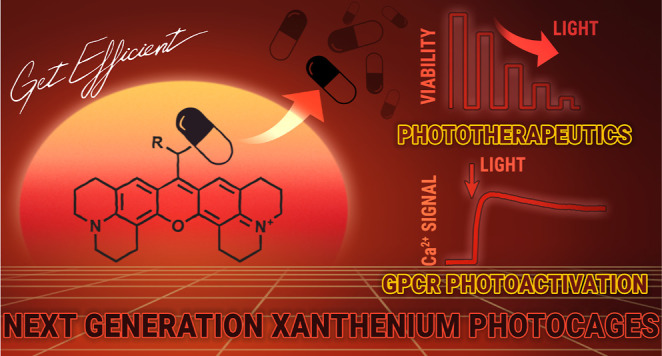

## Introduction

Photocages, also known as photoremovable protecting groups (PPGs) are essential tools of photopharmacology for spatiotemporal control over biological function.^1–4^ They enable irreversible, light-triggered release of bioactive compounds, thereby restricting biological processes to the site of irradiation.^5,6^ Notably, phototherapeutic approaches that rely on photocages linked to cytotoxic drugs as payloads are gaining increased attention for localized cancer therapy.^7^ Compared to photodynamic therapy (PDT), where light triggers the production of reactive oxygen species catalyzed by photosensitizers,^8^ photoactivated chemotherapy (PACT) does not require molecular oxygen and could present an alternative strategy, especially in hypoxic solid tumors.^9,10^ Despite the potential advantages of this emerging modality and the availability of light delivery technologies from PDT,^11,12^ clinical translation of PACT and photocaging in complex biological systems is still hindered by the lack of suitable PPGs.^13^ These requirements include (1) > 600 nm (one photon) activation for tissue penetration (2) considerable dark stability (3) high uncaging efficiency (4) aqueous solubility and biocompatibility (5) oxygen independence and (6) easy synthetic access.^4,14–16^ With the recent emergence of a wide variety of visible light scaffolds,^17–25^ most of these challenges are met individually, however, there is currently no photocage available that meets all these requirements. Despite the recent efforts devoted to visible light absorbing PPG development, most photocage classes, such as coumarins,^26^ BODIPYs,^24,27,28^ metal complexes^10,29,30^ and cyanines^21,22,25,31,32^ suffer from one or more drawbacks, mostly the lack of water solubility or sufficient wavelength/efficiency combinations.^33,34^

In 2023, we reported a set of xanthenium-based photocages analogous to rhodamine-type dyes as attractive scaffolds due to their tunable, visible-light absorption from the green to the red region, high extinction coefficients, and compatibility with aqueous environments (Figure 1a).^35^ Fundamental to their syntheses was the introduction of a “key methyl group” adjacent to the leaving group bearing carbon atom: the stabilization effect of this simple substitution prevented the irreversible, spontaneous formation of side-products in the dark, enabling the successful establishment of this new photocage class. While these PPGs are certainly appealing, their broader application has been so far hindered by limited accessibility, long synthesis routes, and limitations on payload attachment due to the requirement of the key methyl group. We also considered that improved chromophore stability and enhanced efficiency can not only ameliorate existing practices but could open entirely new avenues in drug delivery and light-assisted manipulations in chemical biology.^36^ Especially, when considering the potential translation of this modality into a therapeutic application, achieving long-term aqueous stability and second-scale activation times are not merely advantageous but essential for success.^15^

**Figure 1:**
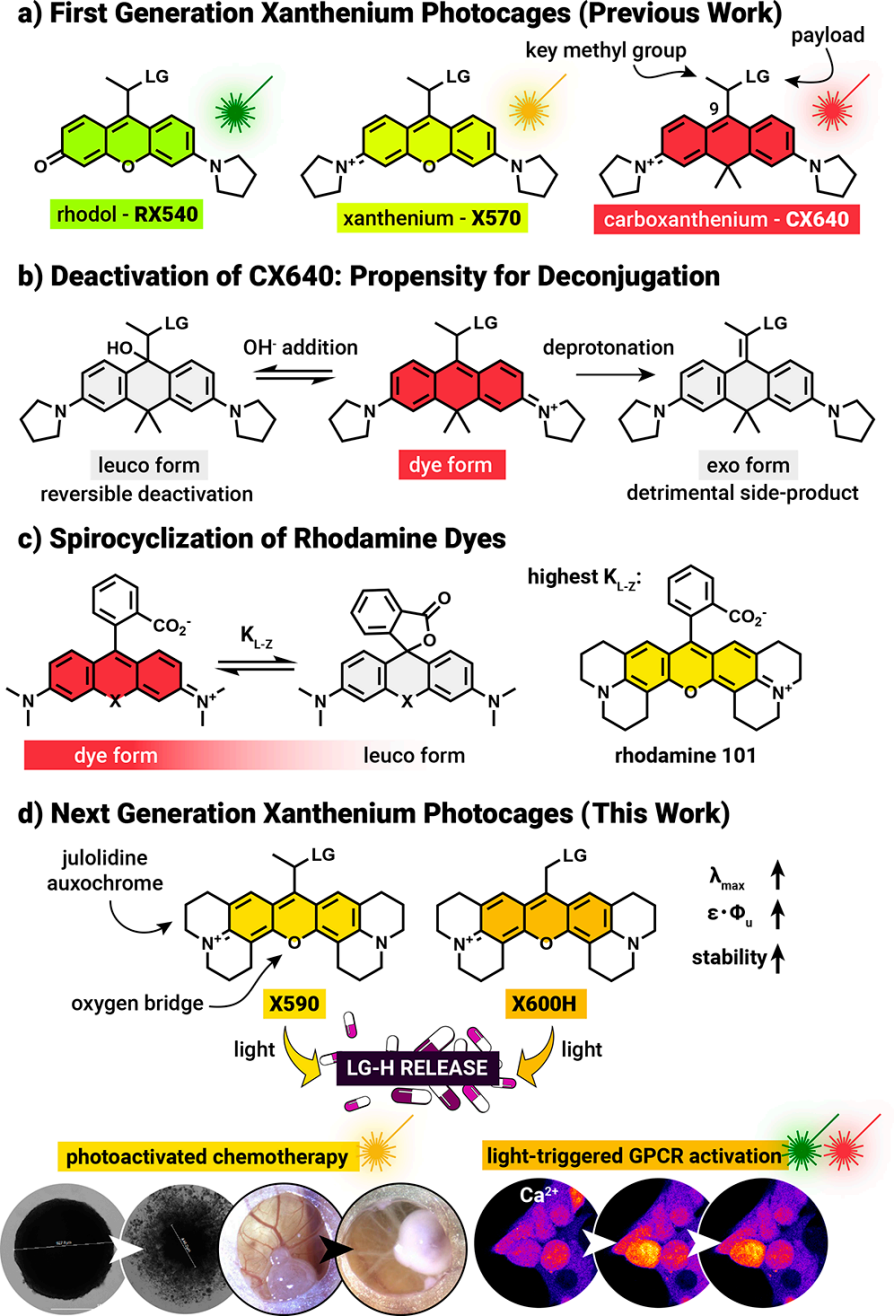
(a) Structures and naming of the first generation of xanthenium photocages; LG = leaving group (b) Ground-state deactivation of CX640 via nucleophile addition or deprotonation (c) Spirocyclization of rhodamine dyes (d) Multiring appended photocages reported in this work.

Initially, we directed our efforts toward increasing the absorption wavelength of the chromophores with the replacement of the position 10 (hetero)­atom to generate Si^37^ or P-rhodamine^38^ analog photocages. These established fluorophores are further red-shifted compared to carborhodamines and could represent attractive PPG scaffolds for efficient, near-infrared (NIR) triggered drug delivery.^39^ After several failed attempts, we realized that 9-alkyl Si/P-pyronines are unstable and form colorless side-products, a hypothesized olephinic “exo” form. We assume that the higher electrophilicity of the xanthene ring in such cases prevents the formation of the stable dye form of these scaffolds.^40–42^ Very recently, we showed that the analogous aminofluorone (rhodol) scaffold, named **RX540** can reversibly form an adduct with water, resulting in a colorless, deactivated or “camouflaged” photocage.^43^ Variation of the substitution pattern on the oxygen auxochrome resulted in different dye-colorless (“leuco”) form equilibria, a process akin to the spirocyclization of xanthene dyes.^44^ While the two ground state deactivation mechanisms are significantly different, we hypothesized that they both related to the electrophilicity of the chromophores which generates a propensity for deconjugation (Figure 1b). This well understood phenomenon is the basis of spirocyclization of rhodamine dyes, the intramolecular nucleophilic attack to position 9 of the xanthene chromophore (Figure 1c).^45–49^ Pyronine dyes lacking the adjacent ring of rhodamines are less explored, however several reports show a similar general behavior with nucleophiles.^42,50^ Xanthenium photocages are no different, however, their deactivation mechanisms, crucial to their ground state stability and for their wider applicability remained elusive.

In this paper, we show how the electrophilicity of the ring system is key to the ground state stabilization of the xanthenium photocage platform. Based on rational design, we present the next generation of xanthenium photocages based on the julolidine auxochrome: these electron donating substituents have a synergistic effect by significantly increasing the photochemical efficiencies and by stabilizing the chromophore scaffold even without the need of the key methyl group (Figure 1d). The new PPGs were applied in the light-controlled delivery of a potent tubulin inhibitor, monomethyl auristatin E (MMAE) and a topoisomerase inhibitor (SN38) with high photoindices in 2D cell cultures, 3D tumor spheroids and in the chorioallantoic membrane (CAM) assay in live chicken embryos, the first proof-of-concept of in vivo compatibility of this photocage family. Furthermore, the suitability of these novel PPGs for fast biological processes was demonstrated by using a caged agonist for the light activation of serotonin 2C G protein-coupled receptors.

## Results

### Propensity for Deconjugation Leads to Ground State Destabilization of Red-Shifted Photocages

To provide experimental evidence to the deactivation mechanism of xanthenium photocages, we first isolated the “exo form” of **CX640**, the most red-shifted carboxanthenium^35^ derivative with *N*-phenlypiperazine (**PP**) as a payload via carbamate (Figure 1b). Omission of the ion exchange step after rearomatization with chloranil results in substantial side-product formation whose structure was assigned to the olephinic exo form (see Section 2 in the Supporting Information). Treatment of **CX640-PP** (iodide salt) with excess triethylamine in dichloromethane also leads to its slow conversion to the exo form, as monitored with HPLC-MS (Figure S2). We suspect that in both cases, the exo form is generated via deprotonation either by the triethylamine base or the chloranil-derived phenolate. Furthermore, we observed a noticeable discoloration of **CX640**-caged compounds in aqueous solutions, pronounced at higher pH values (see stability at pH 9.0 on Figure S3). This process can be ascribed to a second deactivation process by the reversible nucleophilic addition of water/OH^–^, similarly to the deactivation of Si-pyronines reported by Johnsson et al.^50^ NMR studies confirmed this distinct OH-adduct in highly alkaline media (Figure S4). These results, together with the synthetic inaccessibility of Si-pyronine-based photocages, suggest that the electrophilicity of the ring system both in case of the aminofluorone (rhodol) and pyronine (rhodamine)-type photocages can have a major influence on ground state forms. In other words, the propensity for deconjugation of the ring system (either via reversible nucleophile capture or irreversible α-deprotonation) has a central role in the formation of detrimental side-products, determining their synthetic accessibility and long-term applicability in biological systems.

### Design Rationale of Multi-Ring PPGs

In the context of fluorescent dyes, the spirocyclization equilibrium, driven by the nucleophilic addition to the electrophilic chromophore depends on the intramolecular nucleophile, the auxochromes (electron donating substituents in positions 3 and 6) and the (hetero)­atom in position 10.^44–46,48,49,51–53^ Based on the abundant literature about this process we concluded that the julolidine auxochrome with oxygen in position 10 has the lowest propensity for deconjugation among rhodamines.^53^ Furthermore, the multiring system of this auxochrome increases both the absorption/emission maxima of the dyes and their fluorescence quantum yields (e.g., 543 nm/565 nm/0.49 for rhodamine B vs 564 nm/588 nm/0.92 for rhodamine 101).^54^ Thus, we expected that photocages built on the same chromophore could gain (1) enhanced stability due to the reduced propensity for deconjugation; (2) higher absorption wavelengths compared to the pyrrolidine-substituted analog **X570;** (3) superior quantum efficiencies and (4) the synthetic access to a primary alcohol-derived photocage. In line with the naming introduced in this paper, we termed the two new photocages **X590** and **X600H**.

### Synthesis of **X590** and **X600H** Photocages

For the hydroxyethyl-type photocage **X590**, we relied on our established route starting from the corresponding xanthone starting material **3** (Scheme 1).^35^ The xanthone can be accessed from commercially available rhodamine 800 (**2**) in just one step, however, synthesis is also possible from 8-hydroxyjulolidine (Scheme S2 in the Supporting Information), that provides **3** in just 3 steps with an overall yield of 49%. Utilizing the previously established Umpolung strategy,^55,56^ the xanthone can be converted to the direct photocage precursor (**5**) by α-lithiated ethyl vinyl ether addition, subsequent acidic hydrolysis and reduction using sodium borohydride. Payload attachment is possible via standard carbamate formation reactions using either disuccinimidyl carbonate (DSC) or dipyridyl carbonate (DPC).^57^ After coupling to the model amine payload **PP**, the rearomatization was performed using chloranil and subsequent anion replacement with hydrogen iodide. As mentioned above, this step was crucial for the stability of the carboxanthenium photocage **CX640**, however, **X590-PP** with tetrachloro-hydroquinone as the anion was completely stable in the dark (Figure S26). To obtain photocage **X600H**, **1** was reacted with KCN without the oxidation step then subsequent Pinner reaction with methanol/sulfuric acid afforded the reduced 9-carboxypyronine **8**. Reduction by LiAlH_4_ afforded key intermediate **9**, which was used immediately to avoid degradation. Activation of the primary alcohol and subsequent carbamate bond formation with **PP** yielded the reduced photocage which was oxidized using elemental iodine, providing the iodide anion in one step. For comparison, we also synthesized a rhodamine B analog featuring diethylamino auxochromes (**X565,** Scheme S5). Synthesis of a similar compound without the methyl group (**X575H**, Scheme S6) failed, suggesting that the julolidine auxochrome is indeed necessary for adequate stability for isolation.

**Scheme 1:**
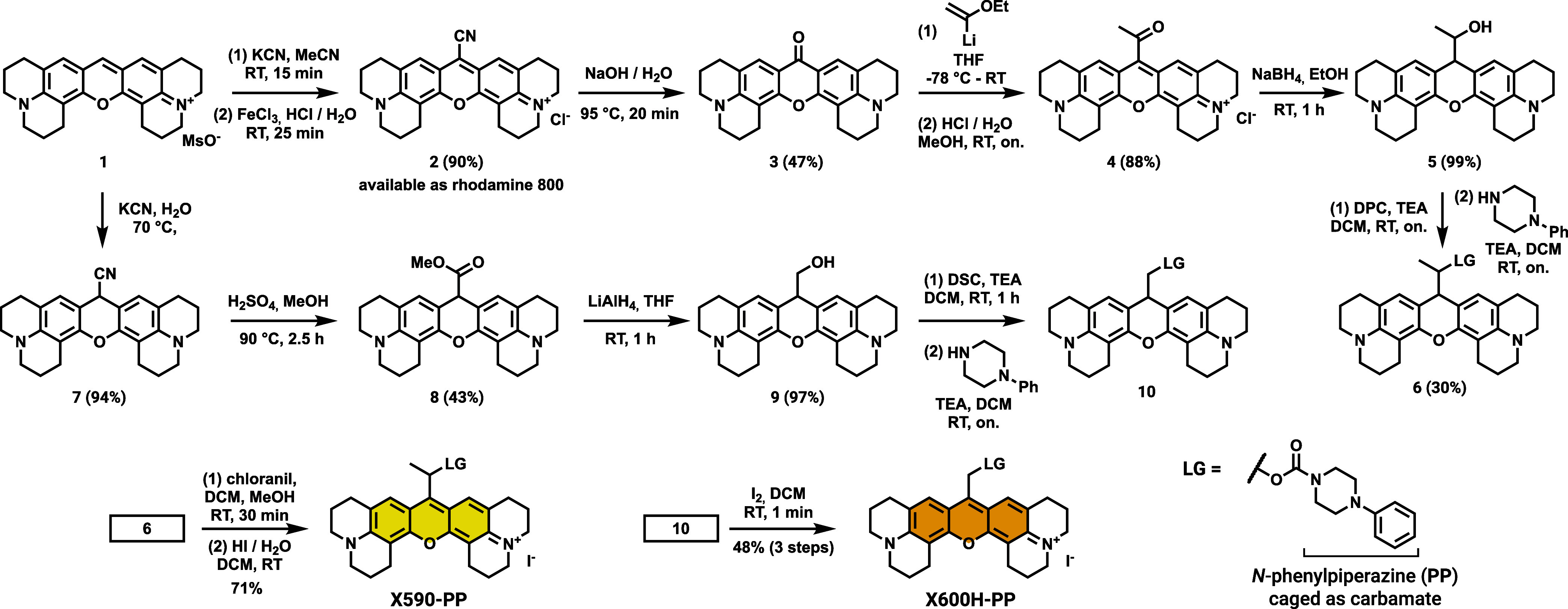
Synthesis of the Photocages and Model Compounds **X590-PP** and **X600H-PP**

### Multi-Ring Systems Improve Efficiency and Dark Stability

With the model compounds in hand, we assessed their spectroscopic properties (Table 1). Compared to their fluorophore counterparts, the absorption and fluorescence maxima (λ_max_/λ_em_) of the compounds are significantly red-shifted (589/610 nm and 601/620 nm for **X590-PP** and **X600H-PP**, respectively, Figures S6 and S7). Both photocages have large absorption coefficients up to the red range and high fluorescence quantum yields in water without signs of aggregation (at least up to 20 μM, Figure S8). Upon exposure to orange (605 nm, 270 mW output power) or red light (630 nm, 400 mW output power) in water/MeCN 9:1, both compounds released *N*-phenylpiperazine rapidly, as monitored by HPLC-MS with uncaging quantum yields of 5.6% for **X590-PP** and 8.1% for **X600H-PP** (Figure 2, see Supporting Information Section 5 for details and Figure S20 for the red light curves). This, together with the high absorption coefficients result in exceptionally high quantum efficiencies exceeding 4000 M^–1^ cm^–1^. Compared to **X565-PP** (0.13%) and **X570-PP** (0.42%), the 43-fold and 13-fold increase, respectively, in the uncaging quantum yield is remarkable (Figure 2f). Although rhodamine 101 is only about twice as bright as rhodamine B, the observed increase is much larger than expected and points to a more complex role of this auxochrome, especially as julolidine-fused coumarins are only 5-fold more efficient than their diethylamino counterparts.^58^

**Table 1:** Photophysical and Photochemical Characterization of the Model Compounds

Compound	λ_max_ (nm)	λ_em_ (nm)	ε × 10^4^ (M^–1^ cm^–1^)	Φ_flu_ (%)	Φ_u_ ^a^ (%)	Φ_deg_ ^b^ (%)	ε × Φ_u_ (M^–1^ cm^–1^)^c^	Φ_Δ_ ^d^ (%)
**X565-PP**	567	589	8.60	16	0.13 ± 0.01	0.22 ± 0.01	112	-
**X570-PP**	568	590	8.16	20	0.42 ± 0.01	0.85 ± 0.06	343	-
**X590-PP**	589	610	7.86	68	5.6 ± 0.1	8.8 ± 1.1	4390	1.3
**X600H-PP**	601	620	5.27	26	8.1 ± 0.2	15.6 ± 2.2	4260	0.71

a Uncaging quantum yield, calculated from payload release.

b Degradation quantum yield, calculated from the disappearance of the conjugates; average of 3 trials.

c Uncaging quantum efficiency at λ_max_.

d ^1^O_2_ quantum yield.

**Figure 2:**
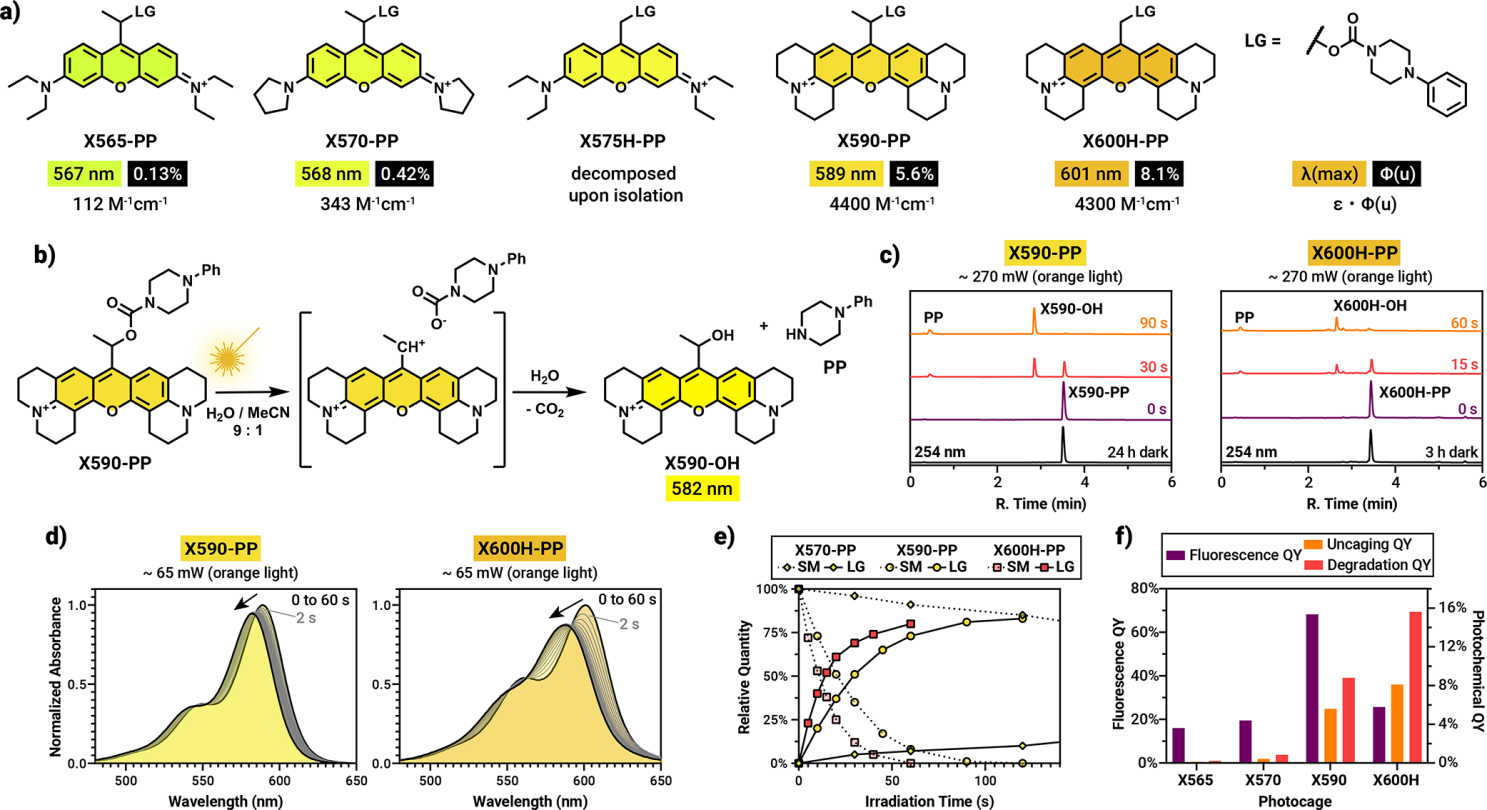
Photochemical studies. (a) Structure of the investigated model compounds together with their absorption maxima, λ­(max), uncaging quantum yields, Φ­(u) and uncaging efficiencies, ε × Φ­(u). (b) Suggested photosolvolysis mechanism of the uncaging of **X590-PP**. (c) Uncaging chromatograms of **X590-PP** and **X600H-PP** upon orange light irradiation. (d) Uncaging followed by UV/vis absorption measurements in water. (e) Uncaging curves of the model compounds as determined from the HPLC measurements. SM = starting material. (f) Comparison of fluorescence vs photochemical quantum yields of the model compounds (uncaging QY: calculated from payload release; degradation QY: calculated from the disappearance of the SM).

Besides the rigidification of the chromophore, the electron donating ability of the appended ring system could also stabilize the previously suggested cationic intermediate, thereby increasing the photolysis efficiency. This mechanism is supported by the exclusive appearance of the blueshifted alcohol **X590-OH**, the photosolvolysis side-product of **X590-PP** in water (HPLC chromatograms in Figure 2b, UV/vis spectra in Figure 2d; for detailed 2D contour chromatograms, see Section 5.2 in the Supporting Information). In case of **X600H-PP**, besides the major product **X600H**-**OH**, we also observed aldehyde formation after irradiation, possibly resulting from photooxidation. Stability experiments revealed unchanged starting material concentrations in the dark for **X590-PP** (in water and at pH 9.0, Section 5.4 in the Supporting Information), however, long-term hydrolysis and background degradation were observed for **X600H-PP** (after ∼24 h, Figure S27). This certainly excludes this photocage from PACT applications, however, rapid uncaging experiments, such as GPCR activation (see below) are still feasible. We also tested the two different anions: the hydroquinone salt of **X590-PP** had similar uncaging rates as the iodide (Figure S10), suggesting its negligible role in the photosolvolysis mechanism. Similarly to **CX640**,^35^ deoxygenation improves the uncaging rate of **X590-PP** by approximately 1.7-fold, confirming that oxygen is not required for the photolysis process (Figure S21). To assess potential photodynamic activity, we conducted singlet oxygen measurements with DPBF using methylene blue as a standard. Some degree of ^1^O_2_ generation was indeed observed for both photocages (around 1%, Figure S33), indicating the necessity of important control experiments in cellular viability assays (see below).

### Design and Synthesis of Photoactivated Chemotherapy Agents Based on **X590**

For PACT applications, **X590** was selected as the caging group due to its impressive stability and efficiency. Compared to the previously reported **CX640** (carboxanthenium) photocage the absorption maximum is lower, however **X590** is still compatible with 630 nm PDT light sources or other LED-based setups with orange to red emission (Figure S20).

We selected two highly potent cytotoxic agents: SN38, a camptothecin-based topoisomerase I inhibitor^59^ and monomethyl auristatin E (MMAE), a nonselective, highly toxic peptide-based tubulin polymerization inhibitor used widely in antibody-drug conjugates.^60,61^ In our previous studies, prodrugs of SN38 were already capable of reaching 2 orders of magnitude photoindices (defined as the ratio of dark/irradiated IC_50_ values), however, photocaging of MMAE was not reported before. Both prodrugs are connected to the PPG via carbamate bonds (Figure 3a), in case of SN38 this necessitates the incorporation of a self-immolative linker. MMAE can be conjugated to the secondary free amine of the peptide, which has an important role in its complex formation with tubulin.^62^ Synthesis of the prodrugs as outlined in Scheme S8 followed the general strategy. Uncaging experiments followed by HPLC-MS (Figures S14 and S15) revealed comparably efficient payload release in case of **X590-MMAE** and the model compound **X590-PP**, however, uncaging of **X590-SN38** was slower. This is in line with our previous observation with other photocages and could be ascribed to a potential quenching effect by the quinoline core.

**Figure 3:**
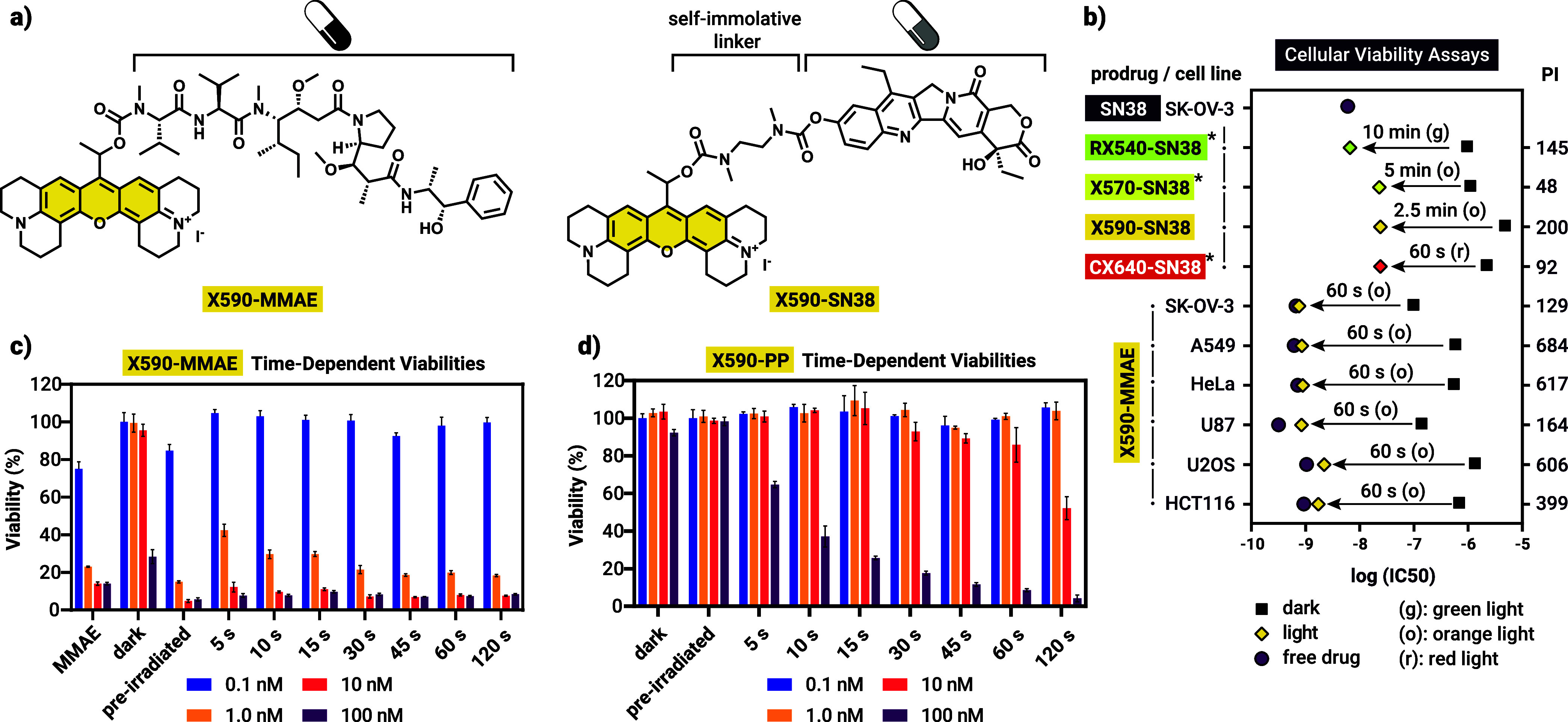
2D cellular viability data. (a) Structure of the prodrugs in this study. (b) Visual comparison of the IC_50_ values determined from viability assays of multiple photocaged drugs across multiple cell lines. The asterisk (*) marks previously determined data from our group.^35,43^ PI: photoindex. (c) Irradation time-dependent viabilities of SK-OV-3 cells treated with **X590-MMAE** and (d) the control compound **X590-PP**. Preirradiated refers to a sample irradiated in medium, then added to the cell culture.

### **X590** Prodrugs Enable Light-Activated Cell Toxicity

Next, we performed viability studies using prodrugs **X590-SN38** and **X590-MMAE** on SK-OV-3 (ovarian carcinoma) cells. In both cases, cells were treated for 72 h with either the prodrug, or the prodrug and 60 s (for MMAE), or 10 min (for SN38) of orange light irradiation (605 nm, 270 mW output power). Resazurin viability assays conducted at the end of the experiment revealed that the toxicities of the parent drugs (IC_50_ for SN38: 6 nM, MMAE: 665 pM, purple circle, Figure 3b) were lowered for both **X590-SN38** (4.8 μM) and **X590-MMAE** (98 nM, black squares), as indicated by the increased IC_50_ values. This remarkable increase in the latter case was rather surprising considering the significant toxicity of this antimitotic agent and its clinical use exclusively in antibody-drug conjugates. In both cases, irradiation restored most of the activity of the parent drug, leading to photoindices of 200 and 129 for SN38 and MMAE prodrugs, respectively. For the following experiments, we selected **X590-MMAE** due to its outstanding photoindex, rapid uncaging kinetics and the possibility of imaging-based readout (see below). Repeating the viability assay over a panel of 6 cell lines, consistently high photoindices were obtained, all above 2 orders of magnitude (Figure 3b and Table 2; for the concentration vs viability curves, see Figures S34 and S35). IC_50_ values and photoindices were reproducible across different laboratories (measured in Budapest and Graz, Figures S38 and S39). Furthermore, light dose-dependent viabilities on SK-OV-3 cells showed that only a fraction of the irradiation time is necessary (Figure 3c). For example, at a prodrug concentration of 10 nM, cell viability was maintained at 95% in the dark, however, 5 s of irradiation, equivalent to a light dose of ∼1.6 J already reduced viability to 12%, highlighting the excellent performance of **X590**. To confirm that phototoxicity indeed results from drug release, we performed the same light dose-dependent experiment with **X590-PP** (Figure 3d). Although the viability decreased after irradiation even below micromolar concentrations, much higher light doses were required in this control experiment. We also confirmed that neither light alone (data not shown) nor the photoproducts arising from the photolysis of **X590-PP** (“preirradiated” in Figure 3d) are toxic. Therefore, we believe that the low but seemingly significant PDT activity of **X590-PP** is responsible for its phototoxicity, similarly to some cases with BODIPY photocages.^63^ Further confirmation of MMAE release was obtained from microtubule imaging experiments. In this case, SK-OV-3 cells were treated for 24 h with either MMAE, **X590-MMAE** in the dark or **X590-MMAE** and light. Due to the shorter time frame and the different readout, higher doses were applied in these experiments. The disruption of the microtubule network was visualized after fixation and permeabilization of the cells using immunolabeling (see experimental details in Supporting Information Section 6.2.1). As can be seen in Figure 4a, **X590-MMAE** did not interfere with microtubule filaments at the dose of 100 nM, however, after irradiation (60 s), clear disruption can be observed by the lack of fluorescence signal, similarly to MMAE.

**Table 2:** IC_50_ Values (in nM) of X590-MMAE on Various Cancer Cell Lines

Cell line	dark	light	MMAE	PI^a^
**SK-OV-3**	98	0.76	0.67	129
**A549**	580	0.85	0.61	684
**HeLa**	540	0.88	0.72	617
**U87** ^b^	140	0.84	0.32	164
**U87** ^c^	410	2.45	0.71	165
**U2OS**	1340	2.20	1.03	606
**HCT116**	680	1.7	0.92	399
**HCT116 (3D)** ^d^	253	1.56	0.44	162
**HeLa (3D)** ^d^	105	0.37	0.49	284

a PI: photoindex (dark/light IC_50_).

b Measured in Budapest (resazurin assay).

c Measured in Graz (MTS assay).

d Measured in tumor spheroids.

**Figure 4:**
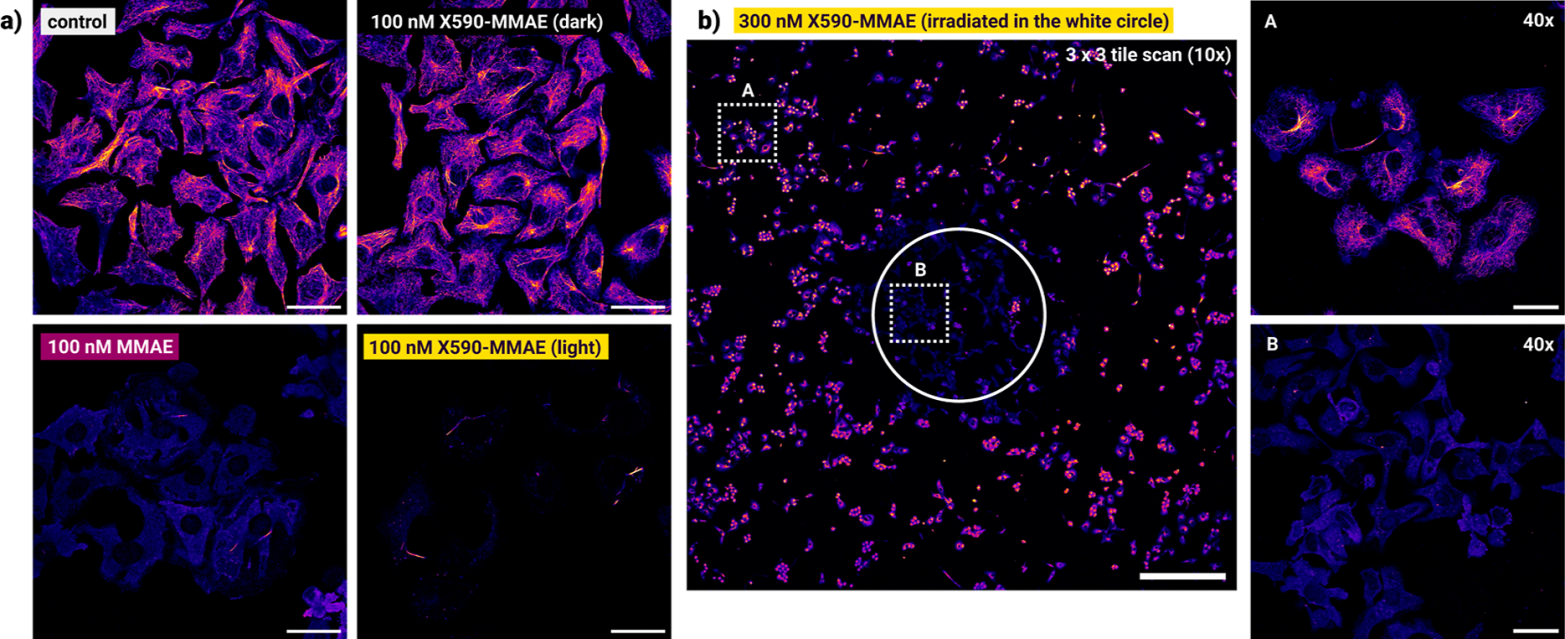
Microtubule immunolabeling of SK-OV-3 cells. (a) Whole well irradiation with **X590-MMAE** (dark and irradiated) or MMAE. Scale bar: 50 μm. (b) Masked experiment: irradiation was performed within the white circle (scale bar: 500 μm). A & B images show detailed structures using a different magnification within the dotted squares of the tile scan image (note that the higher magnification/numerical aperture results in lower slice thickness; scale bar: 50 μm).

Measurement of the intracellular intensity of the fluorescent signal revealed that even at higher concentrations (1 μM), the microtubule network is mostly intact in the dark upon **X590-MMAE** treatment while irradiation at low concentrations (10 nM) exerted almost the same effect as MMAE (Figure S42). Colocalization experiments revealed efficient cellular uptake of the prodrug with clear mitochondrial localization (Figure S47).

### Localized Irradiation Enables Spatiotemporal Control with Light

To assess the localization precision of MMAE uncaging, we performed masked irradiation experiments. First, we treated SK-OV-3 cells with **X590-MMAE** (300 and 500 nM) for 1 h, followed by replacement of the medium. We speculated that without the washing step, MMAE will also be uncaged in the supernatant, resulting in the likely loss of localization due to diffusion. We note that higher concentrations are needed of the prodrug for this experiment, as some of it is washed away (see whole-well irradiation experiments with media replacement followed by microtubule imaging in Figure S43). Then, cells were irradiated with orange light through a hole with a diameter of ∼1 mm, incubated for further 24 h then fixed and permeabilized. The localized effect of MMAE uncaging as visualized by microtubule immunostaining using confocal microscopy can be seen in Figure 4b (for the quantification of intracellular fluorescence, see Figure S46). The wider margin of microtubule destruction compared to the actual diameter of the hole (white circle) can be attributed to light scattering near the border. Impressively, the microtubule network of the cells behind the mask was unaffected, highlighting the local effects of photouncaging. We believe that this is due to the high affinity of MMAE to tubulin, essentially trapping the released payload in the cells.

### **X590**-MMAE Maintains Its Photoindex in 3D Tumor Spheroids

Drug development studies increasingly use tumor spheroids and organoids, as they more accurately approximate real tumor properties such as 3D structure and provision of oxygen and nutrients. Therefore, to investigate the performance of **X590-MMAE** in a more challenging in vitro model, we set up a 3D assay for the evaluation of light-induced drug release. HCT116 (human colon cancer) and HeLa cells were seeded, and the formed spheroids were treated after 2 days with **X590-MMAE** (dark or irradiated for 60 s with orange light) or MMAE (Figure 5). As can be seen in the figure, the high photoindices of the prodrug in 2D were maintained in the spheroid model. **X590-MMAE** was significantly less toxic in the dark with an IC_50_ of 253 nM and 105 nM, compared to 440 pM and 490 pM for free MMAE for HCT116 and HeLa cells, respectively. Upon irradiation for 60 s, the IC_50_ values were reduced to 1.56 nM (HCT116) and 370 pM (HeLa), revealing photoindices of ∼160 and ∼300, respectively. The viability results correlate well with the morphology of the treated spheroids (Figure 5c, for more images see Figures S48–S53). Free MMAE caused clear disruption of the compact spherical architecture of the spheroids at 1 nM, while the prodrug in the dark did not cause any noticeable disintegration up to 100 nM. Light irradiation of **X590-MMAE**-treated spheroids results in similar disruption of the structures as free MMAE, confirming its release.

**Figure 5:**
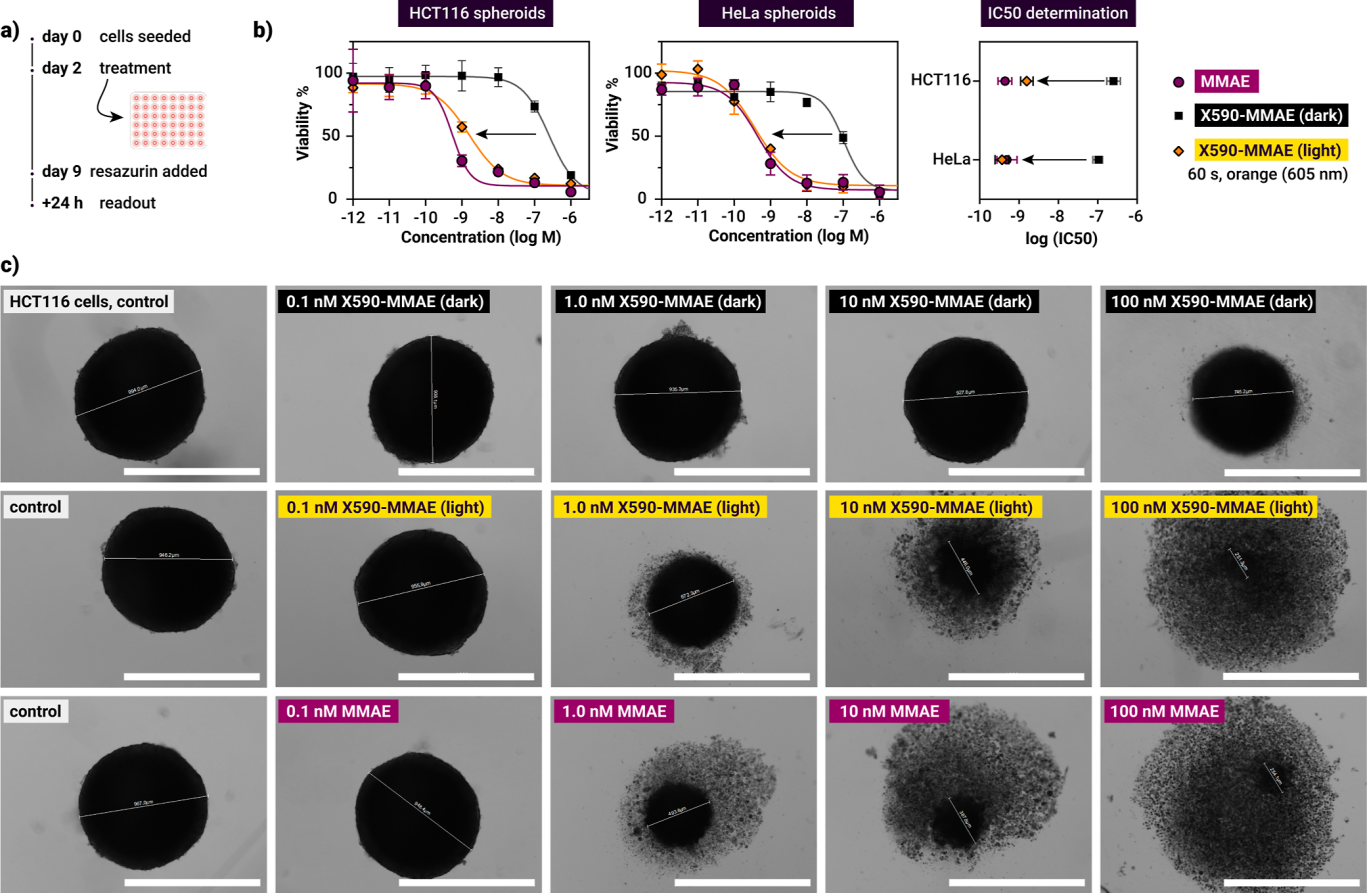
Spheroid results. (a) Treatment protocol for spheroid experiments. (b) Concentration-dependent viabilities in HCT116 and HeLa spheroids upon various treatments. (c) Bright-field microscopy images of HCT116 spheroids treated with X590-MMAE (dark and irradiated) and MMAE. Scale bar: 1000 μm.

### In Vivo Tumor Treatment with **X590-MMAE** Using the CAM Assay

Based on the efficient turn-on effect of the drug upon irradiation in 2D and 3D in vitro conditions, we tested the principle in a biologically more complex tumor system, the chorioallantoic membrane (CAM) model,^64–66^ according to Scheme 2. This model utilizes a chicken embryo as a host system for easily accessible tumor observation and analysis. Initially, we determined the toxicity of different MMAE concentrations in this host system by topically administering different concentrations of MMAE on the CAM and observing the survival of the chicken embryos over 120 h (Figure 6a). This showed that a dose of up to 0.5 nmol of the drug is not toxic to the embryos over 120 h. For the tumor treatment, we used our established U87 tumor model.^67^ U87 cells were engrafted on the membrane, resulting in a self-assembled, three-dimensional vascularized tumor after 72 h (see Figure 6b, growth control tumor surrounded by a silicone ring). After tumor formation, they were treated by topically administering a dose of 0.5 nmol (10 μL, 50 μM) of **X590-MMAE** onto the tumor, followed by irradiation with a homemade LED setup positioned centrally over the CAM tumors for 180 s (590 nm, 120 mW/cm^2^; for more details see Section 8 in the Supporting Information). As positive control, we treated tumors with free MMAE (0.5 nmol). Macroscopic documentation of the tumor sites pre (*t* = 0) and post (*t* = 24 h) topical treatment with both MMAE and **X590-MMAE** (irradiated) showed hampered vascularization, resulting in pale appearing tumors and reduction of blood vessels in the CAM, while **X590-MMAE** alone and the growth control appear with a pink hue coming from their intact vascularization (Figure 6b, 24 h).

**Scheme 2:**
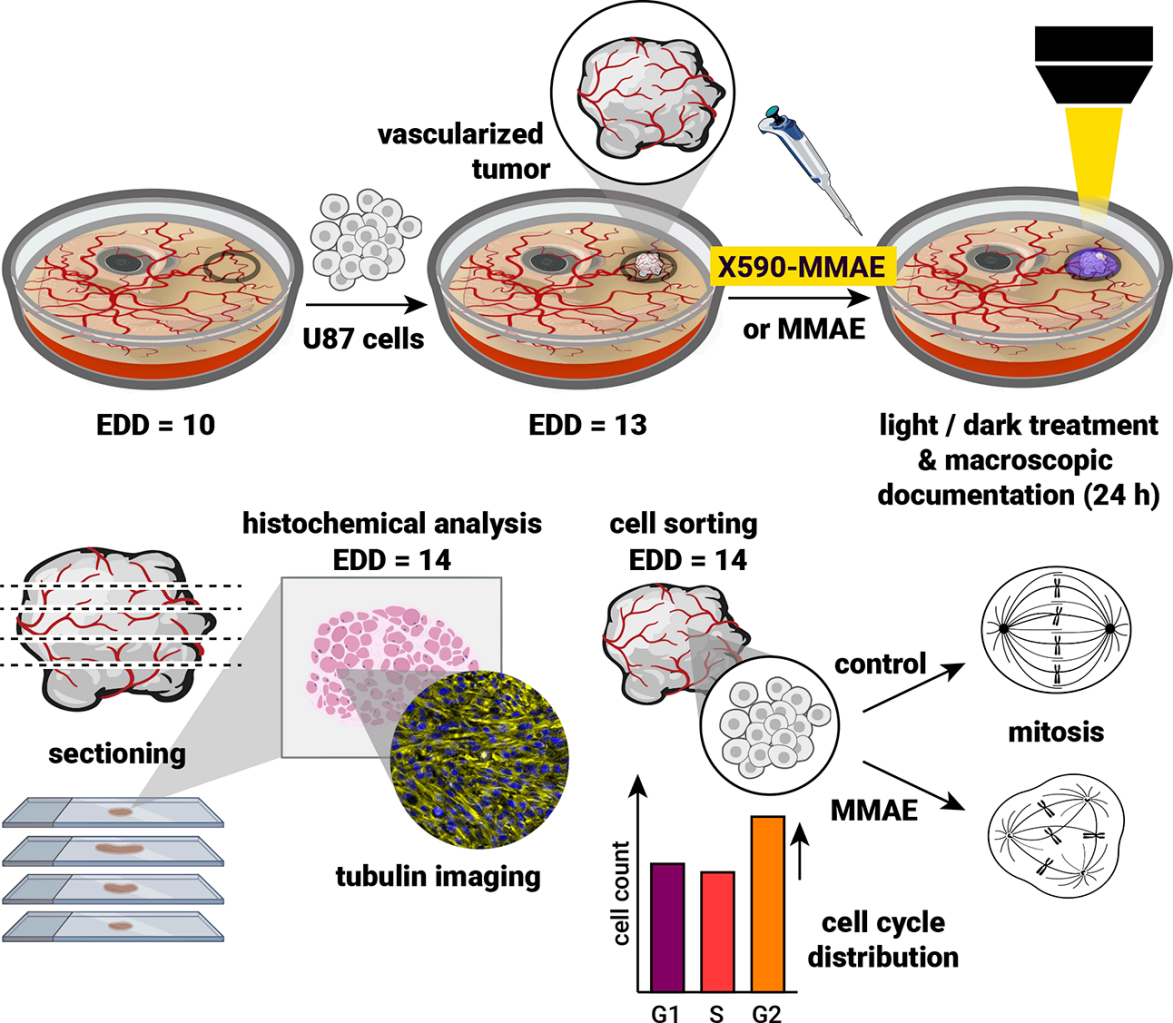
Overview of the Experimental Workflow and Analysis after Tumor Treatment in the CAM Model^a^

a Tumor tissue is processed for histological sectioning and microtubule imaging, followed by high-resolution microscopy of stained sections. In parallel, tumor-derived cells are analyzed via flow cytometry, illustrating effects on mitosis and cell cycle arrest. Quantification of cell cycle distribution indicates treatment-dependent inhibition of cell cycle. EDD = embryonic development day. Tumor tissue is processed for histological sectioning and microtubule imaging, followed by high-resolution microscopy of stained sections. In parallel, tumor-derived cells are analyzed via flow cytometry, illustrating effects on mitosis and cell cycle arrest. Quantification of cell cycle distribution indicates treatment-dependent inhibition of cell cycle. EDD = embryonic development day.

**Figure 6:**
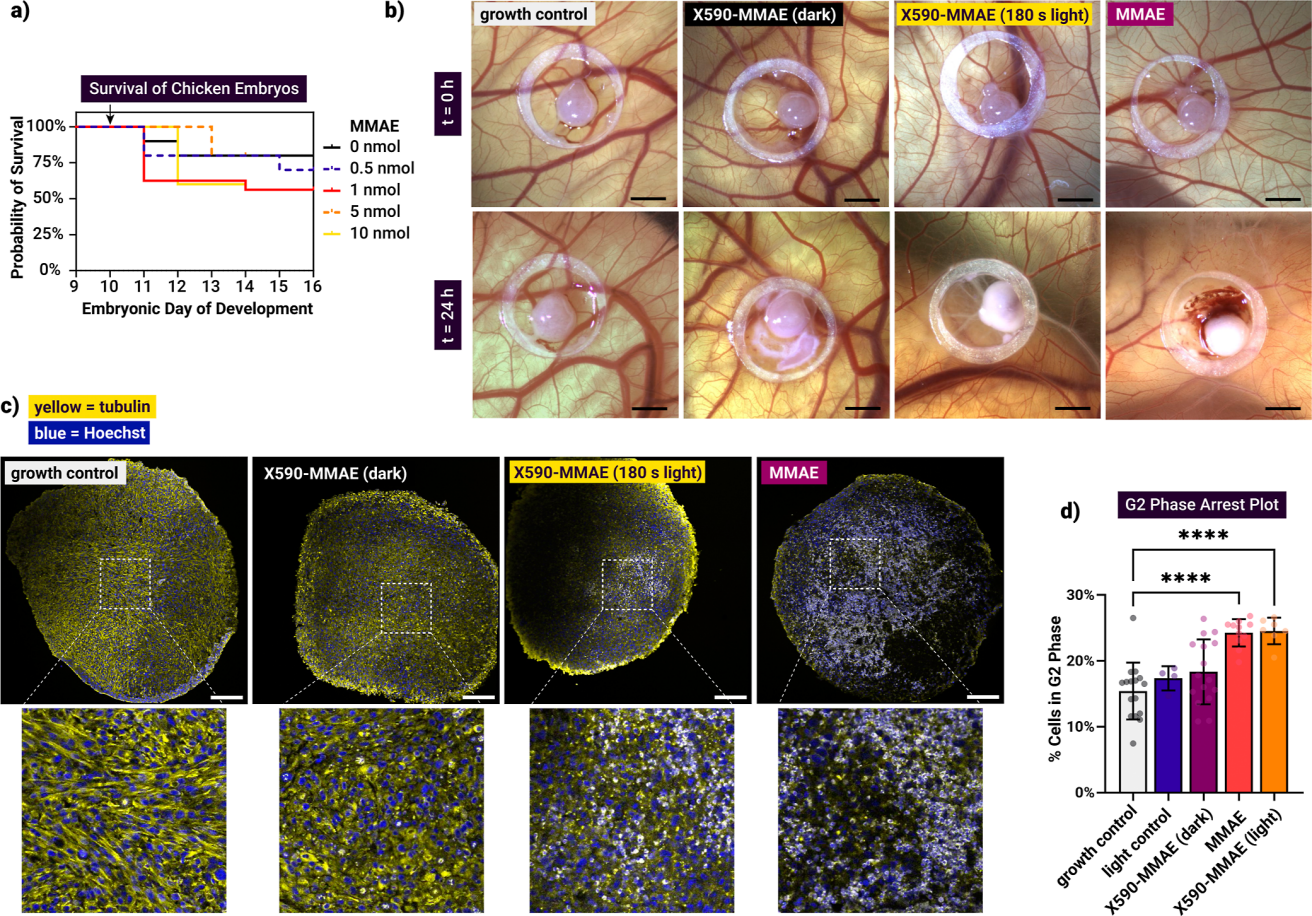
Uncaging of **X590-MMAE** to target U87 tumors cultivated on the CAM. (a) Kaplan–Meier plot for chicken embryo survival upon different MMAE concentrations over 120 h. (b) U87 tumors on CAM before (*t* = 0) and 24 h after photouncaging of **X590-MMAE** (50 μM, 180 s irradiation). Scale bar = 2 mm. (c) Microtubule and nuclei staining of CAM-grown U87, 24 h post treatment. Scale bar = 200 μm. (d) G2 cell cycle profiling of U87 CAM tumors upon different treatment conditions. Growth control = untreated; light = 180 s irradiation; prodrug = 50 μM **X590-MMAE**, no irradiation; positive control = 50 μM MMAE; prodrug + light = **X590-MMAE** + irradiation. Brown Forsythe and Welch ANOVA; ****: *p*-value <0.0001.

Microtubule staining supported macroscopic antitumor effect observations. Immunolabeling was performed in the superficial sections of the 3D tumors, observing the topical effects of this treatment. Tumors treated with **X590-MMAE** in the dark appeared slightly distorted with the microtubule network mostly intact, comparable to the untreated growth controls. In contrast, both free MMAE and **X590-MMAE** with irradiation caused extensive microtubule disruption, shown in a granulated signal (Figure 6c). Similar structural changes were observed in 2D cell cultures and are consistent with the known mechanism of MMAE and confirm effective drug release after irradiation in this model. To observe effects in the whole tumor tissue, we performed cell cycle profiling of the whole CAM grown U87 tumor. MMAE leads to G2/M phase cell cycle arrest and accumulation of cells in the G2 phase, following the mitotic blockade and microtubule destabilization.^68^ This effect was observed in tumors treated with free MMAE (24.52% in G2 phase), as well as those with irradiated **X590-MMAE** treatment (24.54% in G2, Figure 6d). The G2/M arrest was markedly stronger than in any of the control groups (15.42% G2 for untreated tumors, 17.35% irradiation only). In the absence of light **X590-MMAE** produced minimal G2 phase accumulation (18.33% X590-MMAE alone), indicating a necessity to optimize the concentration of the prodrug toward an ideal on/off effect for in vivo applications. This became even more important when we tried to uncage **X590-MMAE** from the circulation. First, we confirmed that the prodrug exerts significantly lessened toxicity to the embryos upon intravenous administration (Figure S56) compared to MMAE. In the latter case all embryos died within a day of treatment, while mortality in case of **X590-MMAE** (dark) did not increase significantly compared to vehicle treatment. However, after injecting the prodrug into the main vein of the chicken embryo and illuminating the tumor with the LED, we were unable to reproduce the G2/M arrest effects of topical administration of the prodrug, most likely because of dilution and rapid clearance (data not shown).

### Photocaged Serotonin Receptor Ligands Enable Light-Triggered GPCR Activation via One-Photon Uncaging

G protein-coupled receptors (GPCRs) represent one of the most important drug targets.^69^ Despite extensive study, GPCR signaling networks within cell remain complex and only partially understood,^70,71^ making them ideal targets for photopharmacological approaches that provide spatial and temporal control of receptor activation. *G*_q_ subunit coupled receptors, like serotonin 2C (5-HT_2C_) receptors, offer a clear, localized readout by transient Ca^2+^-imaging using established fluorescent calcium indicators and confocal microscopy. A previous study demonstrated light-triggered activation of serotonin 2C (5-HT_2C_) receptors using BODIPY photocages,^72^ however their inherent low solubility complicated live cell compatible imaging.

To demonstrate that the new photocages are suitable for GPCR photopharmacology, we conjugated both **X590** and **X600H** to WAY-161503 (**WY**), a 5-HT_2C_ receptor agonist (Figure 7a).^73^ The synthesis was performed similarly as before to form stable carbamate bonds with the secondary amine of **WY** (Scheme S10).

**Figure 7:**
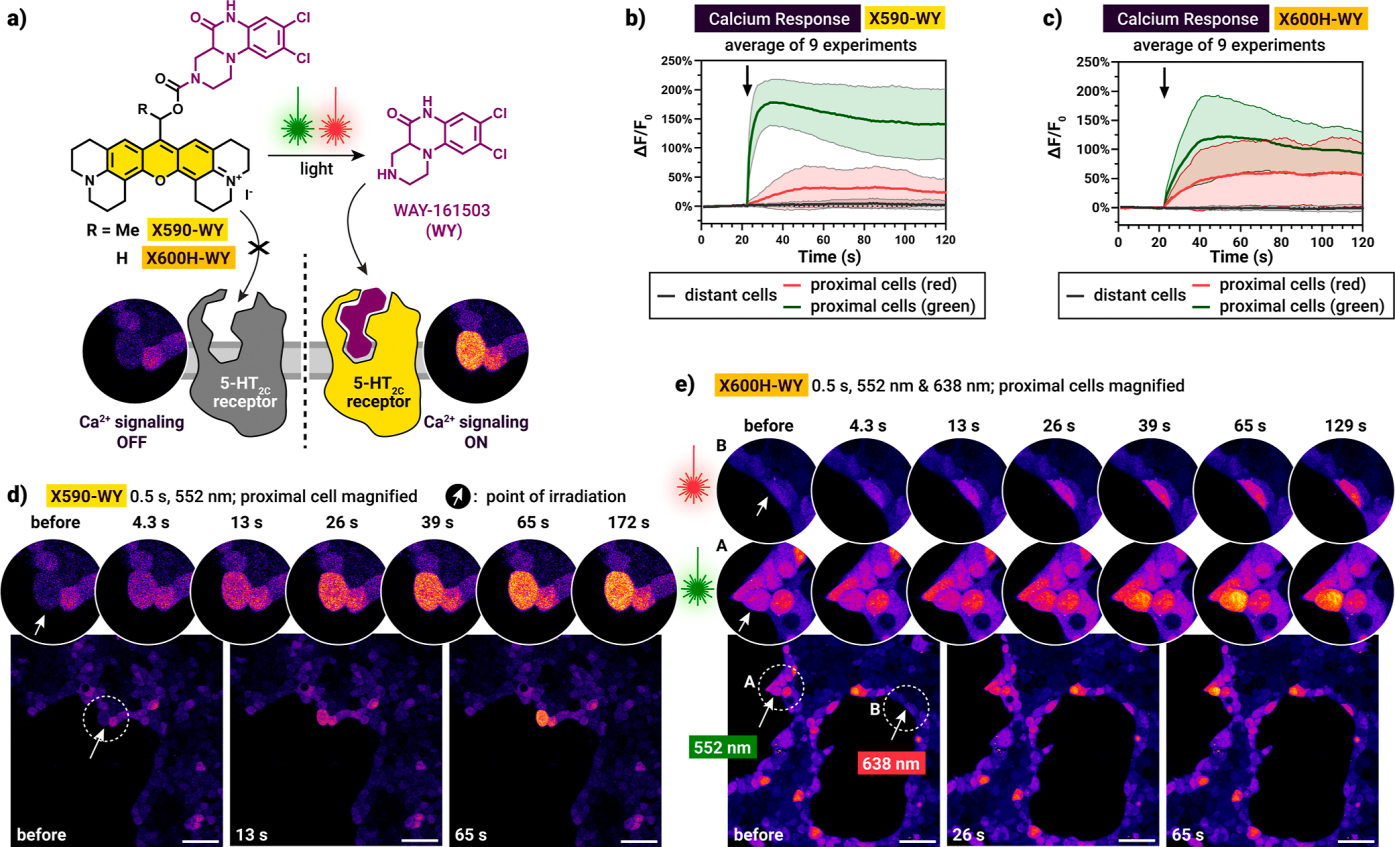
G protein-coupled receptor (GPCR) photopharmacology. (a) Schematic illustration of photocontrolled release of WAY-161503 (**WY**) and subsequent calcium signaling upon activation of serotonin 5-HT_2C_ receptor after green (552 nm) or red (638 nm) light irradiation of **X590-WY** and **X600H-WY** (5 μM). (b) Calcium response of **X590-WY** and (c) **X600H-WY** recorded during fluorescence imaging of Fluo-4 calcium indicator loaded into HEK293T cells expressing 5-HT_2C_R. Changes in fluorescent intensity compared to baseline (Δ*F*) were normalized to baseline fluorescence (Δ*F*/*F*_0_). Calcium response was recorded in the cells adjacent (green—552 nm and red—638 nm) or distant (gray) to the point of irradiation (*n* = 9). (d) A representative example of calcium imaging after **X590-WY** uncaging at 552 nm and (e) **X600H-WY** uncaging after irradiation at 552 and 638 nm for 0.5 s each. Time points represent the time elapsed after uncaging. Scale bars: 50 μm.

The conjugates maintained their spectroscopic properties and uncaging proceeded smoothly in water for both compounds (Figures S16 and S17). Next, we assessed light-triggered GPCR activation on live HEK293T cells transiently expressing 5-HT_2C_ receptors. Imaging and uncaging photolysis were performed with a laser scanning confocal microscope. Cells were loaded with calcium indicator Fluo-4-AM, then either **X590-WY** or **X600H-WY** (5 μM) was added, and a predefined point was irradiated at 100% laser power sequentially at 552 nm and then 638 nm for 0.5 s each. Before and after the irradiation, Ca^2+^ signal was recorded on the Fluo-4 channel (488 nm laser, detection: 500–540 nm). Calcium spikes induced by 5-HT_2C_ receptor activation were defined as changes in fluorescence signal normalized to baseline (Δ*F*/F_0_; see Figure S57 for the general workflow). As can be seen in Figure 7b,c, the brief irradiation resulted in localized, high Ca^2+^ spikes near the predefined site for both compounds, confirming the release of **WY** and subsequent GPCR activation cascade (Supporting Videos 1, 2, 3, and 4; for individual traces see Figures S58–S59). In the case of **X590-WY**, calcium transient appeared within 10 s and showed an average maximal increase of 178% in the normalized fluorescence signal. The calcium spikes after **X600H-WY** uncaging elevated somewhat slower, within 30 s, with an average maximal normalized fluorescence change of 122%. Despite the comparatively low absorption of **X590-WY** and **X600H-WY** at 638 nm, we observed clear uncaging using red laser irradiation and intracellular calcium elevation of 33% and 61%, respectively. We could not observe calcium response in cells distant from the point of irradiation. Uncaging experiments performed in the absence of **X590-WY** or **X600H-WY** did not result in calcium response confirming the specific activation of the target receptor (Figure S60).

## Discussion and Outlook

In this paper we have shown how rational design and optimization can lead to a new set of xanthenium photocages that are useful candidates for photoactivated chemotherapy or light-triggered receptor activation. This was achieved by first identifying the key structural and electronic properties that determine ground state stability. Similarly to xanthene-based fluorophores, photocages also have a propensity for deconjugation. While in case of rhodamine dyes this results in the canonical spirocyclization equilibrium, photocages undergo irreversible transformation into a colorless side-product via deprotonation (“exo form”) or react with nucleophiles reversibly (“leuco form”). While the latter can be exploited for conditional photocages,^43^ the preference toward the exo form in case of more electrophilic pyronines^42^ (e.g., Si-derivatives) precludes their successful synthesis. Following the hypothesis that similarly to spirocyclization, the ground state deactivation mechanism can be readily modulated by the substitution pattern, we designed photocages **X590** and **X600H**. Both scaffolds feature julolidine auxochromes,^53^ strongly electron donating substituents that lower the electrophilic character of the chromophore. The stabilization effect was sufficient to obtain **X600H** derivatives, featuring a xanthenium photocage that lacks the key methyl group.^35^ We devised synthetic routes that furnish key precursors in very few steps from commercially available starting materials. Spectroscopic and photochemical analysis revealed that both PPGs can be activated with green to red light and have exceptionally high photochemical efficiencies in water. Upon irradiation, a clean conversion of the model compound **X590-PP** was observed, resulting in the release of a free amine, **PP** and the photosolvolysis product, **X590-OH**. **X590** has a remarkable stability in the dark when connected to amines via carbamate, even at higher pH values. While the electronic modulation of the julolidine auxochromes enabled the preparation and isolation of **X600H**, its derivatives have lower aqueous stabilities. This lower long-term stability limits the use of **X600H** in prolonged experiments such as in PACT, however the primary alcohol derived caging group could potentially extend the payload scope due to reduced steric problems during synthesis. The auxochrome effect is even more striking when comparing the photochemical quantum yields with the diethylamino-analogs, **X565** and **X575H**. **X575H** could not be isolated, highlighting the need for the key methyl group in lieu of the julolidine auxochrome. While the model compound, **X565-PP** can be prepared, its photochemical efficiency is significantly inferior. This suggests that either the uncaging process benefits much more from the suppression of twisted intramolecular charge transfer (TICT) than fluorescence, or other effects are at play. It is likely that the electron donating auxochromes stabilize the cationic photosolvolysis intermediate, similarly to allylic groups in case of coumarin photocages.^74^

The high efficiency and dark stability of the **X590** scaffold prompted us to explore advanced PACT applications. While its absorption maximum lies well below the NIR range, photocages activatable in the first therapeutic window usually lack either the necessary solubility in water or the required efficiency for future translation of phototherapies. The high absorption coefficient, increased photochemical quantum yield, and compatibility with 630 nm PDT light sources^11^ make **X590** especially attractive. We synthesized two prodrugs featuring SN38 and MMAE as cytotoxic agents. Light-dependent viability assays on 2D cell cultures revealed surprisingly low toxicities of **X590-MMAE** compared to the free drug, while brief irradiation restored the activity of the tubulin polymerization inhibitor MMAE, reaching photoindices of 2–3 orders of magnitude on a panel of 6 cell lines. At higher nanomolar doses, merely 5 s of orange light irradiation (∼1.6 J) was sufficient in reducing viability to 12%. Very importantly, the high potency of the prodrug and light arose from the uncaging as confirmed by confocal microscopy imaging of the microtubule network and light dose-dependent toxicity studies using **X590-PP**. Nevertheless, we observed significant light-induced toxicities in the control experiment, highlighting the requirement of these studies as recently reported in case of BODIPYs.^63^ Crucially, the toxicity of **X590-PP** in the dark as well as **X590-OH**, the free photocage was considerably lower, also suggesting the involvement of a separate photodynamic process, possibly targeted to the mitochondria. This synergistic effect is nonetheless very useful for PACT applications, where the goal is cell death. While the loss of PDT effect in hypoxia (e.g., in highly aggressive solid tumors with reduced oxygen concentrations) is certainly expected, uncaging should still proceed as confirmed in deoxygenized solutions. While one of the greatest advantages of photopharmacology is its high spatiotemporal precision, the localized effect of photoactivated chemotherapy is not necessarily straightforward. Compared to one- or two-photon laser-triggered uncaging in a chemical biology context (e.g., glutamate or GABA uncaging),^75^ drug release is performed on a significantly larger scale, which can lead to unwanted diffusion from the site of irradiation. This is highlighted in a recent important study by Winssinger, Gotta and co-workers where they showed that localized retention of a Plk1 inhibitor after uncaging was only possible by simultaneously uncaging anionic carboxylate functional groups, effectively trapping the payload inside the cells.^76^ Another study, published by Szymanszky and Feringa showed how light-triggered antibiotic release can control bacterial growth.^77^ In our study, by using a simple masked irradiation experiment, we showed how even a noncoherent light source can restrict the effect of PACT to only a subset of cells within a ∼200 μm margin. Notably, the released payload did not need modifications (e.g., impermeabilization after uncaging), which we attribute to its high binding affinity toward tubulin. Preclinical studies on tumor spheroids and organoids are now increasingly preferred, especially with the recent paradigm shift of adopting advanced nonanimal models by the FDA.^78^ Very often, 2D cellular results translate to 3D models poorly, possibly due to decreased permeability of the compact spheroid architecture toward both the (pro)­drugs and light. We showed here that the high 2D photoindices of **X590-MMAE** translated well to 3D spheroid tumor models based on HCT116 and HeLa cell lines. These experiments simulate a more complex tumor environment compared to 2D cell cultures; however, this model still lacks circulation. A further layer of complexity was introduced by using the CAM assay. In this model, tumor growth is supported by host-embryo-mediated neovascularization, enabling nutrient supply and waste removal, therefore the opportunity to study transient drug concentrations, treatment schedules, and acute compound toxicities toward a living host system. Unlike mouse models, the tumor is readily accessible for optical irradiation and microscopic imaging and constitutes a static system that does not require implantable or tethered light sources for photouncaging. While this model system was used in the context of PDT studies,^79^ photoactivated chemotherapy applications were not evaluated with this assay before. The light-dependent toxicity observed in cell cultures also translated to the CAM assay following topical administration as confirmed by visual readout, cell cycle analysis and microtubule imaging. Preliminary intravenous experiments revealed that the prodrug is significantly less toxic to the embryo than the free drug, highlighting the stability of the **X590** conjugate in a model with circulation and metabolism. However, light treatment did not result in notable antitumor effects using the sensitive and early response cell cycle analysis readout. While further dose optimization is highly warranted, drug dilution effects and rapid clearance of the uncaged MMAE from the tumor site could be improved by immobilization or further means of targeting. Nevertheless, **X590-MMAE** exhibits robust light-dependent activation and potent cytotoxicity in the CAM tumor model.

Finally, we demonstrated that these new photocages enable the control of fast biological processes, such as GPCR activation and signaling using one-photon irradiation. The resulting calcium transient of the GPCR activation cascade offers clear visual readout of the localization precision of photouncaging using a spectrally distinct, commercial fluorescent calcium indicator (Fluo-4). This evaluation platform not only serves as a validation tool for new photocages but demonstrates the applicability of the xanthenium scaffold for GPCR photopharmacology. Since about one-third of currently approved drugs target GPCRs,^80^ light activation could offer a valuable approach to increase the selectivity of GPCR-targeted therapeutics or provide a useful tool from an investigative perspective. While optogenetic techniques offer powerful tools for light-triggered receptor activation,^81^ they inherently introduce a strong bias via the requirement of genetic manipulation. Redshifted, small molecular light activatable tools such as **X590-WY** and **X600H-WY** as well as related caged compounds can be used in nontransfected target tissues elucidating the diverse mechanisms of GPCR signaling in complex environments. Ultimately, we expect that such photocaged tools offering excellent spatiotemporal control can help better understand crucial physiological and pathological processes involving various biological signaling pathways.

## Conclusion

In summary, we showed how the propensity for deconjugation in xanthene-based chromophores determines the ground state stability of their photocage analogs. Based on this, we introduced the next generation of xanthenium photocages with julolidine auxochromes, resulting in efficient, green to red light activatable photoremovable protecting groups suitable for photoactivated chemotherapy and light-triggered GPCR activation. Their high performance and aqueous compatibility make them particularly appealing. Especially, **X590-MMAE** and the detailed studies presented herein could contribute to the translation of PACT into future clinical applications and provide enhanced tools for light-controlled interventions.

## Supplementary Material













## Notes

Supporting Information: Experimental details, synthetic procedures, characterization data, spectroscopic characterization, uncaging experiments, stability studies, details on the cell work and viability assessment, imaging experiments, further images, spheroid details and images, light sources, details of the CAM experiment and the GPCR activation experiments (PDF)
Compound Characterization: NMR spectra and HPLC-UV-MS chromatograms of the synthesized compounds (PDF)
Supporting Video 1: Fluo-4 (calcium) signal of the uncaging of **X590-WY** in 25x speed. The arrows indicate the irradiation points and their colors represent the lasers used (AVI)
Supporting Video 2: Fluo-4 (calcium) signal of the uncaging of **X590-WY** in 25x speed (AVI)
Supporting Video 3: Fluo-4 (calcium) signal of the uncaging of **X600H-WY** in 25x speed. The arrows indicate the irradiation points and their colors represent the lasers used (AVI)
Supporting Video 4: Fluo-4 (calcium) signal of the uncaging of **X600H-WY** in 25x speed (AVI)
