# Supplementary material for: Optimized Xanthenium Photocages with Fused Ring Systems for Photoactivated Chemotherapy and G Protein-Coupled Receptor Photopharmacology
Source: J Am Chem Soc. 2026 Apr 8;148(15):16379–93. doi: 10.1021/jacs.6c02825 (PMC13107459; doi:10.1021/jacs.6c02825)

Characterization Data

Optimized xanthenium photocages with fused ring systems for photoactivated chemotherapy and G protein-coupled receptor photopharmacology

Tibor Á. Molnár,[a,b] Eszter Kozma,[a] Anna Benedikti,[a] Márk Holczer,[a] Attila Kormos,[a] Zsombor Gonda,[a] Ákos Balde, [a] Selina Pühringer,[c] Verena Handl,[c] Christian Bayer,[c] Waltraud Huber,[d] Nassim Ghaffari-Tabrizi-Wizsy,[d] Linda Waldherr,[c,e,]* Krisztina Németh,[a,]* Márton Bojtár[a,]*

[a] MTA – HUN-REN TTK Lendület "Momentum" Chemical Biology Research Group, Institute of Organic Chemistry, HUN-REN Research Centre for Natural Sciences. Magyar tudósok krt. 2. H-1117, Budapest, Hungary. e-mail: bojtar.marton@ttk.hu; nemeth.krisztina@ttk.hu

[b] Hevesy György PhD School of Chemistry, Eötvös Loránd University, Pázmány Péter sétány 1/A, 1117 Budapest

[c] Gottfried Schatz Research Center – Division of Medical Physics and Biophysics, Medical University of Graz, 8010 Graz, Austria. e-mail: linda.waldherr@medunigraz.at

[d] Otto Loewi Research Center – Division of Immunology, Research Unit CAM Lab, Medical University of Graz, 8010 Graz, Austria

[e] BioTechMed-Graz, Austria, Auenbruggerplatz 30, 8036 Graz, Austria

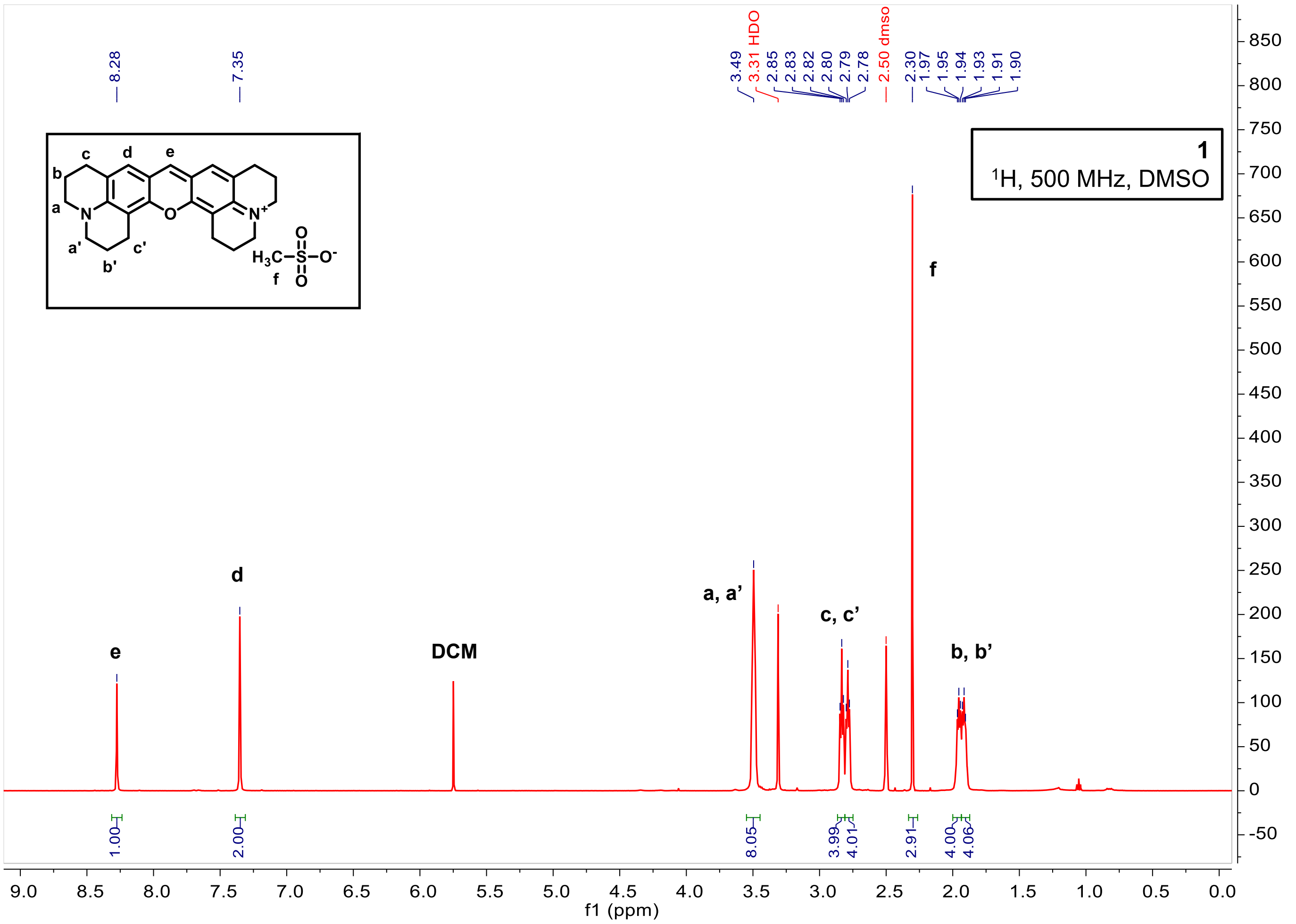

8.28
7.35
3.49
3.31 HDO
2.85
2.83
2.82
2.80
2.79
2.78
2.50 dmso
2.30
1.97
1.95
1.94
1.93
1.91
1.90
1
^{1}H, 500 MHz, DMSO
e
d
DCM
a, a'
c, c'
f
b, b'
1.00
2.00
8.05
3.99
4.01
2.91
4.00
4.06
f1 (ppm)

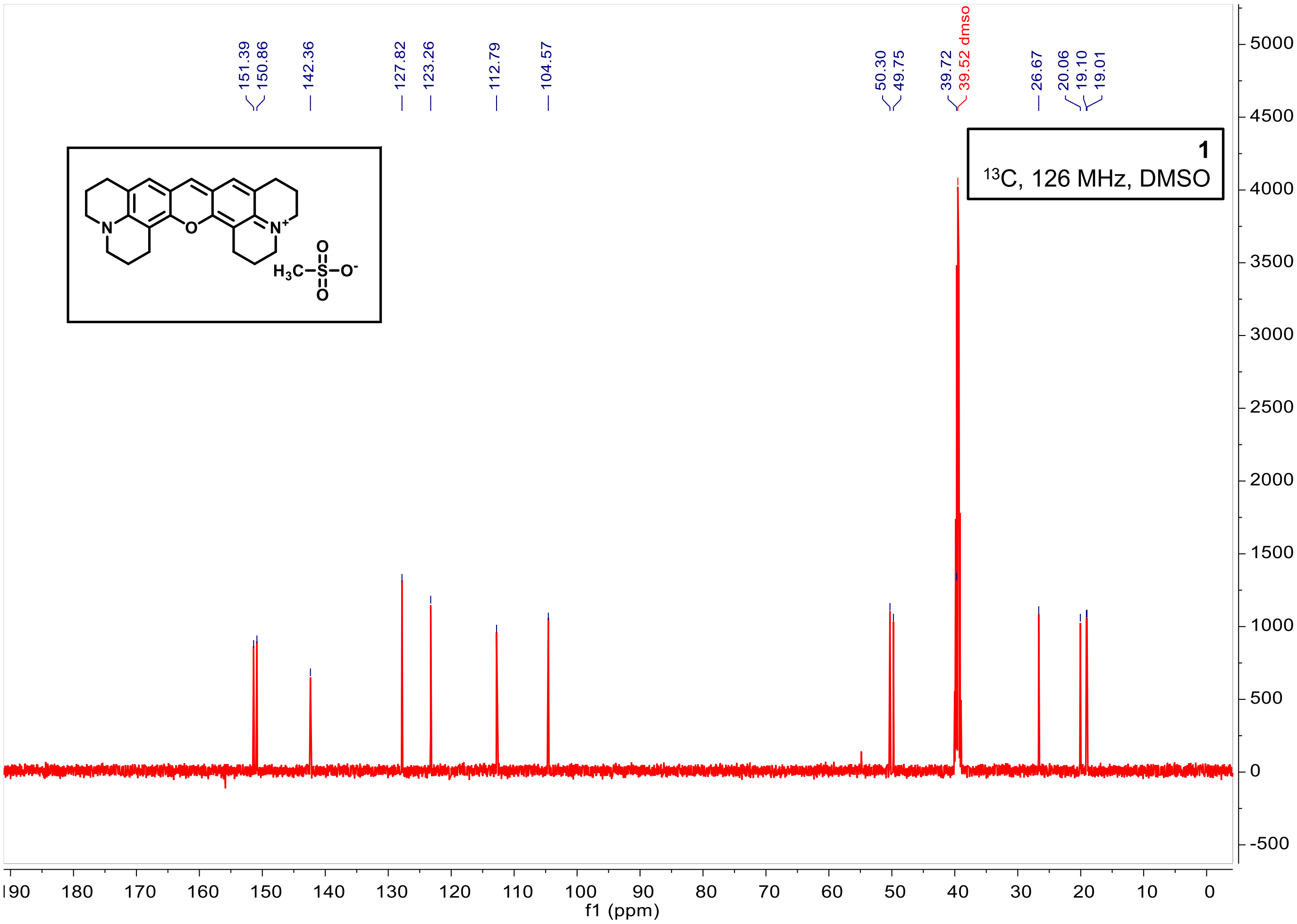

151.39
150.86
142.36
127.82
123.26
112.79
104.57
50.30
49.75
39.72
39.52 dmso
26.67
20.06
19.10
19.01
1
^{13}C, 126 MHz, DMSO
f1 (ppm)

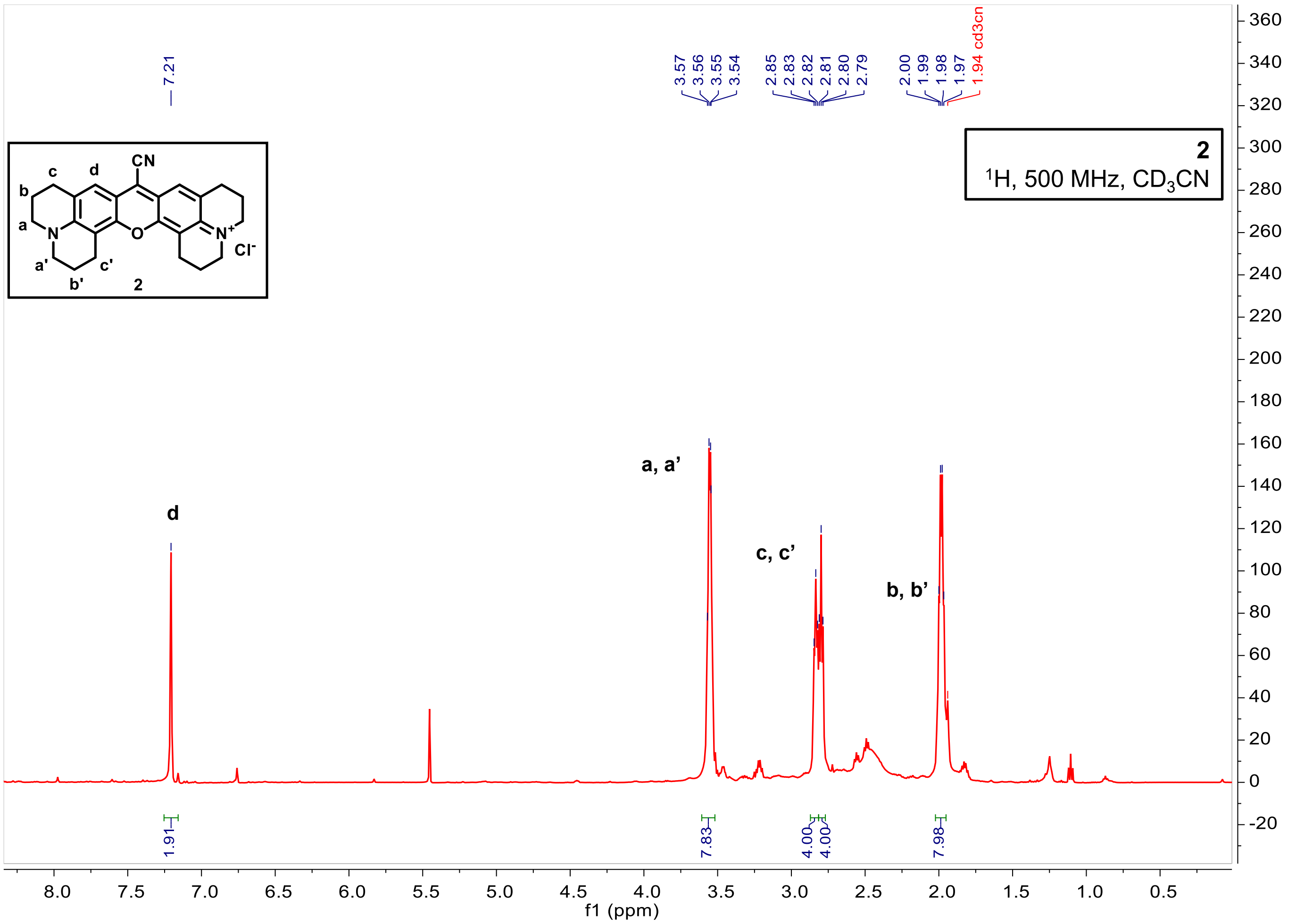
2
¹H, 500 MHz, CD3CN
7.21
3.57
3.56
3.55
3.54
2.85
2.83
2.82
2.81
2.80
2.79
2.00
1.99
1.98
1.97
1.94 cd3cn
d
a, a'
c, c'
b, b'
1.91
7.83
4.00
4.00
7.98
CN
Cl-
2
f1 (ppm)

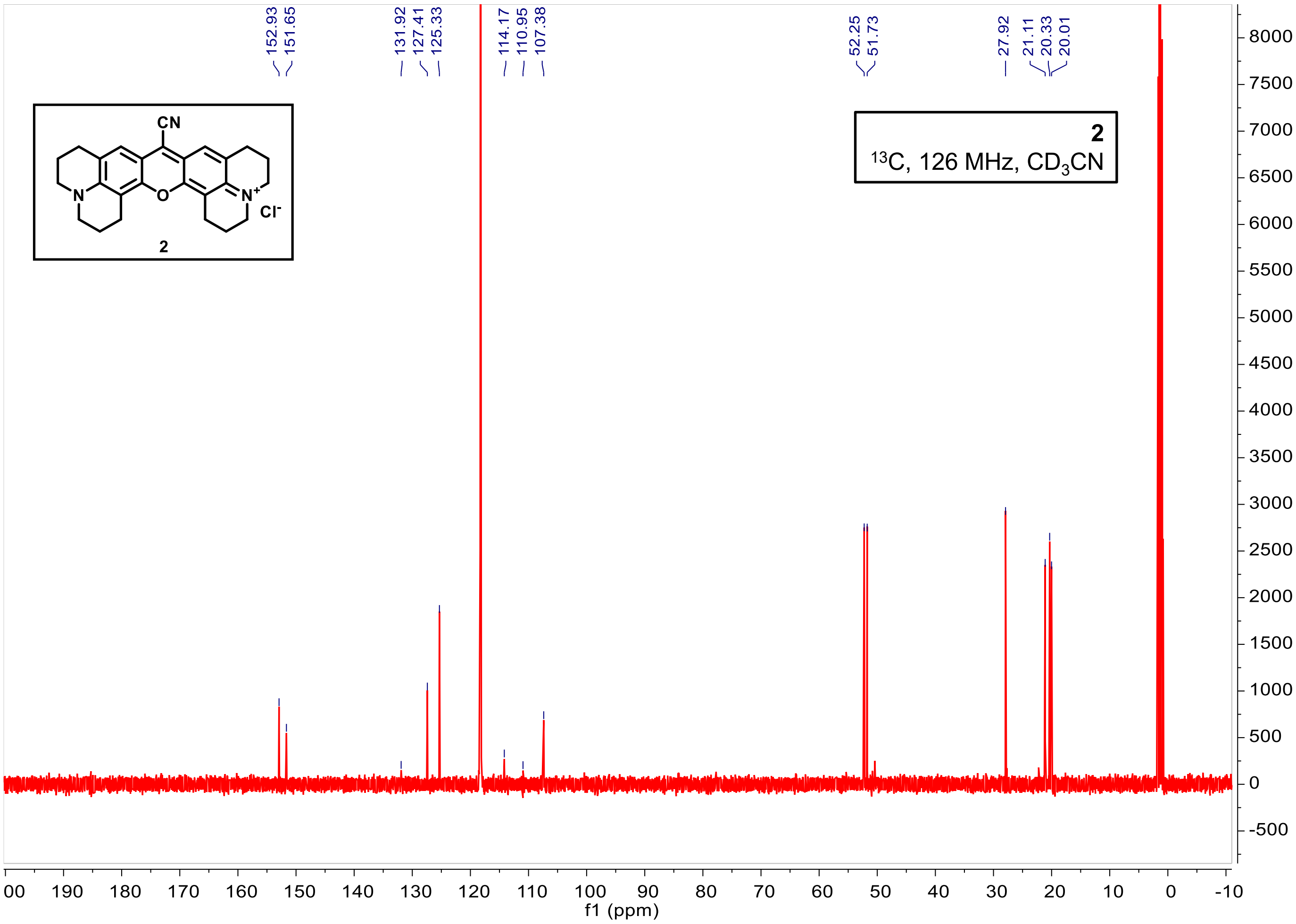
2
¹³C, 126 MHz, CD3CN
152.93
151.65
131.92
127.41
125.33
114.17
110.95
107.38
52.25
51.73
27.92
21.11
20.33
20.01
CN
Cl-
2
f1 (ppm)

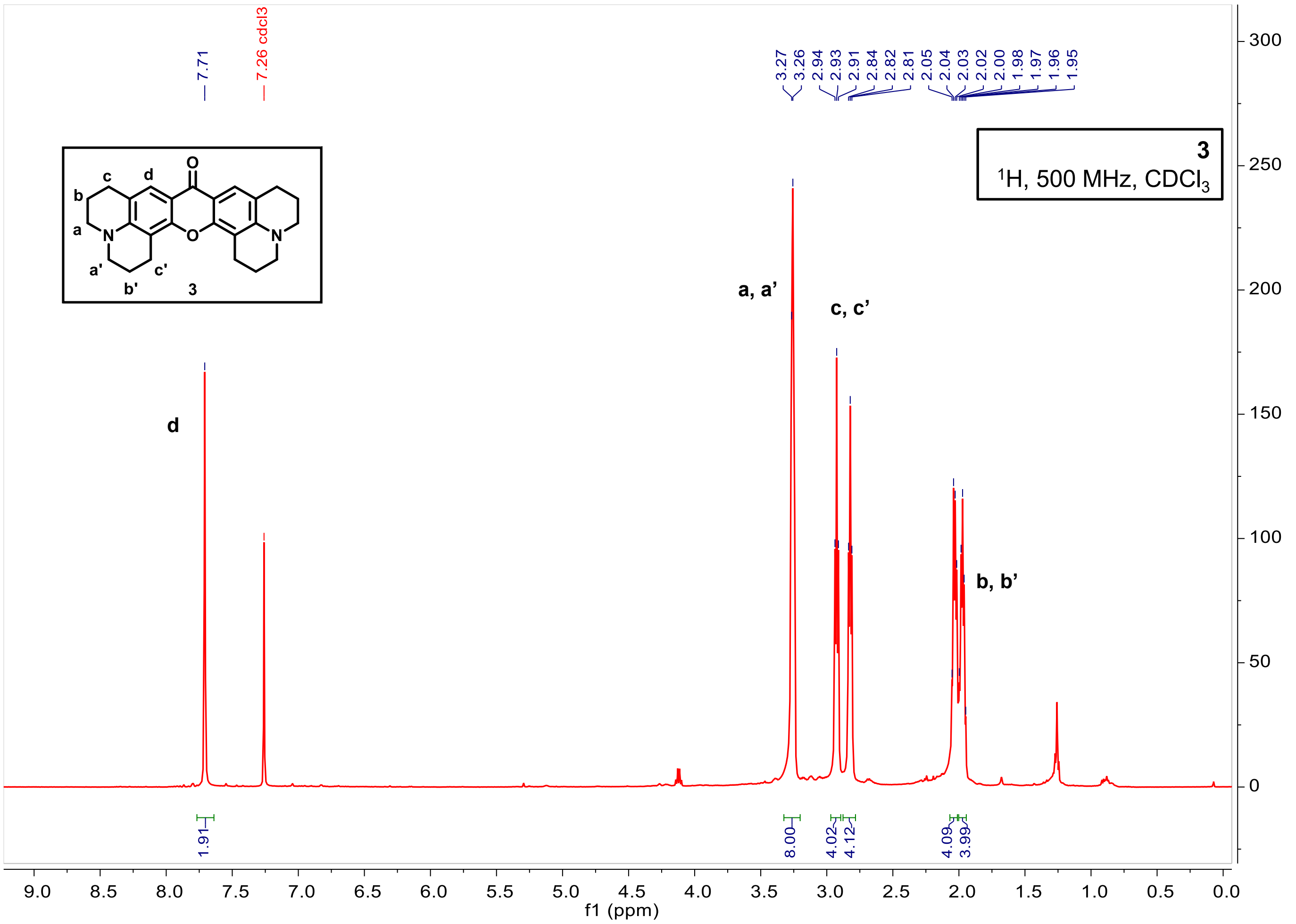
7.71
7.26 cdcl3
3.27
3.26
2.94
2.93
2.91
2.84
2.82
2.81
2.05
2.04
2.03
2.02
2.00
1.98
1.97
1.96
1.95
3
^{1}H, 500 MHz, $CDCl_3$
a, a'
c, c'
b, b'
d
1.91
8.00
4.02
4.12
4.09
3.99
f1 (ppm)

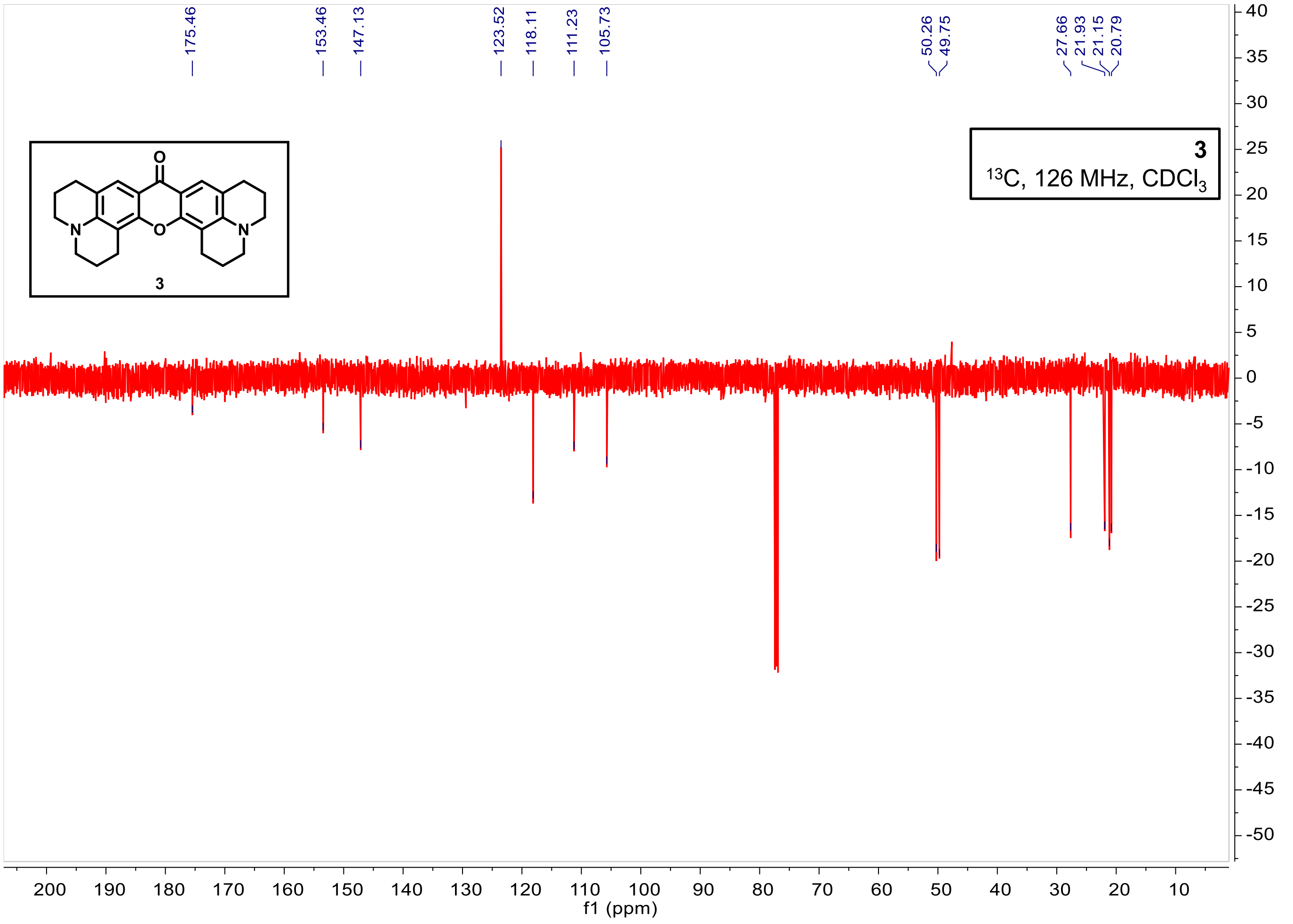
175.46
153.46
147.13
123.52
118.11
111.23
105.73
50.26
49.75
27.66
21.93
21.15
20.79
3
^{13}C, 126 MHz, $CDCl_3$
f1 (ppm)

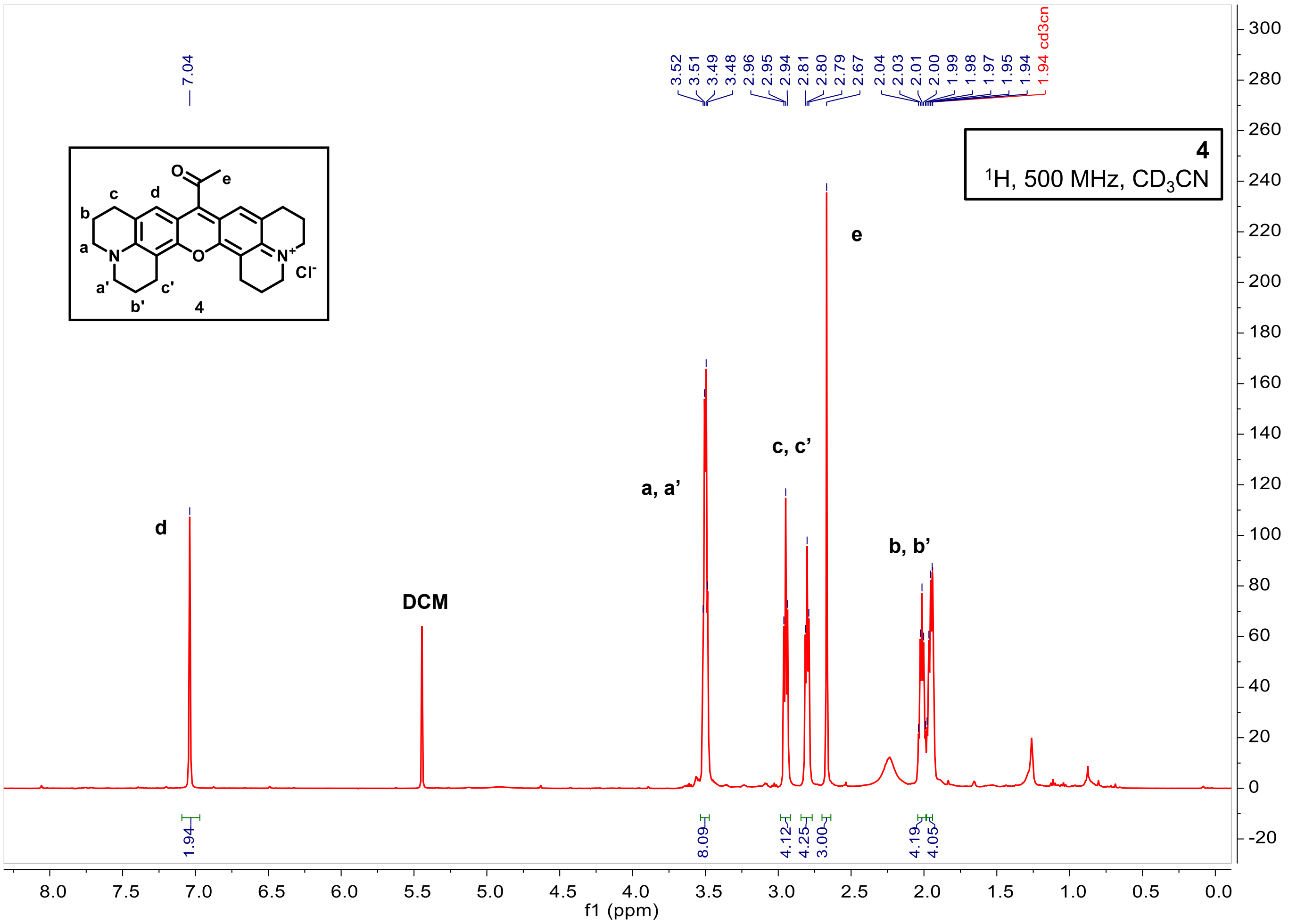
4
^{1}H, 500 MHz, CD_3CN
d
DCM
a, a'
c, c'
e
b, b'
f1 (ppm)
7.04
3.52 3.51 3.49 3.48 2.96 2.95 2.94 2.81 2.80 2.79 2.67 2.04 2.03 2.01 2.00 1.99 1.98 1.97 1.95 1.94 1.94 cd3cn
1.94 8.09 4.12 4.25 3.00 4.19 4.05

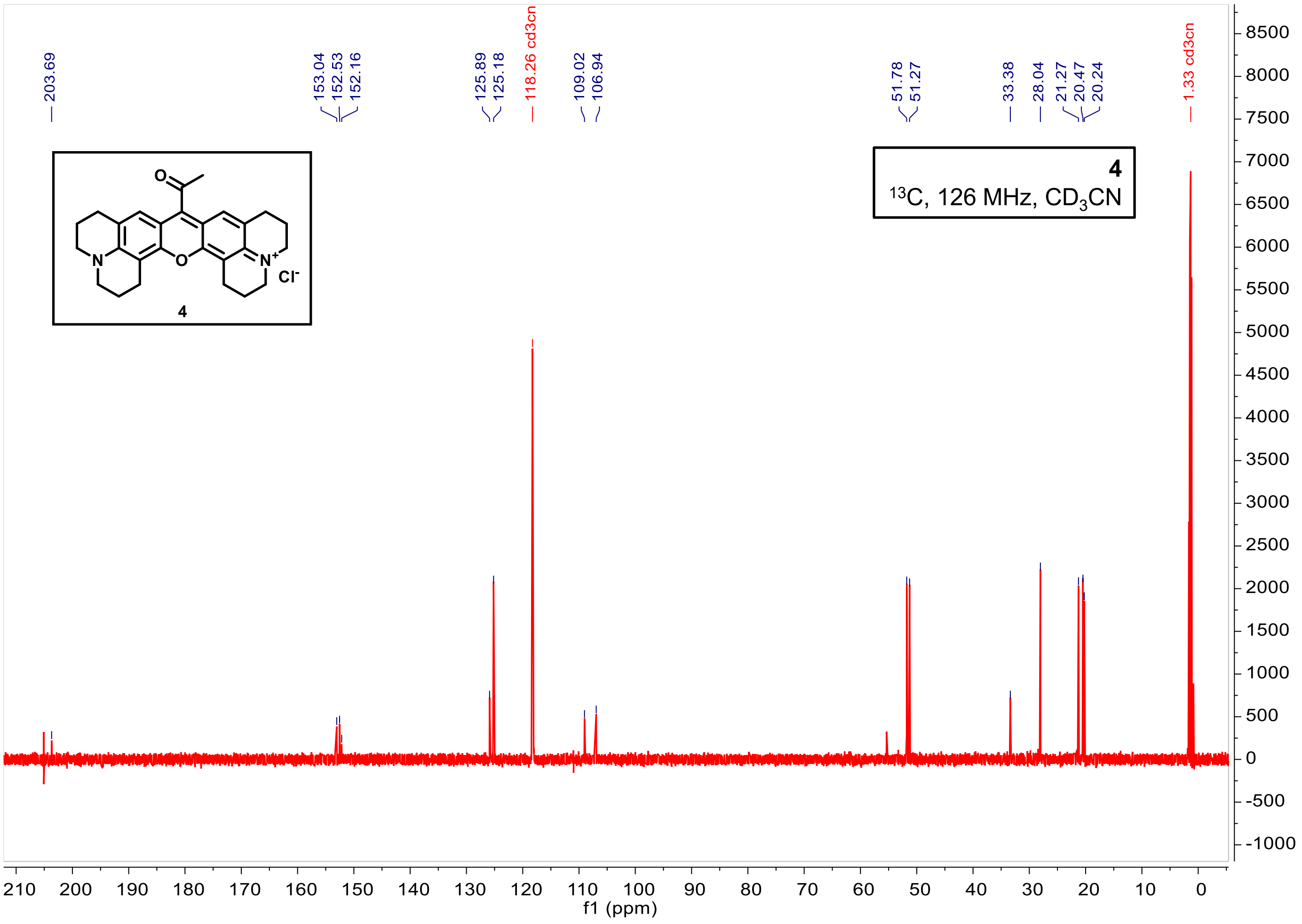
4
^{13}C, 126 MHz, CD_3CN
203.69
153.04 152.53 152.16
125.89 125.18 118.26 cd3cn
109.02 106.94
51.78 51.27
33.38 28.04 21.27 20.47 20.24
1.33 cd3cn
f1 (ppm)

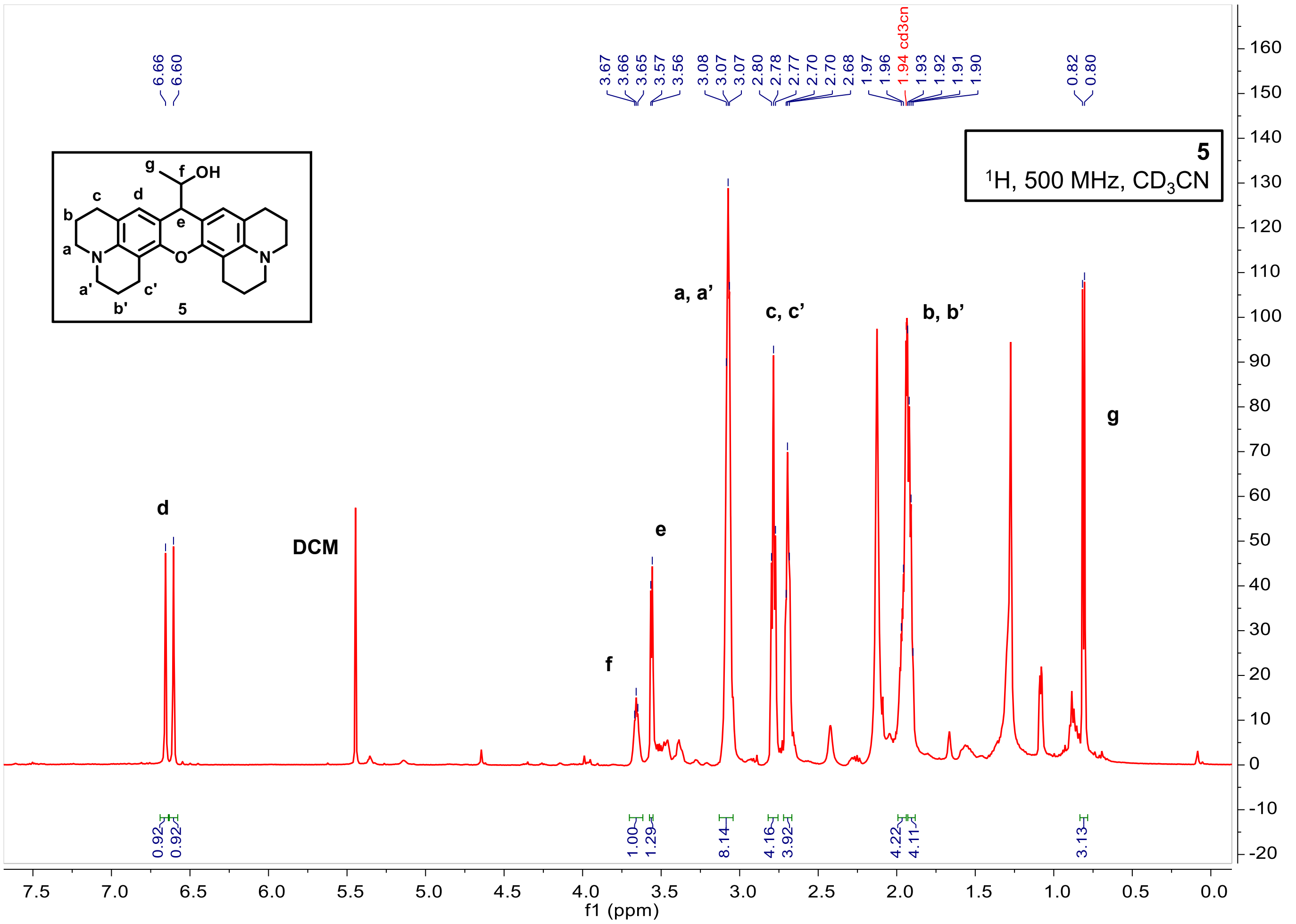
5
¹H, 500 MHz, CD₃CN
d
DCM
e
f
a, a'
c, c'
b, b'
g
f1 (ppm)
cd3cn

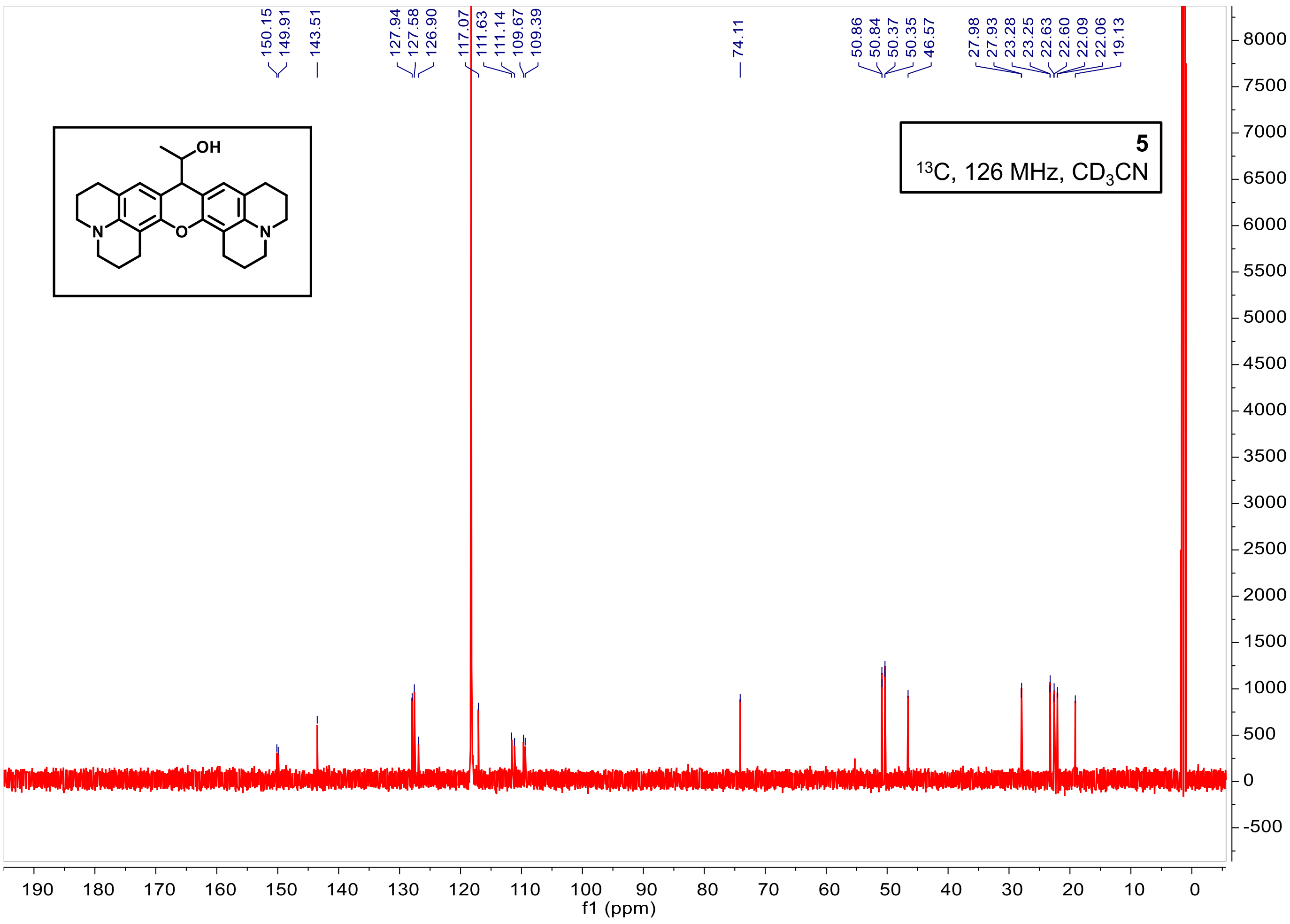
5
¹³C, 126 MHz, CD₃CN
f1 (ppm)

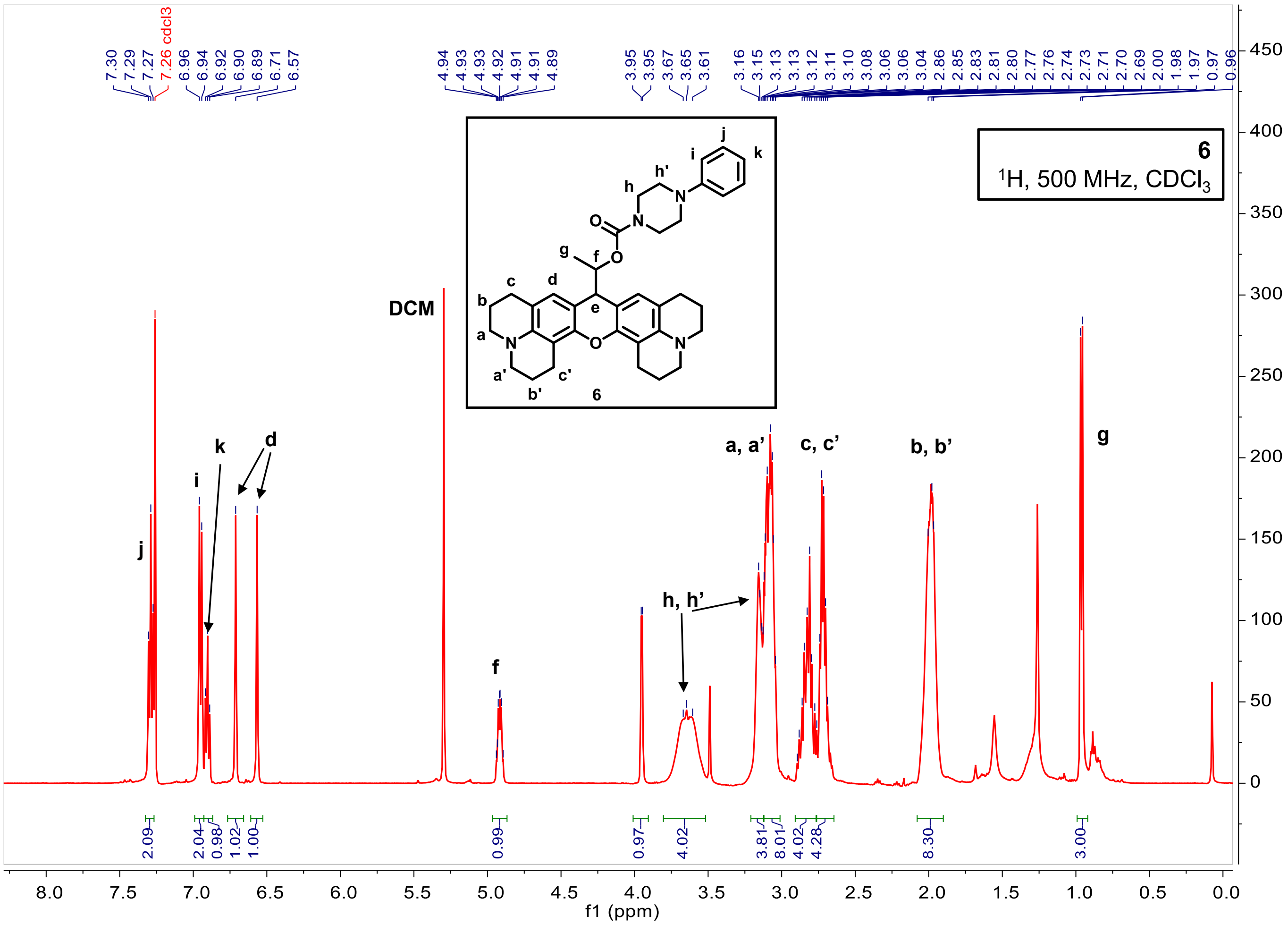
6
1H, 500 MHz, CDCl3
DCM
j
i
k
d
f
h, h'
a, a'
c, c'
b, b'
g
f1 (ppm)

Chromatogram

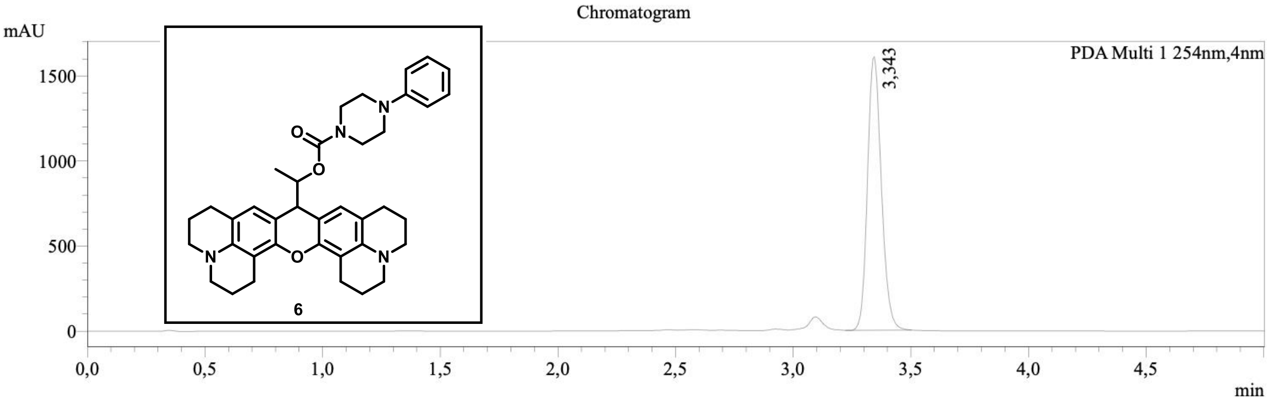

Peak Table

PDA Ch1 254nm

Peak#	Ret. Time	Peak Start	Peak End	Area	Height	Area/Height
1	3,343	3,221	3,504	6583628	1607654	4,095
Total				6583628	1607654	

Contour

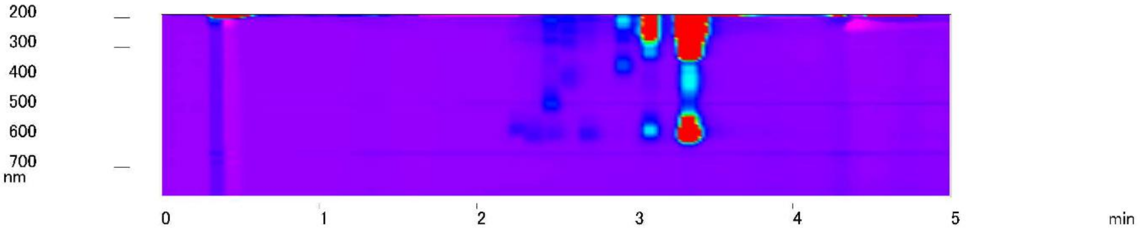

MS Chromatogram

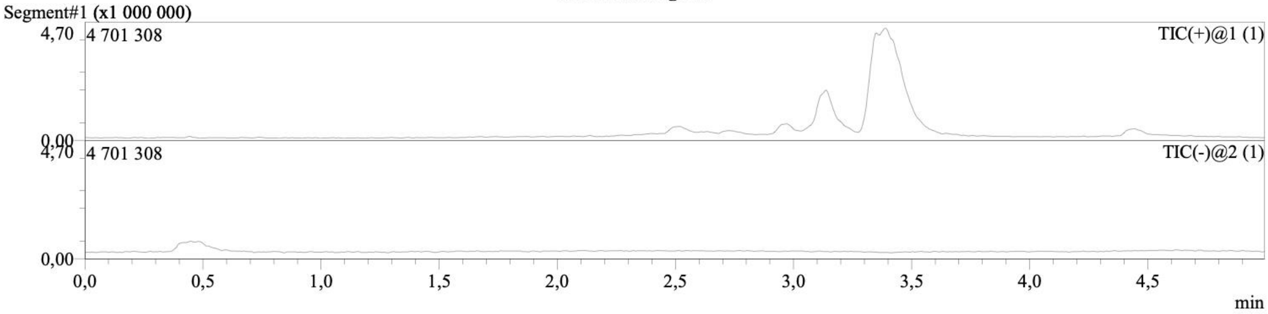

MS Spectrum

Line#:1 R.Time:----(Scan#:----)
MassPeaks:848
Spectrum Mode:Averaged 3,267-3,640(981-1093) Base Peak:603(271562)
BG Mode:Averaged 0,947-1,427(285-429) Segment 1 - Event 1

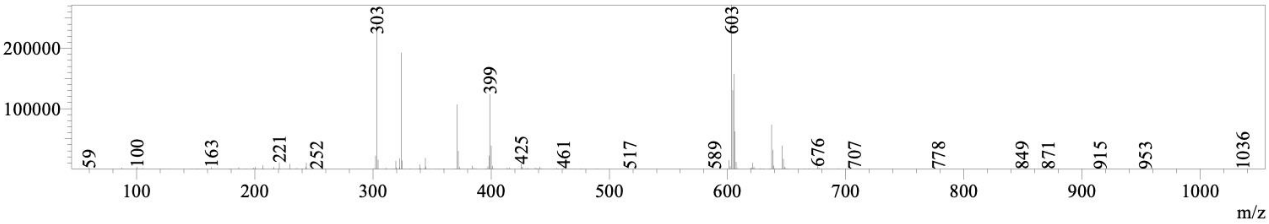

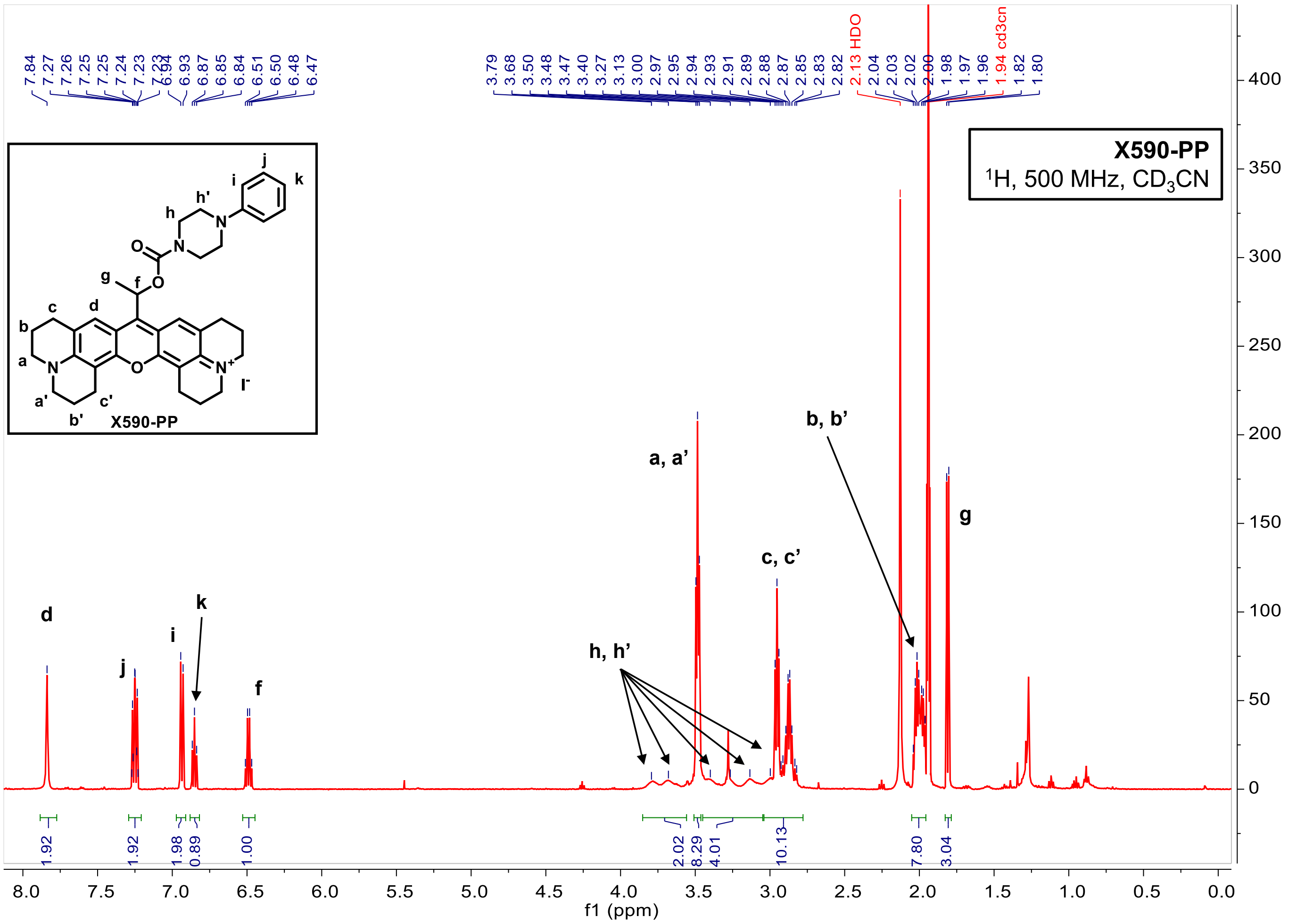

X590-PP
^{1}H, 500 MHz, CD_3CN
7.84 7.27 7.26 7.25 7.25 7.24 7.23 7.23 6.94 6.93 6.87 6.85 6.84 6.51 6.50 6.48 6.47
3.79 3.68 3.50 3.48 3.47 3.40 3.27 3.13 3.00 2.97 2.95 2.94 2.93 2.91 2.89 2.88 2.87 2.85 2.83 2.82
2.13 HDO
2.04 2.03 2.02 2.00 1.98 1.97 1.96
1.94 cd3cn
1.82 1.80
d
j
i
k
f
h, h'
a, a'
c, c'
b, b'
g
1.92 1.92 1.98 0.89 1.00 2.02 8.29 4.01 10.13 7.80 3.04
X590-PP
f1 (ppm)

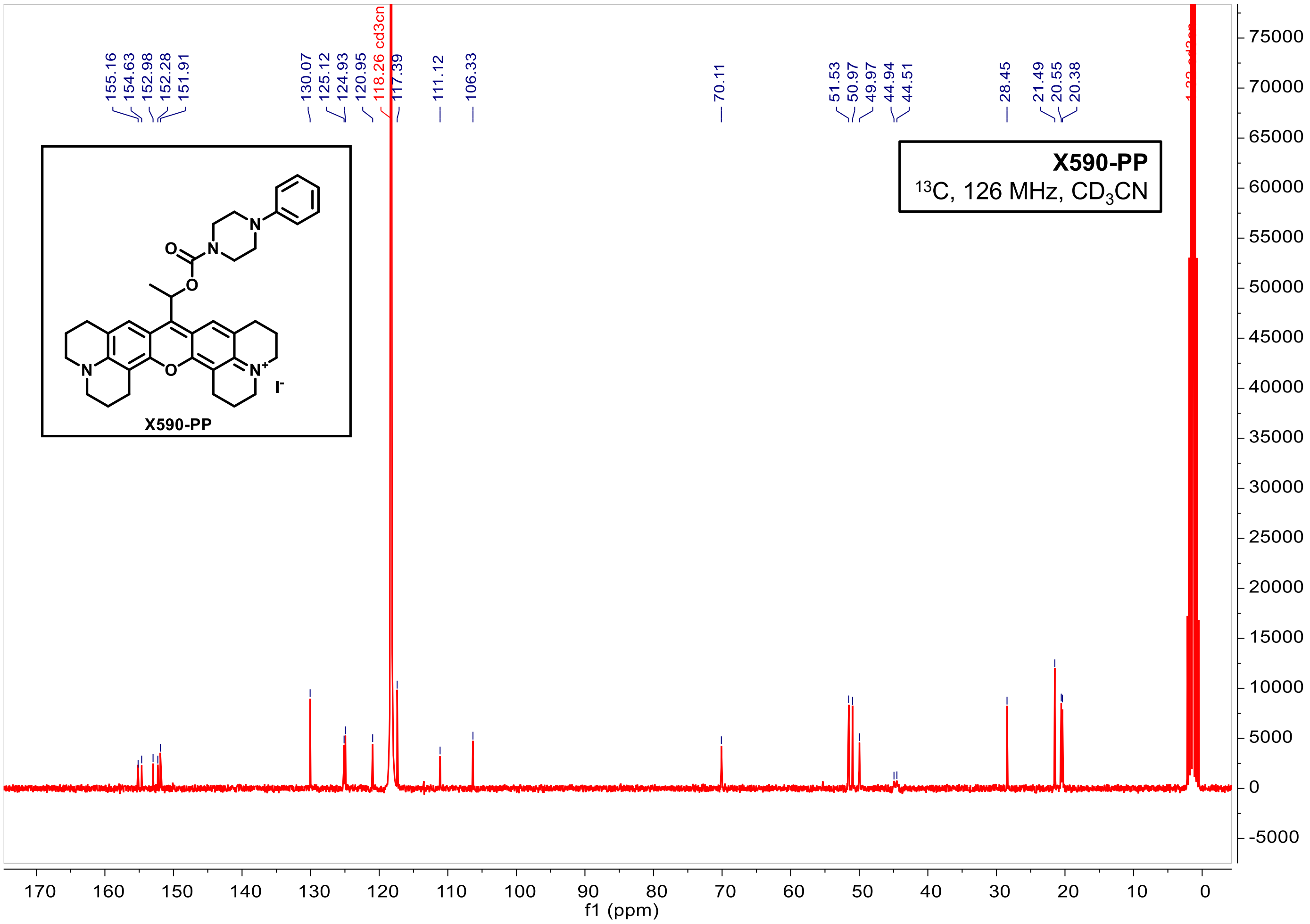

X590-PP
^{13}C, 126 MHz, CD_3CN
155.16 154.63 152.98 152.28 151.91
130.07 125.12 124.93 120.95
118.26 cd3cn
117.39 111.12 106.33
70.11
51.53 50.97 49.97 44.94 44.51
28.45 21.49 20.55 20.38
X590-PP
f1 (ppm)

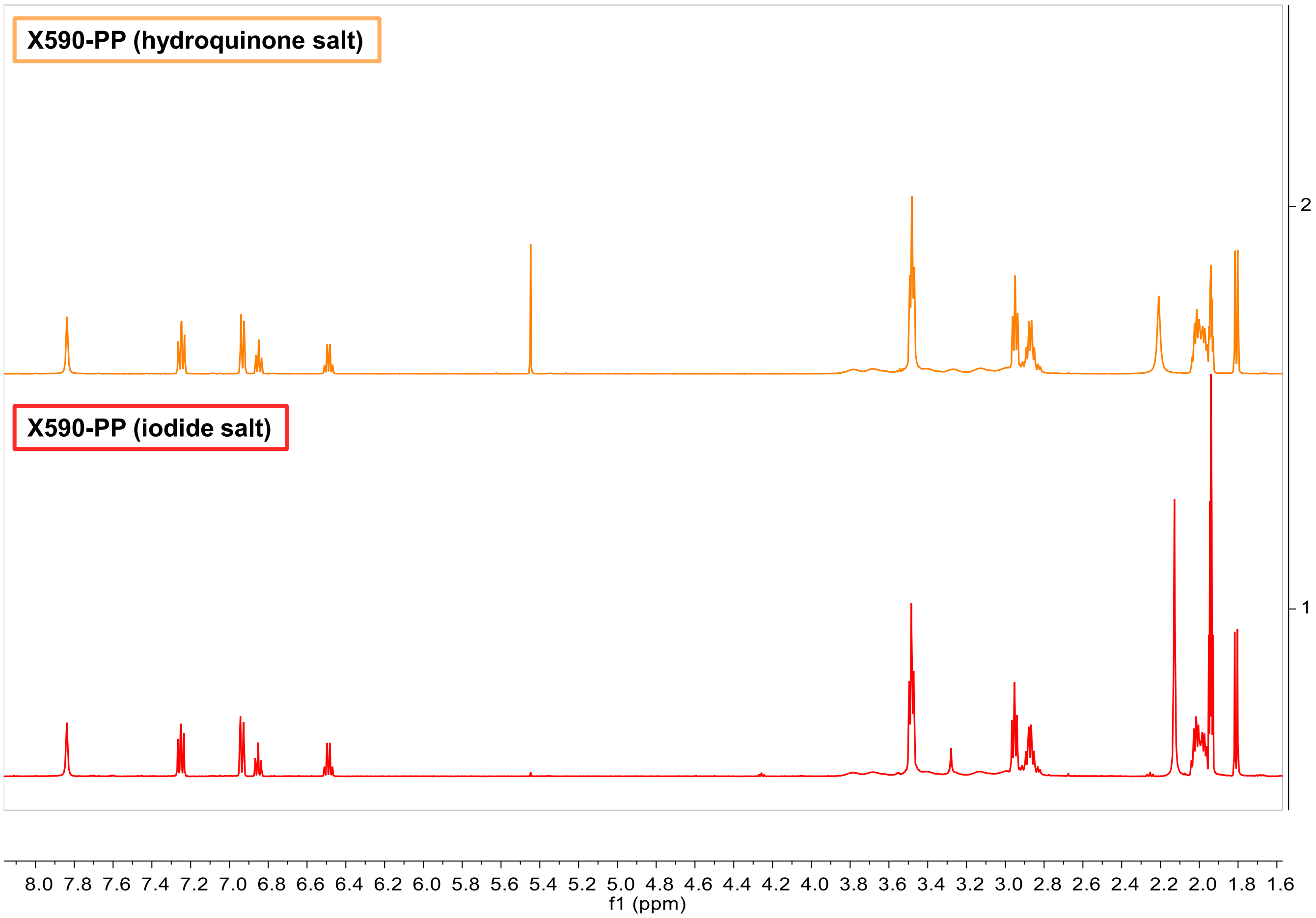
X590-PP (hydroquinone salt)
X590-PP (iodide salt)
2
1
8.0 7.8 7.6 7.4 7.2 7.0 6.8 6.6 6.4 6.2 6.0 5.8 5.6 5.4 5.2 5.0 4.8 4.6 4.4 4.2 4.0 3.8 3.6 3.4 3.2 3.0 2.8 2.6 2.4 2.2 2.0 1.8 1.6
f1 (ppm)

Chromatogram

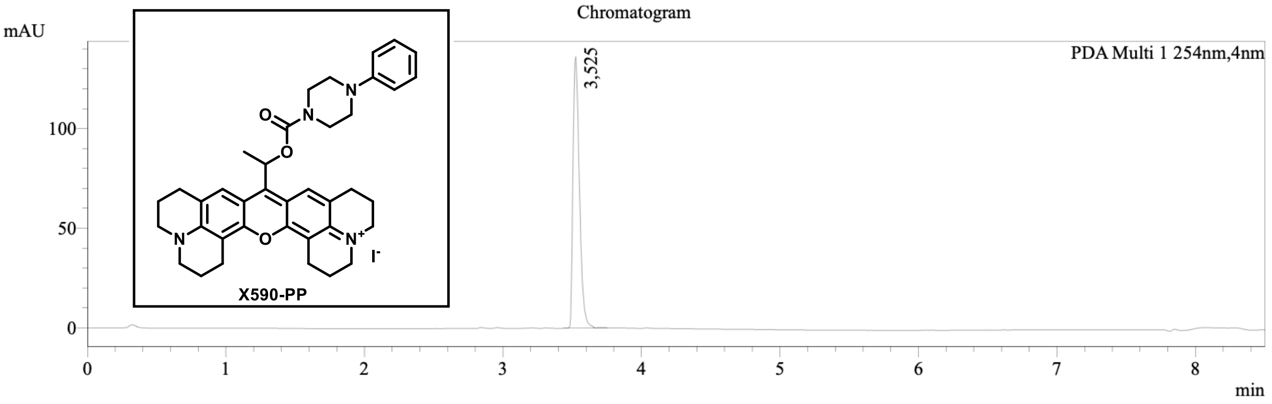

Peak Table

PDA Ch1 254nm

Peak#	Ret. Time	Peak Start	Peak End	Area	Height	Area/Height
1	3,525	3,435	3,755	440785	136335	3,233
Total				440785	136335	

Contour

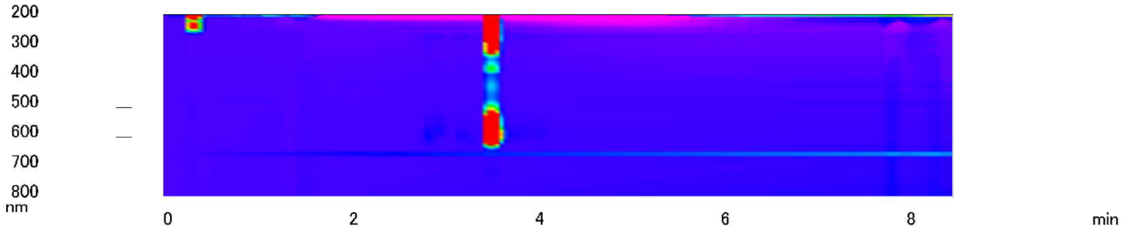

MS Chromatogram

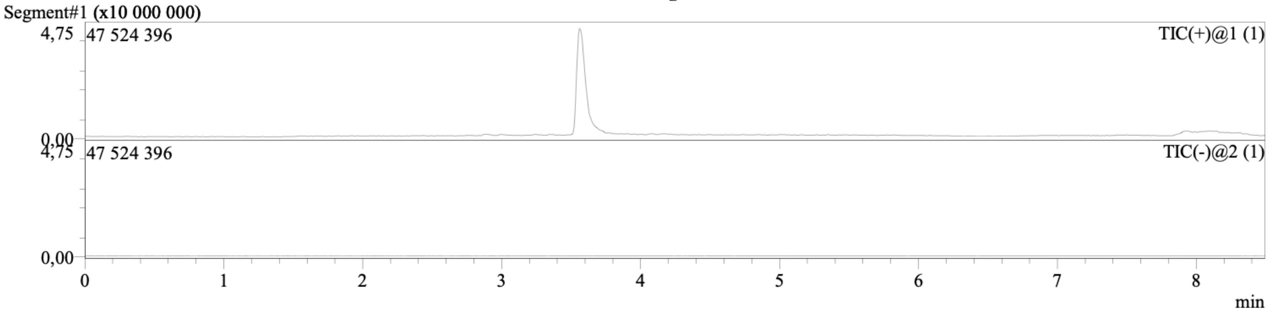

MS Spectrum

Line#:1 R.Time:----(Scan#:----)
MassPeaks:775
Spectrum Mode:Averaged 3,433-4,007(1031-1203) Base Peak:322(1654367)
BG Mode:Averaged 0,620-1,440(187-433) Segment 1 - Event 1

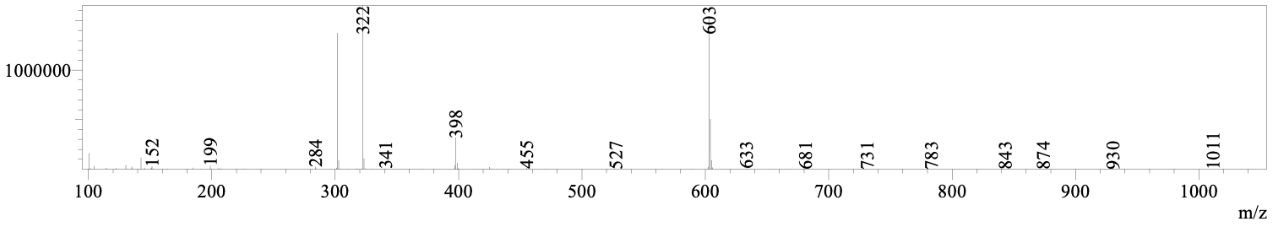

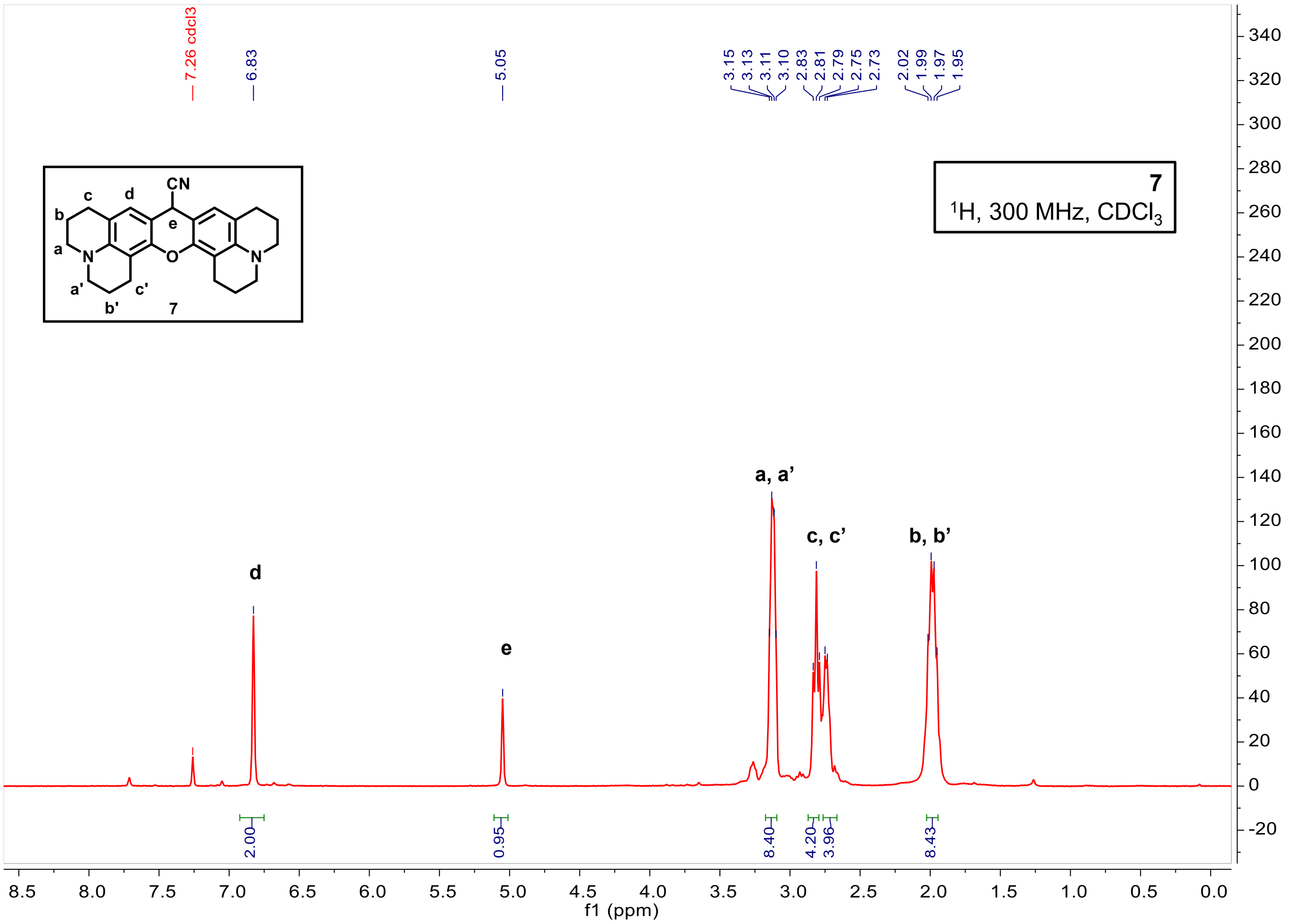
7.26 cdcl3
6.83
5.05
3.15
3.13
3.11
3.10
2.83
2.81
2.79
2.75
2.73
2.02
1.99
1.97
1.95
7
¹H, 300 MHz, CDCl3
d
e
a, a'
c, c'
b, b'
2.00
0.95
8.40
4.20
3.96
8.43
f1 (ppm)

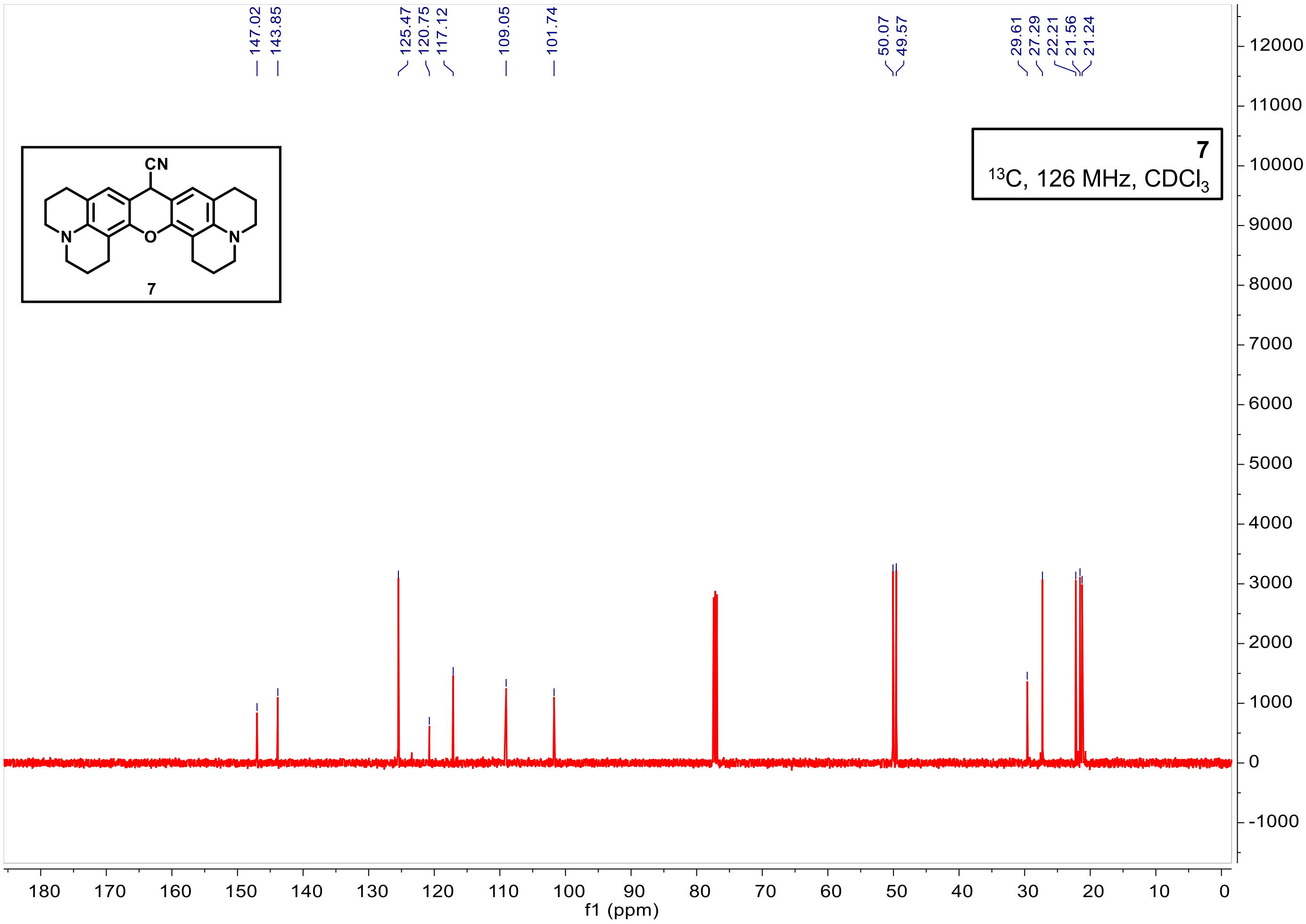
147.02
143.85
125.47
120.75
117.12
109.05
101.74
50.07
49.57
29.61
27.29
22.21
21.56
21.24
7
13C, 126 MHz, CDCl3
f1 (ppm)

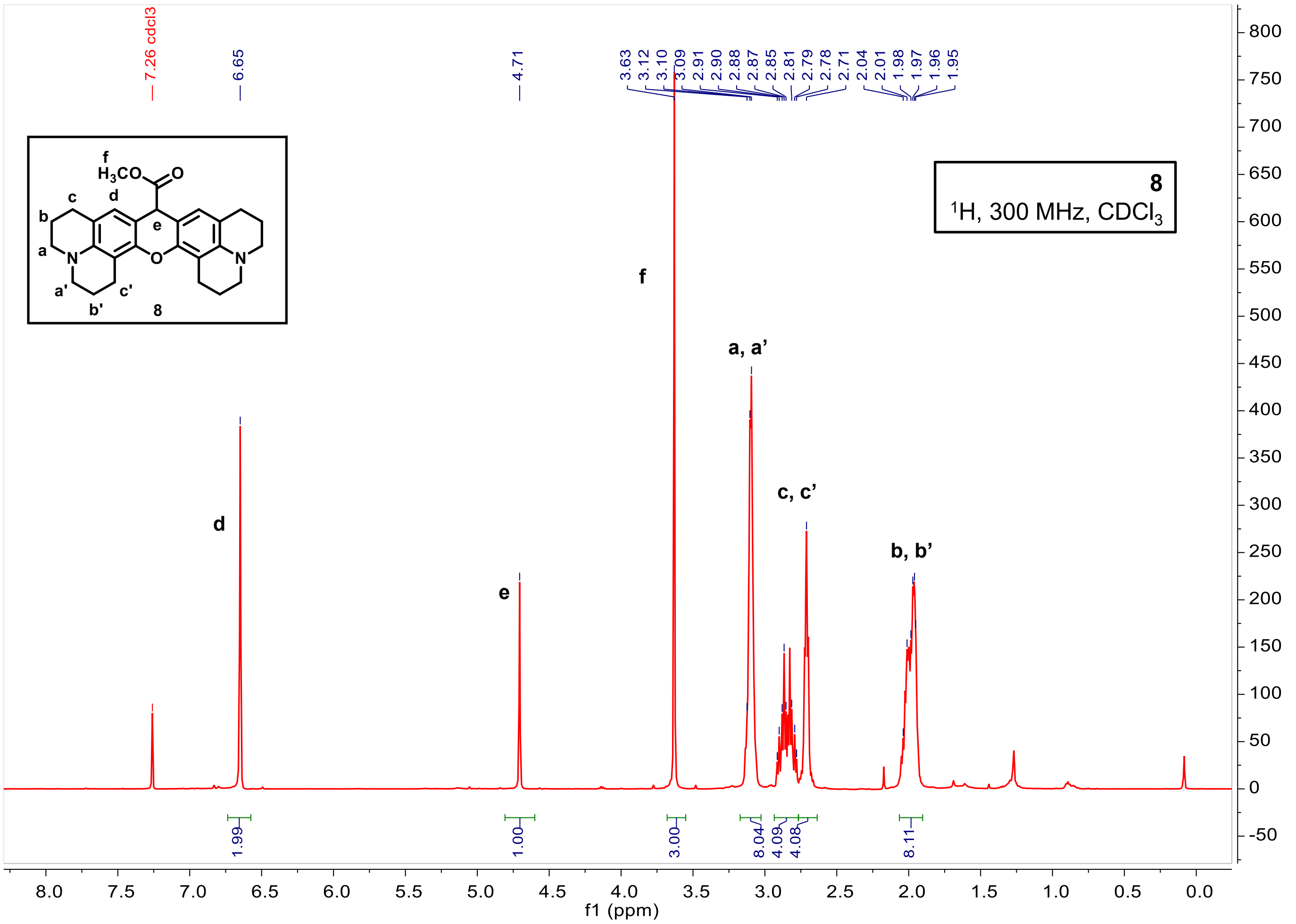
7.26 cdcl3
6.65
4.71
3.63
3.12
3.10
3.09
2.91
2.90
2.88
2.87
2.85
2.81
2.79
2.78
2.71
2.04
2.01
1.98
1.97
1.96
1.95
8
^{1}H, 300 MHz, CDCl$_3$
d
e
f
a, a'
c, c'
b, b'
1.99
1.00
3.00
8.04
4.09
4.08
8.11
f1 (ppm)

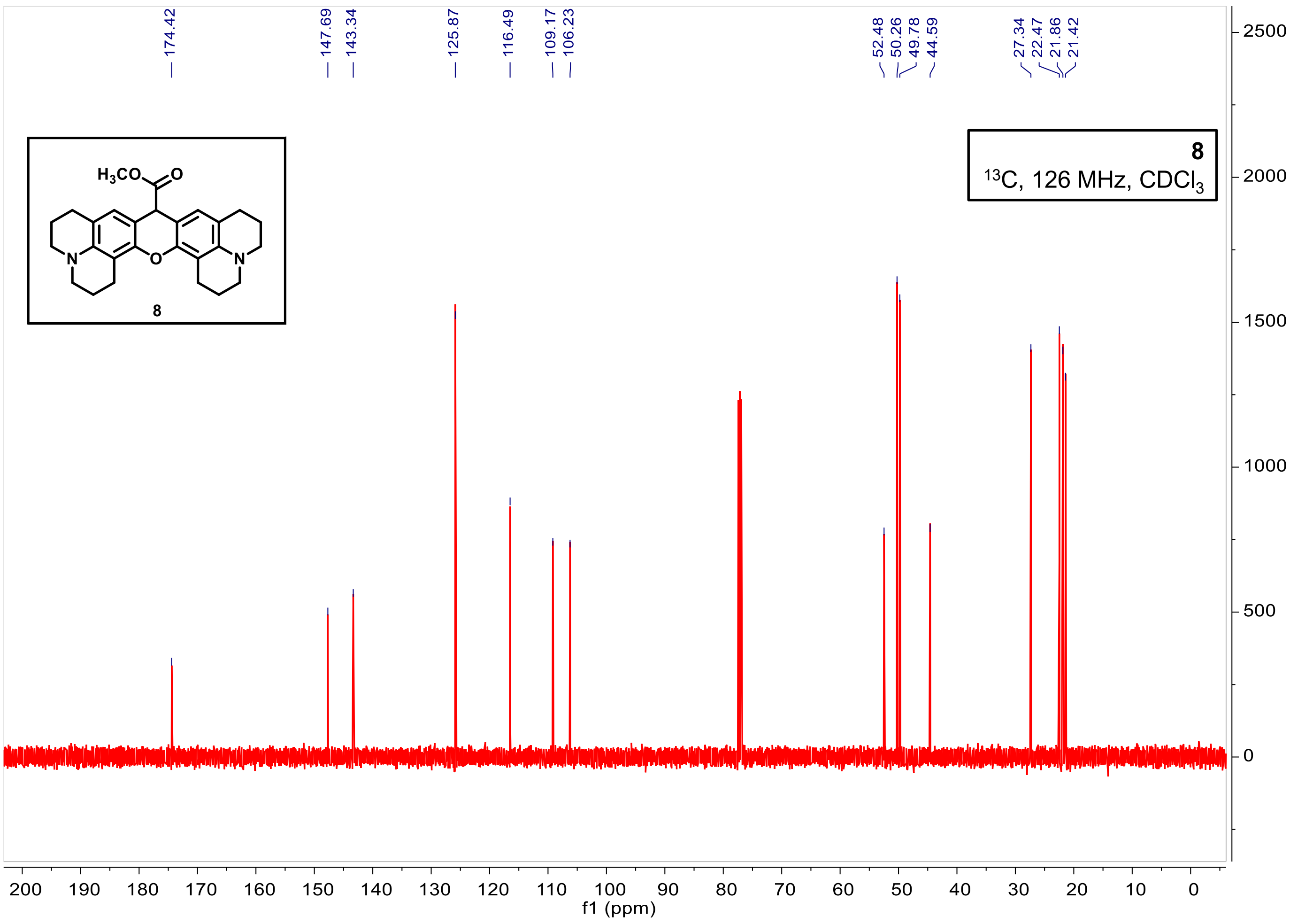
174.42
147.69
143.34
125.87
116.49
109.17
106.23
52.48
50.26
49.78
44.59
27.34
22.47
21.86
21.42
8
^{13}C, 126 MHz, CDCl$_3$
f1 (ppm)

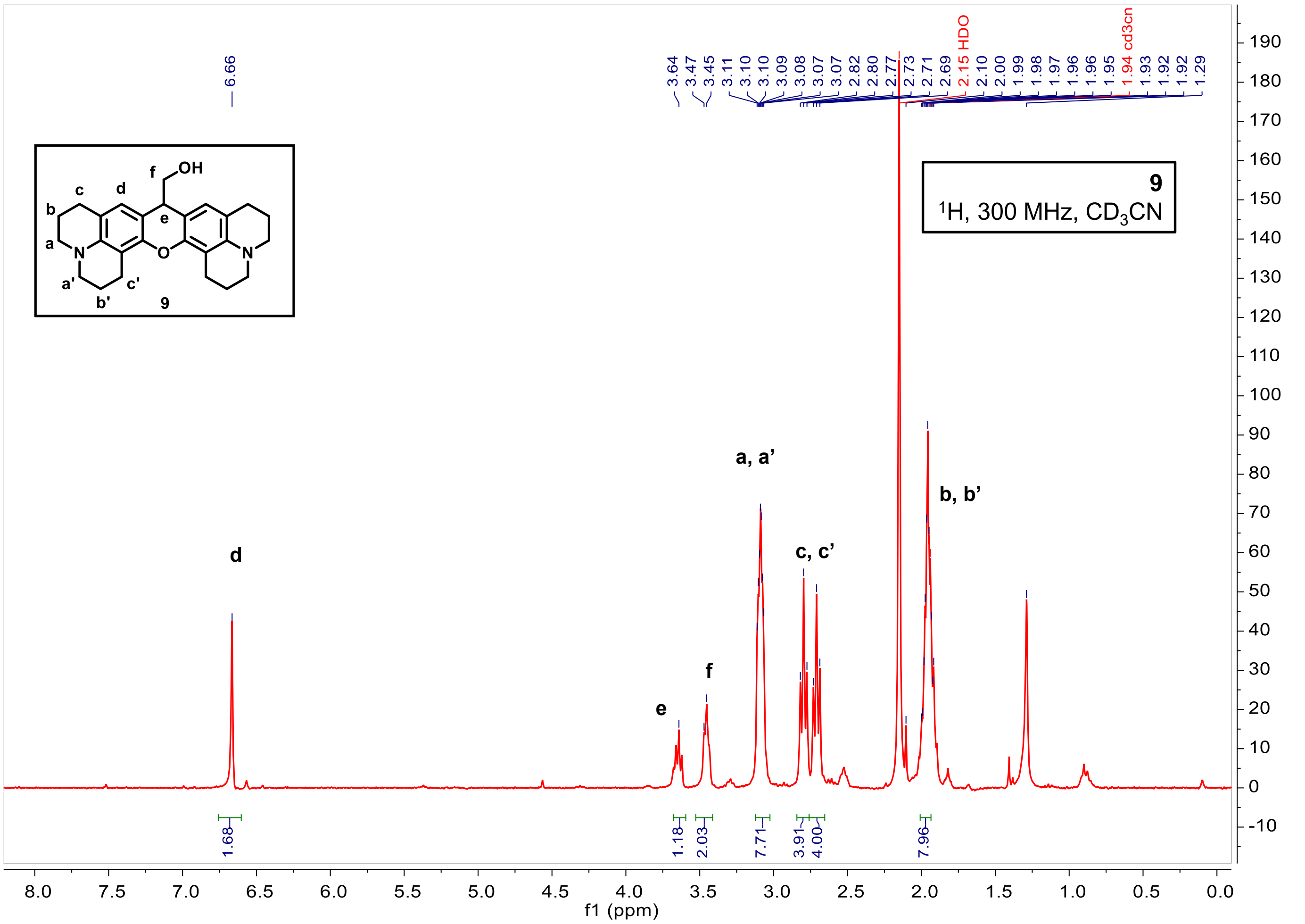

9
^{1}H, 300 MHz, CD_3CN
d
e
f
a, a'
c, c'
b, b'
f1 (ppm)

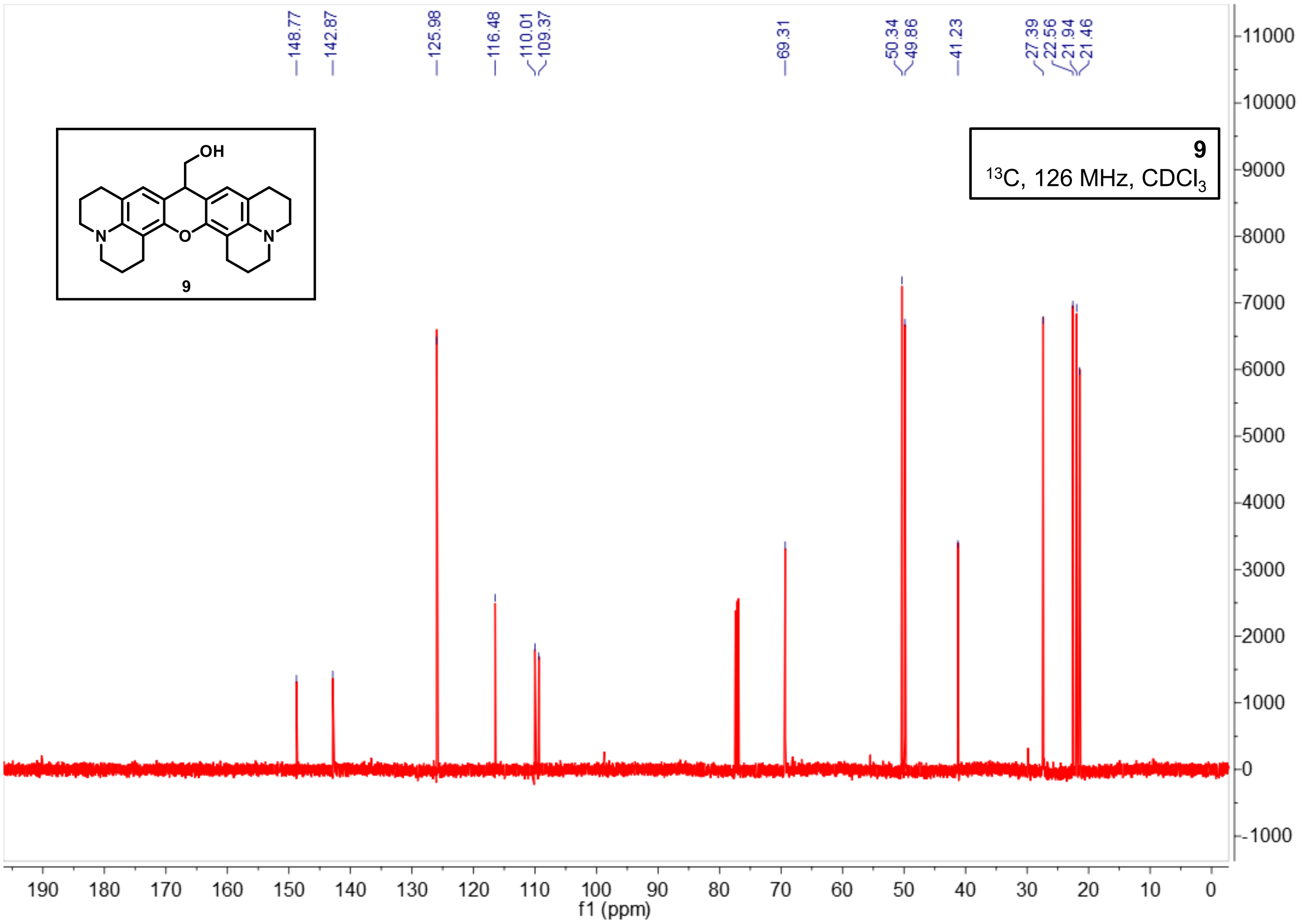

9
^{13}C, 126 MHz, $CDCl_3$
f1 (ppm)

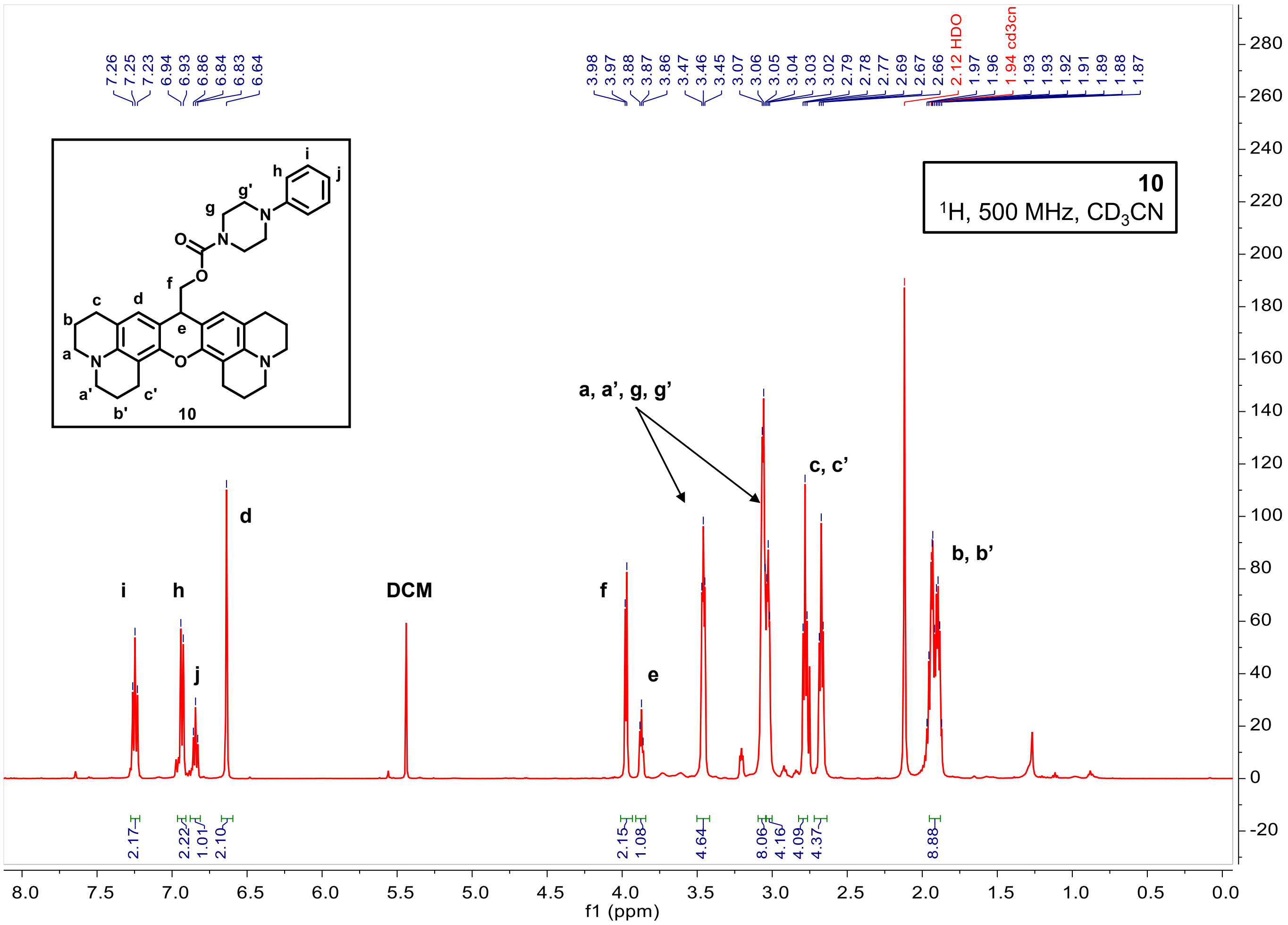

10
^{1}H, 500 MHz, CD_3CN
i
h
j
d
DCM
f
e
a, a', g, g'
c, c'
b, b'
f1 (ppm)

Chromatogram

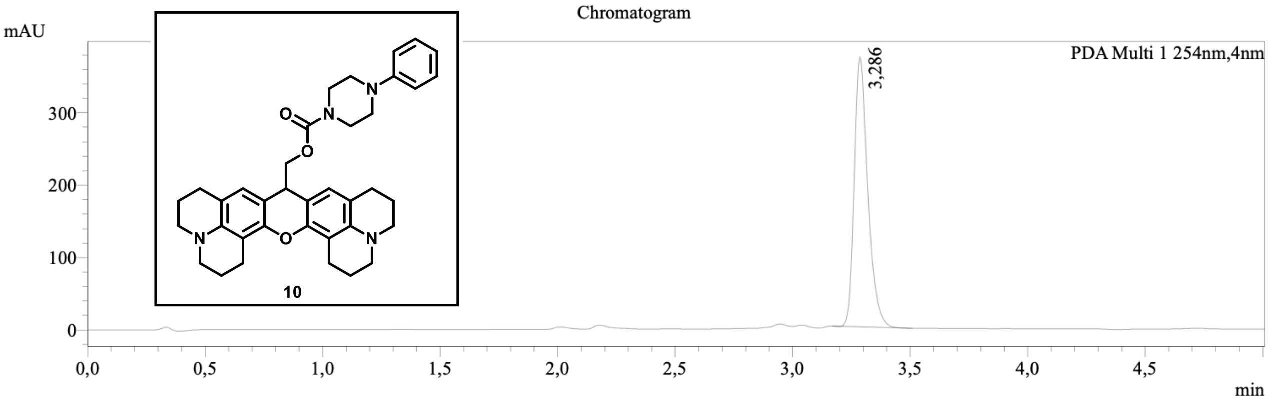

Peak Table

PDA Ch1 254nm

Peak#	Ret. Time	Peak Start	Peak End	Area	Height	Area/Height
1	3,286	3,168	3,509	1496152	373186	4,009
Total				1496152	373186	

Contour

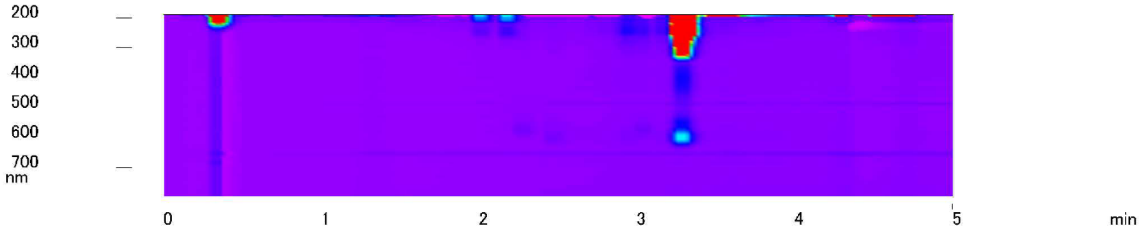

MS Chromatogram

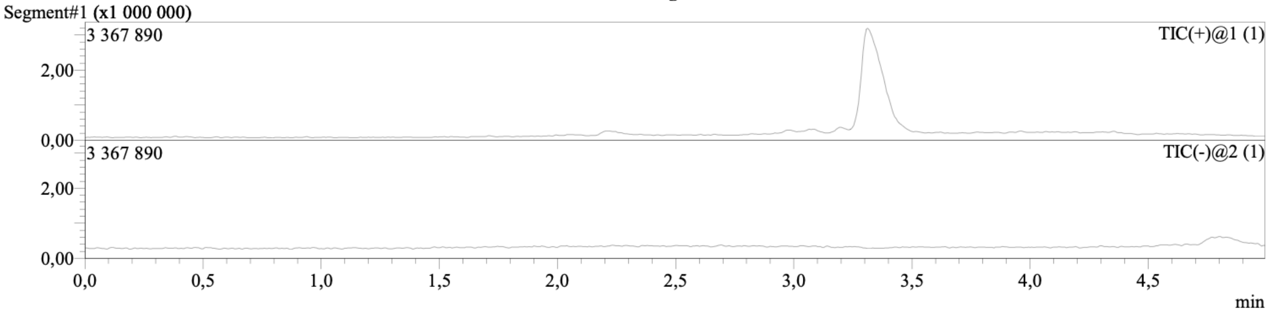

MS Spectrum

Line#:1 R.Time:----(Scan#:----)
MassPeaks:860
Spectrum Mode:Averaged 3,213-3,507(965-1053) Base Peak:296(193151)
BG Mode:Averaged 0,860-1,340(259-403) Segment 1 - Event 1

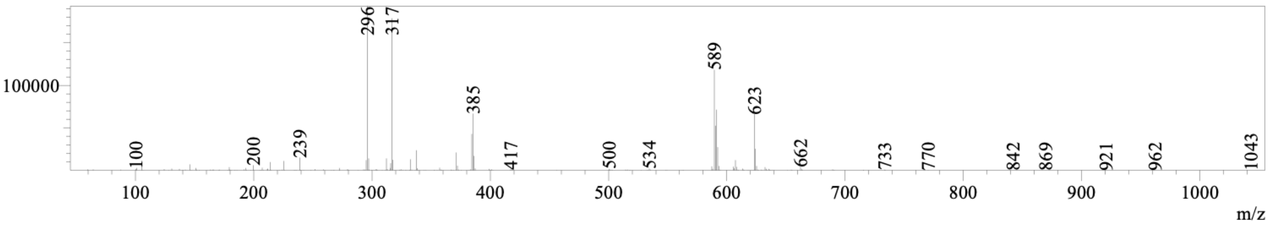

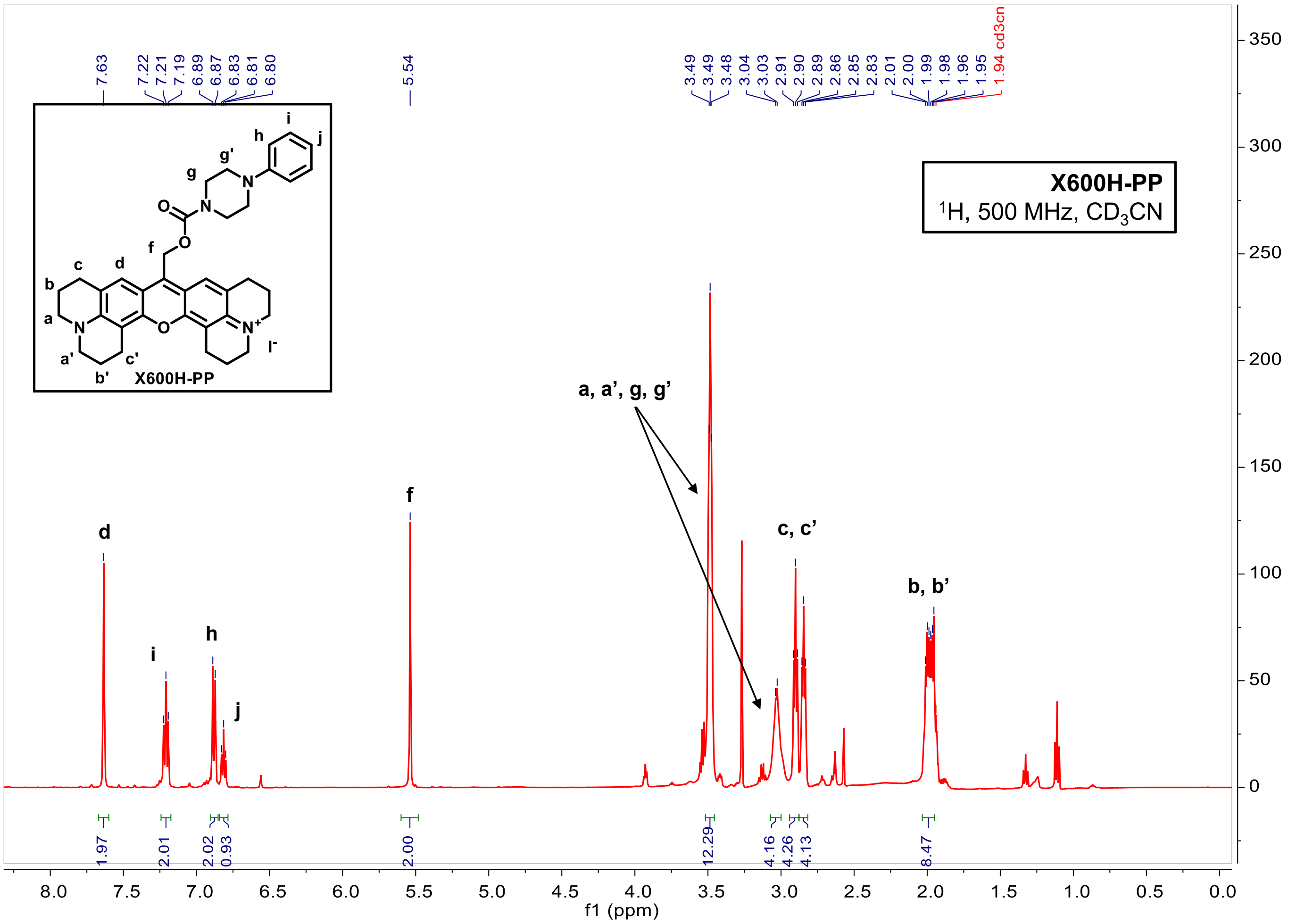
X600H-PP
1H, 500 MHz, CD3CN
d
i
h
j
f
a, a', g, g'
c, c'
b, b'
f1 (ppm)

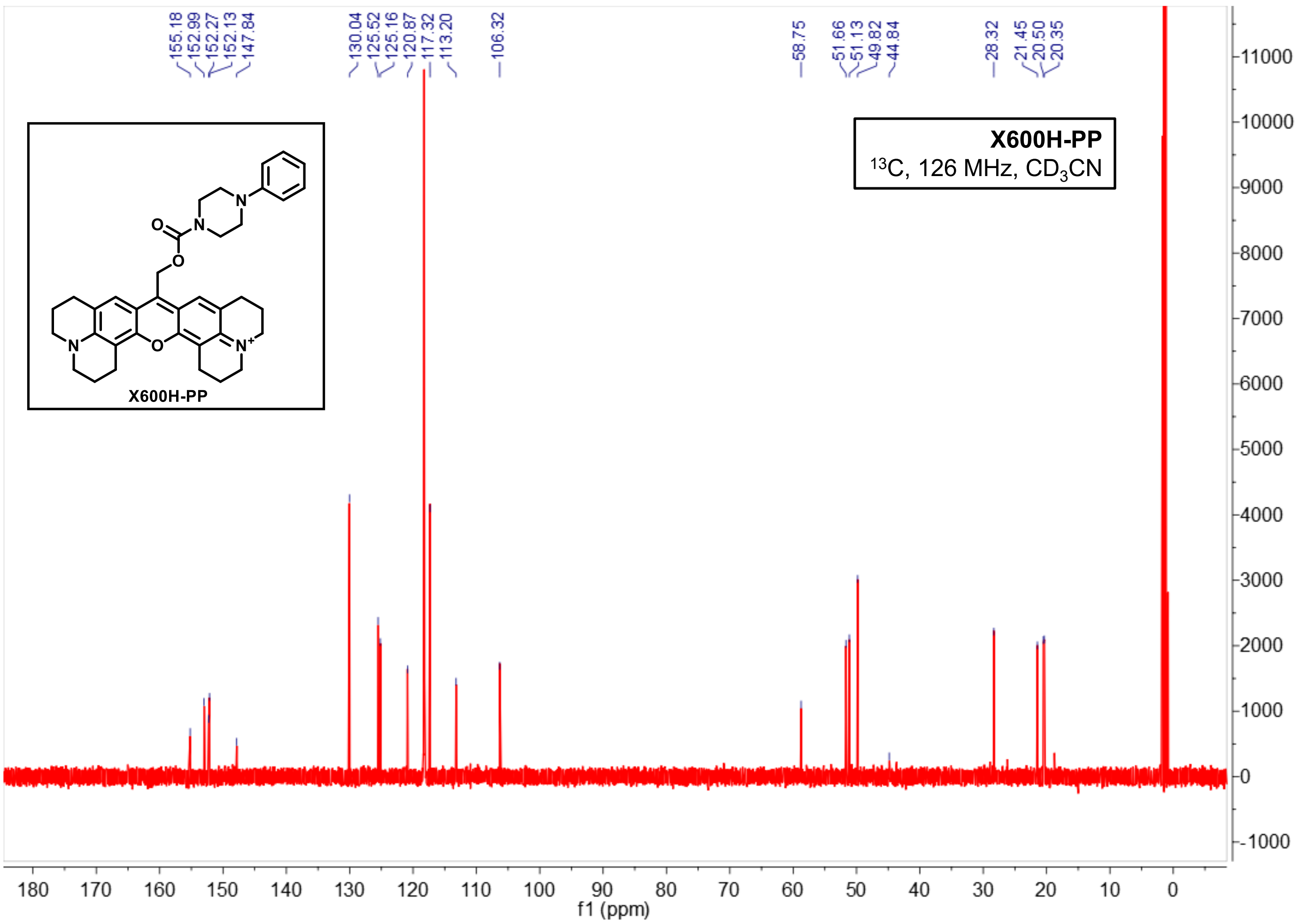
X600H-PP
13C, 126 MHz, CD3CN
f1 (ppm)

Chromatogram

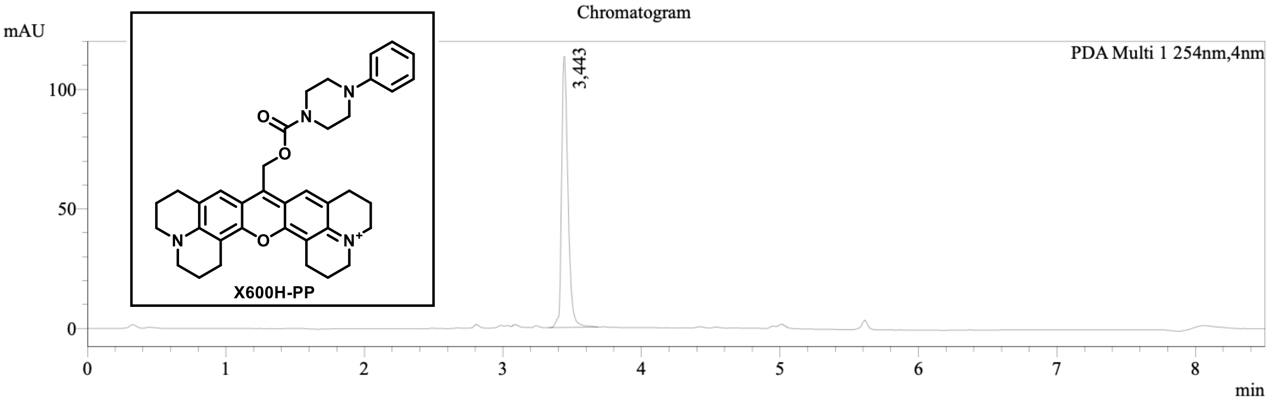

Peak Table

PDA Ch1 254nm

Peak#	Ret. Time	Peak Start	Peak End	Area	Height	Area/Height
1	3,443	3,328	3,685	363004	113269	3,205
Total				363004	113269	

Contour

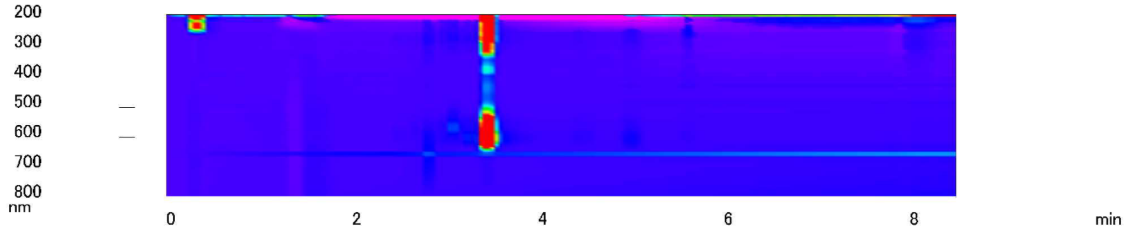

MS Chromatogram

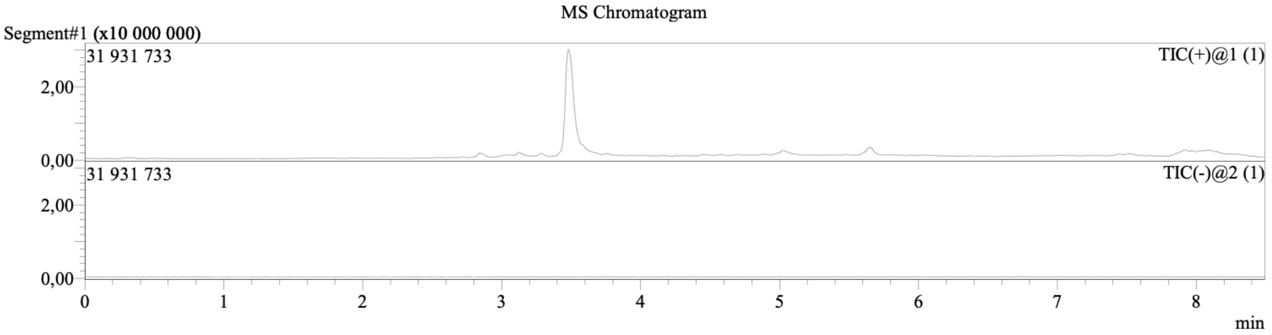

MS Spectrum

Line#:1 R.Time:----(Scan#:----)
MassPeaks:788
Spectrum Mode:Averaged 3,373-3,760(1013-1129) Base Peak:589(1960504)
BG Mode:Averaged 0,647-1,493(195-449) Segment 1 - Event 1

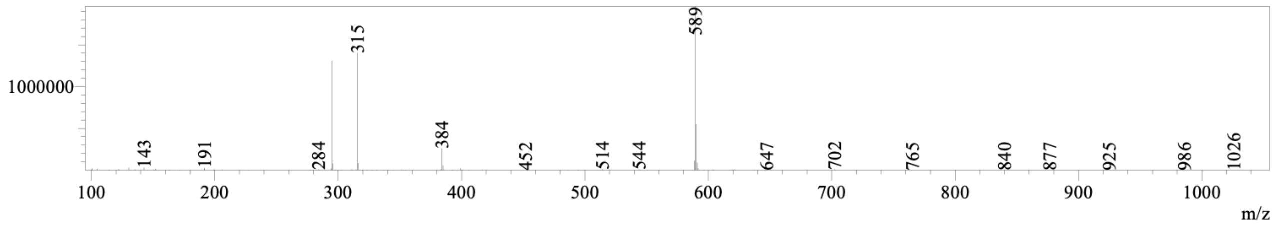

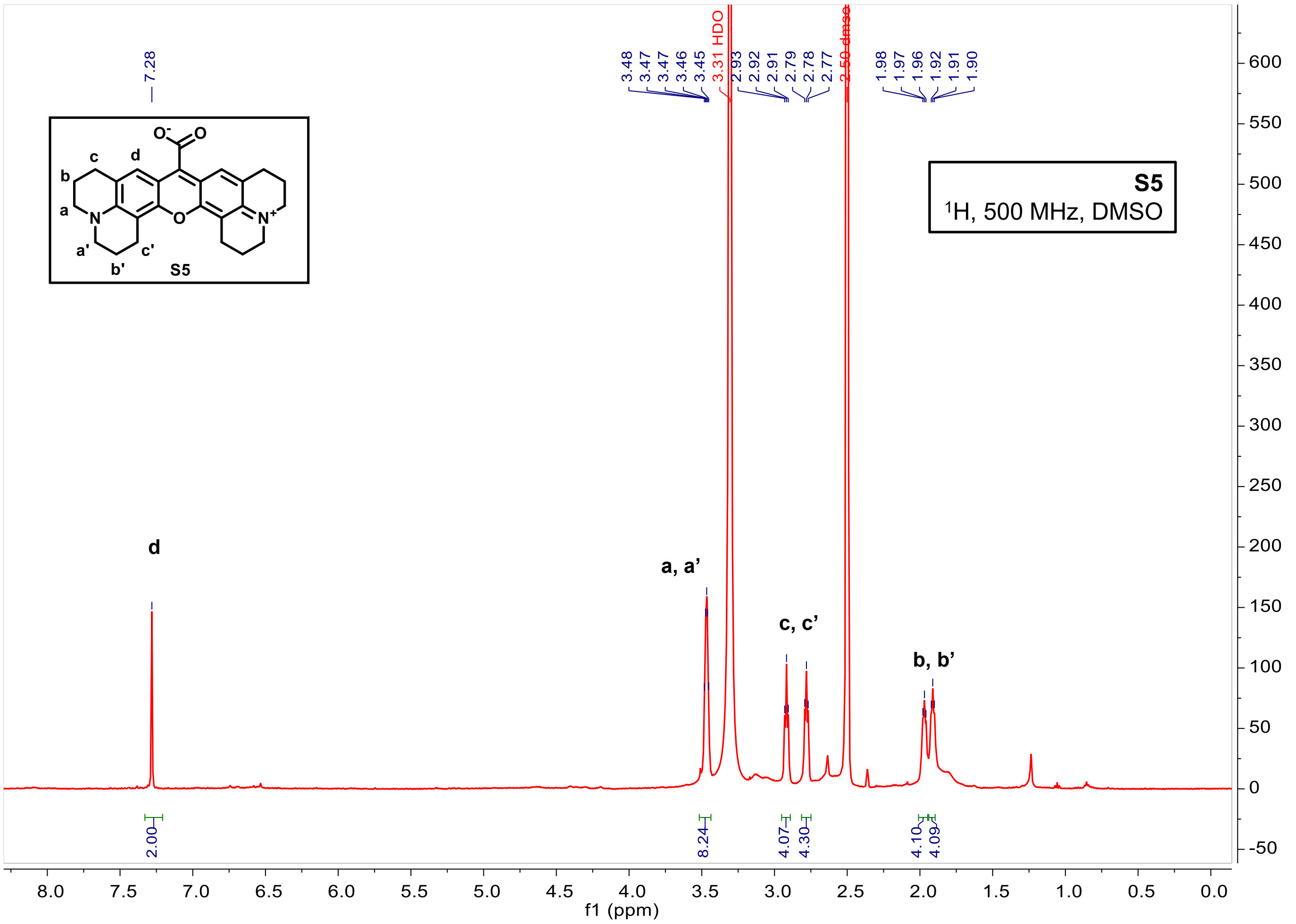

7.28
3.48
3.47
3.47
3.46
3.45
3.31 HDO
2.93
2.92
2.91
2.79
2.78
2.77
2.50 dmso
1.98
1.97
1.96
1.92
1.91
1.90
S5
^{1}H, 500 MHz, DMSO
d
a, a'
c, c'
b, b'
2.00
8.24
4.07
4.30
4.10
4.09
f1 (ppm)

Chromatogram

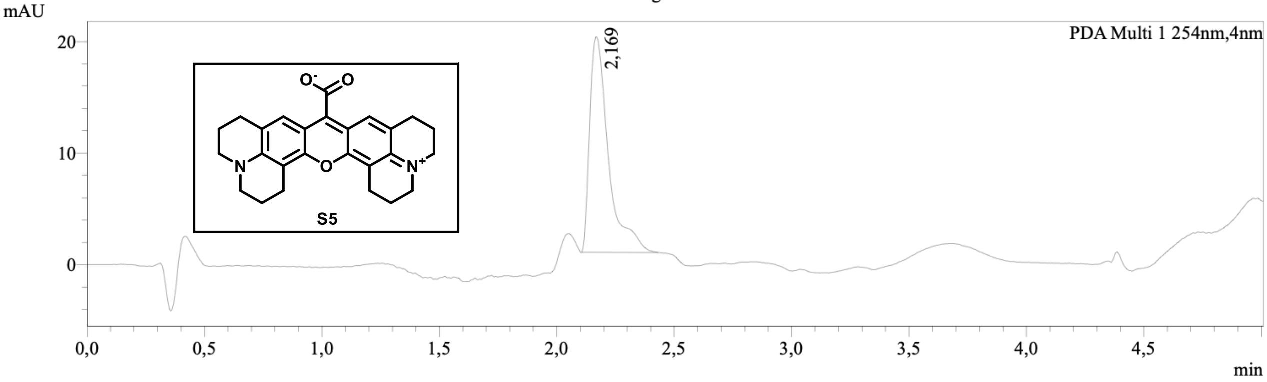

Peak Table

PDA Ch1 254nm

Peak#	Ret. Time	Peak Start	Peak End	Area	Height	Area/Height
1	2,169	2,107	2,432	107579	19320	5,568
Total				107579	19320	

Contour

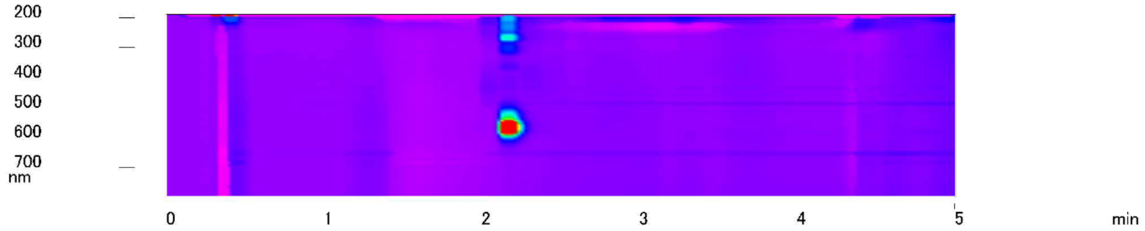

MS Chromatogram

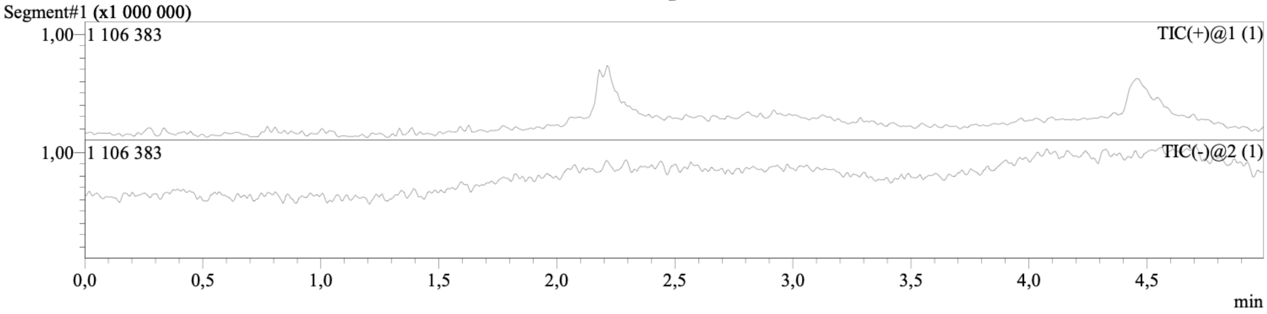

MS Spectrum

Line#:1 R.Time:----(Scan#:----)
MassPeaks:719
Spectrum Mode:Averaged 1,993-2,433(599-731) Base Peak:415(76304)
BG Mode:Averaged 0,507-1,380(153-415) Segment 1 - Event 1

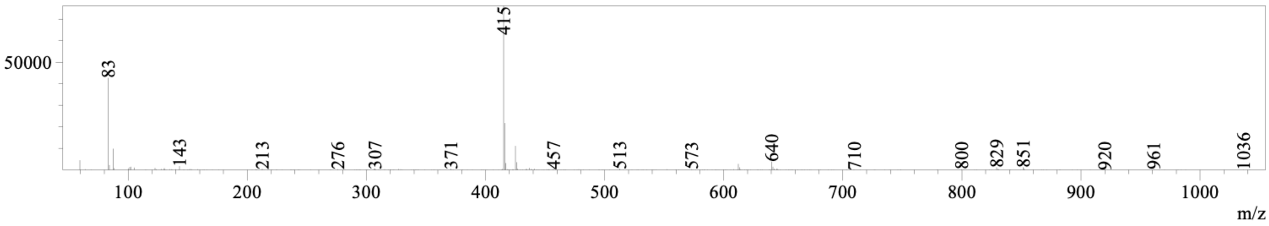

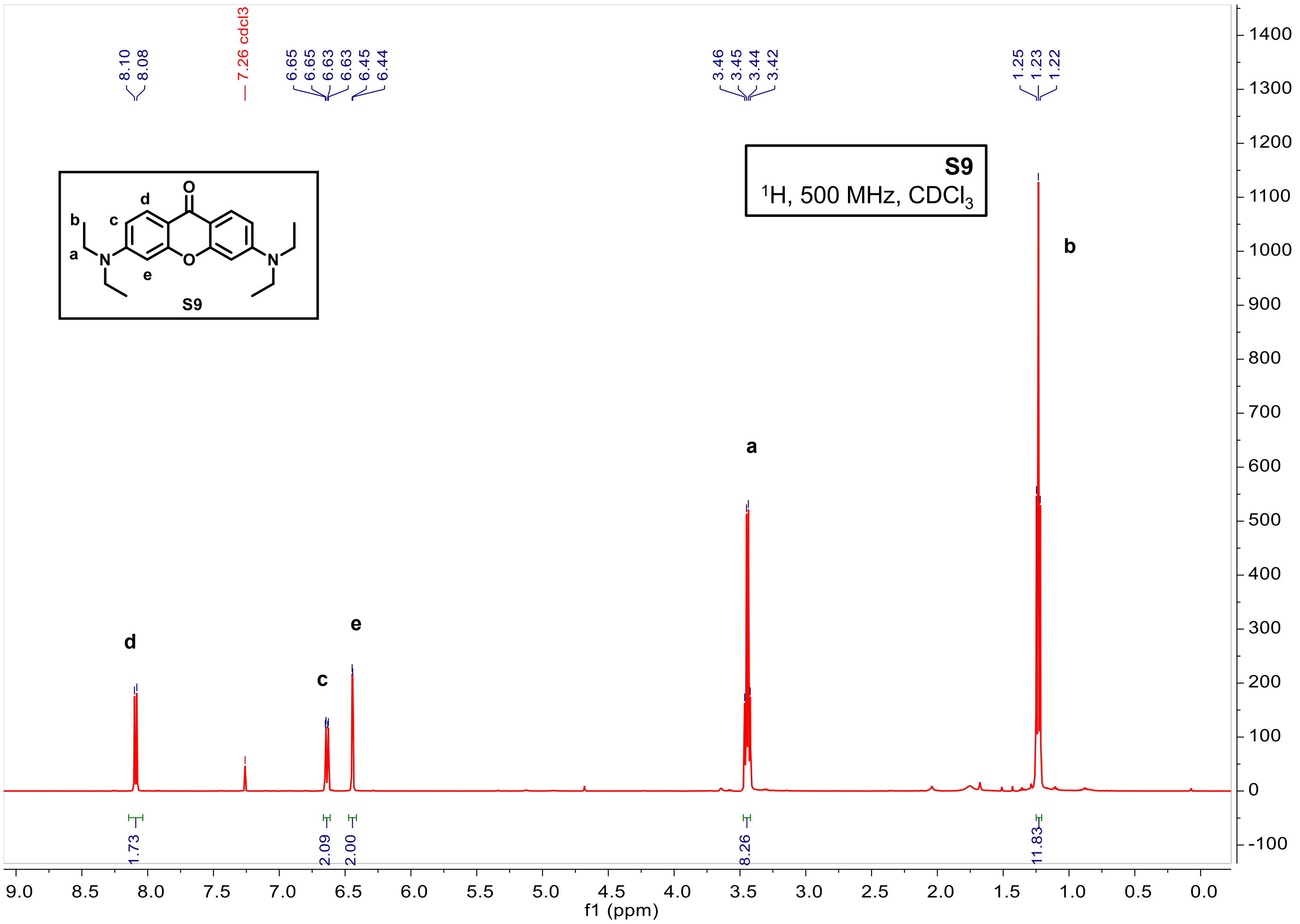
S9
1H, 500 MHz, CDCl3
8.10
8.08
7.26 cdcl3
6.65
6.65
6.63
6.63
6.45
6.44
3.46
3.45
3.44
3.42
1.25
1.23
1.22
d
c
e
a
b
1.73
2.09
2.00
8.26
11.83
f1 (ppm)

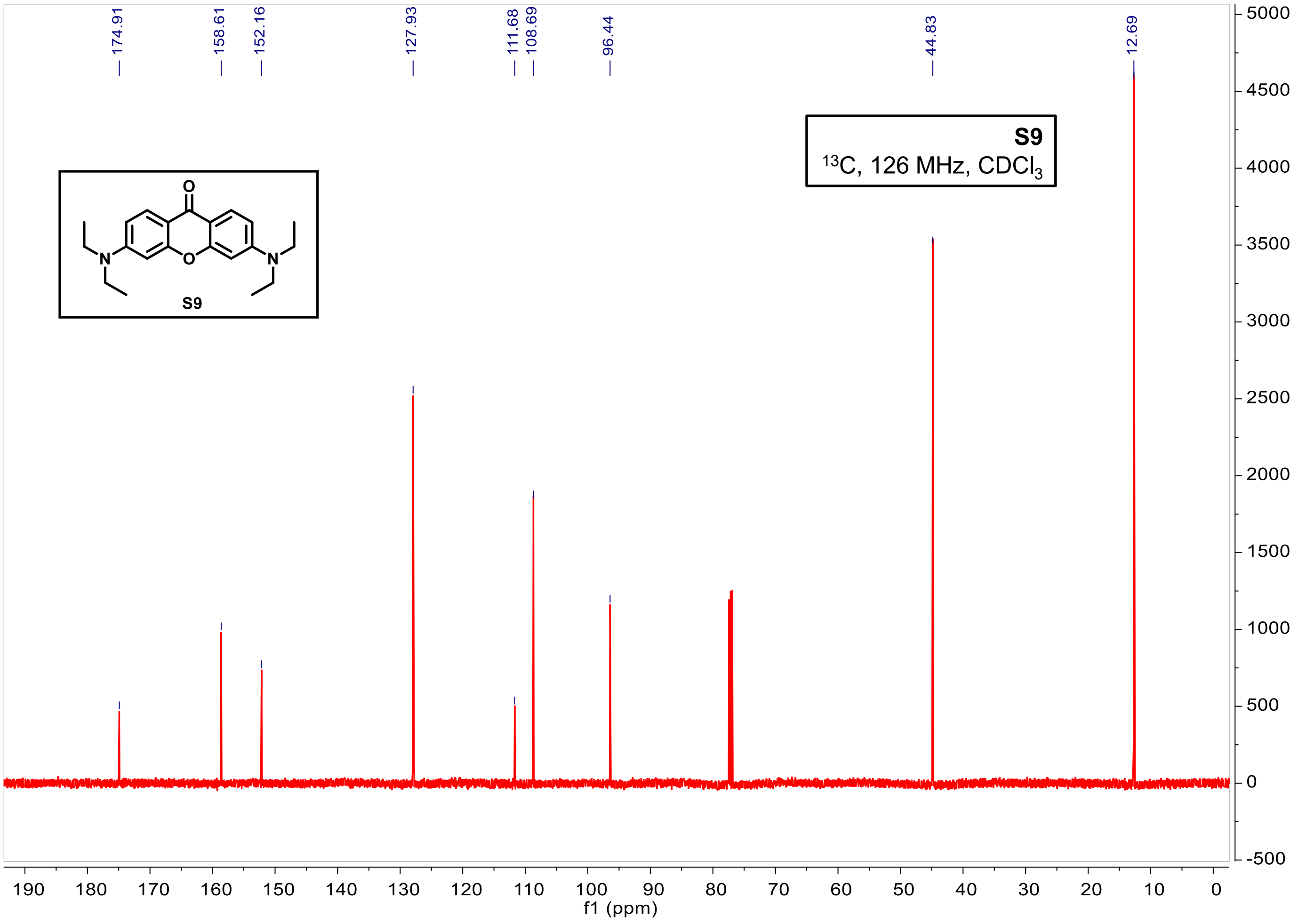
S9
13C, 126 MHz, CDCl3
174.91
158.61
152.16
127.93
111.68
108.69
96.44
44.83
12.69
f1 (ppm)

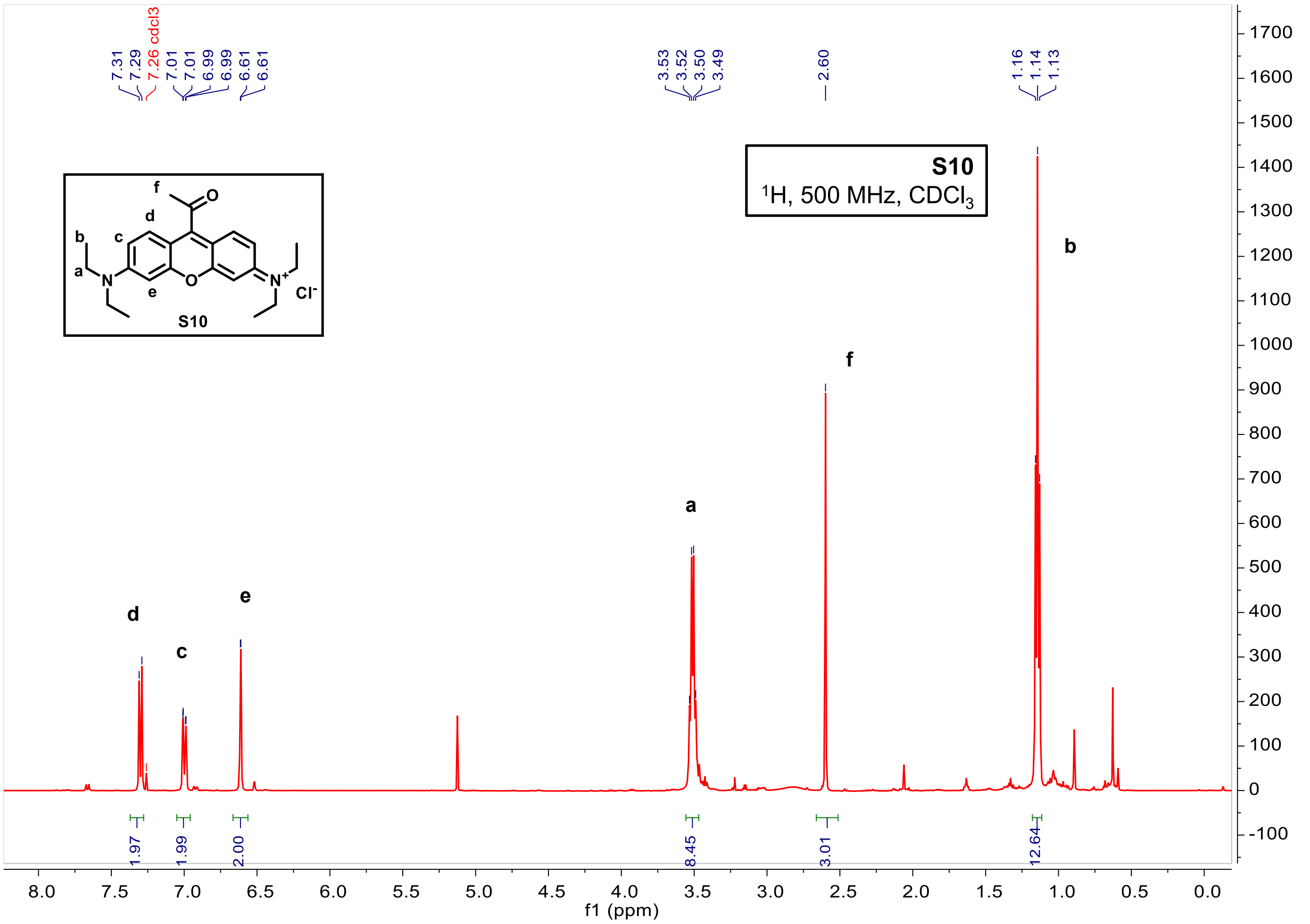
S10
1H, 500 MHz, CDCl3
f1 (ppm)

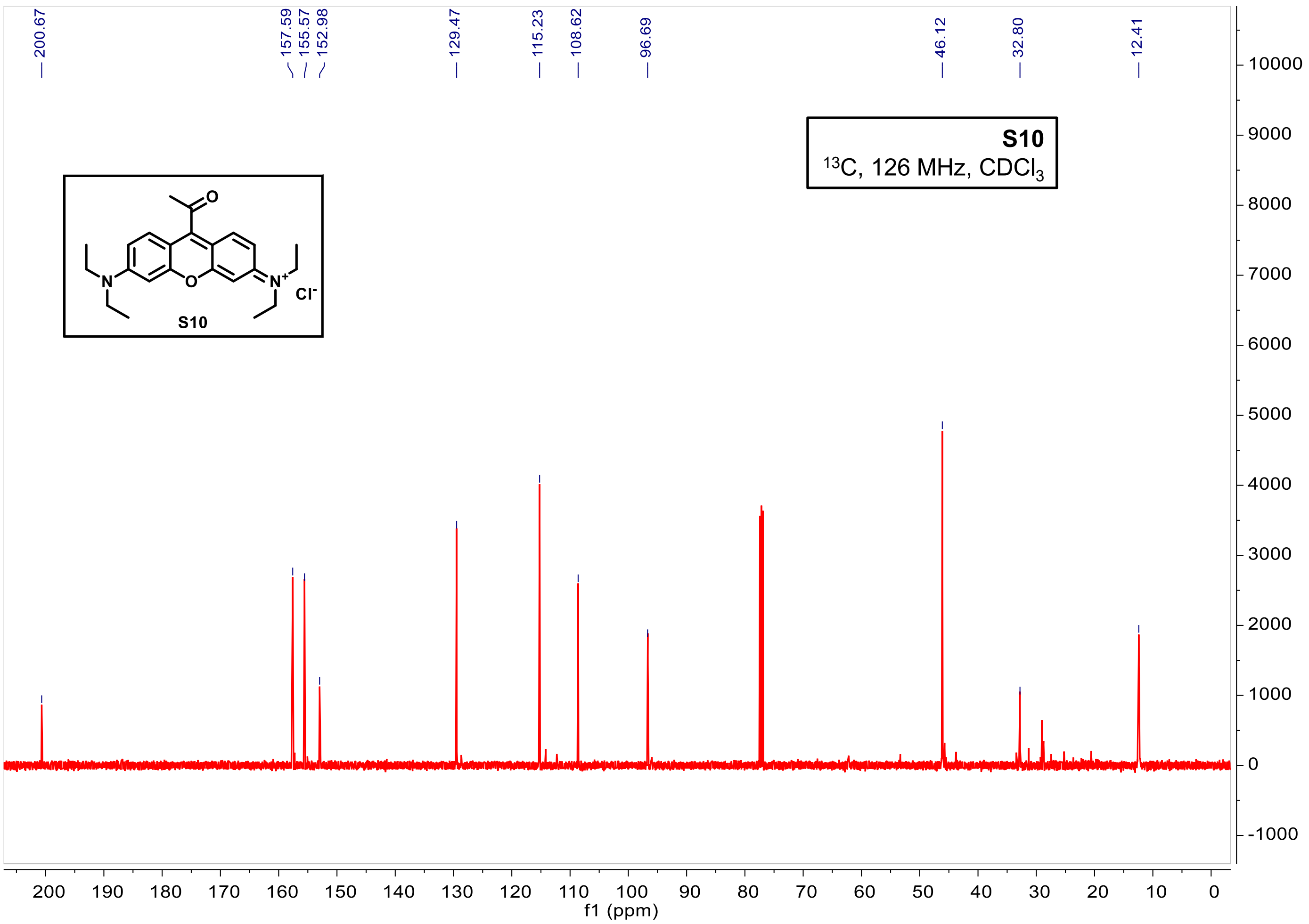
S10
13C, 126 MHz, CDCl3
f1 (ppm)

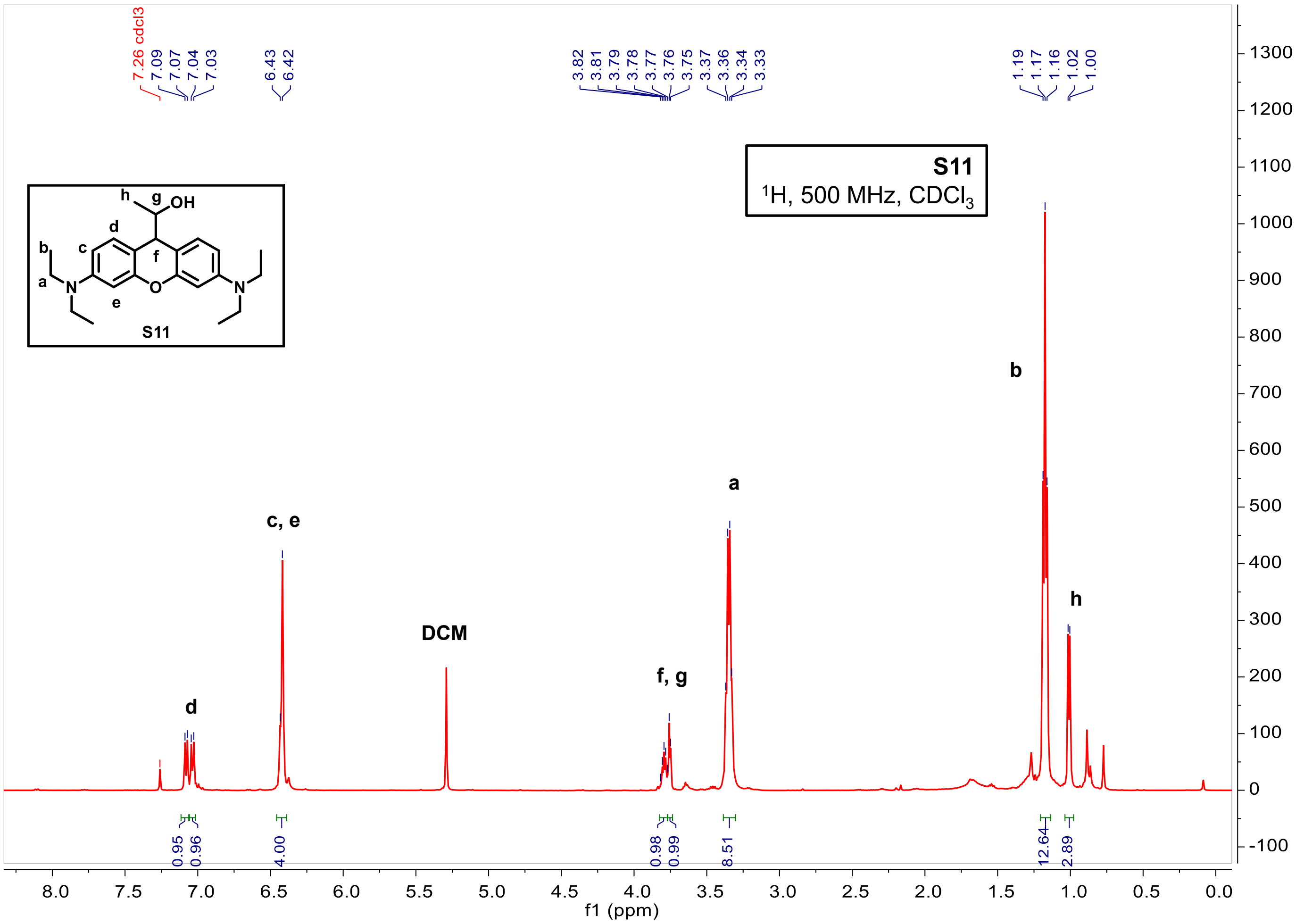
7.26 cdcl3
7.09
7.07
7.04
7.03
6.43
6.42
3.82
3.81
3.79
3.78
3.77
3.76
3.75
3.37
3.36
3.34
3.33
1.19
1.17
1.16
1.02
1.00
S11
^{1}H, 500 MHz, $CDCl_3$
h
g
OH
d
c
b
f
a
N
O
N
e
S11
b
a
c, e
DCM
f, g
d
h
0.95
0.96
4.00
0.98
0.99
8.51
12.64
2.89
f1 (ppm)

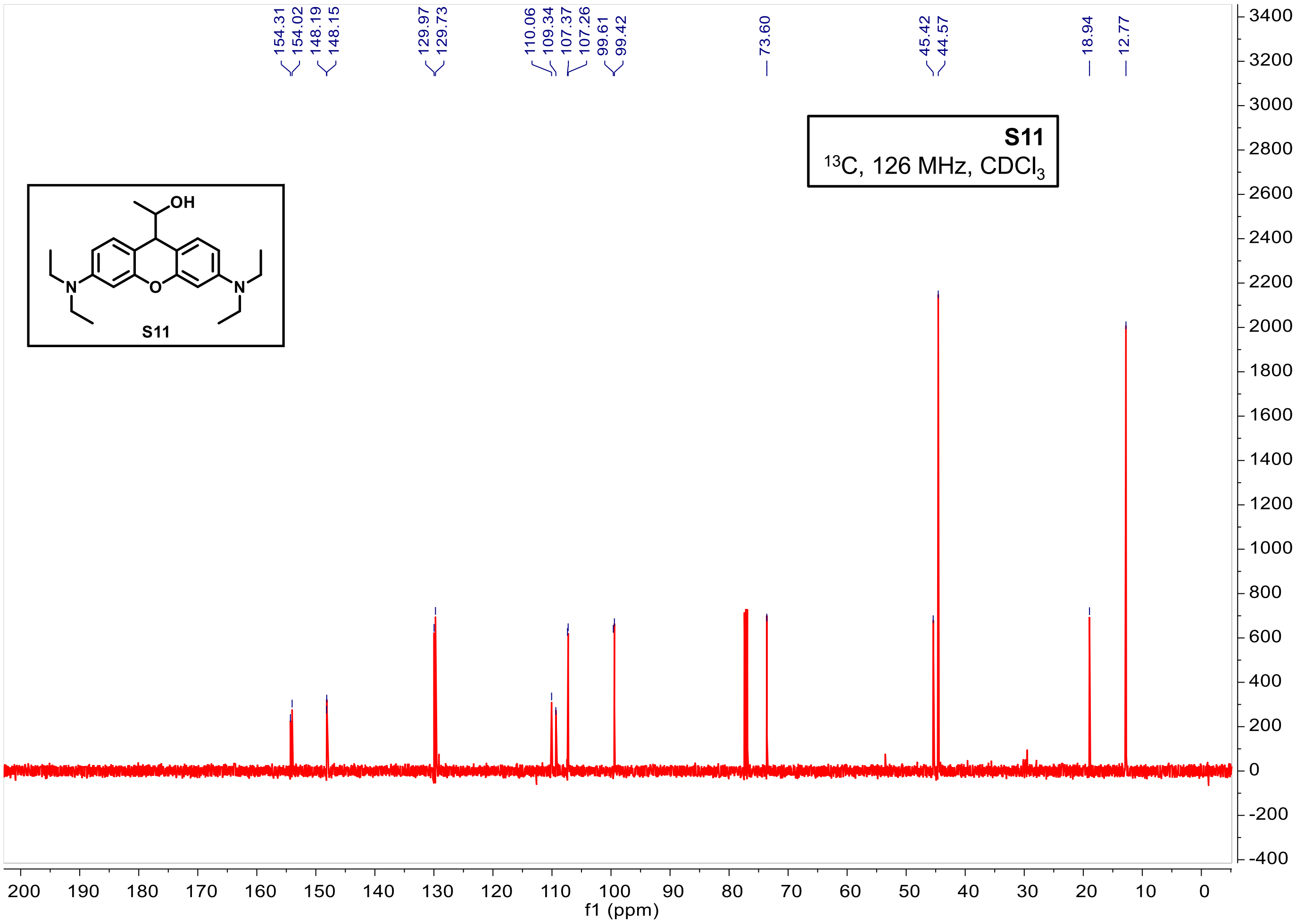
154.31
154.02
148.19
148.15
129.97
129.73
110.06
109.34
107.37
107.26
99.61
99.42
73.60
45.42
44.57
18.94
12.77
S11
^{13}C, 126 MHz, $CDCl_3$
OH
N
O
N
S11
f1 (ppm)

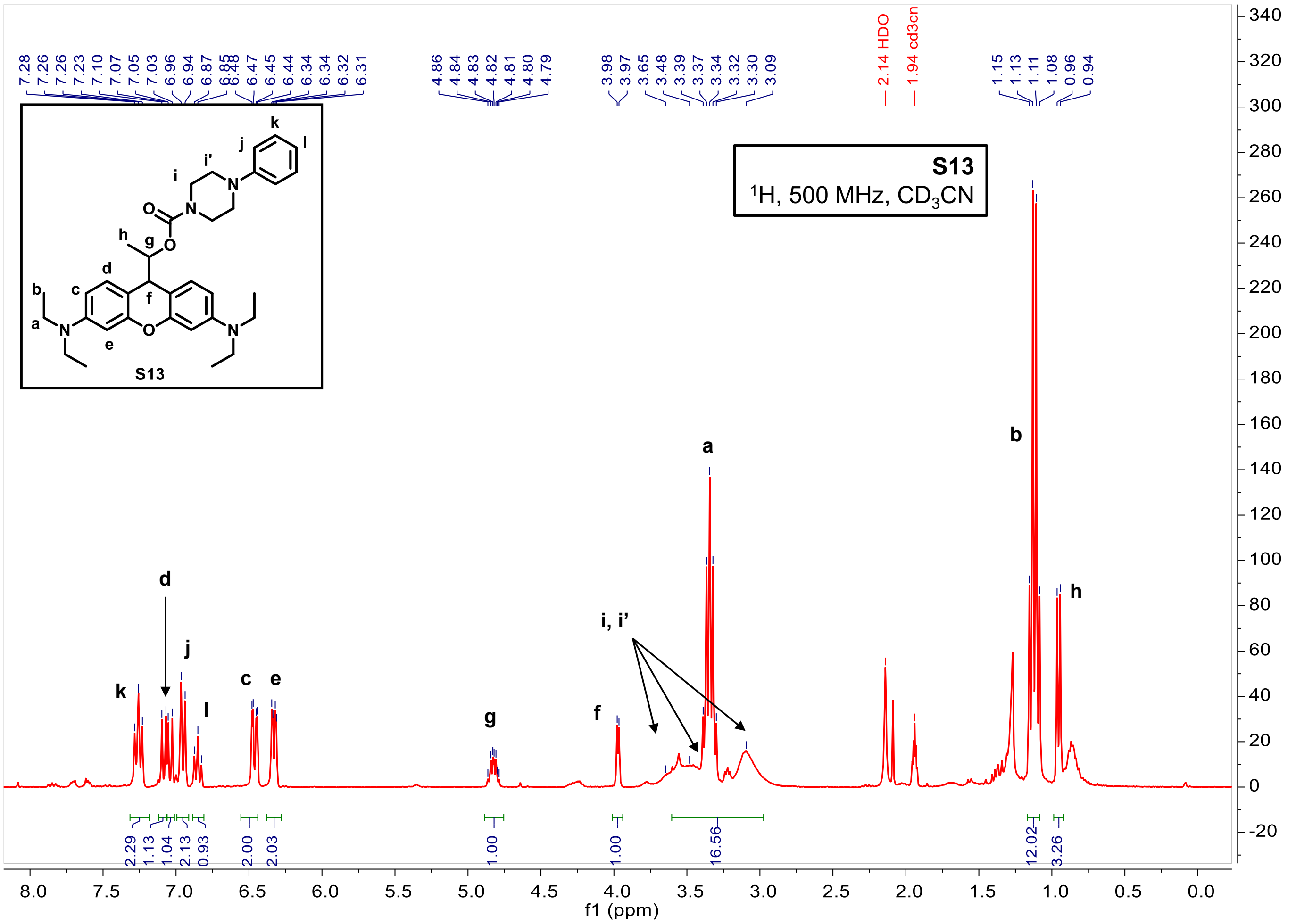
S13
^{1}H, 500 MHz, CD_3CN
2.14 HDO
1.94 cd3cn
a
b
h
i, i'
f
g
c
e
d
j
k
l
f1 (ppm)

Chromatogram

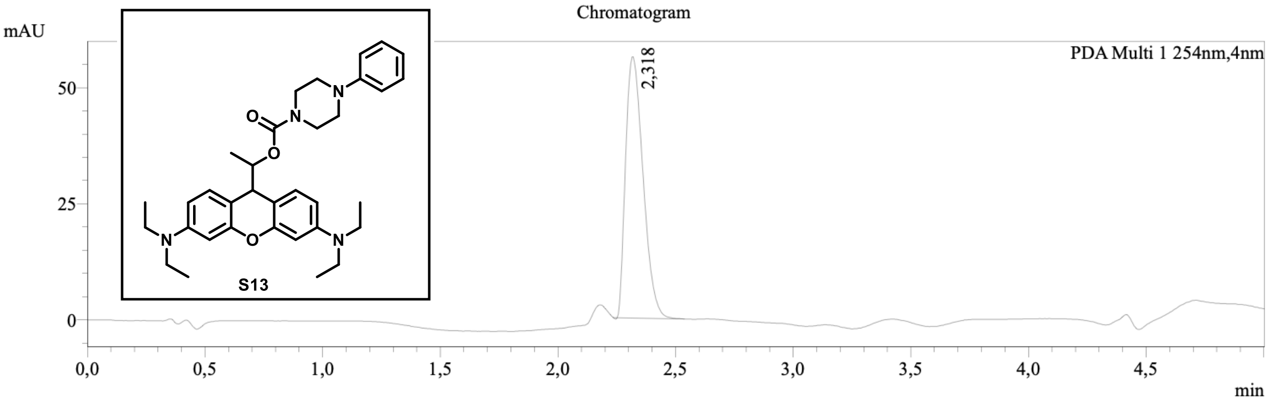

Peak Table

PDA Ch1 254nm

Peak#	Ret. Time	Peak Start	Peak End	Area	Height	Area/Height
1	2,318	2,235	2,539	290597	56234	5,168
Total				290597	56234	

Contour

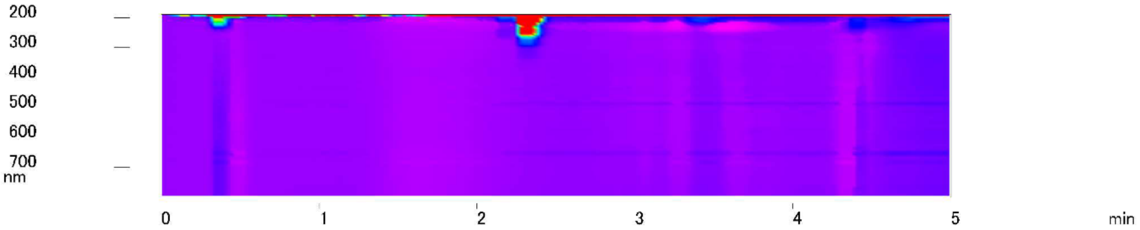

MS Chromatogram

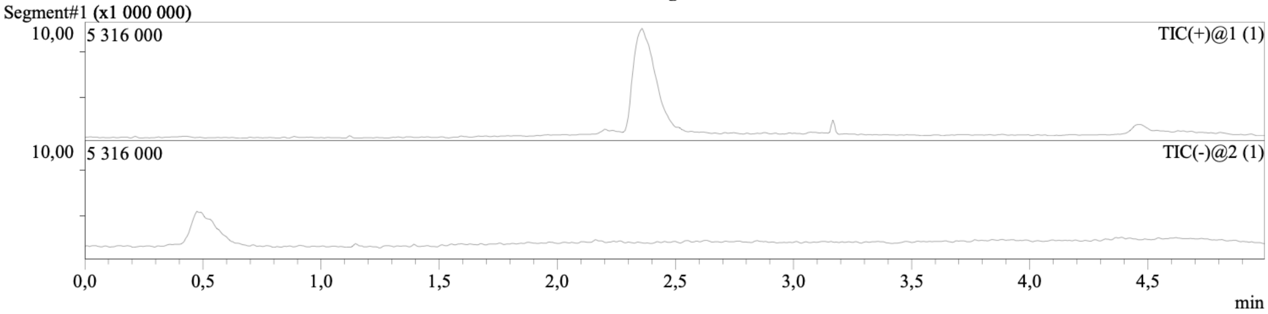

MS Spectrum

Line#:1 R.Time:----(Scan#:----)
MassPeaks:755
Spectrum Mode:Averaged 2,253-2,527(677-759) Base Peak:279(1105050)
BG Mode:Averaged 1,013-1,633(305-491) Segment 1 - Event 1

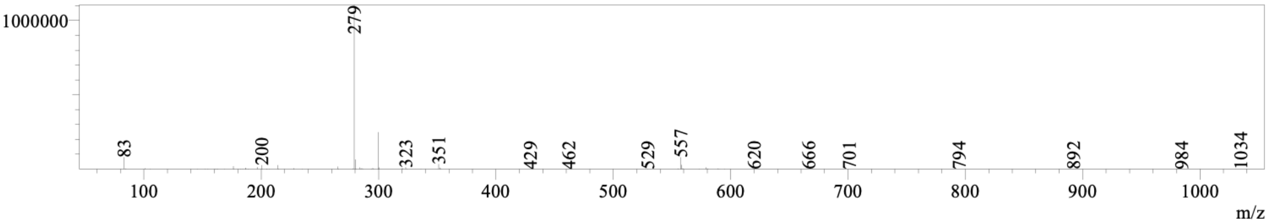

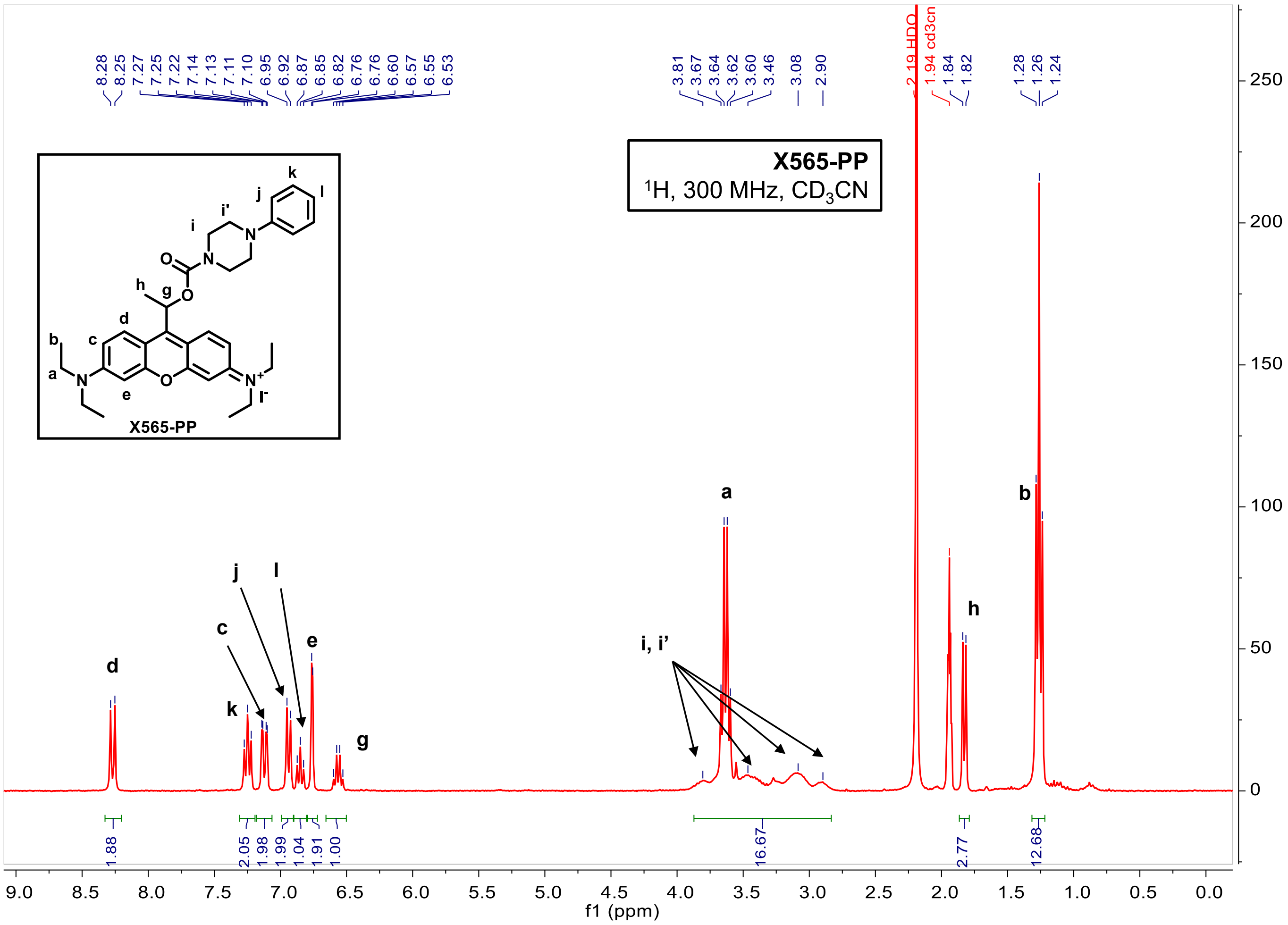
X565-PP
^{1}H, 300 MHz, CD_3CN
X565-PP
a
b
c
d
e
g
h
i, i'
j
k
l
2.19 HDO
1.94 cd3cn
f1 (ppm)

Chromatogram

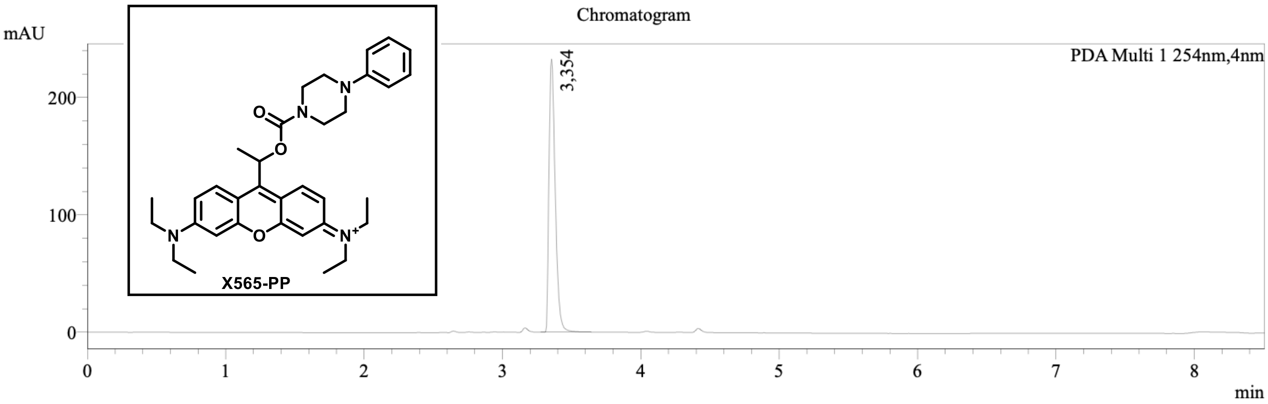

Peak Table

PDA Ch1 254nm

Peak#	Ret. Time	Peak Start	Peak End	Area	Height	Area/Height
1	3,354	3,275	3,643	730617	232735	3,139
Total				730617	232735	

Contour

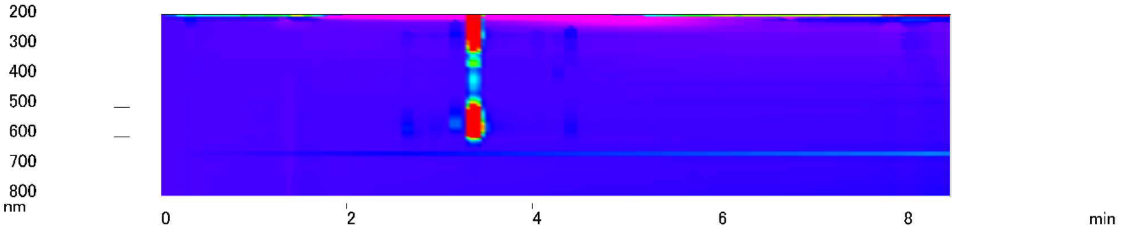

MS Chromatogram

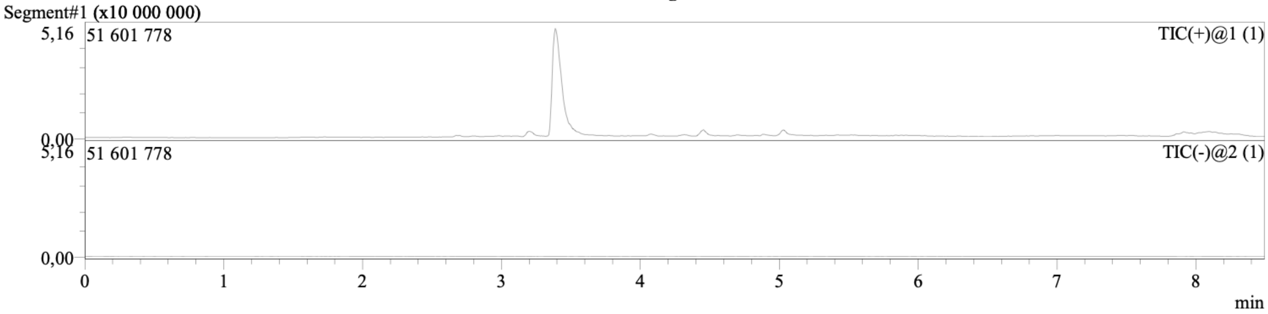

MS Spectrum

Line#:1 R.Time:----(Scan#:----)
MassPeaks:714
Spectrum Mode:Averaged 3,293-3,620(989-1087) Base Peak:555(3663503)
BG Mode:Averaged 0,627-1,307(189-393) Segment 1 - Event 1

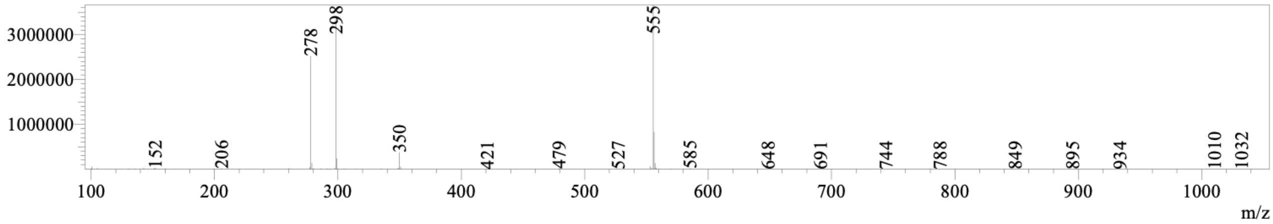

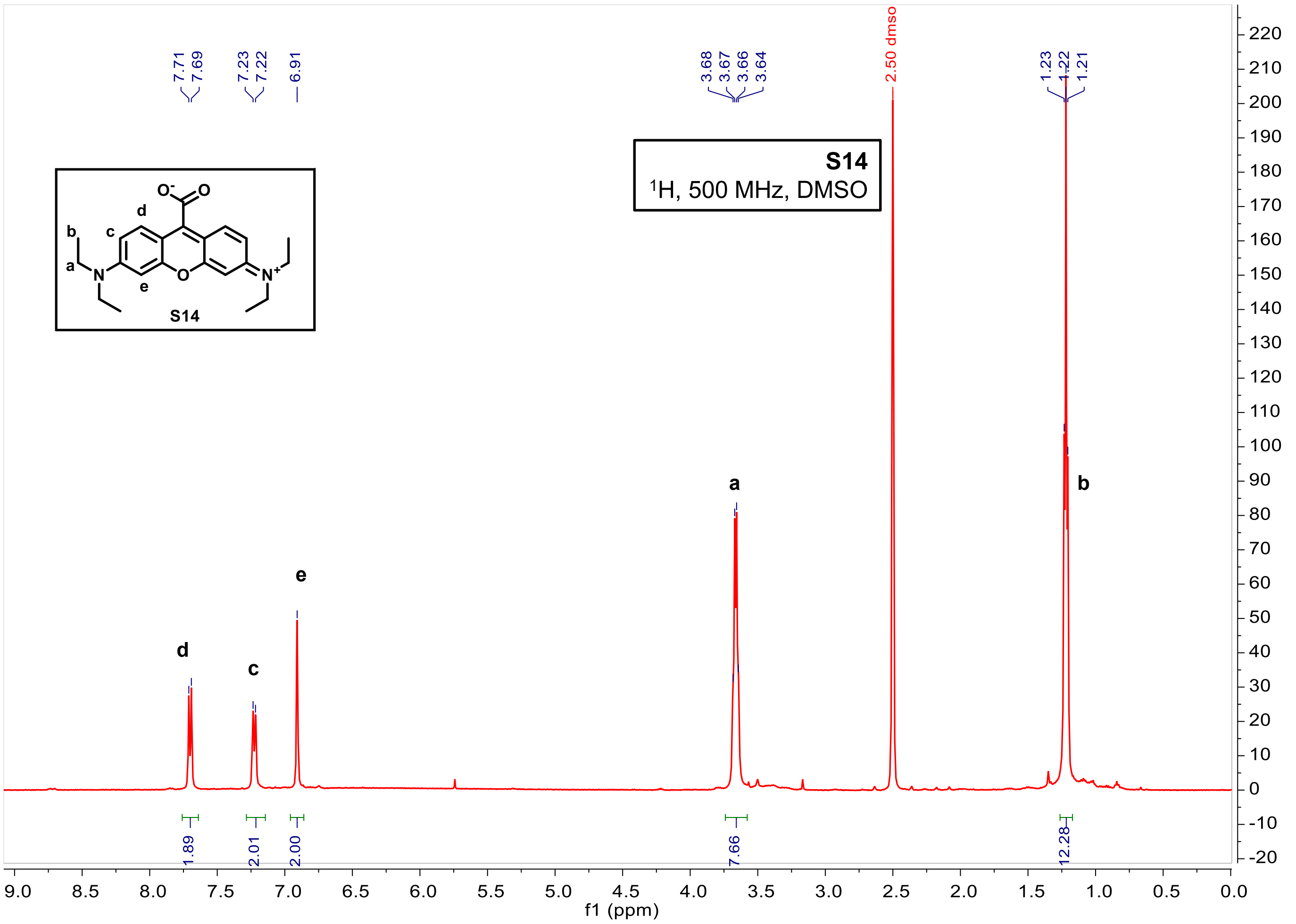
7.71
7.69
7.23
7.22
6.91
3.68
3.67
3.66
3.64
2.50 dmso
1.23
1.22
1.21
S14
1H, 500 MHz, DMSO
a
b
c
d
e
1.89
2.01
2.00
7.66
12.28
f1 (ppm)

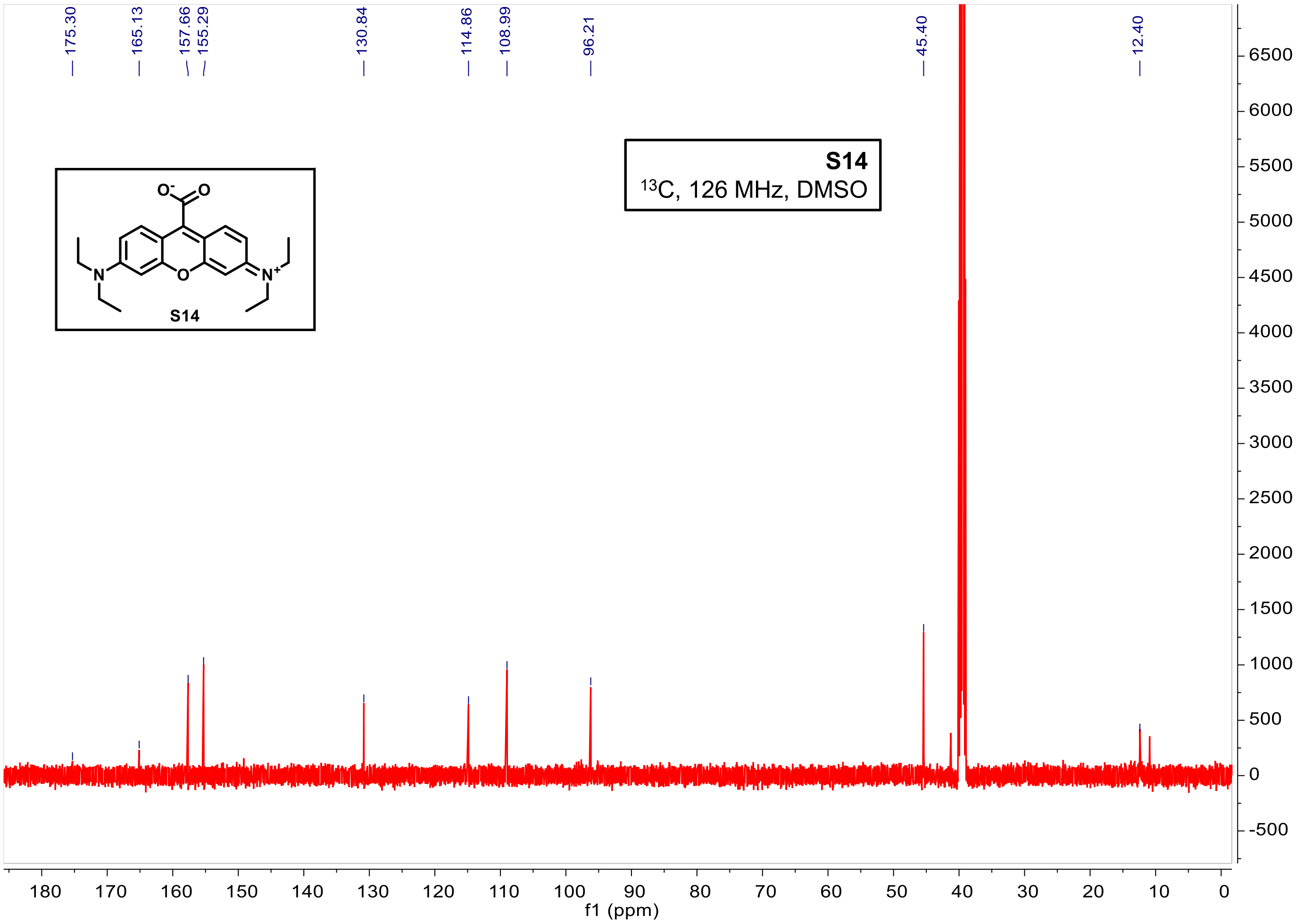
175.30
165.13
157.66
155.29
130.84
114.86
108.99
96.21
45.40
12.40
S14
13C, 126 MHz, DMSO
f1 (ppm)

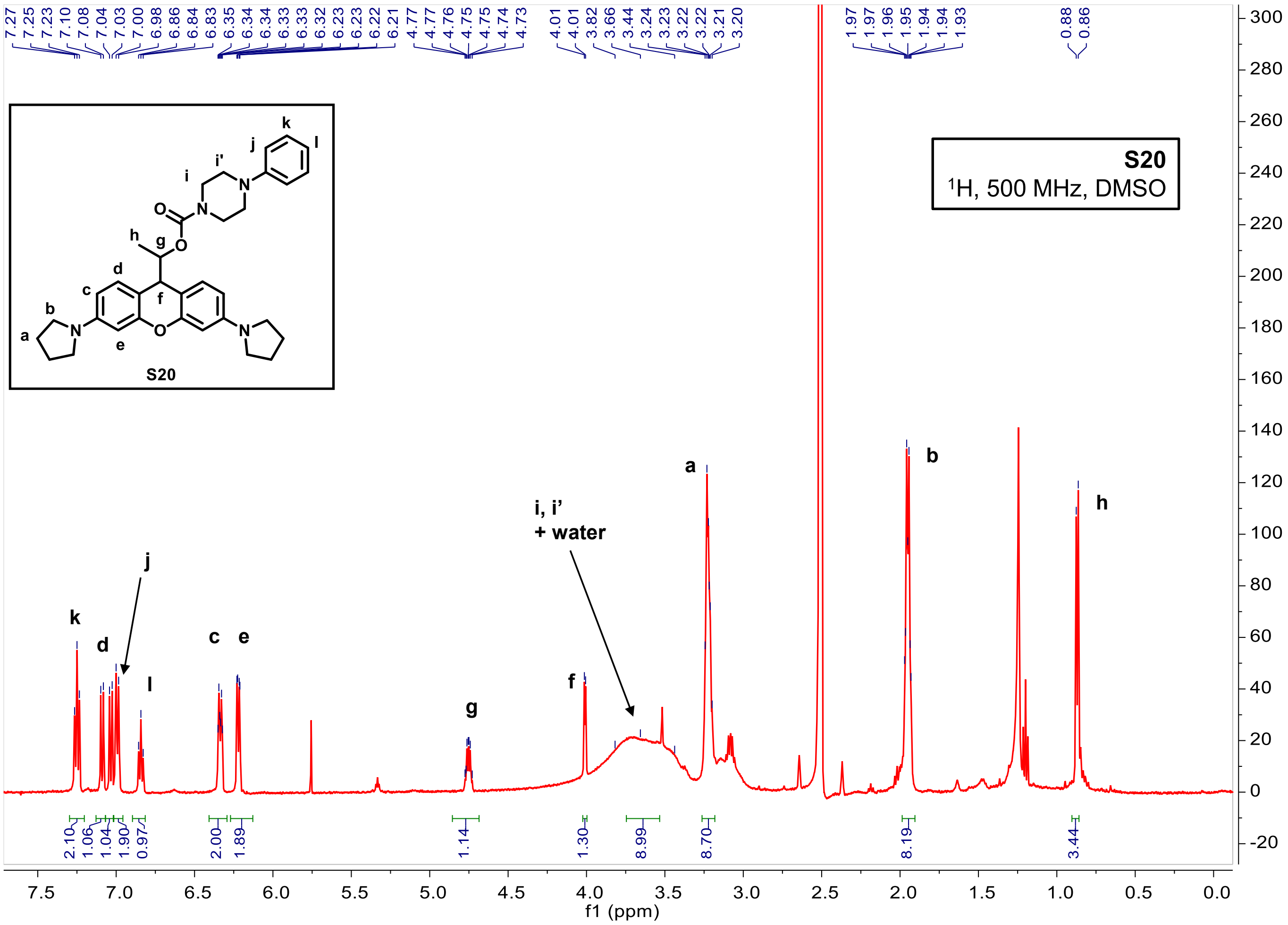
S20
¹H, 500 MHz, DMSO
S20
k
d
j
l
c
e
g
f
i, i'
+ water
a
b
h
f1 (ppm)

Chromatogram

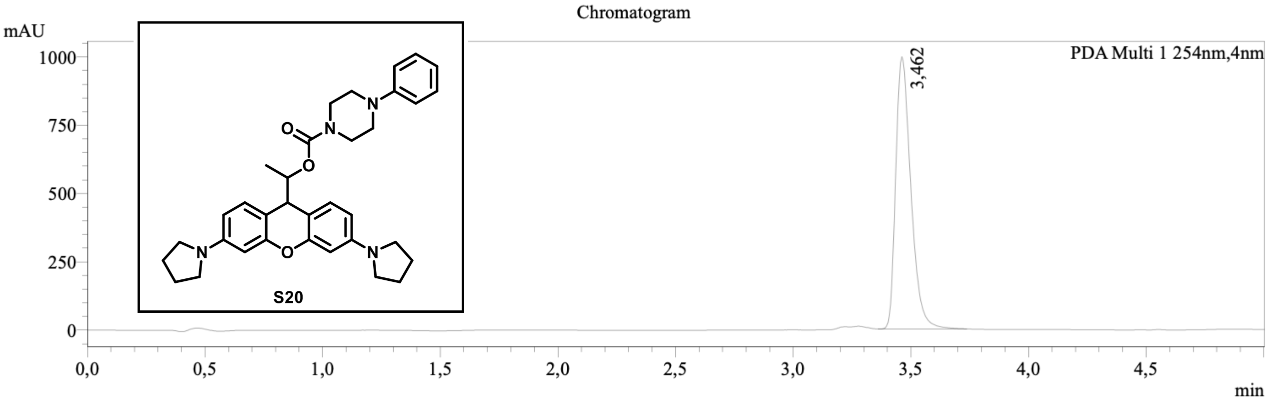

Peak Table

PDA Ch1 254nm

Peak#	Ret. Time	Peak Start	Peak End	Area	Height	Area/Height
1	3,462	3,360	3,739	4469105	996291	4,486
Total				4469105	996291	

Contour

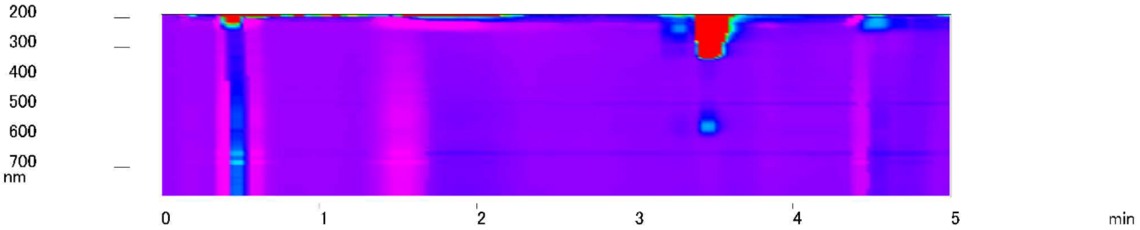

MS Chromatogram

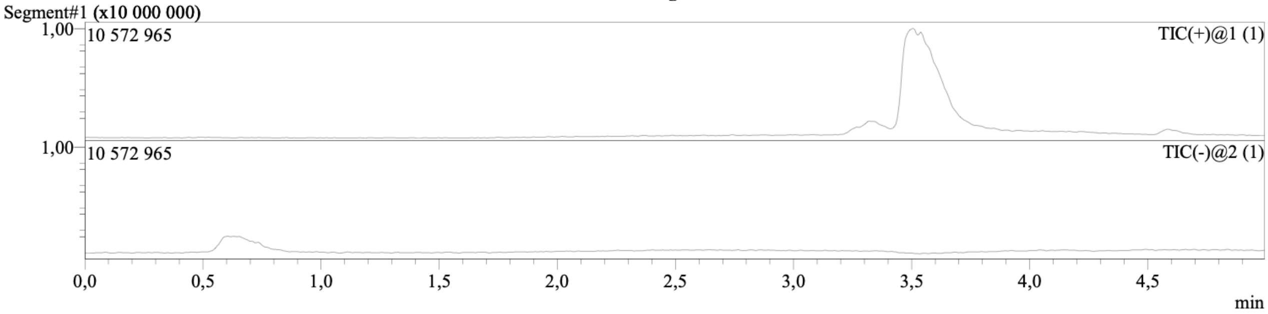

MS Spectrum

Line#:1 R.Time:----(Scan#:----)
MassPeaks:932
Spectrum Mode:Averaged 3,407-3,913(1023-1175) Base Peak:277(861870)
BG Mode:Averaged 1,140-2,000(343-601) Segment 1 - Event 1

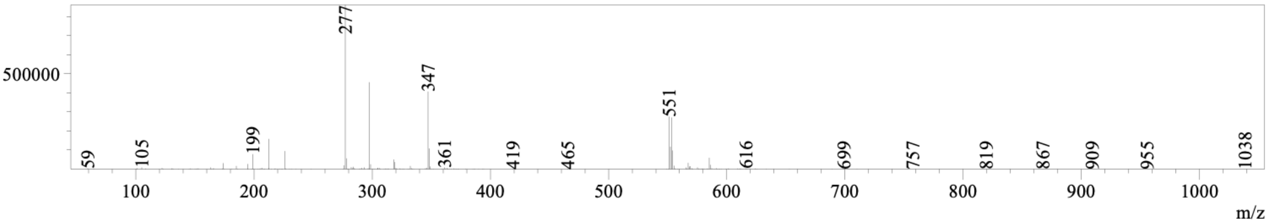

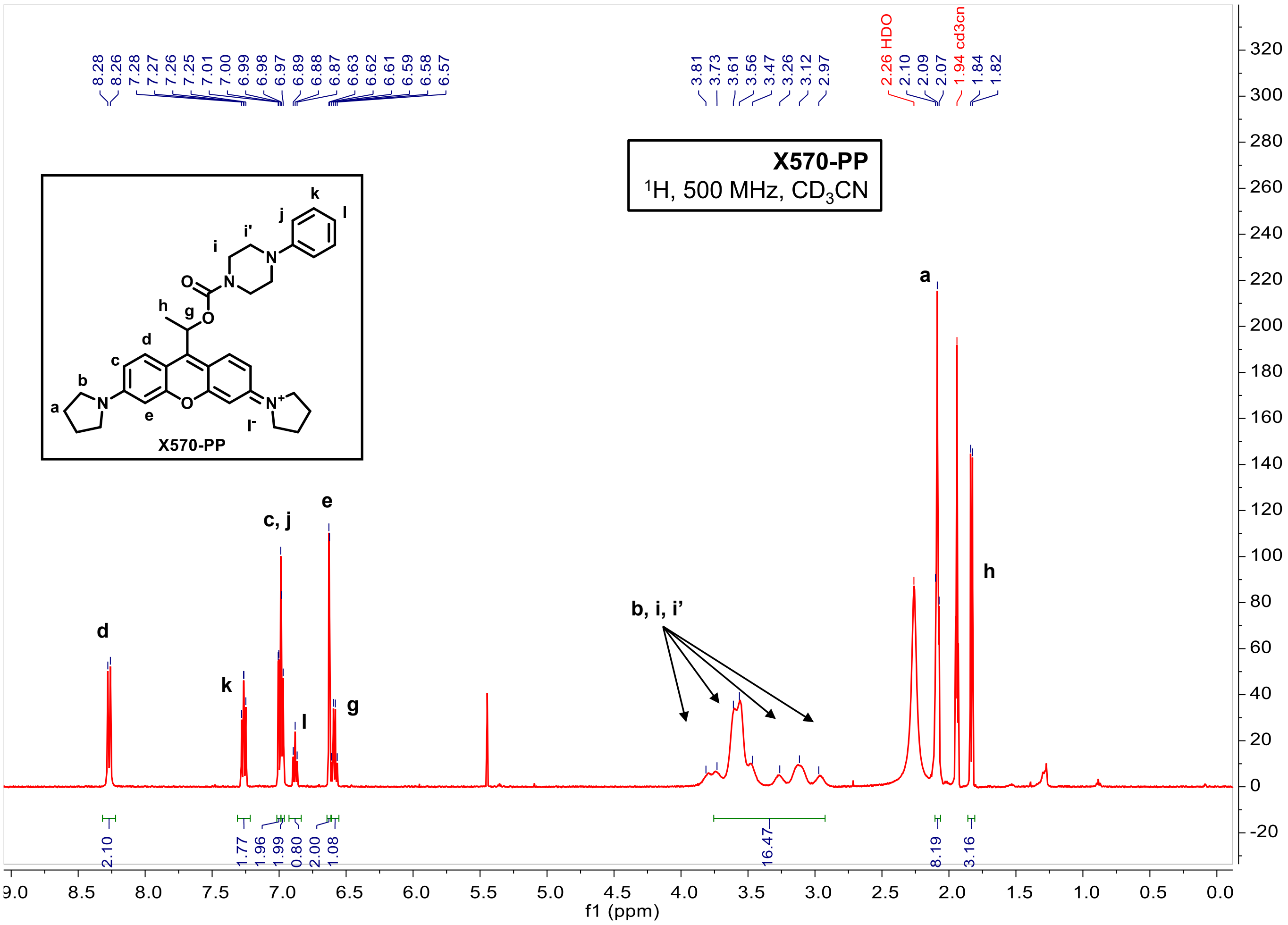
X570-PP
^{1}H, 500 MHz, CD_3CN
X570-PP
d
k
c, j
l
e
g
b, i, i'
a
h
2.26 HDO
1.94 cd3cn
f1 (ppm)

Chromatogram

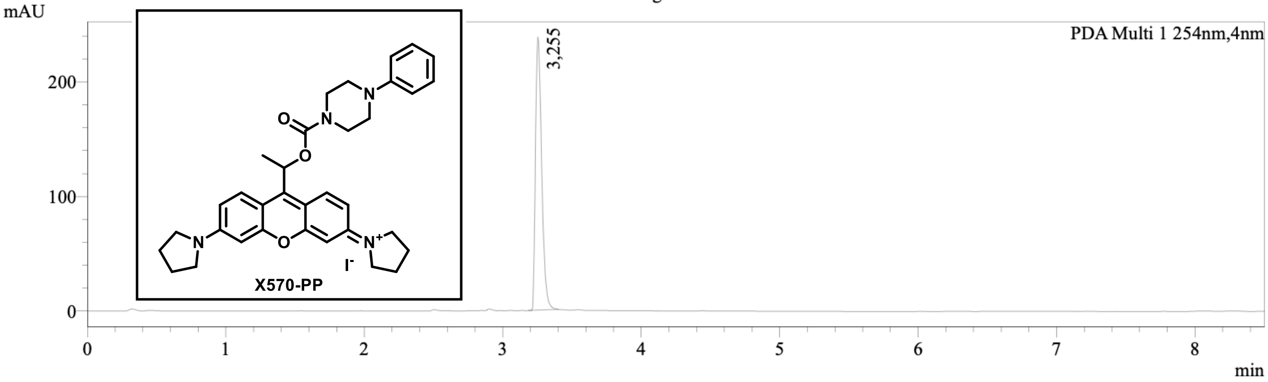

Peak Table

PDA Ch1 254nm

Peak#	Ret. Time	Peak Start	Peak End	Area	Height	Area/Height
1	3,255	3,184	3,403	745586	238264	3,129
Total				745586	238264	

Contour

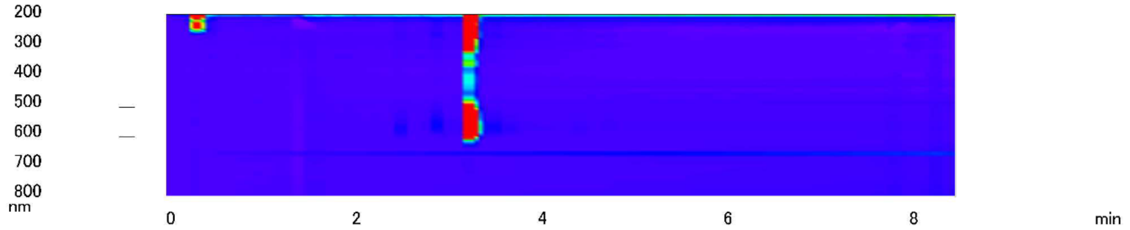

MS Chromatogram

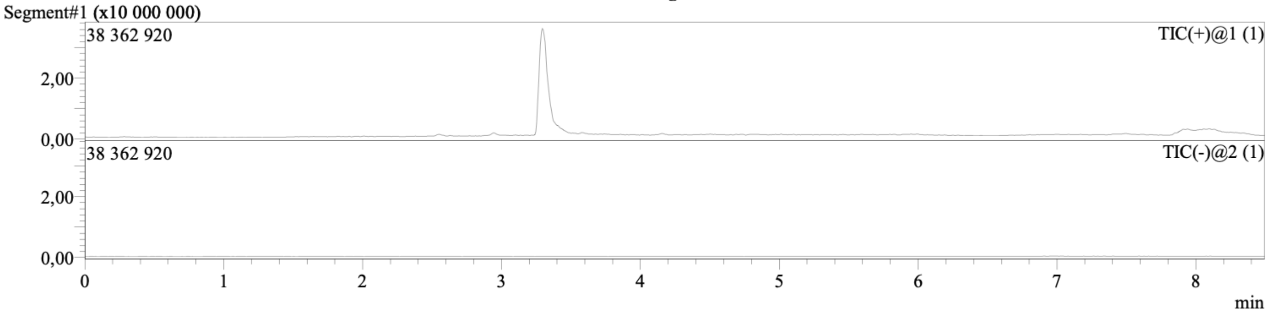

MS Spectrum

Line#:1 R.Time:----(Scan#:----)
MassPeaks:737
Spectrum Mode:Averaged 3,160-3,553(949-1067) Base Peak:551(2387930)
BG Mode:Averaged 0,993-1,553(299-467) Segment 1 - Event 1

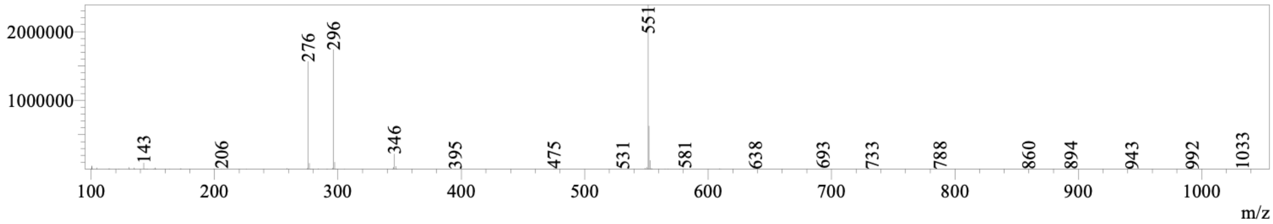

Chromatogram

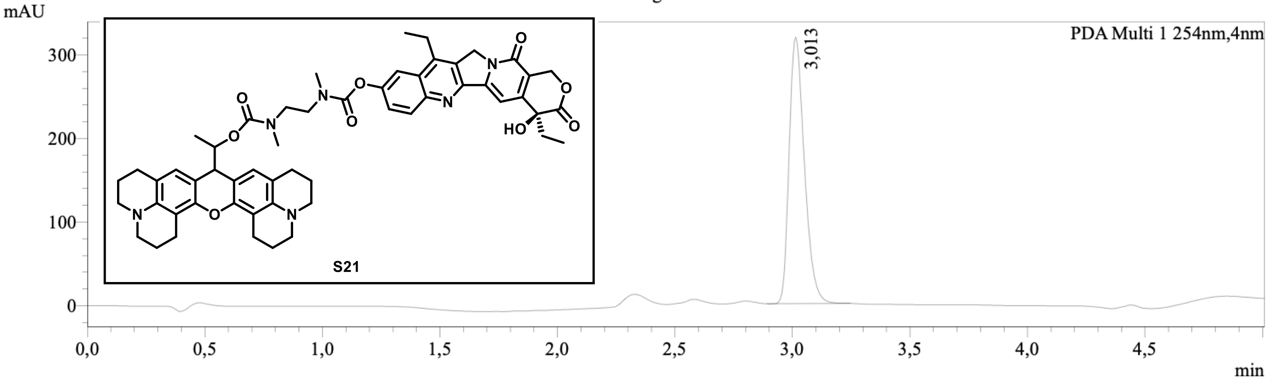

Peak Table

PDA Ch1 254nm

Peak#	Ret. Time	Peak Start	Peak End	Area	Height	Area/Height
1	3,013	2,891	3,248	1438115	318864	4,510
Total				1438115	318864	

Contour

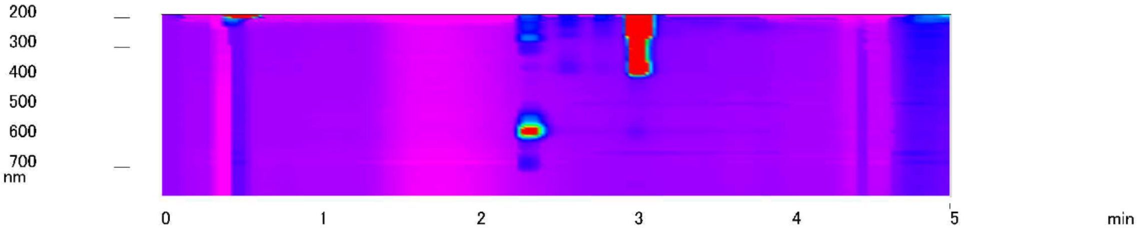

MS Chromatogram

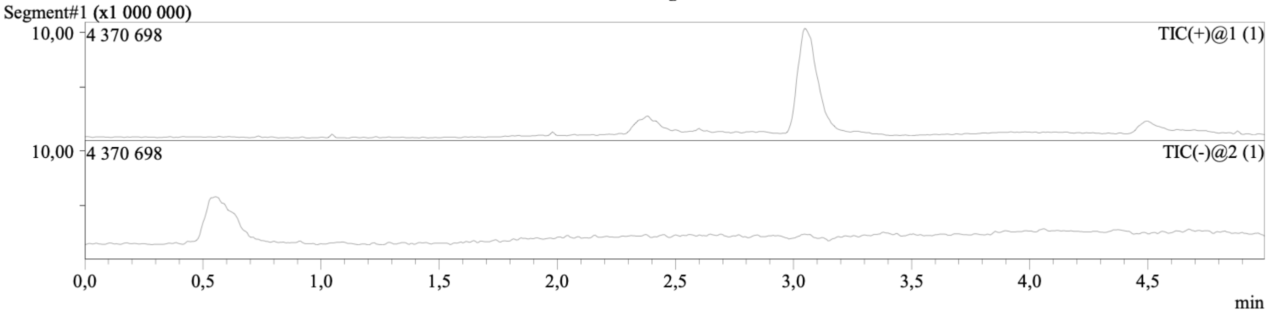

MS Spectrum

Line#:1 R.Time:----(Scan#:----)
MassPeaks:856
Spectrum Mode:Averaged 2,933-3,280(881-985) Base Peak:475(293787)
BG Mode:Averaged 1,173-1,727(353-519) Segment 1 - Event 1

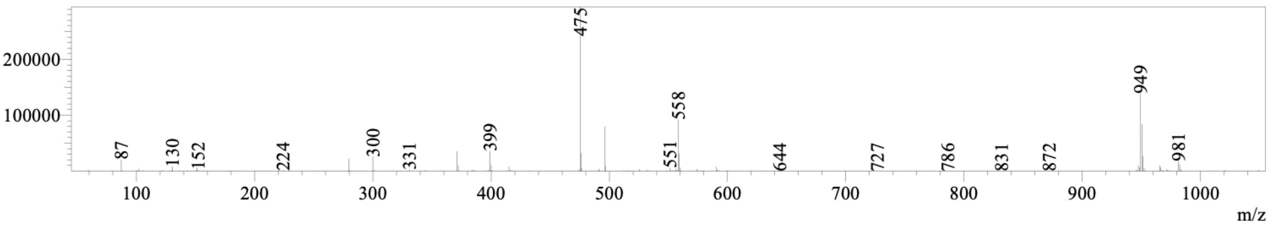

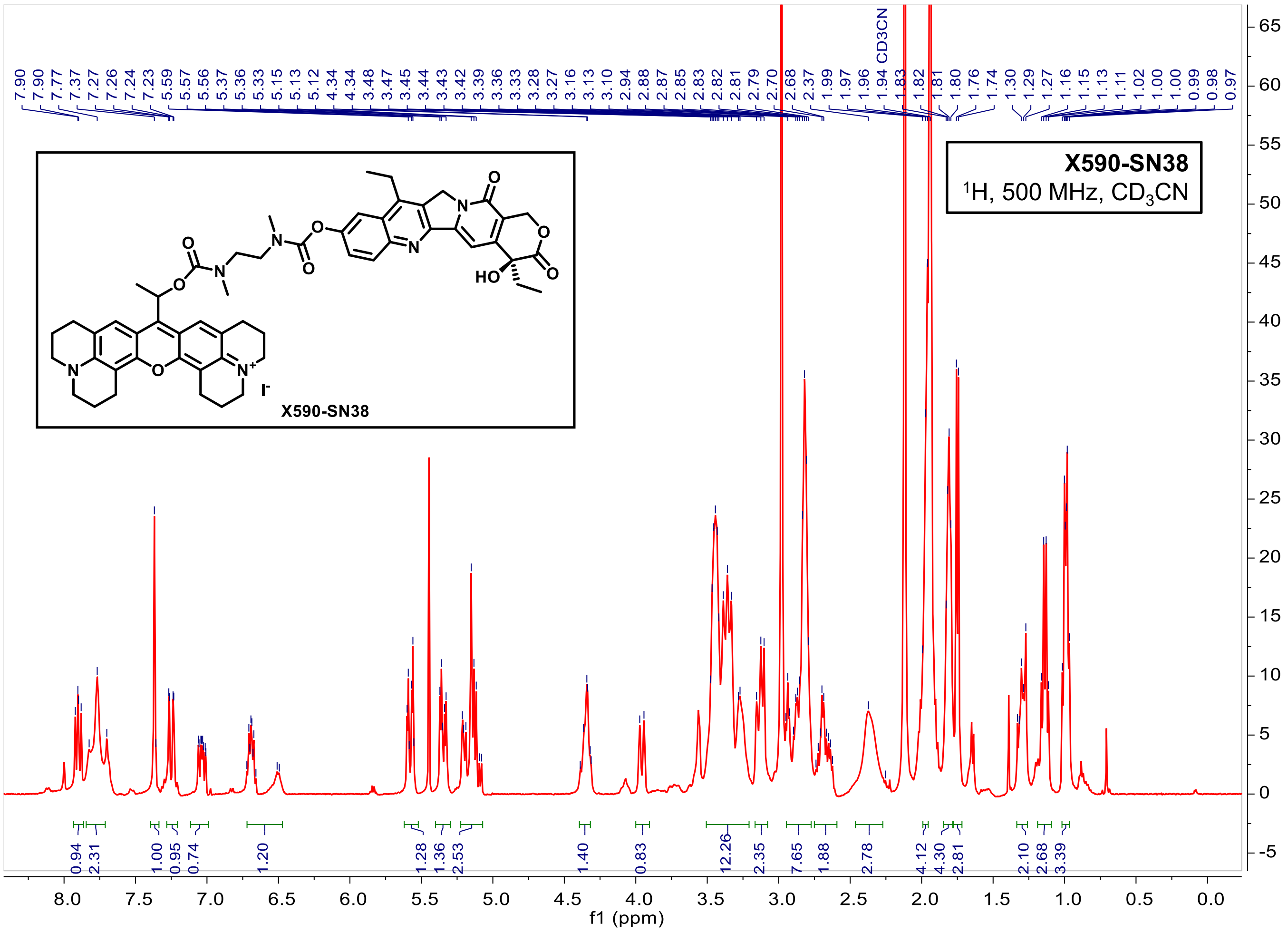
X590-SN38
^{1}H, 500 MHz, CD_3CN
CD3CN
f1 (ppm)

Chromatogram

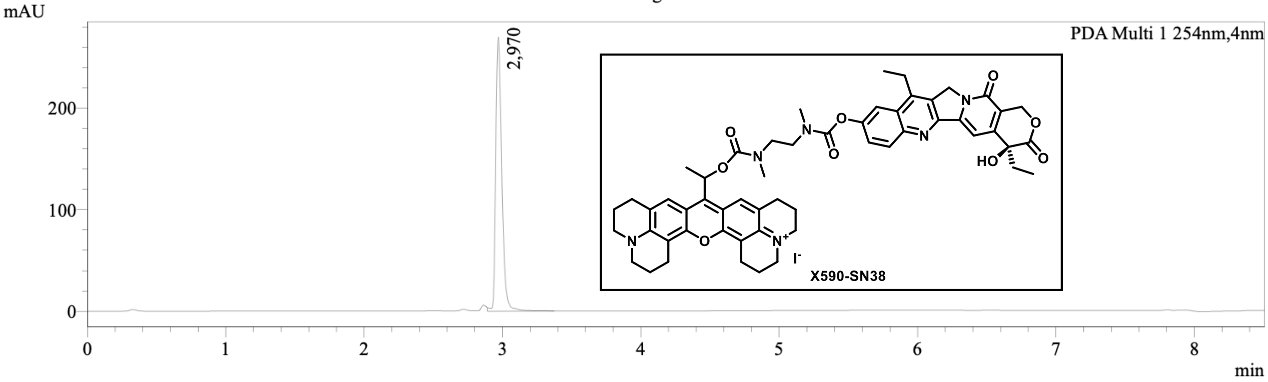

Peak Table

PDA Ch1 254nm

Peak#	Ret. Time	Peak Start	Peak End	Area	Height	Area/Height
1	2,970	2,891	3,376	827789	269954	3,066
Total				827789	269954	

Contour

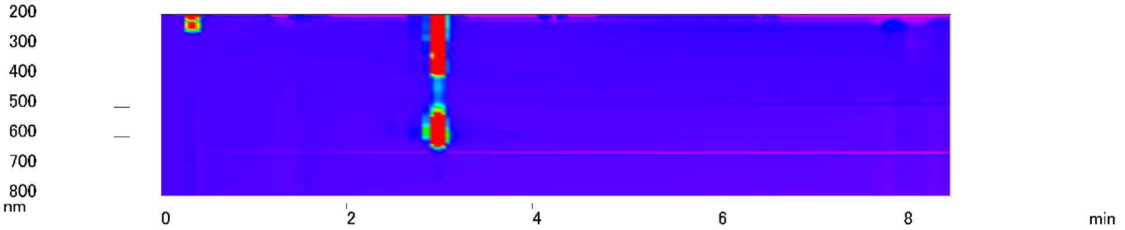

MS Chromatogram

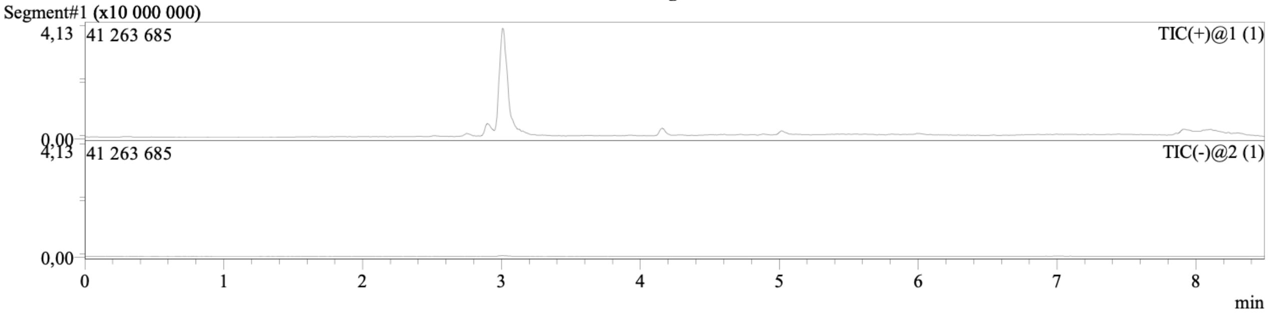

MS Spectrum

Line#:1 R.Time:----(Scan#:----)
MassPeaks:761
Spectrum Mode:Averaged 2,940-3,327(883-999) Base Peak:948(2783668)
BG Mode:Averaged 1,040-2,033(313-611) Segment 1 - Event 1

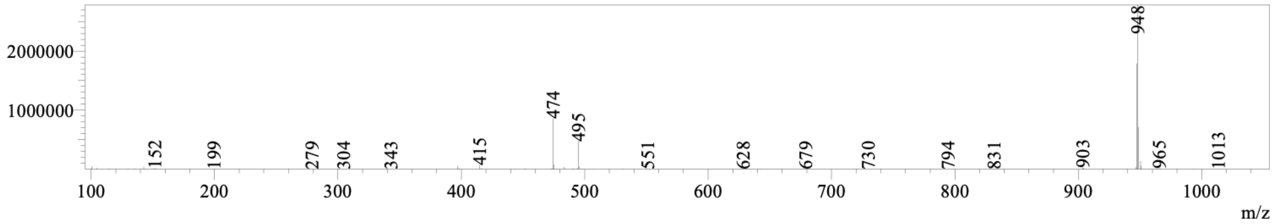

Chromatogram

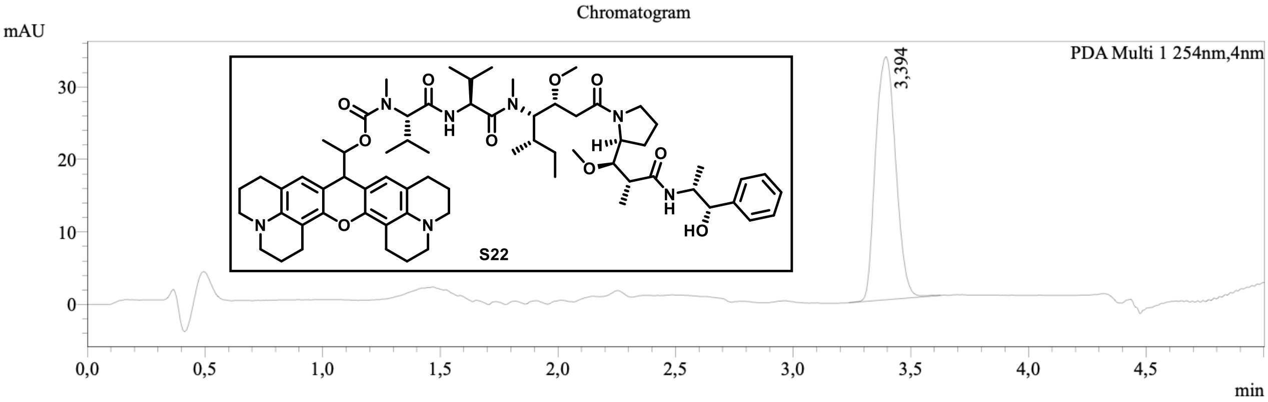

Peak Table

PDA Ch1 254nm

Peak#	Ret. Time	Peak Start	Peak End	Area	Height	Area/Height
1	3,394	3,237	3,627	200762	33519	5,990
Total				200762	33519	

Contour

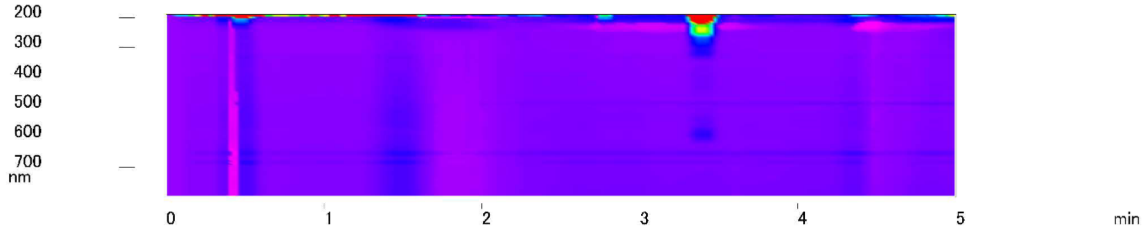

MS Chromatogram

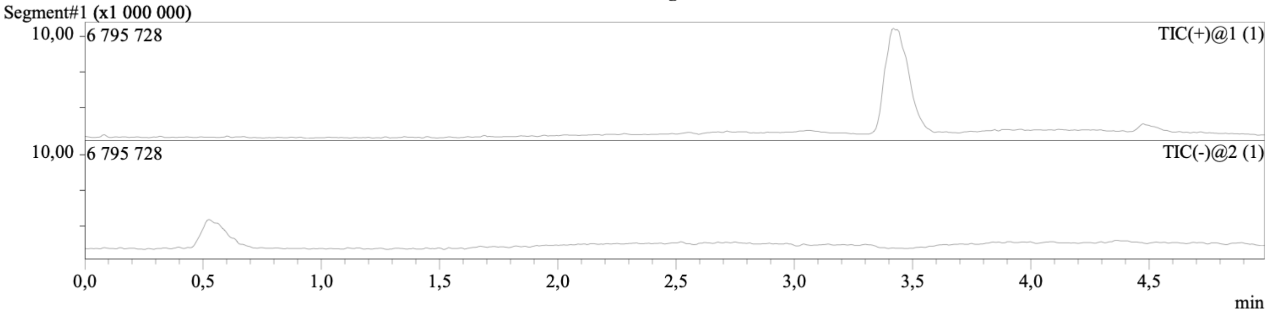

MS Spectrum

Line#:1 R.Time:----(Scan#:----)
MassPeaks:1566
Spectrum Mode:Averaged 3,263-3,668(871-979) Base Peak:581(726817)
BG Mode:Averaged 1,253-2,123(335-567) Segment 1 - Event 1

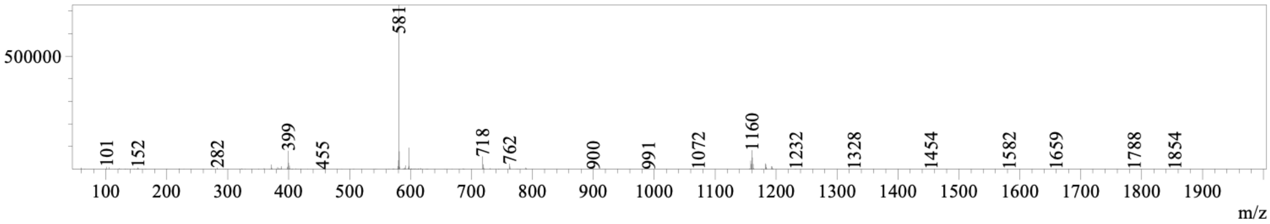

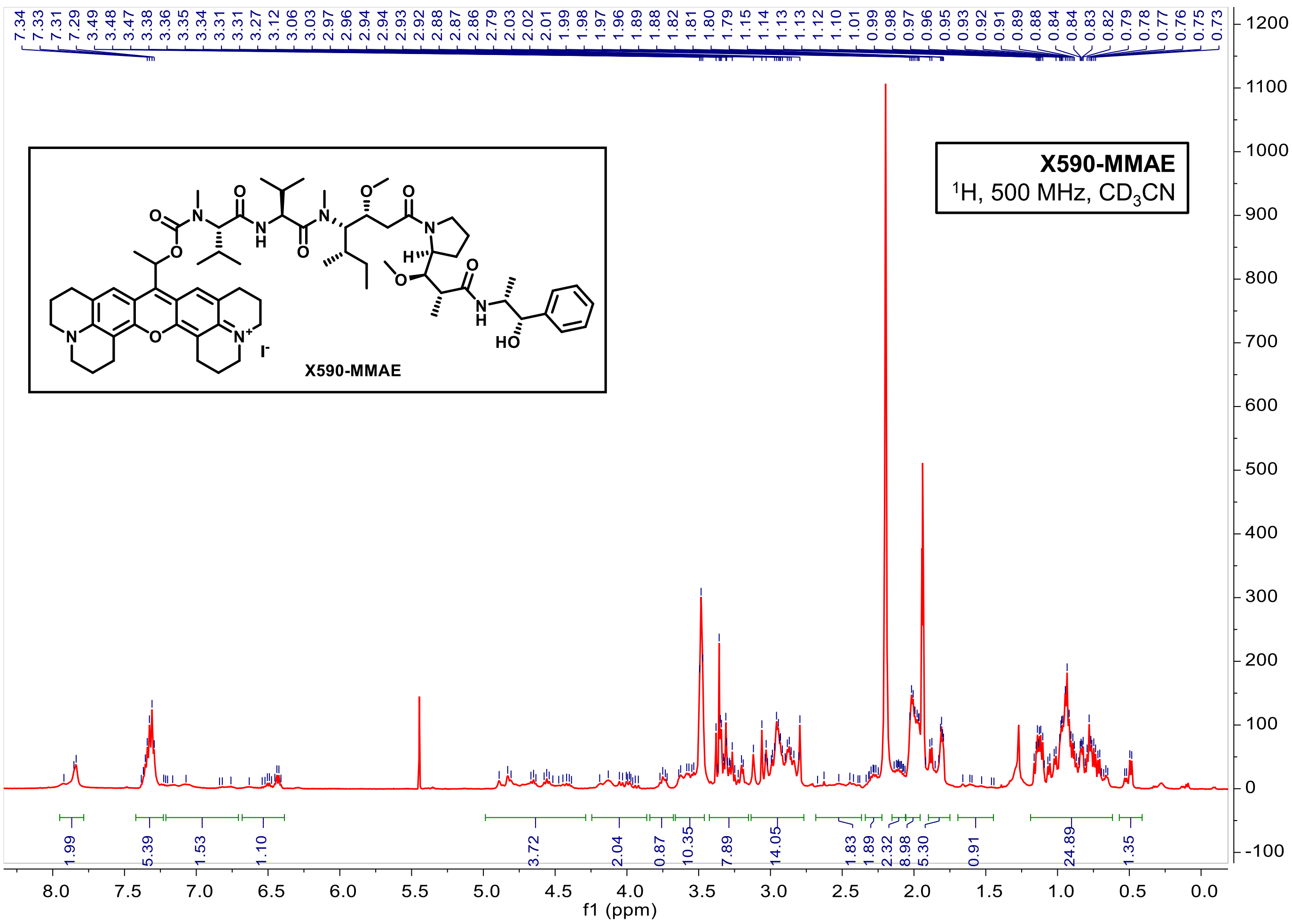
X590-MMAE
¹H, 500 MHz, CD3CN
X590-MMAE
f1 (ppm)

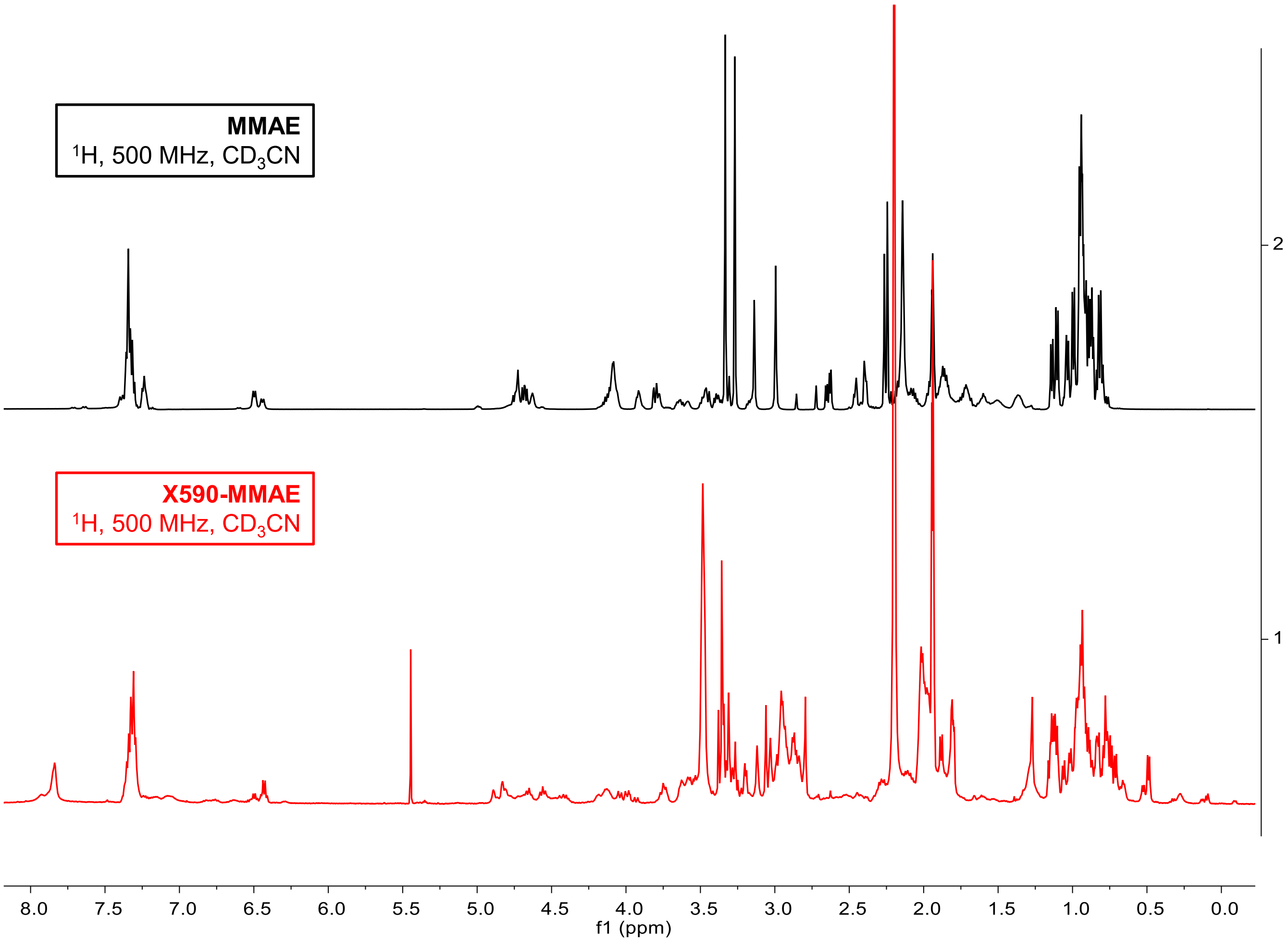
MMAE
^{1}H, 500 MHz, CD_3CN
X590-MMAE
^{1}H, 500 MHz, CD_3CN
8.0 7.5 7.0 6.5 6.0 5.5 5.0 4.5 4.0 3.5 3.0 2.5 2.0 1.5 1.0 0.5 0.0
f1 (ppm)
2
1

Chromatogram

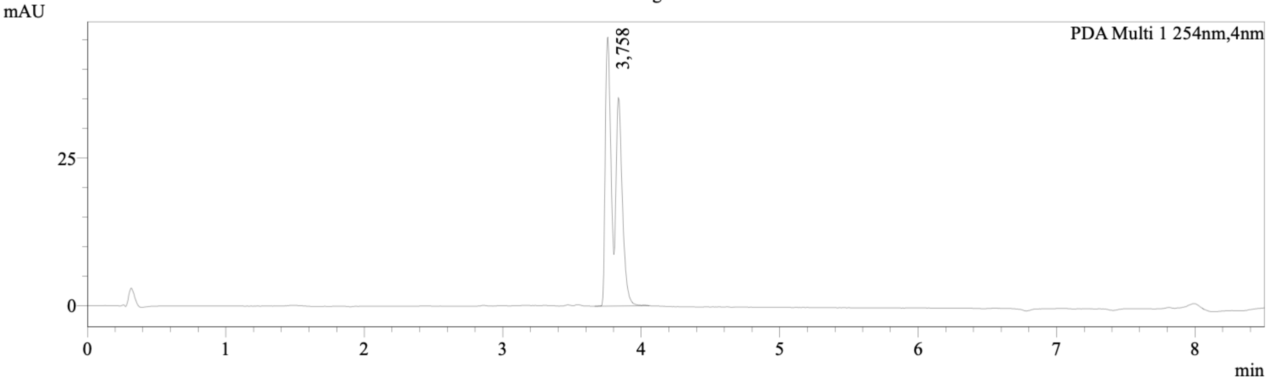

Peak Table

PDA Ch1 254nm

Peak#	Ret. Time	Peak Start	Peak End	Area	Height	Area/Height
1	3,758	3,664	4,059	241564	45444	5,316
Total				241564	45444	

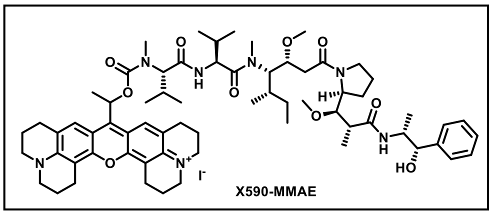

Contour

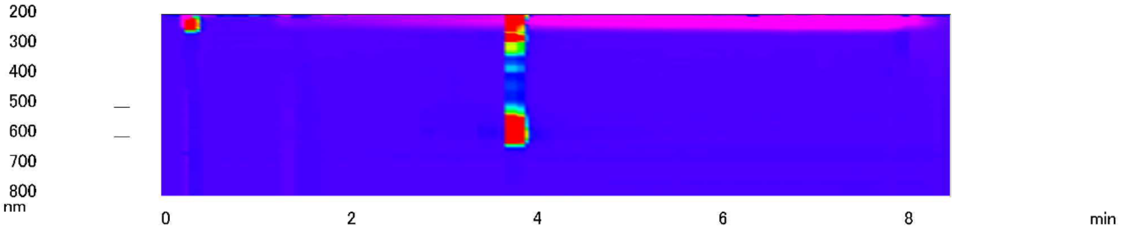

MS Chromatogram

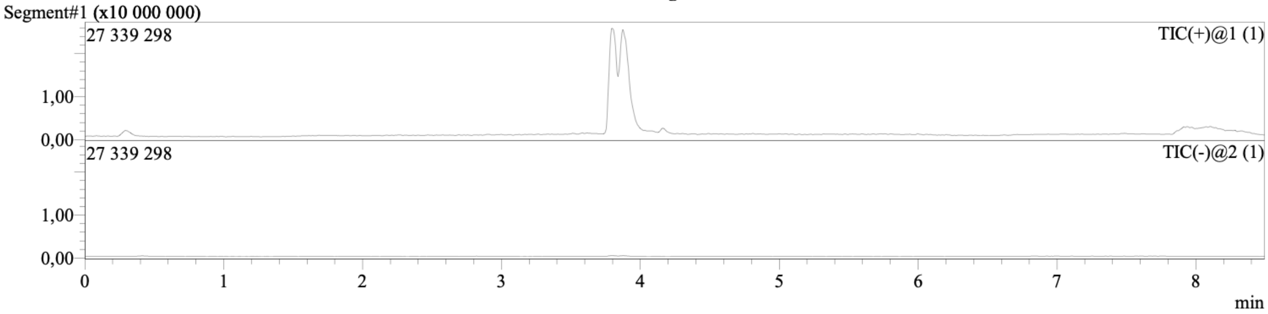

MS Spectrum

Line#:1 R.Time:----(Scan#:----)
MassPeaks:727
Spectrum Mode:Averaged 3,727-4,013(1119-1205) Base Peak:580(6842734)
BG Mode:Averaged 2,033-2,493(611-749) Segment 1 - Event 1

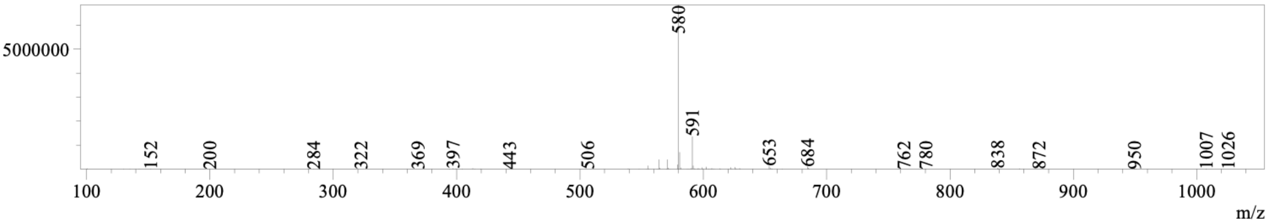

Chromatogram

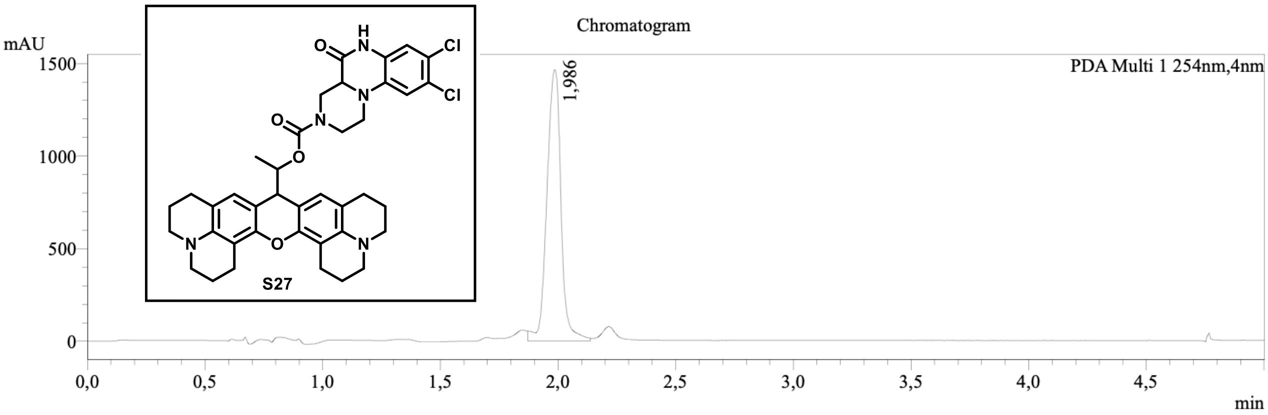

Peak Table

PDA Ch1 254nm

Peak#	Ret. Time	Peak Start	Peak End	Area	Height	Area/Height
1	1,986	1,872	2,136	6107308	1458081	4,189
Total				6107308	1458081	

Contour

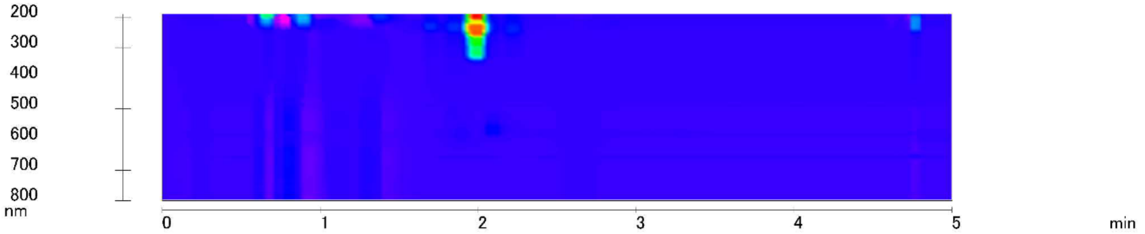

MS Chromatogram

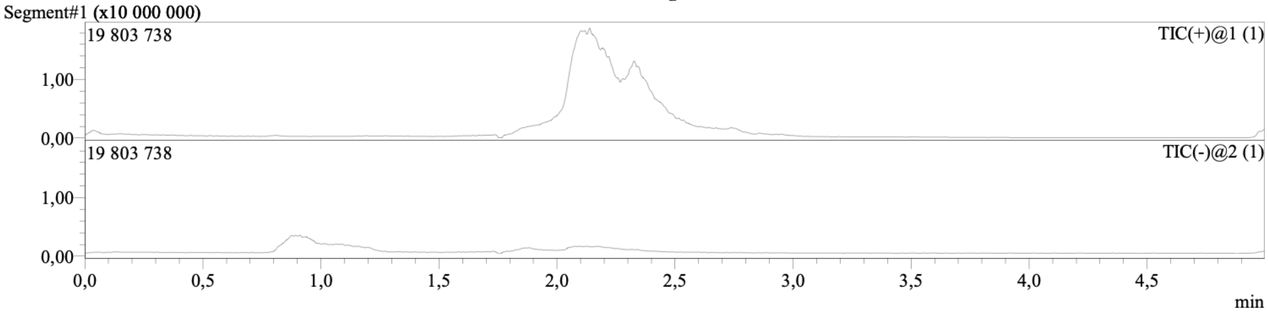

MS Spectrum

Line#:1 R.Time:----(Scan#:----)
MassPeaks:872
Spectrum Mode:Averaged 1,910-2,527(1147-1517) Base Peak:358(1729246)
BG Mode:Averaged 0,527-1,223(317-735) Segment 1 - Event 1

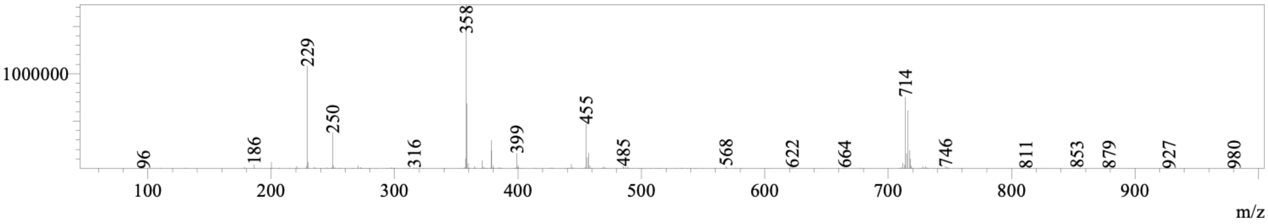

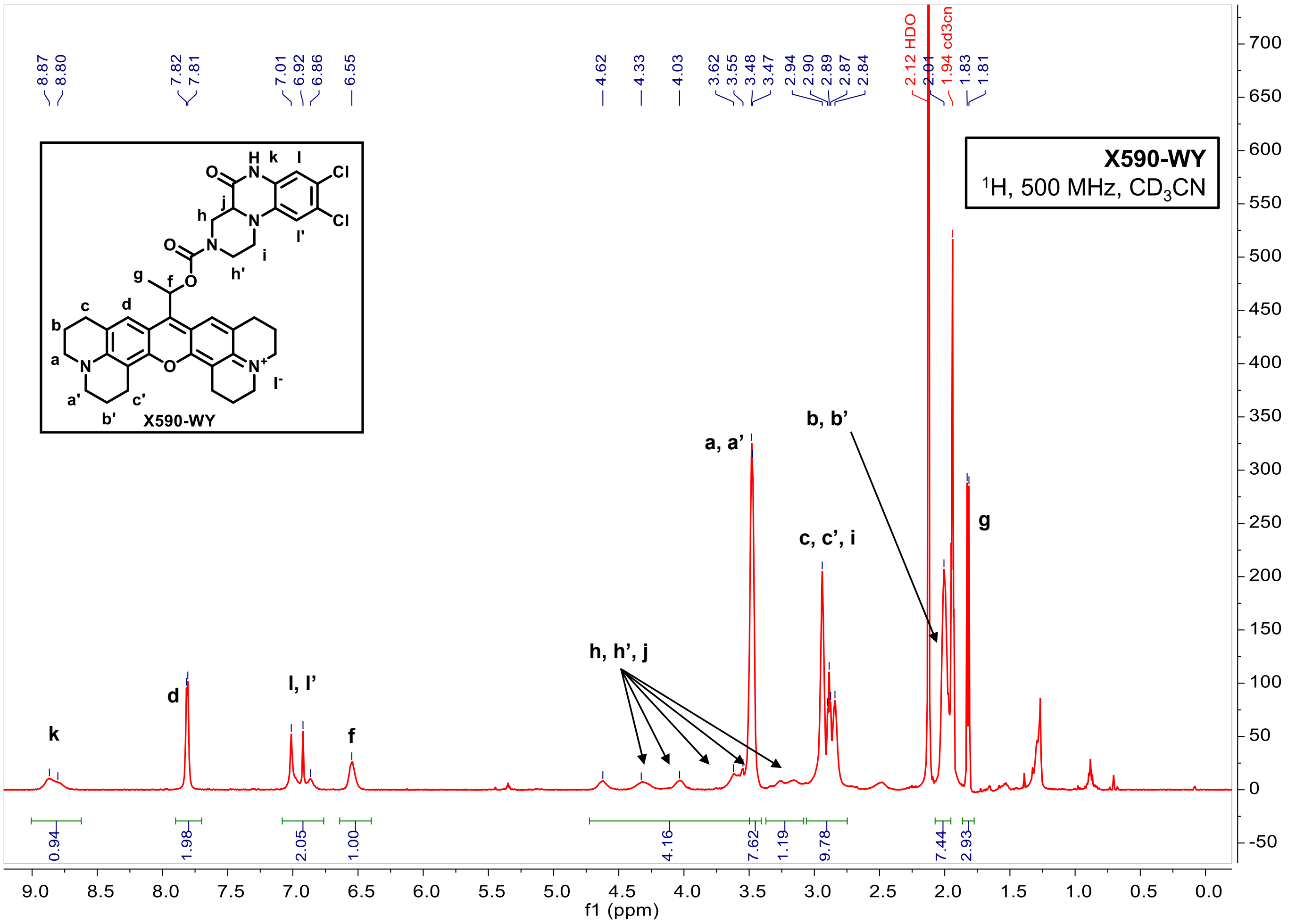
X590-WY
^{1}H, 500 MHz, CD_3CN
k
d
l, l'
f
h, h', j
a, a'
c, c', i
b, b'
g
2.12 HDO
1.94 cd3cn
f1 (ppm)

Chromatogram

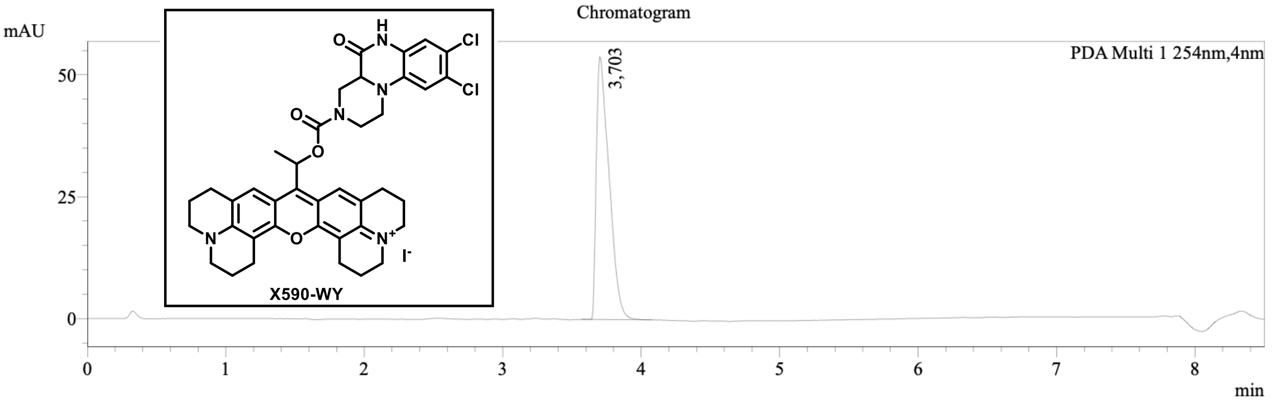

Peak Table

PDA Ch1 254nm

Peak#	Ret. Time	Peak Start	Peak End	Area	Height	Area/Height
1	3,703	3,568	4,080	345167	53927	6,401
Total				345167	53927	

Contour

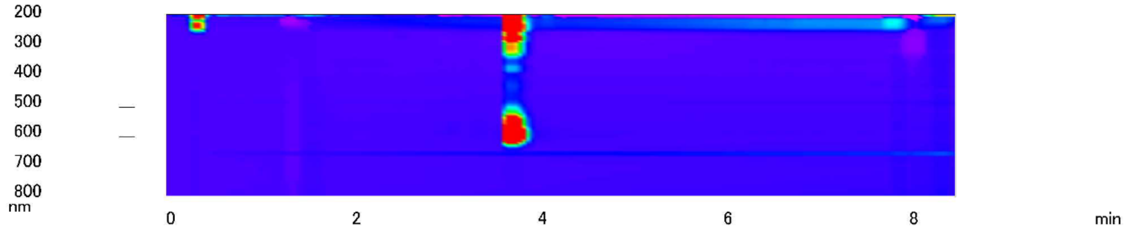

MS Chromatogram

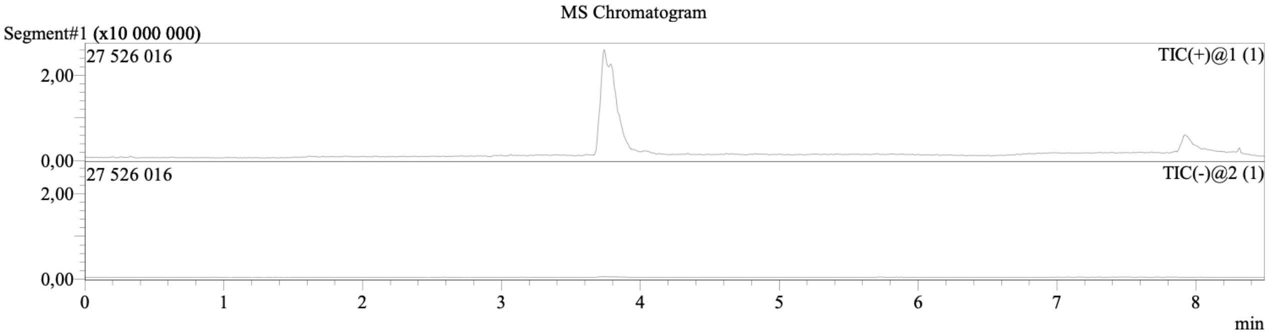

MS Spectrum

Line#:1 R.Time:----(Scan#:----)
MassPeaks:640
Spectrum Mode:Averaged 3,600-4,053(1081-1217) Base Peak:712(2827739)
BG Mode:Averaged 1,707-2,987(513-897) Segment 1 - Event 1

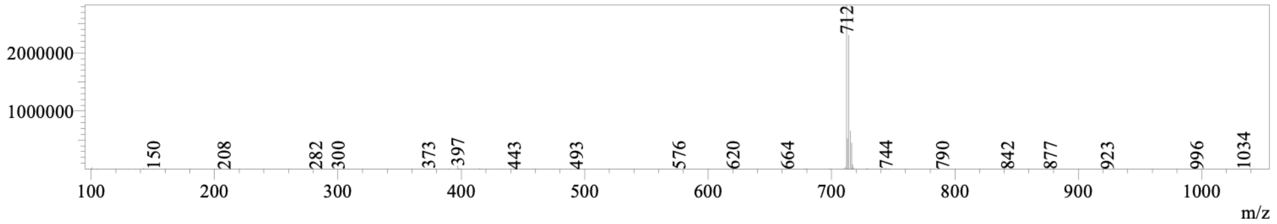

Chromatogram

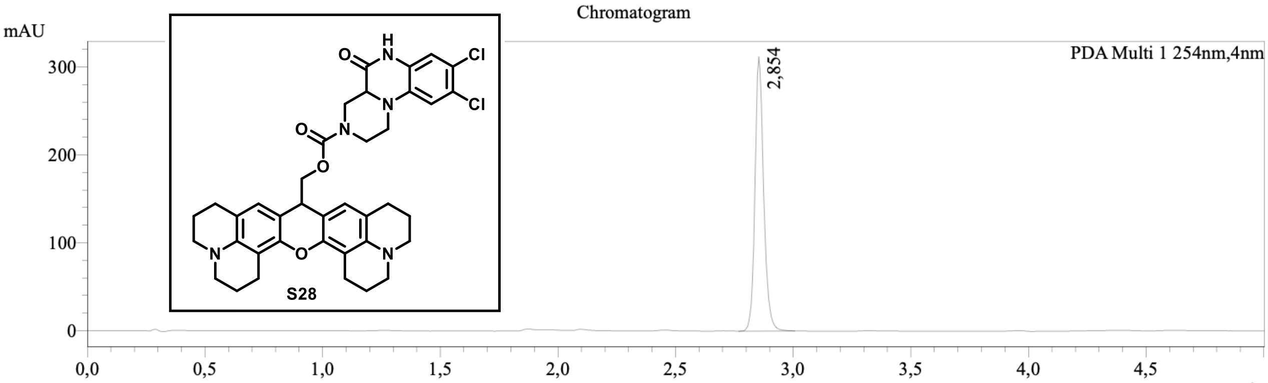

Peak Table

PDA Ch1 254nm

Peak#	Ret. Time	Peak Start	Peak End	Area	Height	Area/Height
1	2,854	2,768	3,008	802654	312157	2,571
Total				802654	312157	

Contour

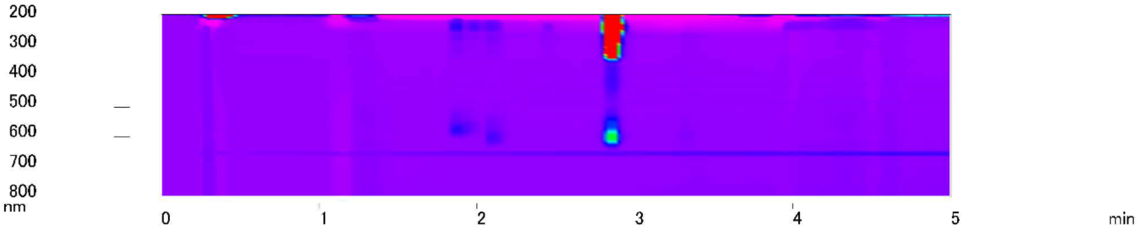

MS Chromatogram

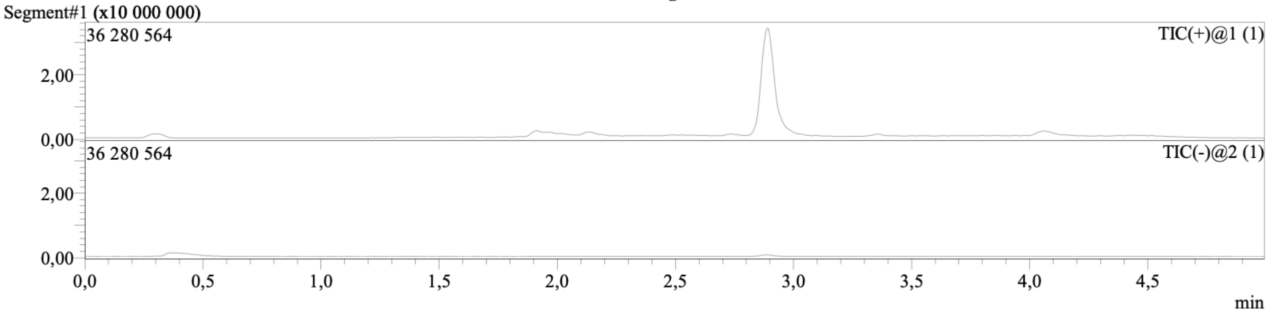

MS Spectrum

Line#:1 R.Time:----(Scan#:----)
MassPeaks:835
Spectrum Mode:Averaged 2,773-3,040(833-913) Base Peak:371(1364932)
BG Mode:Averaged 0,813-1,360(245-409) Segment 1 - Event 1

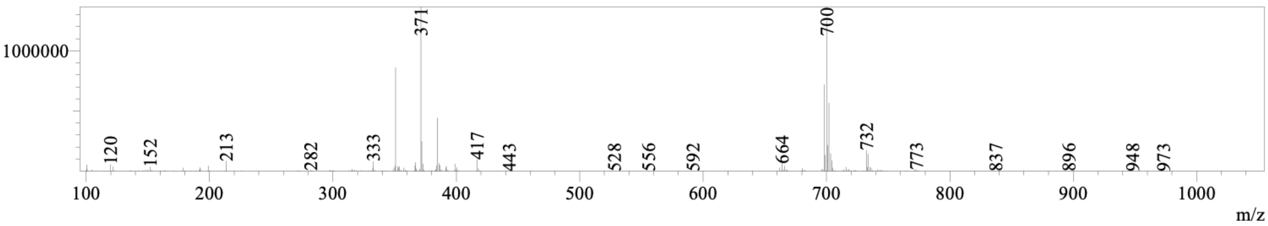

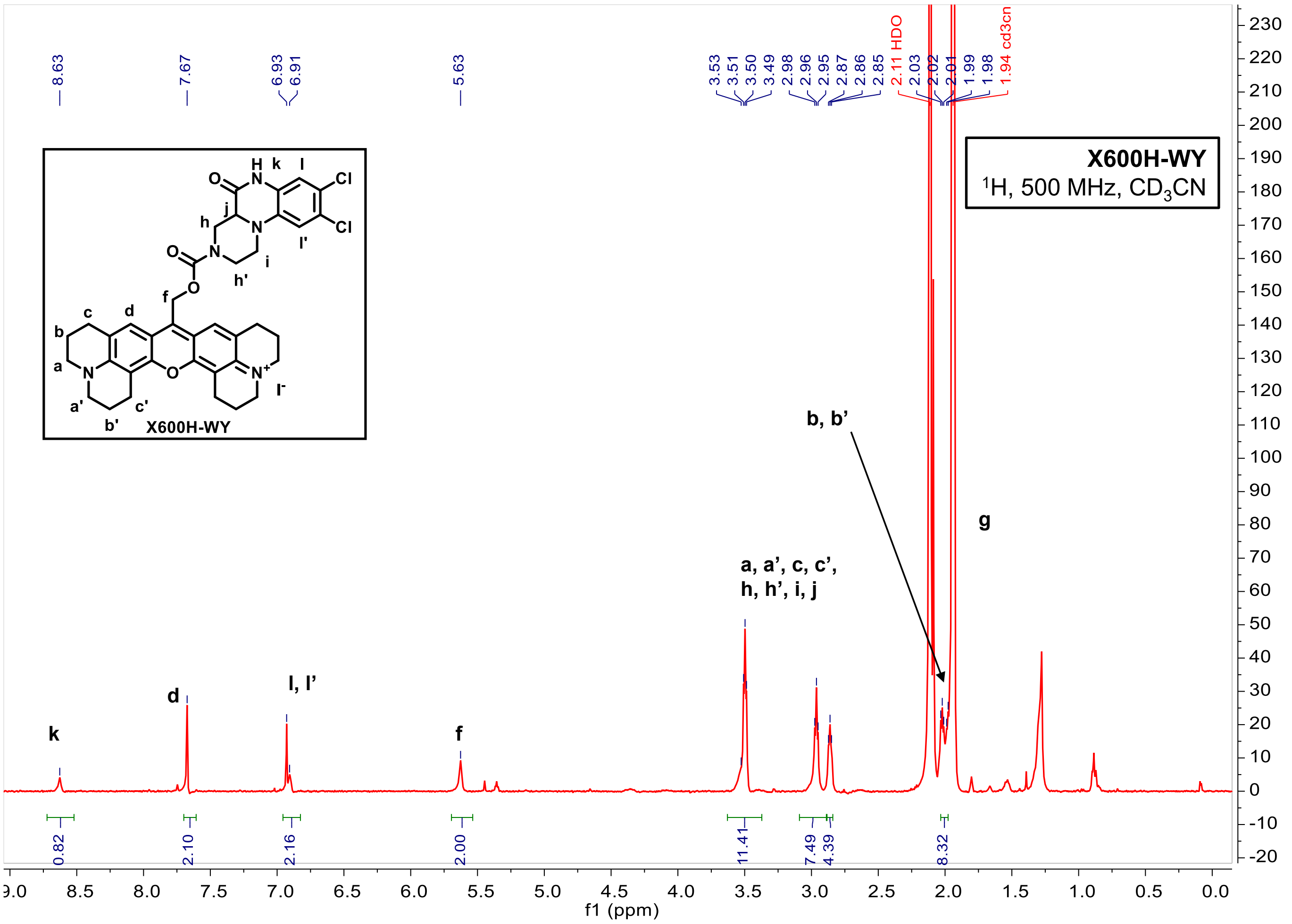
X600H-WY
^{1}H, 500 MHz, CD_3CN
k
d
l, l'
f
a, a', c, c',
h, h', i, j
b, b'
g
8.63
7.67
6.93
6.91
5.63
3.53
3.51
3.50
3.49
2.98
2.96
2.95
2.87
2.86
2.85
2.11 HDO
2.03
2.02
2.01
1.99
1.98
1.94 cd3cn
0.82
2.10
2.16
2.00
11.41
7.49
4.39
8.32
f1 (ppm)
X600H-WY

Chromatogram

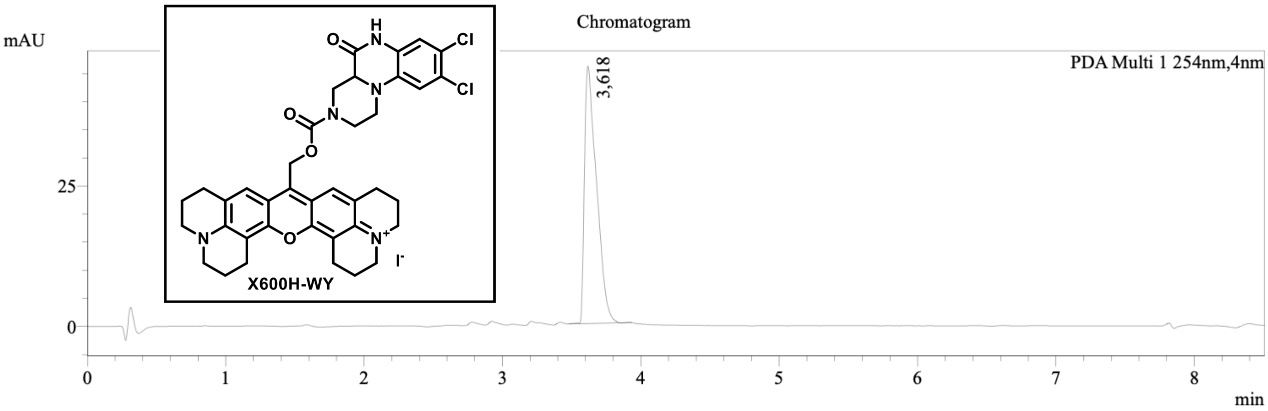

Peak Table

PDA Ch1 254nm

Peak#	Ret. Time	Peak Start	Peak End	Area	Height	Area/Height
1	3,618	3,477	3,936	277561	45834	6,056
Total				277561	45834	

Contour

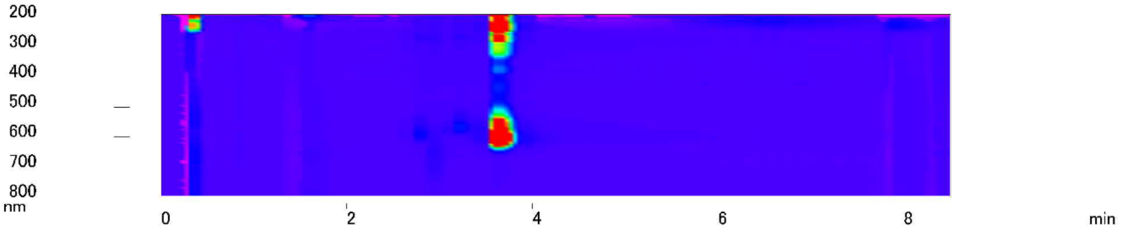

MS Chromatogram

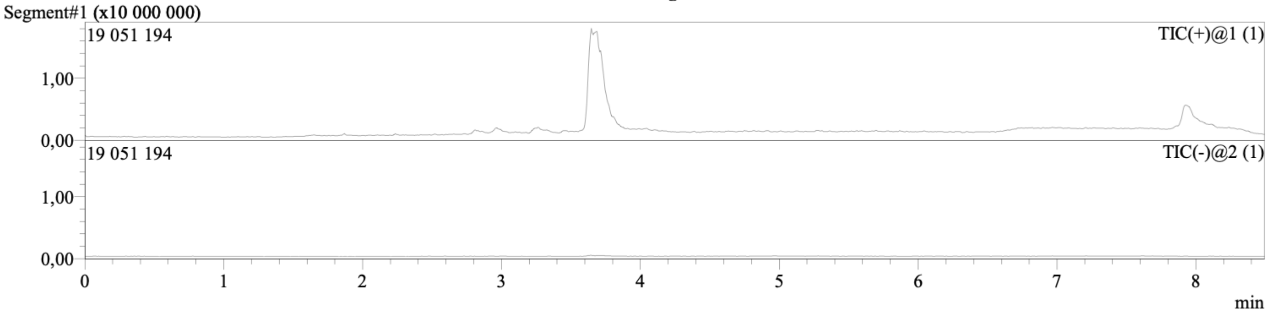

MS Spectrum

Line#:1 R.Time:----(Scan#:----)
MassPeaks:766
Spectrum Mode:Averaged 3,520-3,940(1057-1183) Base Peak:698(2095433)
BG Mode:Averaged 0,513-1,393(155-419) Segment 1 - Event 1

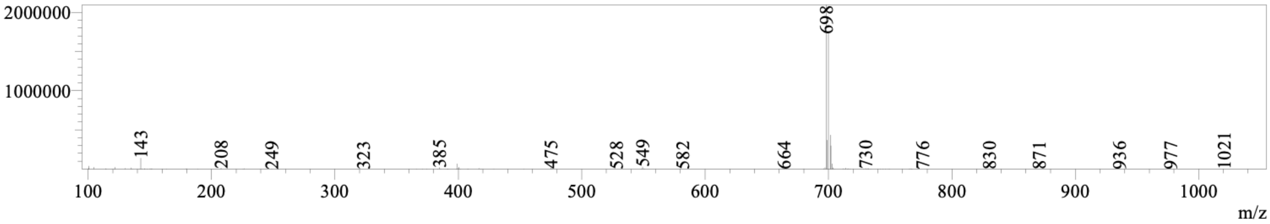

Supplement: Supplementary file 1 [file ja6c02825_si_001.pdf]
