# Supplementary material for: Optimized Xanthenium Photocages with Fused Ring Systems for Photoactivated Chemotherapy and G Protein-Coupled Receptor Photopharmacology
Source: J Am Chem Soc. 2026 Apr 8;148(15):16379–93. doi: 10.1021/jacs.6c02825 (PMC13107459; doi:10.1021/jacs.6c02825)
Supplement: Supplementary file 2 [file ja6c02825_si_002.pdf]

Supporting Information (SI)

Optimized xanthenium photocages with fused ring systems for photoactivated chemotherapy and G protein-coupled receptor photopharmacology

Tibor Á. Molnár,[a,b] Eszter Kozma,[a] Anna Benedikti,[a] Márk Holczer,[a] Attila Kormos,[a] Zsombor Gonda,[a] Ákos Balde, [a] Selina Pühringer,[c] Verena Handl,[c] Christian Bayer,[c] Waltraud Huber,[d] Nassim Ghaffari-Tabrizi-Wizsy,[d] Linda Waldherr,[c,e,*] Krisztina Németh,[a,*] Márton Bojtár[a,*]

[a] MTA – HUN-REN TTK Lendület "Momentum" Chemical Biology Research Group, Institute of Organic Chemistry, HUN-REN Research Centre for Natural Sciences. Magyar tudósok krt. 2. H-1117, Budapest, Hungary. e-mail: bojtar.marton@ttk.hu; nemeth.krisztina@ttk.hu

[b] Hevesy György PhD School of Chemistry, Eötvös Loránd University, Pázmány Péter sétány 1/A, 1117 Budapest

[c] Gottfried Schatz Research Center – Division of Medical Physics and Biophysics, Medical University of Graz, 8010 Graz, Austria. e-mail: linda.waldherr@medunigraz.at

[d] Otto Loewi Research Center – Division of Immunology, Research Unit CAM Lab, Medical University of Graz, 8010 Graz, Austria

[e] BioTechMed-Graz, Austria, Auenbruggerplatz 30, 8036 Graz, Austria

Contents

1. MATERIALS AND METHODS ... 2

2. DEACTIVATION PATHWAYS FOR CX640-PP ... 3

2.1 Characterization of the exo form ... 3
2.2 Long-term aqueous stability of CX640-PP ... 5

3. SYNTHESIS OF THE COMPOUNDS ... 7

3.1 Synthetic Overview ... 7
3.2 Synthesis of the Model Compounds ... 10
3.3 Synthesis of the Prodrugs ... 19
3.4 Synthesis of the Caged WY Compounds ... 20

4. SPECTROSCOPIC PROPERTIES OF THE COMPOUNDS ... 23

5. PHOTOCHEMICAL STUDIES ... 25

5.1 Experimental Details for Photochemical Studies ... 25
5.2 Uncaging Experiments Followed by HPLC-MS ... 26
5.3 Uncaging Experiments Followed by Optical Spectroscopy Methods ... 36
5.4 Dark Stability ... 38
5.5 Photochemical and Singlet Oxygen Quantum Yield Determination ... 44

6. 2D CELL CULTURE FOR PHOTOACTIVATED CHEMOTHERAPY EXPERIMENTS ... 46

6.1 Cell Viability Studies ... 46
6.2 Microtubule Imaging ... 49
6.3 Colocalization Studies ... 55

7. **SPHEROID EXPERIMENTS** 57

7.1 Experimental Details for the Spheroid Experiments 57
7.2 Spheroid Images 57

8. **CAM ASSAY** 60

8.1 Light Source Developed for the CAM Assay 60
8.2 Experimental Details for the CAM Assay 62
8.3 Survival Plot 62

9. **GPCR PHOTOACTIVATION** 63

9.1 Molecular cloning 63
9.2 Cell culture 63
9.3 Confocal imaging and photoactivation 63
9.4 Exemplary Workflow for the Analysis 64
9.5 Calcium Imaging of Uncaging 65

10. **REFERENCES** 68

1. Materials and Methods

All starting materials were purchased from commercial suppliers (Sigma Aldrich, Fluorochem, Merck, Alfa Aesar, Acros, Doug Discovery, Molar Chemicals) and used without further purification. MMAE (99.97%) was purchased from MedChemExpress. Reaction monitoring was performed by analytical thin-layer chromatography (TLC) on Merck silica gel 60 F254 precoated aluminum plates. Flash column chromatography was performed using a Teledyne ISCO CombiFlash automated system with RediSep Gold columns. NMR spectra were recorded on a Varian Inova 500 MHz or a Varian Inova 300 MHz spectrometer. Chemical shifts (δ) are reported in parts per million (ppm) relative to residual solvent signals or TMS. Coupling constants are given in hertz (Hz). Analytical reversed-phase HPLC-UV/Vis-MS analyses were performed on a Shimadzu LC-MS-2020 system equipped with a Gemini C18 column (100 × 2.0 mm, 5 µm, 110 Å). Detection was achieved using a diode array detector (190-800 nm) and an electrospray ionization mass spectrometer (ESI-MS). Two LC-MS gradient elution methods were employed, both using the same mobile phase system and conditions. Eluent A consisted of 94.9% H_2O, 5.0% MeCN, and 0.1% HCOOH; eluent B consisted of 94.9% MeCN, 5.0% H_2O, and 0.1% HCOOH. The flow rate was set to 0.8 mL/min, and the column temperature was maintained at 40 °C. The longer gradient method (8.5 min) used the following profile: 0.00 min – 0% B, 6.00 min – 100% B, 7.00 min – 100% B, 7.50 min – 0% B, 8.50 min – 0% B. The shorter gradient method (5.0 min) used the following profile: 0.00 min – 0% B, 2.50 min – 100% B, 3.13 min – 100% B, 3.75 min – 0% B, 5.00 min – 0% B. Semi-preparative HPLC was conducted using a Wufeng Chrom LC100 system with a Gemini C18 column (150 × 21 mm, 5 µm, 110 Å).

Fluorescence spectra were recorded on a Jasco FP-8300 spectrofluorometer. UV/Vis spectra were collected using a Jasco V-750 spectrophotometer. Either PMMA (for longer aqueous experiments) or quartz cuvettes with 1 cm pathlength were used. Fluorescence quantum yields were determined using rhodamine 101 (in EtOH) as a standard (Φ_{flu} = 0.91) [1]

High-resolution mass spectrometric measurements were performed using a Sciex TripleTOF 5600+ hybrid Quadrupole-TOF LC/MS/MS system in positive electrospray mode.

2. Deactivation Pathways for CX640-PP

2.1 Characterization of the exo form

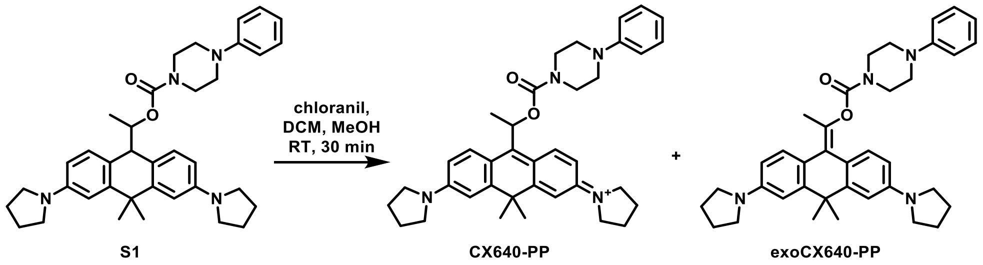

Scheme S1. Synthesis of the exo form of ***CX640-PP*** *[2]*

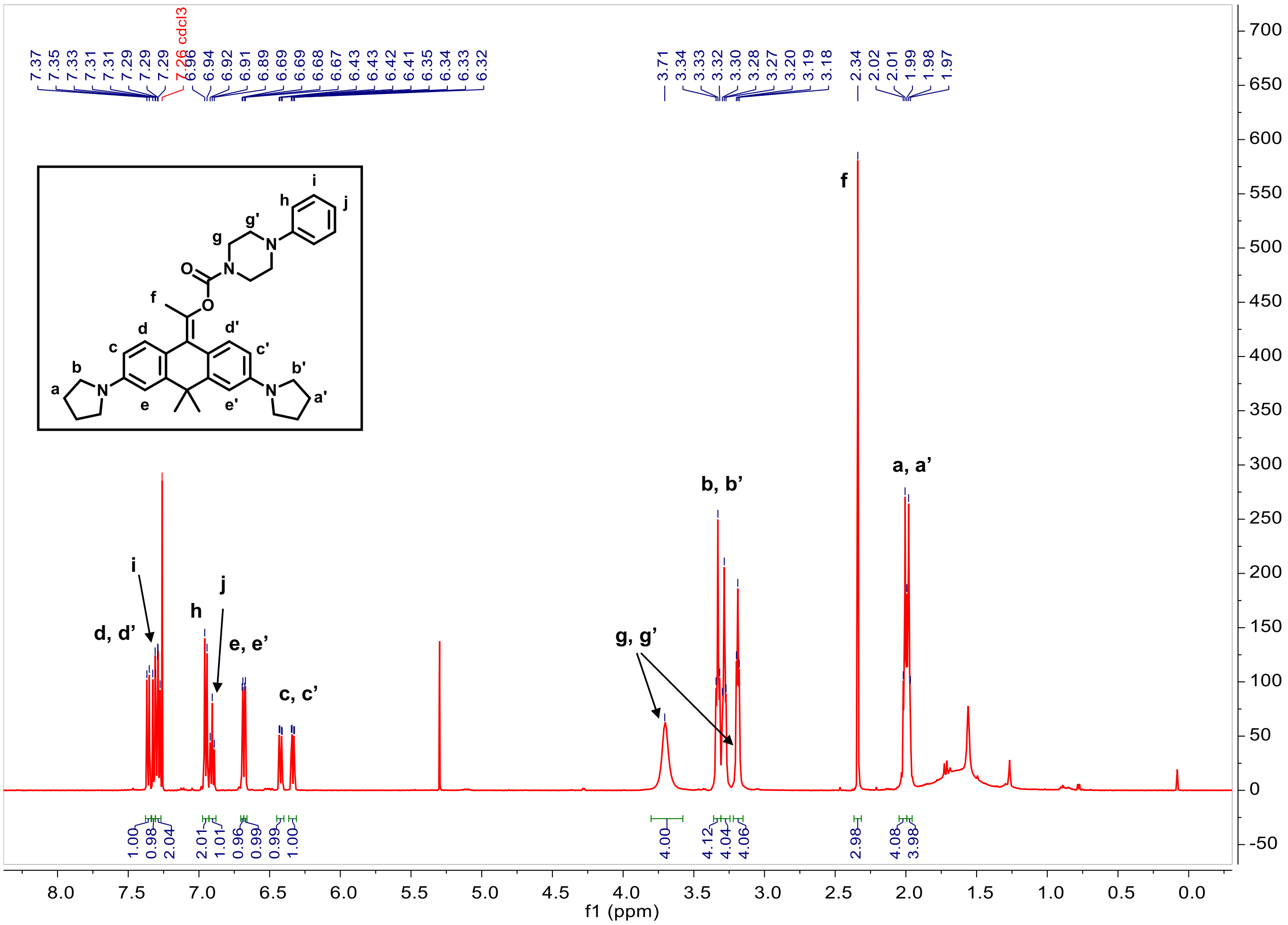

Figure S1. *^{1}H-NMR spectrum of the exo form of* ***CX640-PP***

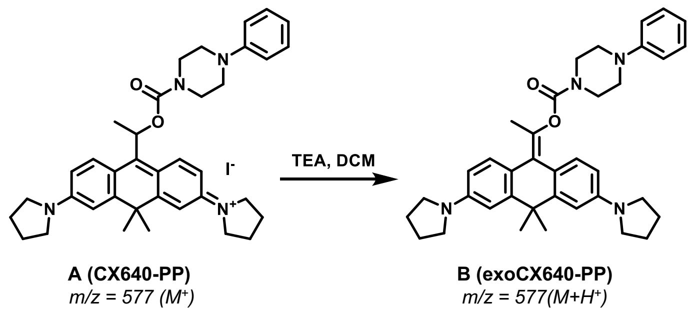

reaction with 10 equivalent triethylamine:

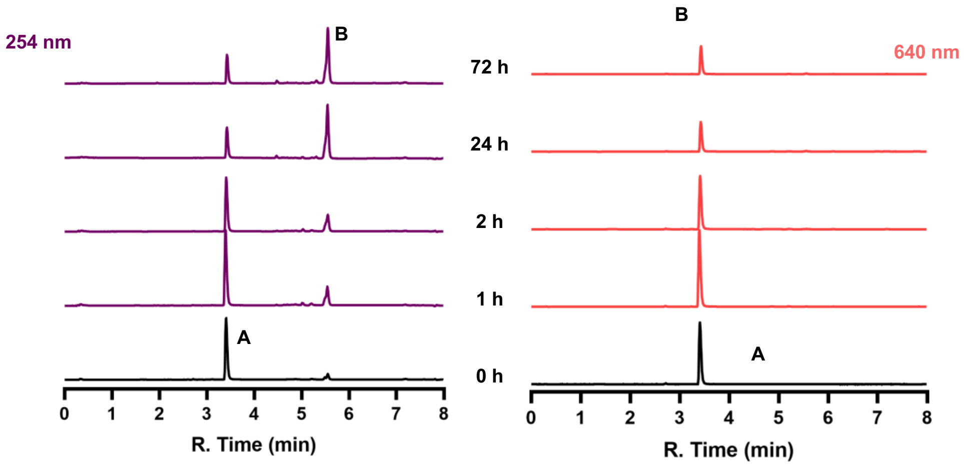

reaction with 10 equivalent triethylamine (2D contour chromatograms):

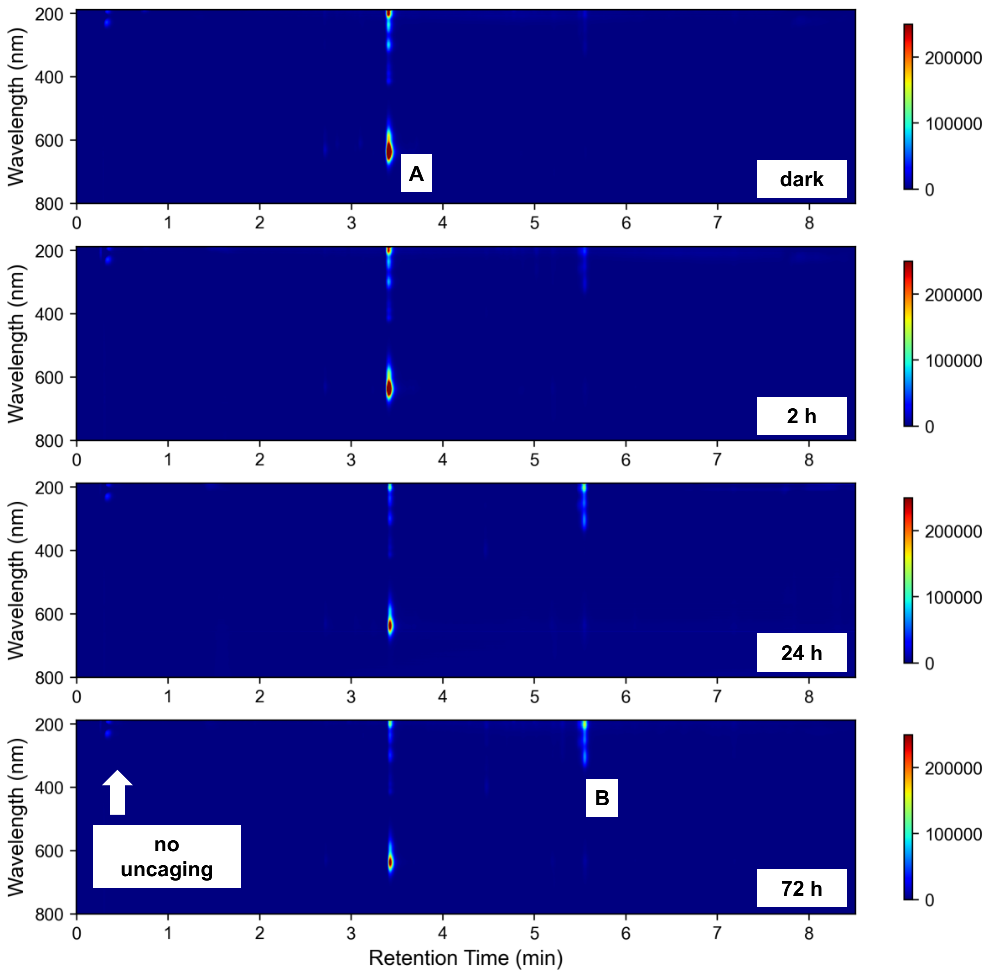

Figure S2. Chromatograms of the conversion of ***CX640-PP*** *(I^-) to the exo form followed by HPLC-MS*

2.2 Long-term aqueous stability of CX640-PP

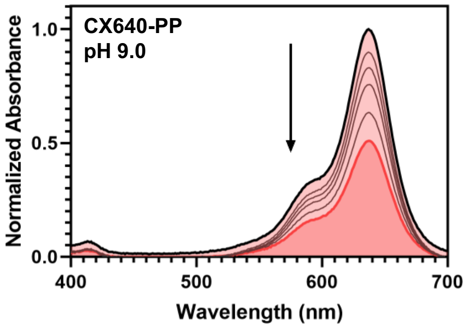

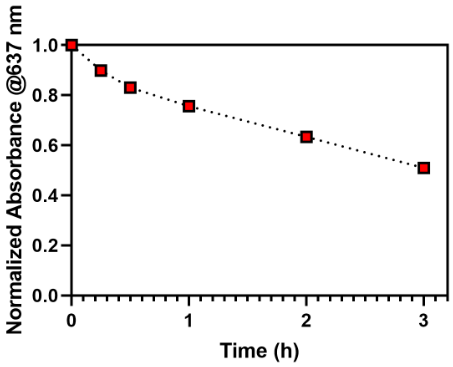

*Figure S3. Normalized absorption spectra of **CX640-PP** (~3 μM, Britton-Robinson buffer, pH 9.0) recorded over time. Normalized absorbance values are plotted on the right.*

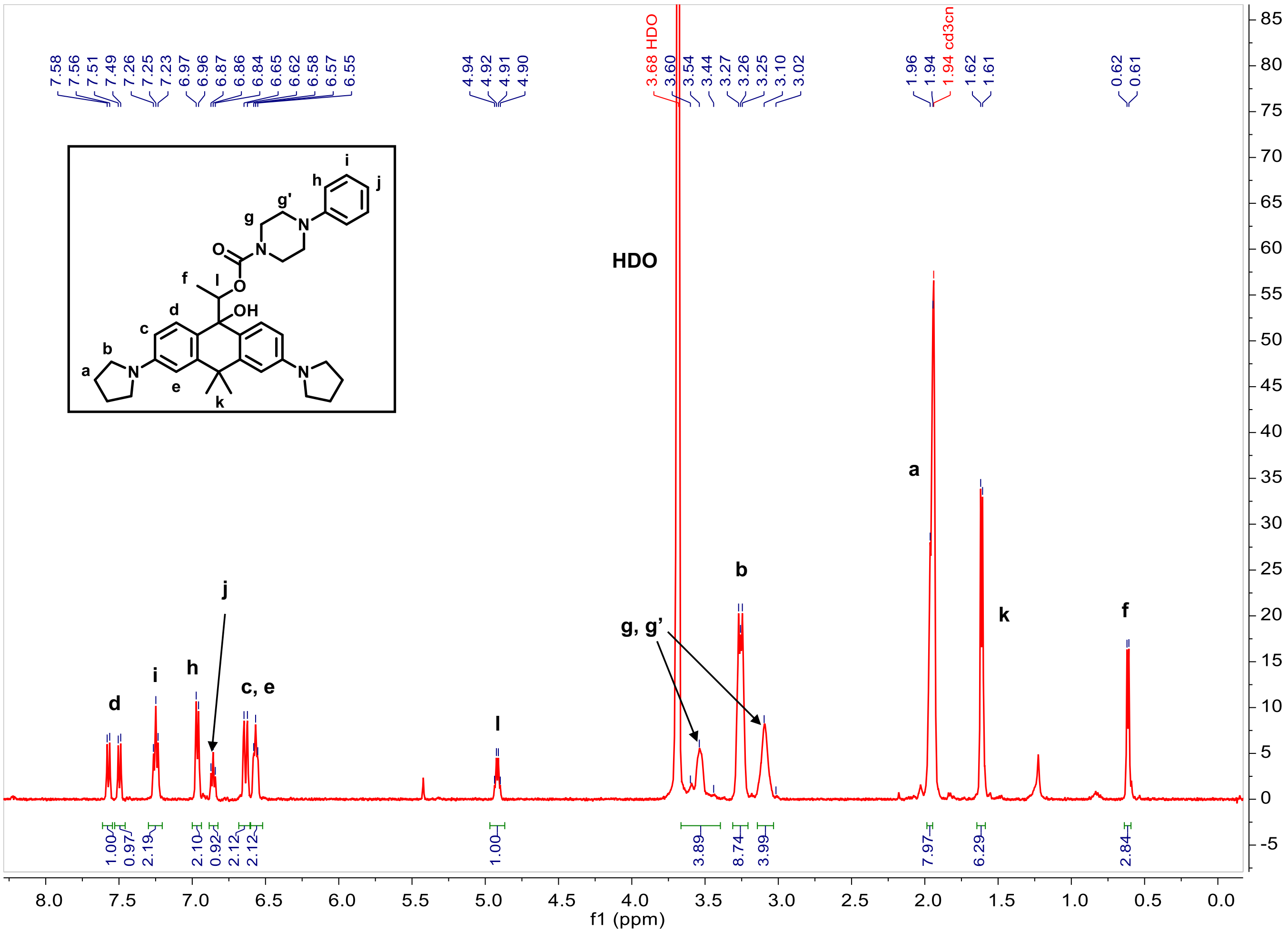

*Figure S4. ^{1}H-NMR spectrum of the leuco (OH-adduct) form of **CX640-PP** in CD_3CN-NaOD-D_2O*

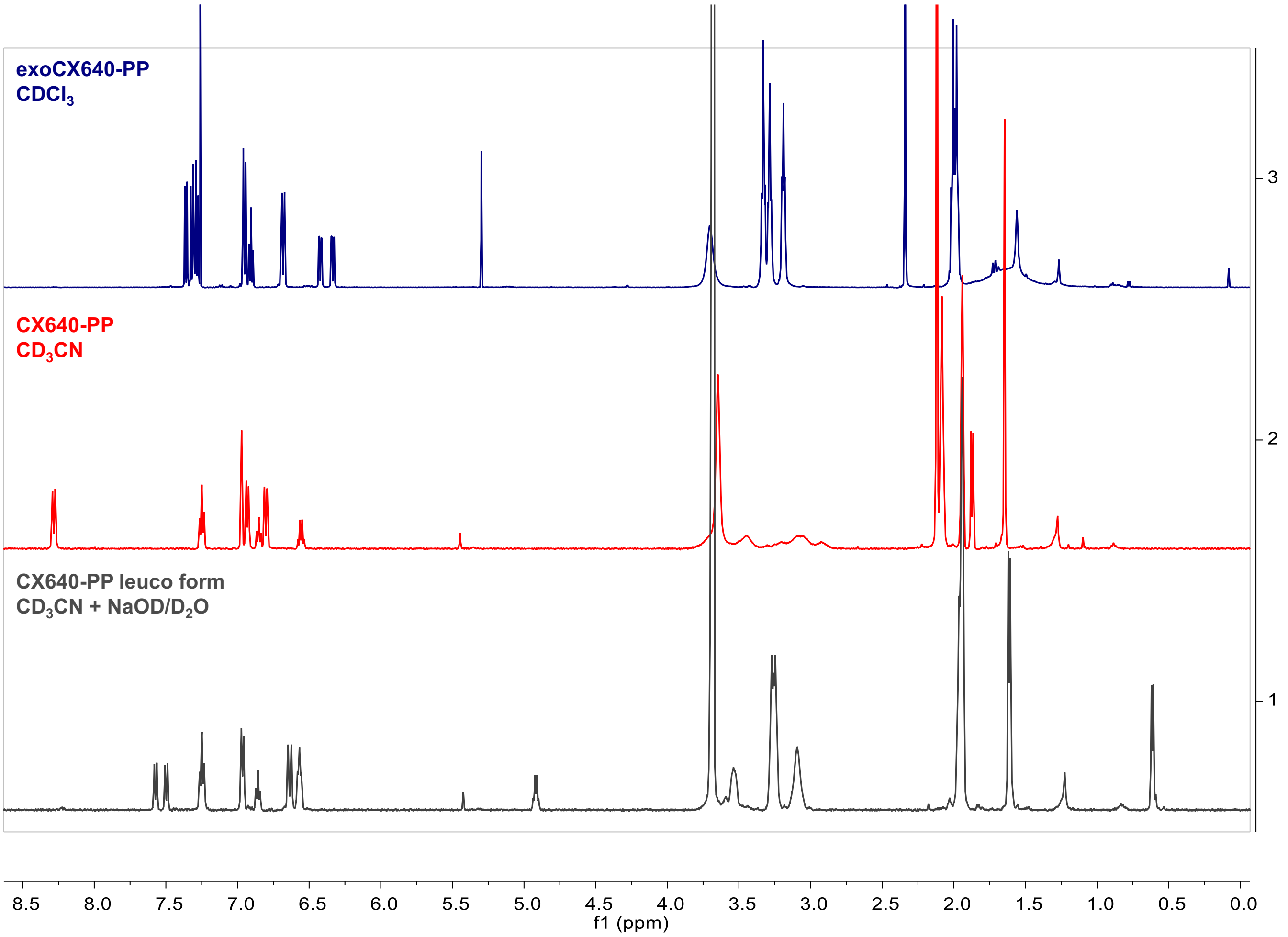

*Figure S5. Stacked ^{1}H-NMR spectra of the multiple forms of **CX640-PP** (for the assignment of CX640-PP, see ref. [2])*

3. Synthesis of the Compounds

3.1 Synthetic Overview

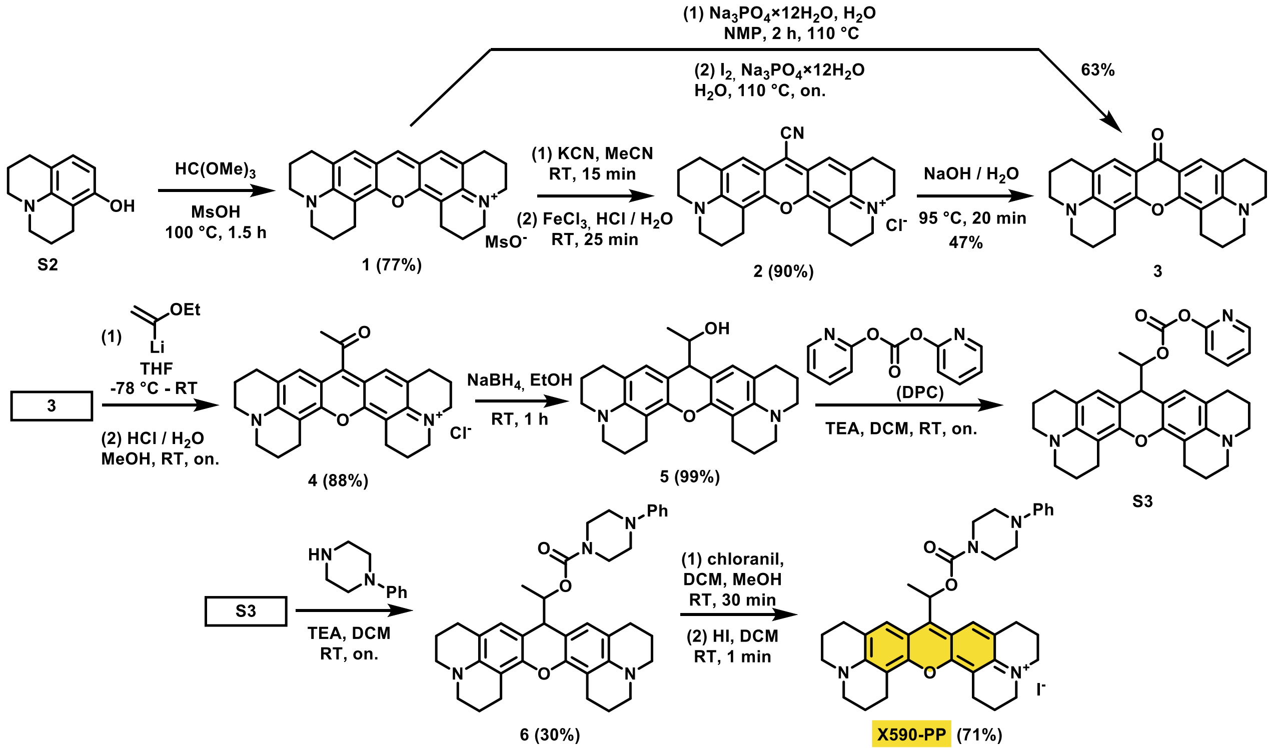

*Scheme S2. Synthesis of **X590-PP**.*

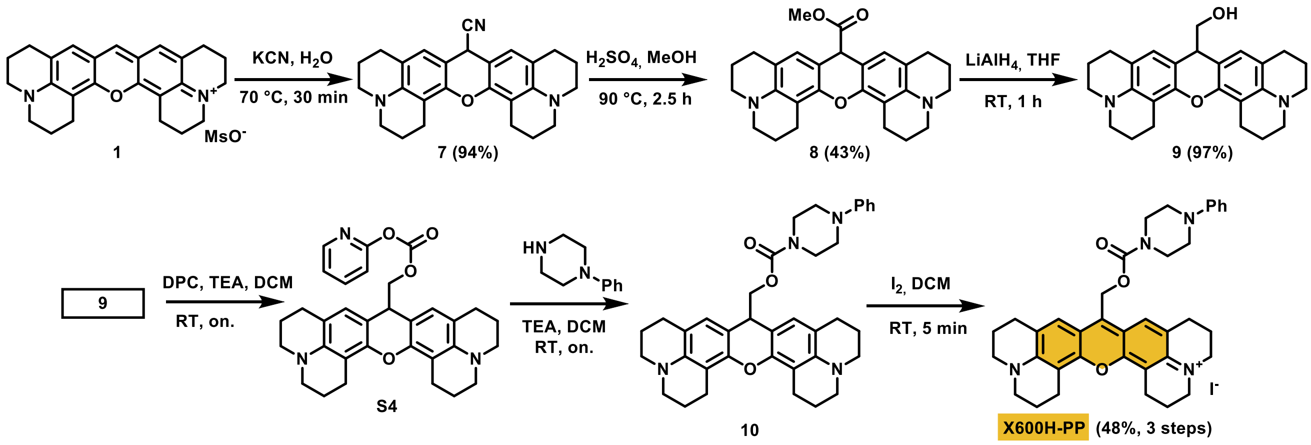

*Scheme S3. Synthesis of **X600H-PP**.*

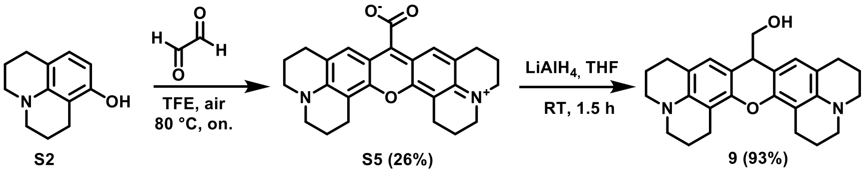

*Scheme S4 Alternative Synthesis of **9***

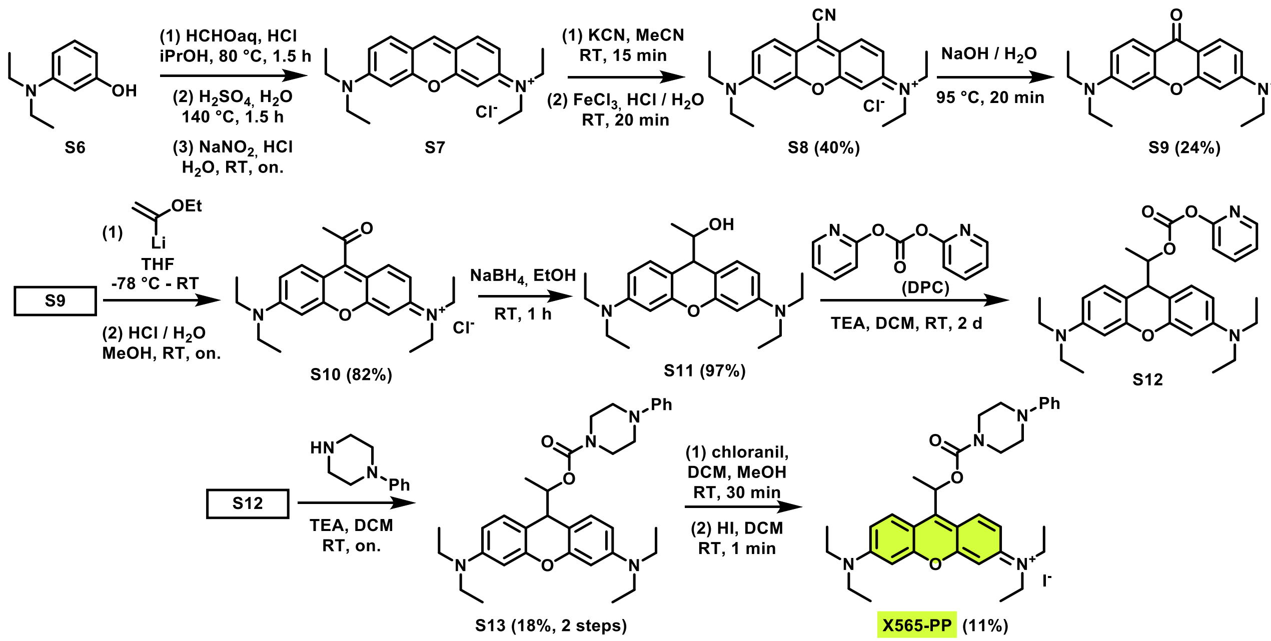

*Scheme S5. Synthesis of **X565-PP**.*

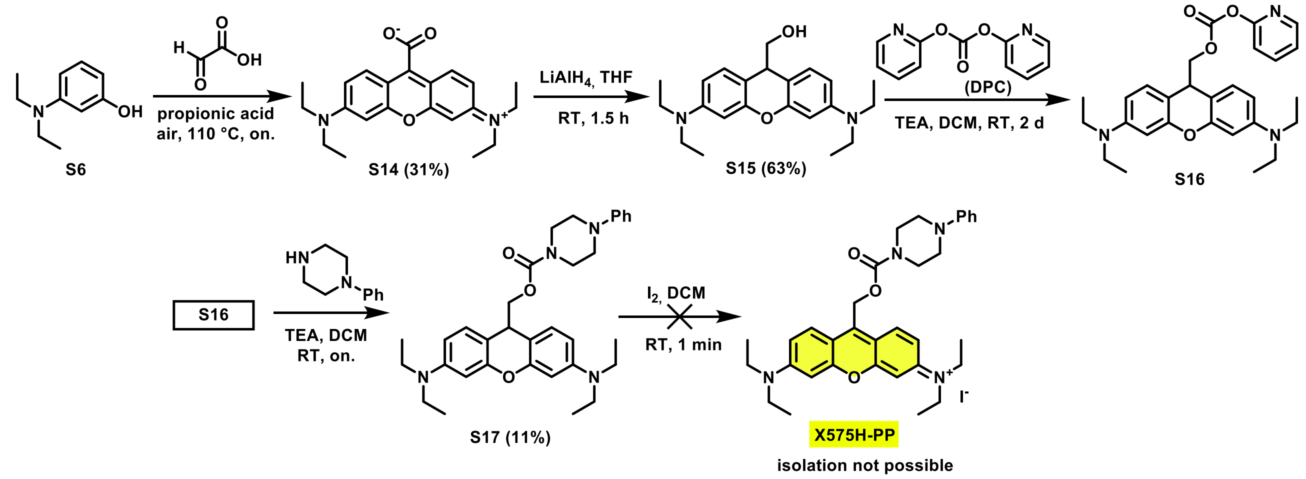

*Scheme S6. Attempted synthesis of **X575-PP**.*

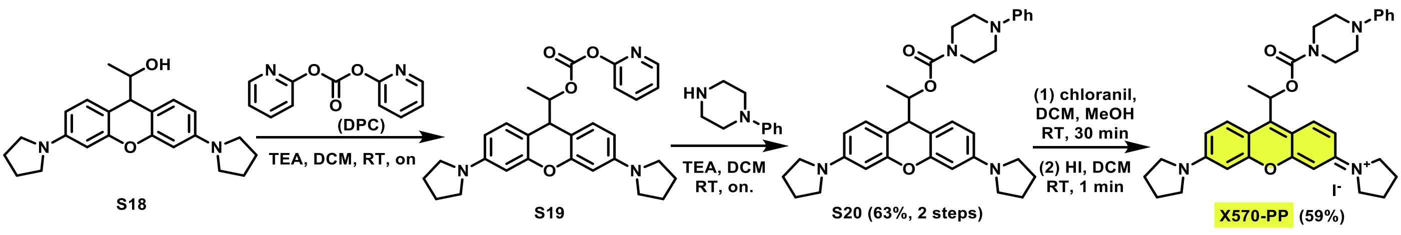

*Scheme S7. Synthesis of **X570-PP**.*

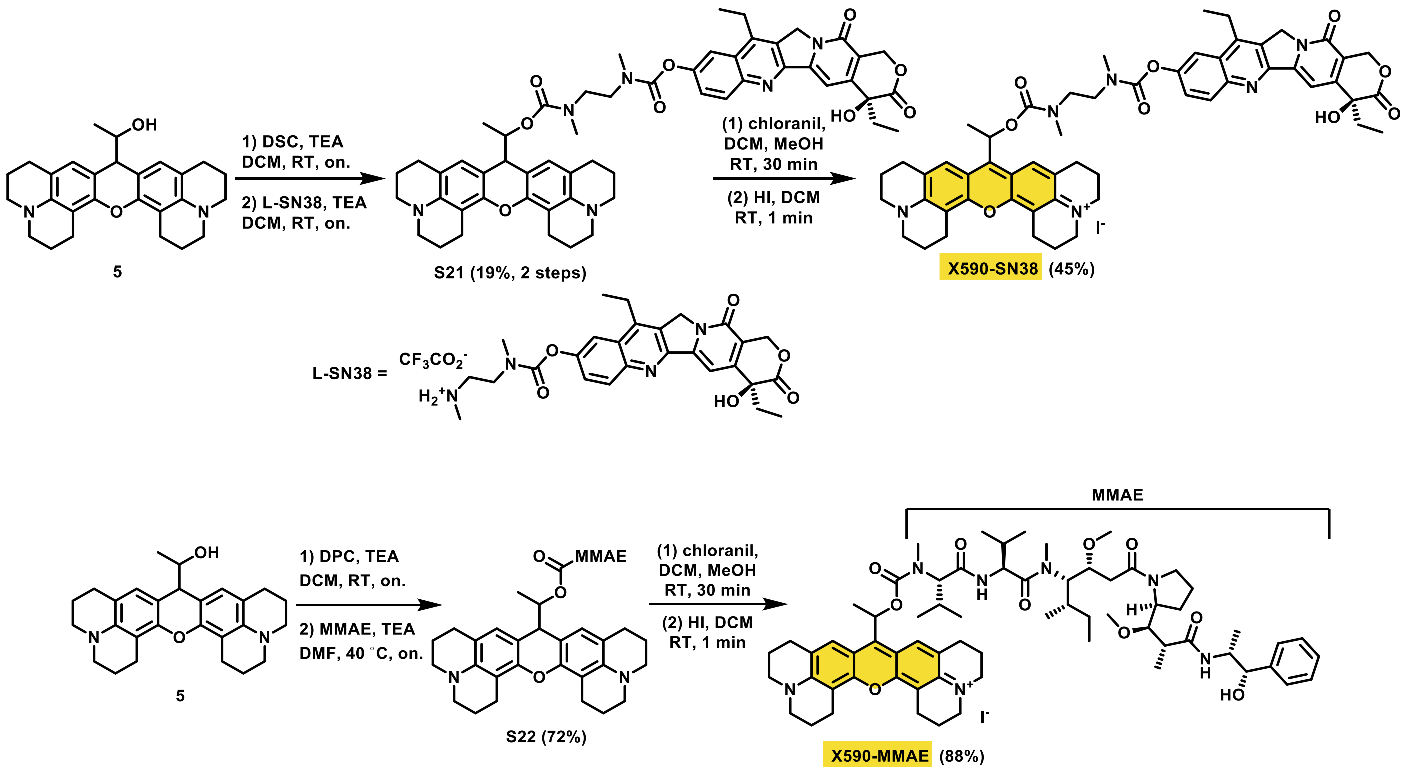

Scheme S8. Synthesis of the prodrugs.

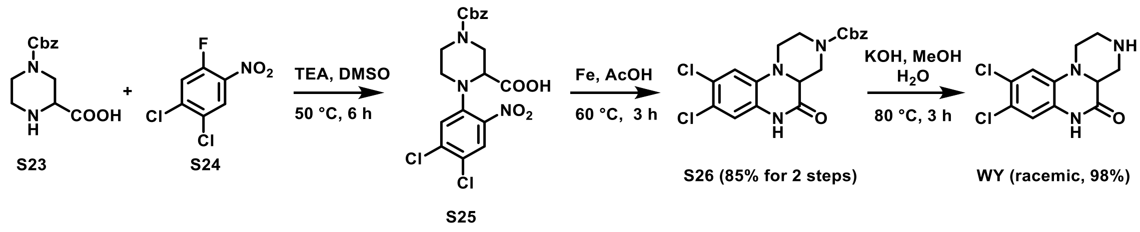

*Scheme S9. Synthesis of **WY**.*

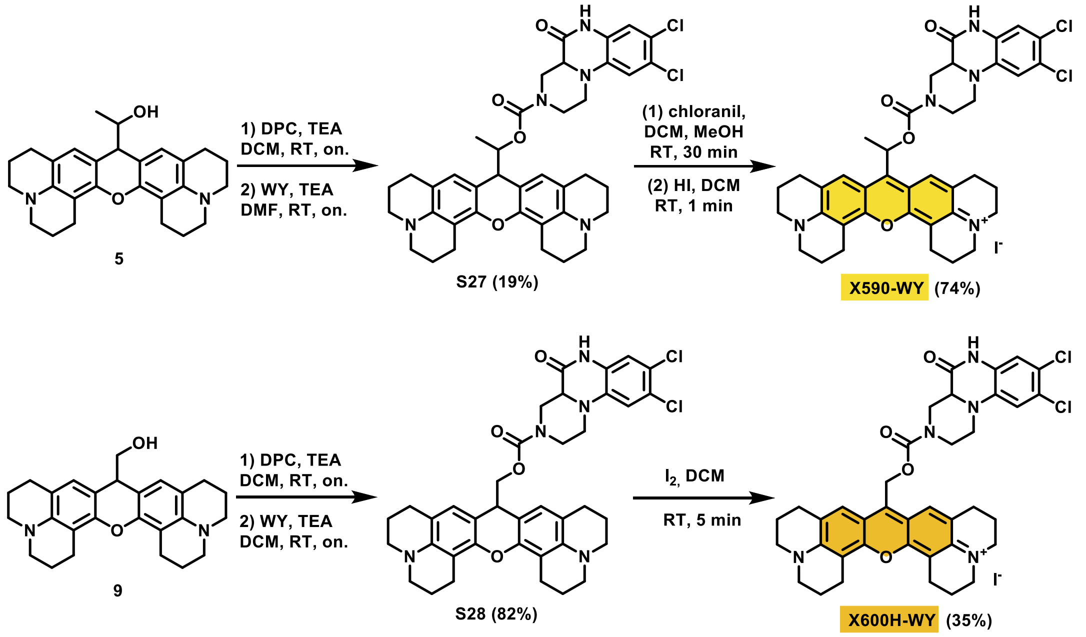

*Scheme S10. Synthesis of the caged **WY** compounds.*

3.2 Synthesis of the Model Compounds

Bisjulolidinyl-pyronine 1 (mesylate salt) [3]

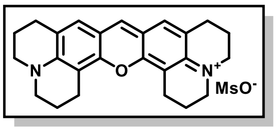

To a round-bottom flask, 8-hydroxyjulolidine (**S2**, 3.02 g, 16.0 mmol) and trimethyl orthoformate, 10.5 ml, 96.0 mmol, 6.0 equiv.) was added together with methanesulfonic acid (6.2 ml, 96.0 mmol, 6 equiv.). The mixture was stirred for an hour at 100 °C under inert atmosphere. Then, cc. NaCl solution (250 ml) was added, and the resulting mixture was extracted with DCM (3 x 500 ml). The combined organic phase was dried over Na_2SO_4, filtered and evaporated. The crude product was purified by column chromatography (eluent: DCM/MeOH 0% to 10%) to afford a purple crystalline solid with intense fluorescence in solution.

Yield: 2.87 g (77%)

^{1}H NMR (500 MHz, DMSO-d_6) δ 8.28 (s, 1H), 7.35 (s, 2H), 3.52 – 3.47 (m, 8H), 2.83 (t, *J* = 6.3 Hz, 4H), 2.79 (t, *J* = 6.0 Hz, 4H), 2.30 (s, 3H), 1.98 – 1.93 (m, 4H), 1.93 – 1.89 (m, 4H).

^{13}C NMR (126 MHz, DMSO-d_6) δ 151.39, 150.86, 142.36, 127.82, 123.26, 112.79, 104.57, 50.30, 49.75, 39.72, 26.67, 20.06, 19.10, 19.01.

HRMS: $[M]^+$: calcd for $[C_{25}H_{27}N_2O]^+$: 371.2117, found: 371.2131.

Rhodamine 800 (chloride salt, 2)

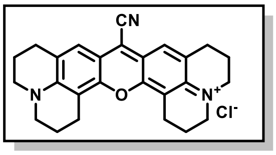

A 250 mL two-neck round-bottom flask was charged with compound **1** (1.01 g, 2.17 mmol) and dissolved in 60 ml of acetonitrile and 30 mL of water. A dropping funnel and gas-absorption trap (loaded with 10% NaOH in water) were attached. Potassium cyanide (0.406 g, 6.23 mmol, 2.3 equiv.) was dissolved in 12 ml of water and then added to the reaction mixture. After 15 min, a calculated amount of $FeCl_3$ solution (3.66 g, 13.55 mmol, 5.0 equiv. $FeCl_3 \cdot 6H_2O$, in 4.0 mL of 2 M HCl, 12 mL of water) was added dropwise. The mixture was stirred for an additional 25 min, then saturated $NaHCO_3$ solution was added, and the mixture was extracted three times with DCM. The combined organic layers were dried over $MgSO_4$ and concentrated. The product (commercially available as rhodamine 800) was obtained as dark-blue crystals and used without further purification.

Yield: 0.835 g (90%)

^{1}H NMR (500 MHz, CD_3CN) δ 7.21 (s, 2H), 3.59 – 3.52 (m, 8H), 2.83 (d, *J* = 6.0 Hz, 4H), 2.80 (t, *J* = 6.3 Hz, 4H), 2.03 – 1.96 (m, 8H).

^{13}C NMR (126 MHz, CD_3CN) δ 152.93, 151.65, 131.92, 127.41, 125.33, 114.17, 110.95, 107.38, 52.25, 51.73, 27.92, 21.11, 20.33, 20.01.

HRMS: $[M]^+$: calcd for $[C_{26}H_{26}N_3O]^+$: 396.2071, found: 396.2078.

Bis(julolidinyl)-9H-xanthene-9-one (3) – via hydrolysis of 2 [4]

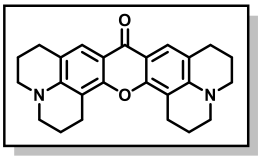

Compound **2** (0.835 g) was added to a 100 ml round-bottom flask and dissolved in 70 ml of 10% aqueous NaOH, then stirred at 95 °C for 20 min. After completion of the hydrolysis, the mixture was cooled to room temperature and extracted three times with DCM, followed by washing with brine. The organic phase was dried over $MgSO_4$ and concentrated. Purification was carried out by flash chromatography (eluent: DCM/MeOH 20:1) to yield a yellow crystalline solid.

Yield: 354 mg (47%)

^{1}H NMR (500 MHz, $CDCl_3$) δ 7.71 (s, 2H), 3.32 – 3.20 (m, 8H), 2.93 (t, *J* = 6.5 Hz, 4H), 2.82 (t, *J* = 6.2 Hz, 4H), 2.03 (p, *J* = 6.2 Hz, 4H), 1.97 (p, *J* = 6.2 Hz, 4H).

^{13}C NMR (126 MHz, $CDCl_3$) δ 175.46, 153.46, 147.13, 123.52, 118.11, 111.23, 105.73, 50.26, 49.75, 27.66, 21.93, 21.15, 20.79.

HRMS: $[M+H]^+$: calcd for $[C_{25}H_{27}N_2O_3]^+$: 387.2067, found: 387.2063.

Bis(julolidinyl)-9H-xanthene-9-one (3) via chemical redox cycling [5]

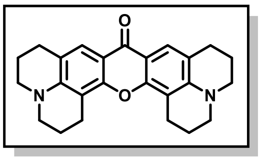

To a 50 mL round-bottomed flask, mesylate salt **1** (1.29 g, 2.76 mmol, 1.00 equiv.) and sodium phosphate tribasic dodecahydrate (2.10 g, 5.52 mmol, 2.00 equiv.) was added and dissolved in 17 ml *N*-methyl-2-pyrrolidone (NMP). Then water (0.56 ml, 31.46 mmol, 11.4 equiv.) was added and the flask was equipped with a temperature probe. The reaction mixture was stirred for 2 h at 110 °C then allowed to cool room temperature. During the first 2 h, the starting material is converted to a 1:1 mixture of product and a non-fluorescent by-product, giving a brown solution. Elemental iodine (0.69 g, 2.76 mmol, 1.00 equiv.), sodium phosphate (1.05 g, 2.76 mmol, 1.00 equiv.) and an additional portion of water (0.22 ml, 12.56 mmol, 4.55 equiv.) were added to the flask and the mixture was stirred overnight at 110 °C. Introduction of iodine to the solution results in a color change to a purple solution. After cooling to 23 ºC, the crude reaction mixture was filtered through a pad of celite, washed with DCM and methanol (20 ml) and concentrated on a rotary evaporator the bulk of the solvent removal. NMP is removed by vacuum distillation at 70 ºC with constant stirring until the pressure is gradually lowered (250 - 50 mmHg). The pressure was subsequently reduced to 1 mmHg and the mixture was stirred and further heated to 85 ºC. The majority of the NMP is removed after 1.5 h and the distillation is halted, affording a dark purple oil. Purification was carried out by flash chromatography (eluent: DCM/MeOH 20:1) to yield a yellow crystalline solid.

Yield: 811 mg (63%, average of 4 preparations). Characterization data is identical as above.

Bisjulolidinyl-9-acylpyronine 4

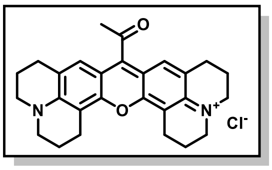

Ethyl vinyl ether (570 μl, 6.0 mmol, 7.5 equiv.) and 7.0 ml of anhydrous THF were placed into a previously dried flask under inert atmosphere. The reaction mixture was cooled to -78 °C in a dry ice-acetone bath. A solution of ^{t}BuLi in pentane (2.35 ml, 1.7 M, 4.0 mmol, 5 equiv.) was added dropwise, and the mixture was stirred for 20 min. The mixture was then warmed in an ice-water bath until decolorization occurred (approx. 5 min), followed by re-cooling to -78 °C. Xanthone **3** (309 mg, 0.8 mmol, 1 equiv.) was dissolved in 10.5 ml of anhydrous THF under inert atmosphere and added to the re-cooled mixture. Stirring continued for ~20 min at -78 °C and then for an additional 1 h at room temperature. Subsequently, MeOH/NH_4Cl solution was added carefully until gas evolution ceased. The mixture was extracted three times with DCM, washed with brine, and dried over Na_2SO_4. After concentration, the residue was dissolved in 10 ml of methanol and 1.0 ml of conc. HCl and stirred for 24 h. The mixture was extracted five times with DCM and washed with water, dried over Na_2SO_4 and evaporated to dryness, yielding a dark purple solid in pure form. If necessary, the product can be purified by reverse-phase (C18) flash chromatography (95:5 water:MeCN + 0.1% TFA to MeCN + 0.1% TFA).

Yield: 315.0 mg (88%)

^{1}H NMR (500 MHz, CD_3CN) δ 7.04 (s, 2H), 3.53 – 3.47 (m, 8H), 2.95 (t, J = 6.4 Hz, 4H), 2.80 (t, J = 6.1 Hz, 4H), 2.67 (s, 3H), 2.01 (p, J = 6.3 Hz, 4H), 1.96 (p, J = 6.3 Hz, 4H).

^{13}C NMR (126 MHz, CD_3CN) δ 203.69, 153.04, 152.53, 152.16, 125.89, 125.18, 109.02, 106.94, 51.78, 51.27, 33.38, 28.04, 21.27, 20.47, 20.24.

HRMS: $[M]^+$: calcd for $[C_{27}H_{29}N_2O_2]^+$: 413.2223, found: 413.2214.

Bisjulolidinyl-xanthene alcohol 5

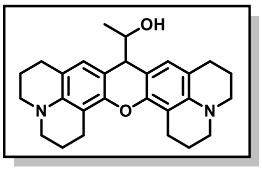

Ketone **4** (314 mg, 0.7 mmol, 1 equiv.) was dissolved in 25 ml EtOH, cooled in an ice water bath then sodium borohydride (265 mg, 7.0 mmol, 10 equiv.) was added and the reaction mixture was stirred at room temperature for 1 hour. Upon completion of the double reduction, water (25 ml) was carefully added, and the mixture was evaporated to half volume, then it was extracted three times with DCM (20 ml), then the combined organic phase evaporated to dryness, then redissolved with EtOH and evaporated to dryness in two times yielding a colorless/slightly pink amorphous solid that required no further purification.

Yield: 291 mg (99%)

^{1}H NMR (500 MHz, CD_3CN) δ 6.66 (s, 1H), 6.60 (s, 1H), 3.70 – 3.62 (m, 1H), 3.56 (d, *J* = 4.7 Hz, 1H), 3.13 – 3.04 (m, 8H), 2.78 (t, *J* = 6.3 Hz, 4H), 2.72 – 2.67 (m, 4H), 1.98 – 1.91 (m, 8H), 0.81 (d, *J* = 6.3 Hz, 3H).

^{13}C NMR (126 MHz, CD_3CN) δ 150.15, 149.91, 143.51, 127.94, 127.58, 126.90, 117.07, 111.63, 111.14, 109.67, 109.39, 74.11, 50.86, 50.84, 50.37, 50.35, 46.57, 27.98, 27.93, 23.28, 23.25, 22.63, 22.60, 22.09, 22.06, 19.13. *Note: some signals are duplicated.*

HRMS: $[M+H]^+$: calcd for $[C_{27}H_{33}N_2O_2]^+$: 417.2536, found: 417.2544.

Carbamate 6

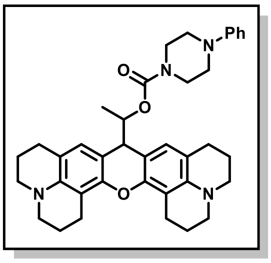

Activation with dipyridyl carbonate: Compound **5** (39.3 mg, 94.3 μmol) was dissolved in 3.5 ml of DCM (stabilized with amylene), then di-2-pyridyl carbonate (DPC,[6] 24.5 mg, 113 μmol, 1.2 equiv.) and triethylamine (19.7 μl, 142 μmol, 1.5 equiv.) were added, and the reaction mixture was stirred at room temperature for 24 h. Upon completion of the activation, saturated aqueous $NaHCO_3$ solution was added, and the mixture was extracted three times with DCM. The organic phase was dried over $MgSO_4$ and concentrated.

Carbamate bond formation: the crude mixed carbonate was dissolved in 4 ml DCM, then *N*-phenylpiperazine (21.6 μl, 141 μmol, 1.5 equiv.) and TEA (26.3 μl, 189 μmol, 2 equiv.) were added. The reaction mixture was stirred for 24 h at room temperature, after which 10% aqueous citric acid was added, followed by three extractions with DCM. The organic layer was dried over $MgSO_4$ and concentrated. The product was purified by reverse phase (C18) flash chromatography (95:5 water:MeCN + 0.1% TFA to MeCN + 0.1% TFA), extracted with DCM, dried over $MgSO_4$, and concentrated yielding an amorphous, slightly purple solid.

Note that in our first report,[2] we used normal phase chromatography which was detrimental to the yields for the carbamate formation (due to unwanted oxidation on the column). Reverse phase chromatography is better suited for these compounds.

Yield: 17 mg (30%)

^{1}H NMR (500 MHz, $CDCl_3$) δ 7.29 (t, *J* = 7.8 Hz, 2H), 6.95 (d, *J* = 8.1 Hz, 2H), 6.90 (t, *J* = 7.3 Hz, 1H), 6.71 (s, 1H), 6.57 (s, 1H), 4.94 – 4.89 (m, 1H), 3.95 (d, *J* = 3.7 Hz, 1H), 3.72 – 3.58 (m, 4H), 3.20 – 3.11 (m, 4H), 3.13 – 3.03 (m, 8H), 2.90 – 2.76 (m, 4H), 2.75 – 2.68 (m, 4H), 2.06 – 1.92 (m, 8H), 0.96 (d, *J* = 6.4 Hz, 3H).

See Characterization Data for HPLC-UV-MS analysis.

HRMS: $[M+H]^+$: calcd for $[C_{38}H_{45}N_4O_3]^+$: 605.3486, found: 605.3458.

X590-PP

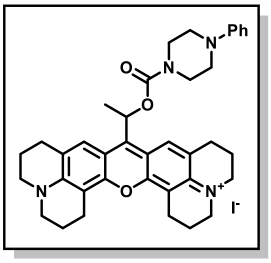

Rearomatization: Compound **6** (14 mg, 23 μmol) was dissolved in 1 ml of DCM. In the dark (foil-wrapped), *p*-chloranil (7.4 mg, 30 μmol, 1.3 equiv.) was added. The reaction mixture was stirred for 30 min, and LC–MS analysis confirmed complete oxidation/rearomatization. The product was purified directly by flash chromatography (eluent: DCM/MeOH 9:1) and concentrated.

Ion exchange: In case of **X590-PP** (iodide), anion exchange was performed using hydroiodic acid (57%). Hydroiodic acid (1.4 μl, 7.5 M, 1.0 equiv.) was measured and added to the reaction mixture, and the product was immediately purified by flash chromatography (eluent: DCM/MeOH 9:1). Based on NMR and LC–MS analysis, the product was obtained pure as a pink crystalline solid.

Yield: 12 mg (71%)

^{1}H NMR (500 MHz, CD_3CN) δ 7.84 (s, 2H), 7.29 – 7.21 (m, 2H), 6.94 (d, *J* = 7.9 Hz, 2H), 6.85 (t, *J* = 7.3 Hz, 1H), 6.49 (q, *J* = 7.0 Hz, 1H), 3.51 – 3.46 (m, 8H), 3.85 – 2.84 (m, 8H), 2.95 (t, *J* = 6.4 Hz, 4H), 2.91 – 2.83 (m, 4H), 2.05 – 1.96 (m, 8H), 1.81 (d, *J* = 7.0 Hz, 3H).

^{13}C NMR (75 MHz, CD_3CN) δ 155.16, 154.63, 152.98, 152.28, 151.91, 130.07, 125.12, 124.93, 120.95, 117.39, 111.12, 106.33, 70.11, 51.53, 50.97, 49.97, 44.94, 44.51, 28.45, 21.49, 20.55, 20.38.

See Characterization Data for HPLC-UV-MS analysis.

HRMS: $[M]^+$: calcd for $[C_{38}H_{43}N_4O_3]^+$: 603.3329, found: 603.3308.

Bisjulolidinyl-9-cyanoxanthene 7

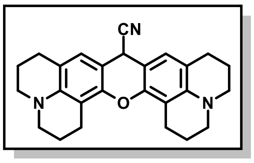

To a screw-capped vial, pyronine **1** (933 mg, 2.0 mmol, 1 equiv.) was added and dissolved in water (8 mL). To this vigorously stirred solution was added a solution of potassium cyanide (600 mg, 9.2 mmol, 4.6 equiv.) in water (6 mL). The vial was heated to 70 °C in an oil bath and stirred for 30 min. The hot solution was then filtered and the precipitate washed with water to yield a lilac powder of high purity based on ^1H NMR, which was used directly in the next step.

Yield: 748 mg (94%)

^1H NMR (300 MHz, $CDCl_3$) δ 6.83 (s, 2H), 5.05 (s, 1H), 3.17 – 3.09 (m, 8H), 2.81 (t, J = 6.6 Hz, 4H), 2.76 – 2.67 (m, 4H), 2.03 – 1.94 (m, 8H).

^{13}C NMR (126 MHz $CDCl_3$) δ 147.02, 143.85, 125.47, 120.75, 117.12, 109.05, 101.74, 50.07, 49.57, 29.61, 27.29, 22.21, 21.56, 21.24.

HRMS: $[M+H]^+$: calcd for $[C_{26}H_{28}N_3O]^+$: 398.2226, found: 398.2229.

Bisjulolidinyl-9-methoxycarbonylxanthene 8

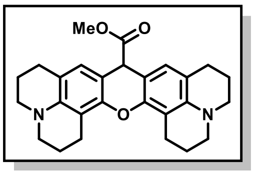

Nitrile **7** (398 mg, 1.0 mmol) was added to a mixture of sulfuric acid (7.5 ml) and methanol (7.5 ml), and the mixture was heated at reflux under nitrogen for 2.5 h. After cooling to room temperature, saturated $NaCO_3$ solution was carefully added, and the pH was set to ~9. The resulting mixture was extracted three times with EtOAc and the organic extracts were dried over Na_2SO_4. After filtration, the solvent was removed to give the crude methyl ester. Purification was carried out by flash chromatography (eluent: DCM/MeOH 0 to 10% gradient) to yield a colorless solid.

Yield: 152 mg (43%)

^1H NMR (500 MHz, $CDCl_3$) δ 6.65 (s, 2H), 4.71 (s, 1H), 3.63 (s, 3H), 3.18 – 3.04 (m, 8H), 2.92 – 2.77 (m, 4H), 2.71 (t, J = 5.9 Hz, 4H), 2.07 – 1.90 (m, 8H).

^{13}C NMR (126 MHz, $CDCl_3$) δ 174.42, 147.69, 143.34, 125.87, 116.49, 109.17, 106.23, 52.48, 50.26, 49.78, 44.59, 27.34, 22.47, 21.86, 21.42.

HRMS: $[M+H]^+$: calcd for $[C_{27}H_{31}N_2O_3]^+$: 431.2329, found: 431.2338.

Bisjulolidinyl-9-hydroxymethylxanthene 9

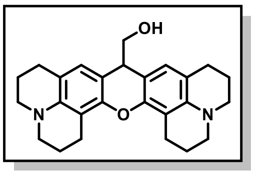

Bisjulolidinyl-9-methoxycarbonylxanthene **8** (99 mg, 0.23 mmol, 1.0 equiv.) and 400 μl of anhydrous THF were placed into a previously dried flask under inert atmosphere. To this vigorously stirred solution was added a solution of $LiAlH_4$ in THF (460 μl, 0.92 mmol, 4.0 equiv.) and the mixture was stirred for 60 min. Upon completion of the reduction, methanol (1 ml) was carefully added, and the mixture was evaporated to half volume, then it was extracted with EtOAc and cc. NaCl (10-10 ml), then the inorganic phase was washed with EtOAc (10 ml), dried over Na_2SO_4 and after filtration evaporated to dryness. The crude product required no further purification.

Yield: 89 mg (97%)

^1H NMR (300 MHz, CD_3CN) δ 6.66 (s, 2H), 3.64 (t, J = 5.7 Hz, 1H), 3.45 (t, J = 5.2 Hz, 2H), 3.12 – 3.04 (m, 8H), 2.80 (t, J = 6.7 Hz, 4H), 2.71 (t, J = 6.6 Hz, 4H), 2.01 – 1.88 (m, 8H).

^{13}C NMR (126 MHz, $CDCl_3$) δ 148.77, 142.87, 125.98, 116.48, 110.01, 109.37, 69.31, 50.34, 49.86, 41.23, 27.39, 22.56, 21.94, 21.46.

HRMS: $[M+H]^+$: calcd for $[C_{26}H_{31}N_2O_2]^+$: 403.2381, found: 403.2362.

Carbamate 10

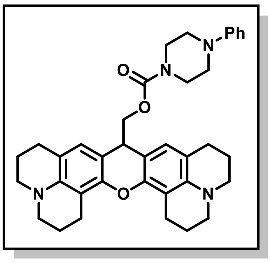

Activation with disuccinimidyl carbonate: Compound **9** (89 mg, 230 µmol, 1.0 equiv.) was dissolved in 6 ml of DCM (stabilized with amylene), then disuccinimidyl carbonate (DSC, 277 mg, 1.08 mmol, 4.6 equiv.) and triethylamine (226 µl, 1.62 mmol, 7.0 equiv.) were added, and the reaction mixture was stirred at room temperature for 24 h. Upon completion of the activation, saturated aqueous $NaHCO_3$ solution was added, and the mixture was extracted three times with DCM. The organic phase was rapidly dried over $MgSO_4$ and concentrated. The crude product required no further purification.

Carbamate bond formation: the crude mixed carbonate was dissolved in 6 ml DCM, then *N*-phenylpiperazine (42.2 µl,276 µmol, 1.2 equiv.) and TEA (188 µl, 1.03 mmol, 4.5 equiv.) were added. The reaction mixture was stirred for 24 h at room temperature, after it was evaporated to dryness The crude product was used without further purification. If necessary, the product can be purified by reverse-phase (C18) flash chromatography (95:5 water:MeCN + 0.1% TFA to MeCN + 0.1% TFA).

^1H NMR (500 MHz, CD_3CN, purified product) δ 7.25 (t, J = 7.9 Hz, 2H), 6.93 (d, J = 8.1 Hz, 2H), 6.84 (t, J = 7.2 Hz, 1H), 6.64 (s, 2H), 3.97 (d, J = 5.6 Hz, 2H), 3.87 (t, J = 5.4 Hz, 1H), 3.48 – 3.43 (m, 4H), 3.09 – 3.03 (m, 8H), 3.04 – 3.01 (m, 4H), 2.78 (t, J = 6.6 Hz, 4H), 2.67 (t, J = 6.4 Hz, 4H), 1.97 – 1.86 (m, 8H).

See Characterization Data for HPLC-UV-MS analysis.

HRMS: $[M+H]^+$: calcd for $[C_{37}H_{43}N_4O_3]^+$: 591.3329, found: 591.3338.

X600H-PP

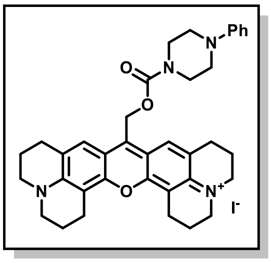

Crude product **10** (0.23 mmol, 1.0 equiv.) was dissolved in 1 ml of DCM. In the dark (foil-wrapped), iodine (52.3 mg, 0.20 mmol, 0.9 equiv.) was added. The reaction mixture was stirred for 1 min, and LC–MS analysis confirmed complete oxidation/rearomatization. The product was purified directly by flash chromatography (eluent: DCM/MeOH 9:1) and concentrated.

Yield: 76 mg (46%)

^1H NMR (500 MHz, CD_3CN) δ 7.63 (s, 2H), 7.21 (t, J = 7.9 Hz, 2H), 6.88 (d, J = 8.2 Hz, 2H), 6.81 (t, J = 7.3 Hz, 1H), 5.54 (s, 2H), 3.52 – 3.44 (m, 12H), 3.06 – 2.97 (m, 4H), 2.90 (t, J = 6.4 Hz, 4H), 2.84 (t, J = 6.0 Hz, 4H), 2.03 – 1.95 (m, 8H).

^{13}C NMR (126 MHz, CD_3CN) δ 155.18, 152.99, 152.27, 152.13, 147.84, 130.04, 125.52, 125.16, 120.87, 117.32, 113.20, 106.32, 58.75, 51.66, 51.13, 49.82, 44.84, 28.32, 21.45, 20.50, 20.35.

See Characterization Data for HPLC-UV-MS analysis.

HRMS: $[M+H]^+$: calcd for $[C_{37}H_{41}N_4O_3]^+$: 589.3173, found: 589.3171.

Bisjulolidinyl-9-carboxypyronine S5

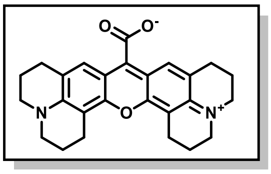

1.00 g (5.28 mmol) of 3-hydroxyjulolidine **S2** was weighed into a 50 mL round-bottom flask and dissolved it in 10 mL of methanesulfonic acid, 539 mg (2.64 mmol, 0,5 equiv.) of ethyl glyoxalate was added, and the mixture was stirred for 1 hour in an oil bath at 80 °C. Then, the mixture was cooled to room temperature, and the pH was adjusted to neutral with 10% NaOH solution, which was then extracted three times with DCM and washed with water. The combined organic phases were dried over $MgSO_4$. The crude product was dissolved in a mixture of 15 mL MeOH and 15 mL of 10% NaOH, then stirred for 1 hour at room temperature. After completion pH was adjusted to neutral with 2 M HCl, followed by extraction with DCM. It

was purified by column chromatography (eluent: DCM:MeOH, 95:5, + 0.1% TFA) to afford **S5** as a pure purple solid.

Yield: 283 mg (26%)

^{1}H NMR (500 MHz, DMSO-d_6) δ 7.28 (s, 2H), 3.52 – 3.42 (m, 8H), 2.92 (t, J = 6.1 Hz, 4H), 2.84 – 2.73 (m, 4H), 2.00 – 1.94 (m, 4H), 1.94 – 1.88 (m, 4H).

See Characterization Data for HPLC-UV-MS analysis.

HRMS: $[M+H]^+$: calcd for $[C_{26}H_{27}N_2O]^+$: 415.2016, found: 415.2025.

Bisjulolidinyl-9-hydroxymethylxanthene 9 (from the reduction of S5)

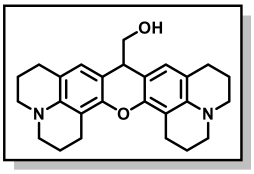

Into a dried 10 mL round-bottom flask flushed with N_2, 113 mg (234 μmol) of compound **S7** was weighed. 4.0 mL of anhydrous THF and 0.47 mL of a 2 M $LiAlH_4$ (935 μmol, 4 equiv.) solution in THF was added. The reaction mixture was stirred under an inert atmosphere at room temperature for 1.5 hours, then placed in an ice bath. Methanol was added until gas production stopped. A saturated potassium–sodium tartrate solution was added to the reaction mixture, which was then extracted four times with DCM and washed with water. The combined organic phases were dried over $MgSO_4$, filtered through Celite, and concentrated to obtain a pale purple solid.

Yield: 88 mg (93%). Characterization data is identical as above.

N-(9-cyano-6-(diethylamino)-3H-xanthen-3-ylidene)-N-ethylethanaminium (S8) [7]

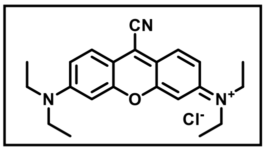

226 mg (630 μmol) of compound **S7** was weighed into a 50 mL two-neck round-bottom flask, then dissolved it in 12 mL of acetonitrile and 6.0 mL of water. A dropping funnel and a gas-absorption trap (loaded with 10% NaOH in water) were attached. 105 mg of KCN (1.61 mmol, 2.6 equiv.) was dissolved in 2.4 mL of water and then added it to the reaction mixture. After 15 minutes, a calculated amount of $FeCl_3$ solution was added dropwise (944 mg, 3.50 mmol, 5.0 equiv. $FeCl_3{\cdot}6H_2O$, 0.80 mL of 2 M hydrochloric acid, 2.4 mL of water). The mixture was stirred for an additional 25 minutes, then saturated $NaHCO_3$ solution was added, and the mixture was extracted three times with DCM. The combined organic phases were dried over $MgSO_4$ and concentrated. The product was obtained as green crystals and used without further purification.

Yield: 96 mg (40%)

3,6-Bis(diethylamino)-9H-xanthen-9-one (S9) [7]

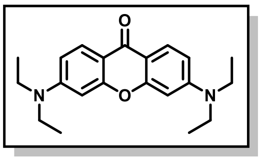

96 mg (249 μmol) of compound S9 was dissolved in 8.0 mL of 10% NaOH solution in a 10 mL round-bottom flask. The reaction mixture was stirred at 95 °C for 25 minutes. After completion of the hydrolysis, the mixture was cooled to room temperature and extracted three times with DCM, then washed with saturated aqueous brine. The combined organic layers were dried over $MgSO_4$ and concentrated. The product was purified by flash chromatography (eluent: DCM:MeOH, 9:1) to afford a yellow solid.

Yield: 20 mg (24%)

^{1}H NMR (500 MHz, $CDCl_3$) δ 8.09 (d, J = 9.0 Hz, 2H), 6.64 (dd, J = 9.0, 2.4 Hz, 2H), 6.44 (d, J = 2.4 Hz, 2H), 3.44 (q, J = 7.1 Hz, 8H), 1.23 (t, J = 7.1 Hz, 12H).

^{13}C NMR (126 MHz, $CDCl_3$) δ 174.91, 158.61, 152.16, 127.93, 111.68, 108.69, 96.44, 44.83, 12.69.

HRMS: $[M+H]^+$: calcd for $[C_{21}H_{27}N_2O_2]^+$: 339.2067, found: 339.2060.

N-(9-acetyl-6-(diethylamino)-3H-xanthen-3-ylidene)-N-ethylethanaminium chloride (S10)

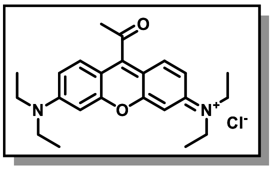

81 µL of ethyl vinyl ether (0,84 mmol, 7.5 equiv.) and 2.5 mL of absolute THF were placed into a dried 10 mL flask under an inert atmosphere. The reaction mixture was placed in a dry ice–acetone bath at −78 °C. Then 0.33 mL of a 1.7 M tert-BuLi solution in pentane (0.56 mmol) was added dropwise, and the mixture was stirred for 20 minutes. The reaction mixture was warmed in an ice–water bath until it became colorless (approx. 5 min), then cooled back to −78 °C. Separately, 38 mg of compound **S9** (112 µmol) was dissolved in 3.0 mL of absolute THF under an inert atmosphere and added to the recooled mixture. The reaction was stirred for approximately 20 minutes under these conditions, then for an additional 1 hour at room temperature. Subsequently, MeOH/NH_4Cl solution was cautiously added until gas evolution ceased. The reaction mixture was extracted three times with DCM, then washed with saturated aqueous brine and dried over $MgSO_4$. After evaporation, the residue was dissolved in 40 mL of methanol and 0.4 mL of concentrated hydrochloric acid and stirred for 24 hours. The reaction mixture was extracted five times with DCM and then washed with water. The product was purified by reverse-phase (C18) flash chromatography (95:5, water:MeCN + 0.1% TFA → MeCN + 0.1% TFA) to afford a pink solid.

Yield: 37 mg (82%)

^{1}H NMR (500 MHz, $CDCl_3$) δ 7.30 (d, *J* = 9.4 Hz, 2H), 7.00 (dd, *J* = 9.6, 1.6 Hz, 2H), 6.61 (d, *J* = 1.6 Hz, 2H), 3.51 (q, *J* = 7.0 Hz, 8H), 2.60 (s, 3H), 1.14 (t, *J* = 7.1 Hz, 12H).

^{13}C NMR (126 MHz, $CDCl_3$) δ 200.67, 157.59, 155.57, 152.98, 129.47, 115.23, 108.62, 96.69, 46.12, 32.80, 12.41.

HRMS: $[M]^+$: calcd for $[C_{23}H_{29}N_2O_2]^+$: 365.2223, found: 365.2235.

1-(3,6-bis(diethylamino)-9H-xanthen-9-yl)ethan-1-ol (S11)

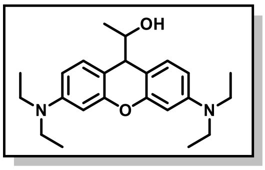

37 mg (92 µmol) of compound S10 was dissolved in 5.0 ml of ethanol, cooled in an ice water bath then 35 mg of $NaBH_4$ (92 µmol, 10 equiv.) was added. The reaction mixture was stirred at room temperature for 1 hour, then water was added and the mixture was extracted three times with DCM, followed by washing with saturated aqueous brine. The organic phase was dried over $MgSO_4$ and concentrated.

Yield: 33 mg (97%)

^{1}H NMR (500 MHz, $CDCl_3$) δ 7.08 (d, *J* = 8.0 Hz, 1H), 7.04 (d, *J* = 9.0 Hz, 1H), 6.45 – 6.40 (m, 4H), 3.79 (p, *J* = 6.0 Hz, 1H), 3.75 (d, *J* = 5.0 Hz, 1H), 3.35 (q, *J* = 6.6 Hz, 8H), 1.17 (t, *J* = 6.9 Hz, 12H), 1.01 (d, *J* = 6.0 Hz, 3H).

^{13}C NMR (126 MHz, $CDCl_3$) δ 154.31, 154.02, 148.19, 148.15, 129.97, 129.73, 110.06, 109.34, 107.37, 107.26, 99.61, 99.42, 73.60, 45.42, 44.57, 18.94, 12.77. *Note: aromatic signals are duplicated.*

HRMS: $[M+H]^+$: calcd for $[C_{23}H_{33}N_2O_2]^+$: 369.2536, found: 369.2547.

1-(3,6-bis(diethylamino)-9H-xanthen-9-yl)ethyl 4-phenylpiperazine-1-carboxylate (S13)

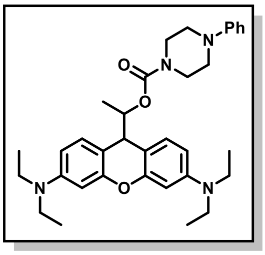

Activation with dipyridyl carbonate: Compound S11 was dissolved in 4 ml of DCM (stabilized with amylene), then 23 mg of DPC (108 µmol, 1.2 equiv.) and 19 µL of TEA (134 µmol, 1.5 equiv.) were added, and the mixture was stirred at room temperature for 24 hours. Upon completion of the activation aqueous $NaHCO_3$ solution was added, and the mixture was extracted three times with amylene-stabilized DCM. The organic phase was dried over $MgSO_4$ and concentrated.

Carbamate bond formation: The crude mixed carbonate was used without further purification: it was dissolved in 4 mL of amylene-stabilized DCM, then 20 µL of *N*-phenylpiperazine (134 µmol, 1.5 equiv.) and 25 µL of TEA (220 µmol, 2.0 equiv.) were added. The reaction mixture was stirred at room temperature for 24 hours, after which 10% citric acid was added, and the mixture was extracted three times with amylene-stabilized DCM. The organic phase was dried over $MgSO_4$ and concentrated. The product was

purified by reverse-phase flash chromatography (95:5, water:MeCN + 0.1% TFA → MeCN + 0.1% TFA), extracted with DCM, dried over $MgSO_4$, and concentrated to afford a pale purple solid.

Yield: 9.0 mg (18%)

^{1}H NMR (300 MHz, Acetonitrile-d_3) δ 7.32 – 7.21 (m, 2H), 7.08 (d, J = 8.5 Hz, 1H), 7.04 (d, J = 8.6 Hz, 1H), 6.95 (d, J = 8.1 Hz, 2H), 6.85 (t, J = 7.2 Hz, 1H), 6.46 (dd, J = 8.5, 2.4 Hz, 2H), 6.33 (dd, J = 7.0, 2.4 Hz, 2H), 4.88 – 4.77 (m, 1H), 3.97 (d, J = 3.8 Hz, 1H), 3.62 – 3.29 (m, 8H), 3.24 – 2.96 (m, 4H), 1.12 (q, J = 7.0 Hz, 12H), 0.95 (d, J = 6.4 Hz, 3H).

See Characterization Data for HPLC-UV-MS analysis.

HRMS: $[M+H]^+$: calcd for $[C_{34}H_{45}N_4O_3]^+$: 557.3486, found: 557.3487.

X565-PP

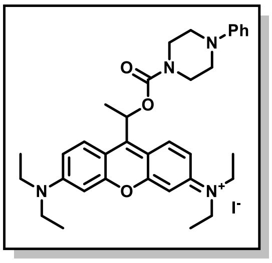

Rearomatization: 9.0 mg of compound **S13** (16 µmol) into a 10 mL round-bottom flask and dissolved it in 1.0 mL of DCM. In the dark (wrapped in aluminum foil), 5.2 mg of *p*-chloroanil (21 µmol, 1.3 equiv.) was added. The reaction mixture was stirred for 30 minutes, then purified by flash chromatography (eluent: DCM:MeOH, 9:1).

Ion Exchange: Counterion exchange was carried out using 7.5 M HI. The HI solution was extracted with tributyl phosphate, transferring I_2 into the organic phase. Then, 1.4 µL of 7.5 M HI solution (1.0 equiv.) was measured and added to the reaction mixture, and the product was immediately purified by flash chromatography (eluent: DCM:MeOH, 9:1) to obtain pure **X565-PP** as a purple solid.

Yield: 4.0 mg (11%)

^{1}H NMR (300 MHz, CD_3CN) δ 8.27 (d, J = 9.7 Hz, 2H), 7.25 (t, J = 7.9 Hz, 2H), 7.12 (dd, J = 9.7, 2.3 Hz, 2H), 6.94 (d, J = 8.1 Hz, 2H), 6.85 (t, J = 7.2 Hz, 1H), 6.76 (d, J = 2.4 Hz, 2H), 6.56 (q, J = 7.0 Hz, 1H), 3.63 (q, J = 7.1 Hz, 8H), 3.88 – 2.81 (m, 8H), 1.83 (d, J = 7.0 Hz, 3H), 1.26 (t, J = 7.1 Hz, 12H).

See Characterization Data for HPLC-UV-MS analysis.

HRMS: $[M]^+$: calcd for $[C_{34}H_{43}N_4O_3]^+$: 555.3329, found: 555.3341.

6-(diethylamino)-3-(diethyliminio)-3H-xanthene-9-carboxylate (S14)

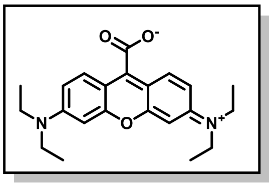

2.54 g of 3-diethylaminophenol (15.4 mmol) was weighed into a 50 mL round-bottom flask and dissolved it in 30 mL of propionic acid. 699 mg of glyoxylic acid monohydrate (7.59 mmol, 0.5 equiv.) was added, and the mixture was stirred at 110 °C for 24 hours. The reaction mixture was concentrated and then dried under high vacuum. The product was purified by column chromatography (eluent: DCM:MeOH, 95:5, + 0.1% TFA) to afford **S14** as a pure purple solid.

Yield: 883 mg (31%)

^{1}H NMR (500 MHz, DMSO-d_6) δ 7.70 (d, J = 9.4 Hz, 2H), 7.23 (d, J = 8.9 Hz, 2H), 6.91 (s, 2H), 3.66 (q, J = 6.3 Hz, 8H), 1.22 (t, J = 6.8 Hz, 12H).

^{13}C NMR (126 MHz, DMSO-d_6) δ 175.30, 165.13, 157.66, 155.29, 130.84, 114.86, 108.99, 96.21, 45.40, 12.40.

HRMS: $[M+H]^+$: calcd for $[C_{22}H_{27}N_2O_3]^+$: 367.2016, found: 367.2027.

1-(3,6-di(pyrrolidin-1-yl)-9H-xanthen-9-yl)ethyl 4-phenylpiperazine-1-carboxylate (S20)

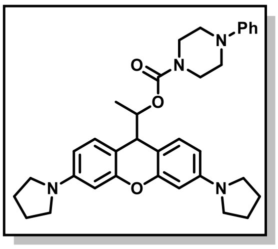

Activation with dipyridyl carbonate: Compound **S18** (72 mg, 198 mmol) was dissolved in 2.0 ml of DCM (stabilized with amylene), then di-2-pyridyl carbonate (51.2 mg, 237 mmol, 1.2 equiv.) and triethylamine (41 ml, 296 mmol, 1.5 equiv.) were added, and the reaction mixture was stirred at room temperature for 24 h. Upon completion of the activation, saturated aqueous $NaHCO_3$ solution was added, and the mixture was extracted three times with DCM. The organic phase was dried over $MgSO_4$ and concentrated.

Carbamate bond formation: the crude mixed carbonate was dissolved in 2 ml DCM, then *N*-phenylpiperazine (45 µl, 297 µmol, 1.5 equiv.) and TEA (55 µl, 396 µmol, 2 equiv.) were added. The reaction mixture was stirred for 24 h at room temperature, after which 10% aqueous citric acid was added, followed by three extractions with DCM. The organic layer was dried over $MgSO_4$ and concentrated. The product was purified by reverse phase (C18) flash chromatography (95:5 water:MeCN + 0.1% TFA to MeCN + 0.1% TFA), extracted with DCM, dried over $MgSO_4$, and concentrated yielding an amorphous, slightly purple solid.

Yield: 69 mg (63%)

^{1}H NMR (500 MHz, DMSO-d_6) δ 7.25 (t, J = 7.7 Hz, 2H), 7.09 (d, J = 8.3 Hz, 1H), 7.03 (d, J = 8.4 Hz, 1H), 6.99 (d, J = 8.1 Hz, 2H), 6.84 (t, J = 7.3 Hz, 1H), 6.37 – 6.31 (m, 2H), 6.22 (dd, J = 7.2, 2.3 Hz, 2H), 4.78 – 4.72 (m, 1H), 4.01 (d, J = 3.9 Hz, 1H), 4.01 – 3.44 (m, 8H), 3.26 – 3.19 (m, 8H), 1.99 – 1.92 (m, 8H), 0.87 (d, J = 6.5 Hz, 3H).

See Characterization Data for HPLC-UV-MS analysis.

HRMS: $[M+H]^+$: calcd for $[C_{34}H_{41}N_4O_3]^+$: 553.3173, found: 553.3185.

X570-PP

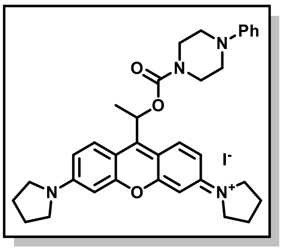

Rearomatization: Compound **S20** (69 mg, 120 µmol) was dissolved in 2 ml of DCM. In the dark (foil-wrapped), *p*-chloranil (37 mg, 150 µmol, 1.2 equiv.) was added. The reaction mixture was stirred for 30 min, and LC–MS analysis confirmed complete oxidation/rearomatization. The product was purified directly by flash chromatography (eluent: DCM/MeOH 9:1) and concentrated.

Ion exchange: In case of **X570-PP** (iodide), anion exchange was performed using hydroiodic acid (57%). Hydroiodic acid (17 µl, 7.5 M, 1.0 equiv.) was measured and added to the reaction mixture, and the product was immediately purified by flash chromatography (eluent: DCM/MeOH 9:1). Based on NMR and LC–MS analysis, the product was obtained pure as a pink crystalline solid.

Yield: 50 mg (59%)

^{1}H NMR (500 MHz, Acetonitrile-d_3) δ 8.27 (d, J = 9.6 Hz, 2H), 7.30 – 7.24 (m, 2H), 7.01 – 6.98 (m, 2H), 6.98 (d, J = 6.6 Hz, 2H), 6.88 (t, J = 7.3 Hz, 1H), 6.63 (d, J = 2.4 Hz, 2H), 6.59 (q, J = 7.0 Hz, 1H), 3.67 – 3.52 (m, 8H), 3.82 – 2.90 (m, 8H), 2.11 – 2.05 (m, 8H), 1.83 (d, J = 7.0 Hz, 3H).

See Characterization Data for HPLC-UV-MS analysis.

HRMS: $[M]^+$: calcd for $[C_{34}H_{39}N_4O_3]^+$: 551.3016, found: 551.3031.

3.3 Synthesis of the Prodrugs

X590-SN38 precursor (S21)

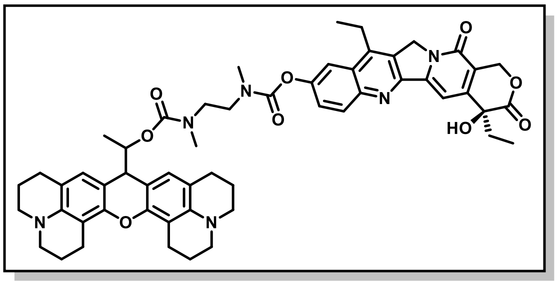

Activation with disuccinimidyl carbonate: Compound **5** (35 mg, 85 µmol) was dissolved in 2.0 ml of DCM (stabilized with amylene), then disuccinimidyl carbonate (65 mg, 260 µmol, 3 equiv.) and triethylamine (71 µl, 510 µmol, 6 equiv.) were added, and the reaction mixture was stirred at room temperature for 24 h. Upon completion of the activation, saturated aqueous $NaHCO_3$ solution was added, and the mixture was extracted three times with DCM. The organic phase was dried over $MgSO_4$ and concentrated.

Carbamate bond formation: the crude mixed carbonate was dissolved in 2 ml DCM, then **L-SN38** [2] (53 mg, 85 µmol, 1 equiv.) and TEA (36 µl, 260 µmol, 3 equiv.) were added. The reaction mixture was stirred for 24 h at room temperature, after which 10% aqueous citric acid was added, followed by three extractions with DCM. The organic layer was dried over $MgSO_4$ and concentrated. The product was purified by reverse phase (C18) flash chromatography (95:5 water:MeCN + 0.1% TFA to MeCN + 0.1% TFA), extracted with DCM, dried over $MgSO_4$, and concentrated yielding an amorphous, slightly purple solid.

Yield: 15 mg (19%)

The product was of sufficient quality according to the HPLC-UV-MS data (see Characterization Data) to proceed with the next steps immediately.

HRMS: $[M+2H]^{2+}$: calcd for $[C_{55}H_{62}N_6O_9]^{2+}$: 475.2284, found: 475.2268.

X590-SN38

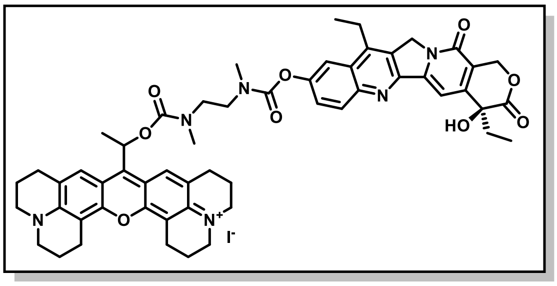

Oxidation: Compound **S21** (15 mg, 16 µmol) was dissolved in 1 ml of DCM. In the dark (foil-wrapped), *p*-chloranil (5.1 mg, 21 µmol, 1.3 equiv.) was added. The reaction mixture was stirred for 30 min, and LC–MS analysis confirmed complete oxidation/rearomatization. The product was purified directly by flash chromatography (eluent: DCM/MeOH 9:1) and concentrated.

Ion exchange: In case of **X590-SN38** (iodide), anion exchange was performed using hydroiodic acid (57%). Hydroiodic acid (2.1 µl, 7.5 M, 1.0 equiv.) was measured and added to the reaction mixture, and the product was immediately purified by flash chromatography (eluent: DCM/MeOH 9:1). Based on NMR and LC–MS analysis, the product was obtained pure as a pink crystalline solid.

Yield: 6.7 mg (45%)

^{1}H NMR (500 MHz, Acetonitrile-d_3) δ 7.94 – 7.86 (m, 1H), 7.85 – 7.69 (m, 2H), 7.37 (s, 1H), 7.31 – 7.20 (m, 1H), 7.09 – 7.00 (m, 1H), 6.77 – 6.45 (m, 1H), 5.62 – 5.53 (m, 1H), 5.39 – 5.29 (m, 1H), 5.24 – 5.07 (m, 2H), 4.42 – 4.27 (m, 2H), 4.00 – 3.91 (m, 1H), 3.53 – 3.19 (m, 12H), 3.19 – 3.06 (m, 2H), 2.95 – 2.75 (m, 7H), 2.75 – 2.59 (m, 2H), 2.49 – 2.24 (m, 3H), 1.99 – 1.95 (m, 4H), 1.87 – 1.77 (m, 4H), 1.75 (d, *J* = 7.1 Hz, 3H), 1.34 – 1.24 (m, 2H), 1.18 – 1.09 (m, 3H), 1.03 – 0.95 (m, 3H).

See Characterization Data for HPLC-UV-MS analysis.

HRMS: $[M+H]^{2+}$: calcd for $[C_{55}H_{60}N_6O_9]^{2+}$: 474.2216, found: 474.2193.

X590-MMAE precursor (S22)

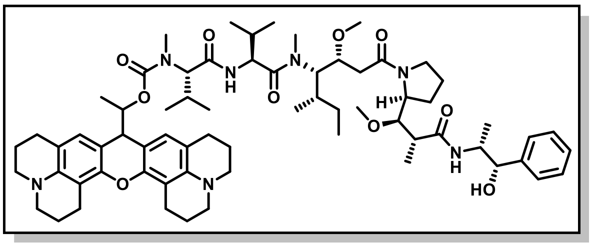

Activation with dipyridyl carbonate: Compound **5** (41.2 mg, 100 mmol) was dissolved in 2.0 ml of DCM (stabilized with amylene), then di-2-pyridyl carbonate (28.1 mg, 130 mmol, 1.3 equiv.) and triethylamine (20.9 ml, 150 mmol, 1.5 equiv.) were added, and the reaction mixture was stirred at room temperature for 24 h. Upon completion of the activation, saturated aqueous $NaHCO_3$ solution was added, and the mixture was extracted three times with DCM. The organic phase was dried over $MgSO_4$ and concentrated.

Carbamate bond formation: the crude mixed carbonate was dissolved in 3 ml DMF, then MMAE (35 mg, 50 mmol, 0.5 equiv.) and DIPEA (51 μl, 300 mmol, 3 equiv.) were added. The reaction mixture was stirred for 24 h at 40 °C, after which 10% aqueous citric acid was added, followed by three extractions with DCM. The organic layer was dried over $MgSO_4$ and concentrated. The product was purified by reverse phase (C18) flash chromatography (95:5 water:MeCN + 0.1% TFA to MeCN + 0.1% TFA), extracted with DCM, dried over $MgSO_4$, and concentrated yielding an amorphous, slightly purple solid.

Yield: 41 mg (72%)

The product was of sufficient quality according to the HPLC-UV-MS data (Characterization Data) to proceed with the next steps immediately.

HRMS: $[M+H]^+$: calcd for $[C_{67}H_{99}N_7O_{10}]^{2+}$: 580.8721, found: 580.8743.

X590-MMAE

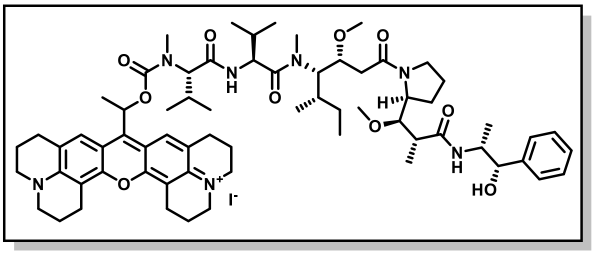

Oxidation: Compound **S22** (41 mg, 35 μmol) was dissolved in 1 ml of DCM. In the dark (foil-wrapped), *p*-chloranil (11 mg, 46 μmol, 1.3 equiv.) was added. The reaction mixture was stirred for 30 min, and LC–MS analysis confirmed complete oxidation/rearomatization. The product was purified directly by flash chromatography (eluent: DCM/MeOH 9:1) and concentrated.

Ion exchange: Hydroiodic acid (4.7 μl, 7.5 M, 1.0 equiv.) was measured and added to the reaction mixture, and the product was immediately purified by flash chromatography (eluent: DCM/MeOH 9:1). Based on NMR and LC–MS analysis, the product was obtained pure as a pink crystalline solid.

Yield: 36 mg (88%)

^{1}H NMR (500 MHz, Acetonitrile-d_3) δ 7.99 – 7.76 (m, 2H), 7.39 – 7.25 (m, 5H), 7.24 – 6.69 (m, 2H), 6.68 – 6.35 (m, 1H), 4.96 – 4.30 (m, 4H), 4.24 – 3.85 (m, 2H), 3.74 (q, J = 7.9, 6.2 Hz, 1H), 3.68 – 3.44 (m, 10H), 3.41 – 3.16 (m, 8H), 3.15 – 2.76 (m, 14H), 2.68 – 2.37 (m, 2H), 2.36 – 2.22 (m, 2H), 2.15 – 2.06 (m, 2H), 2.06 – 1.96 (m, 9H), 1.91 – 1.74 (m, 5H), 1.70 – 1.44 (m, 1H), 1.18 – 0.60 (m, 25H), 0.56 – 0.43 (m, 1H).

See Characterization Data for HPLC-UV-MS analysis.

HRMS: $[M+H]^{2+}$: calcd for $[C_{67}H_{97}N_7O_{10}]^{2+}$: 579.8653, found: 579.8626.

3.4 Synthesis of the Caged WY Compounds

Racemic WAY-161503 (**WY**) was synthesized as described in [8].

X590-WY precursor (S27)

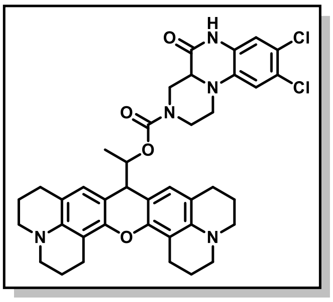

Activation with disuccinimidyl carbonate: Compound **5** (46.7 mg, 112 μmol) was dissolved in 2.0 ml of DCM (stabilized with amylene), then disuccinimidyl carbonate (86.2 mg, 336 μmol, 3 equiv.) and triethylamine (93.8 μl, 673 μmol, 6 equiv.) were added, and the reaction mixture was stirred at room temperature for 24 h. Upon completion of the activation, saturated aqueous $NaHCO_3$ solution was added, and the mixture was extracted three times with DCM. The organic phase was dried over $MgSO_4$ and concentrated.

Carbamate bond formation: the crude mixed carbonate was dissolved in 2 ml DCM, then **WAY-161503** (36.6 mg, 134 μmol, 1.2 equiv.) and TEA (46.8 μl, 336 μmol, 3 equiv.) were added. The reaction mixture was stirred for 24 h at room temperature, after which 10% aqueous citric acid was added, followed by three extractions with DCM. The organic layer was dried over $MgSO_4$ and concentrated. The product was purified by reverse phase (C18) flash chromatography (95:5 water:MeCN + 0.1% TFA to MeCN + 0.1% TFA), extracted with DCM, dried over $MgSO_4$, and concentrated yielding an amorphous, slightly purple solid.

Yield: 15 mg (19%)

The product was of sufficient quality according to the HPLC-UV-MS data (Characterization Data) to proceed with the next steps immediately.

HRMS: $[M+H]^+$: calcd for $[C_{39}H_{42}N_5O_4Cl_2]^+$: 714.2608, found: 714.2603.

X590-WY

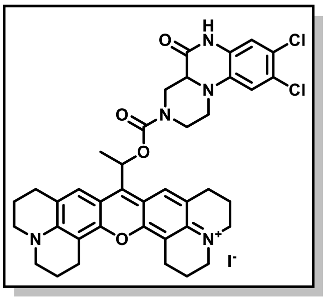

Oxidation: Compound **S27** (15 mg, 21 μmol) was dissolved in 1 ml of DCM. In the dark (foil-wrapped), *p*-chloranil (6.2 mg, 25 μmol, 1.2 equiv.) was added. The reaction mixture was stirred for 30 min, and LC–MS analysis confirmed complete oxidation/rearomatization. The product was purified directly by flash chromatography (eluent: DCM/MeOH 9:1) and concentrated.

Ion exchange: Hydroiodic acid (2.8 μl, 7.5 M, 1.0 equiv.) was measured and added to the reaction mixture, and the product was immediately purified by flash chromatography (eluent: DCM/MeOH 9:1). Based on NMR and LC–MS analysis, the product was obtained pure as a pink crystalline solid.

Yield: 13 mg (74%)

^1H NMR (500 MHz, CD_3CN) δ 8.94 – 8.72 (m, 1H), 7.91 – 7.74 (m, 2H), 7.08 – 6.80 (m, 2H), 6.55 (br s, 1H), 4.68 – 3.49 (m, 4H), 3.51 – 3.44 (m, 8H), 3.32 – 3.09 (m, 1H), 2.99 – 2.79 (m, 10H), 2.05 – 1.95 (m, 8H), 1.82 (d, *J* = 7.0 Hz, 3H).

See Characterization Data for HPLC-UV-MS analysis.

HRMS: $[M]^+$: calcd for $[C_{39}H_{40}N_5O_4Cl_2]^+$: 712.2451, found: 712.2452.

X600H-WY precursor (S27)

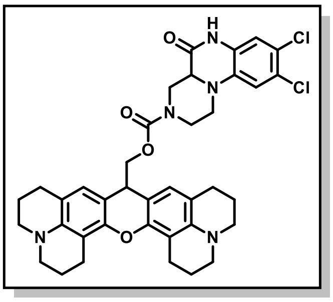

Activation with disuccinimidyl carbonate: Compound **5** (6 mg, 14.9 μmol) was dissolved in 1.0 ml of DCM (stabilized with amylene), then disuccinimidyl carbonate (11.5 mg, 44.7 μmol, 3 equiv.) and triethylamine (12.5 μl, 89.4 μmol, 6 equiv.) were added, and the reaction mixture was stirred at room temperature for 24 h. Upon completion of the activation, saturated aqueous $NaHCO_3$ solution was added, and the mixture was extracted three times with DCM. The organic phase was dried over $MgSO_4$ and concentrated.

Carbamate bond formation: the crude mixed carbonate was dissolved in 1.0 ml DCM, then **WAY-161503** (4.9 mg, 17.9 μmol, 1.2 equiv.) and TEA 6.2 μl, 44.7 μmol, 3 equiv.) were added.

The reaction mixture was stirred for 24 h at room temperature, after which 10% aqueous citric acid was added, followed by three extractions with DCM. The organic layer was dried over $MgSO_4$ and concentrated. The product was purified by reverse phase (C18) flash chromatography (95:5 water:MeCN + 0.1% TFA to MeCN + 0.1% TFA), extracted with DCM, dried over $MgSO_4$, and concentrated yielding an amorphous, slightly purple solid.

Yield: 8.6 mg (82%)

The product was of sufficient quality according to the HPLC-UV-MS data (see Characterization Data) to proceed with the next steps immediately.

HRMS: $[M+H]^+$: calcd for $[C_{38}H_{40}N_5O_4Cl_2]^+$: 700.2451, found: 700.2385.

X600H-WY

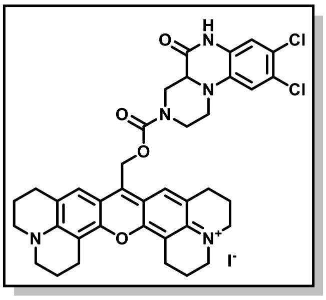

Rearomatization: Compound **10** (8.6 mg, 12.0 µmol, 1.0 equiv.) was dissolved in 1.0 ml of DCM. In the dark (foil-wrapped), then iodine (4.0 mg, 16 µmol, 1.3 equiv.) in DCM (0.5 ml) was added. The reaction mixture was stirred for 1 min, and LC–MS analysis confirmed complete oxidation/rearomatization. The product was purified directly by flash chromatography (eluent: DCM/MeOH 9:1) and concentrated.

Yield: 3.6 mg (35%)

^1H NMR (500 MHz, CD_3CN) δ 8.63 (s, 1H), 7.67 (s, 2H), 6.93 (s, 2H), 5.63 (s, 2H), 3.57 – 3.41 (m, 11H), 3.01 – 2.92 (m, 8H), 2.92 – 2.82 (m, 4H), 2.05 – 1.94 (m, 8H).

See Characterization Data for HPLC-UV-MS analysis.

HRMS: $[M]^+$: calcd for $[C_{38}H_{38}N_5O_4Cl_2]^+$: 698.2295, found: 698.2264.

4. Spectroscopic Properties of the Compounds

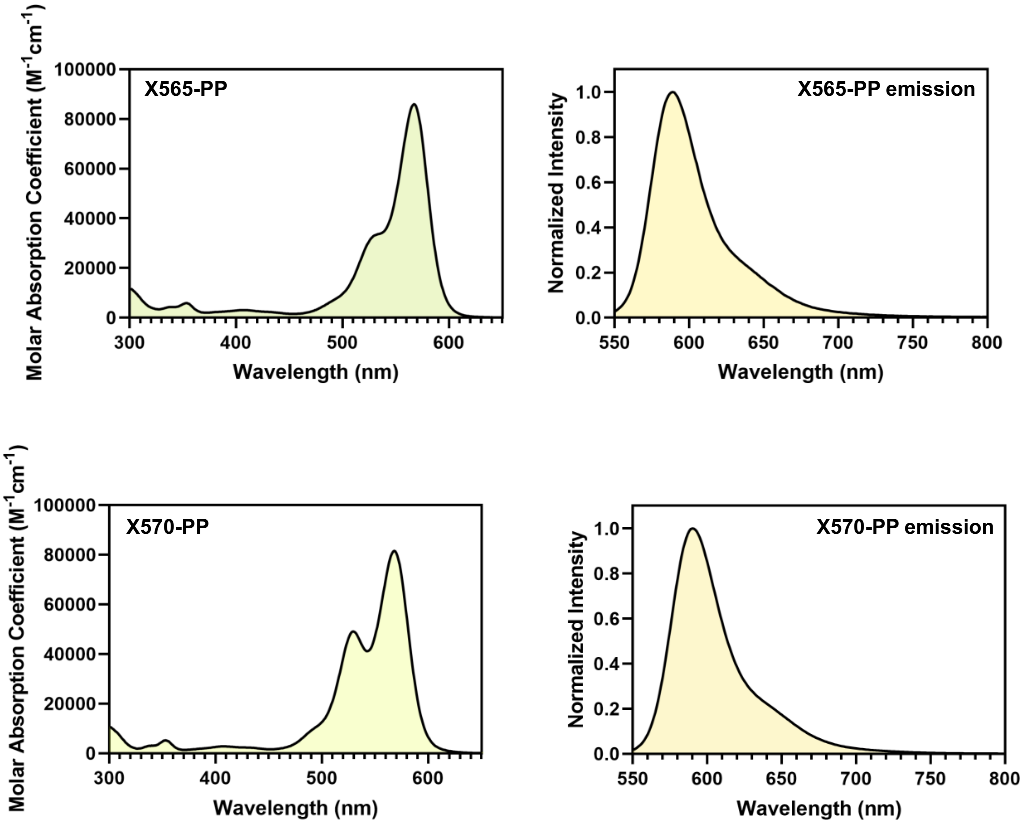

*Figure S6. Absorption and fluorescence spectra of the model compounds **X565-PP** and **X570-PP** in water*

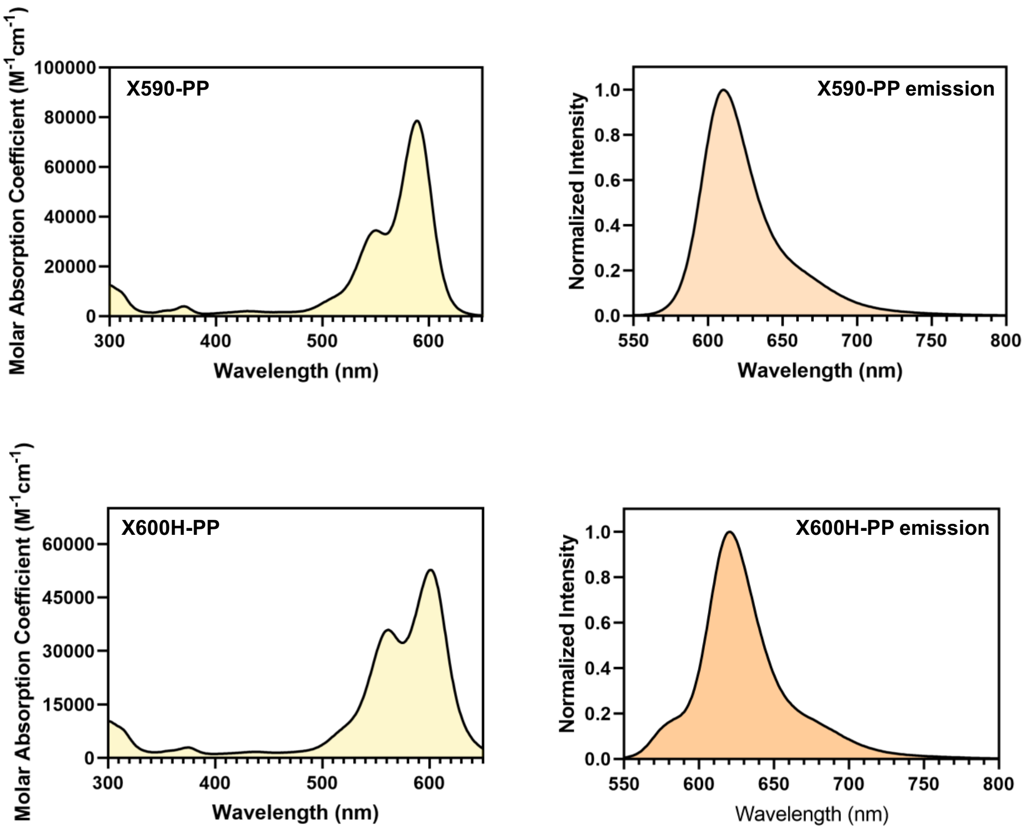

*Figure S7. Absorption and fluorescence spectra of the model compounds **X590-PP** and **X600H-PP** in water*

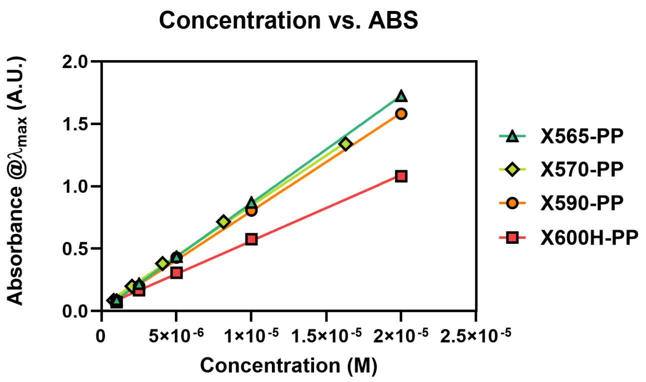

Figure S8. Concentration vs. absorbance of the model compounds in water

5. Photochemical Studies

5.1 Experimental Details for Photochemical Studies

The uncaging experiments were performed using custom-built LED panels with commercial light sources [2] with the following specifications per color: green: ~4W input power, λ_{max} = 549 nm half-width: 16 nm, output power: 147 mW; orange: 4W input power, λ_{max} = 605 nm half-width: 10 nm, output power: 270 mW and red: 10W input power, λ_{max} = 630 nm half-width: 10 nm, output power: 400 mW. For most experiments, the green LED was used at 50% power setting (80 mW). Sample solutions were prepared, each containing 1 ml of solvent (90% v/v water-MeCN or 100% methanol for the photochemical quantum yield determination; optical path length: 14 mm) and 0.1 mM concentration of the compounds. The samples were irradiated for a given time using continuous water cooling for the light source and carefully avoiding overheating. Then, the samples were transferred to the HPLC-UV-MS system, and the chromatograms were recorded. The 3D chromatograms were plotted at various wavelengths, dependent on the payload/photocage. Representative contour plots prepared by Python/matplotlib are also shown. The peaks corresponding to the payloads/starting materials (SMs) were integrated and compared to reference calibration sets. The possible photoproducts were identified by their corresponding m/z value and from the UV/VIS spectra from the DAD-equipped instrument. For stability determination, the HPLC sample solutions were kept in the dark and chromatograms were recorded multiple times. Output power was measured using a Thorlabs PM100D Optical Power Meter equipped with an S121C (400 nm – 1100 nm) sensor.

Deoxygenated uncaging experiments were carried out in 0.1 mM solution of **X590-PP** in 90% v/v water-MeCN. The 1 mM stock solutions of the caged compounds were diluted to 10 mL under N_2 to receive the above-mentioned composition and nominal concentration. The solution was then deoxygenated with the freeze-pump-thaw technique (5 times) and the flask was finally filled with N_2. The solution was distributed under N_2 to get eight LC-MS samples with the same concentration and volume. The samples were irradiated through different periods of times with orange light.

Uncaging experiments followed by UV/VIS absorption spectroscopy were performed using orange light at 25% power (λ_{max} = 605 nm half-width: 10 nm, output power: ~65 mW) in water (~5 μM concentration, < 1% MeCN). For the photochemical quantum yield determination, please refer to Section 5.5.

5.2 Uncaging Experiments Followed by HPLC-MS

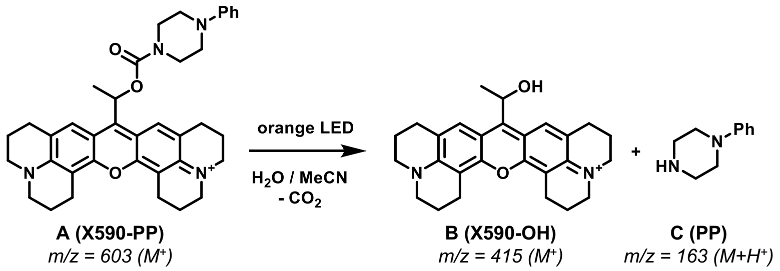

irradiation in H_2O/MeCN 9:1 with orange light:

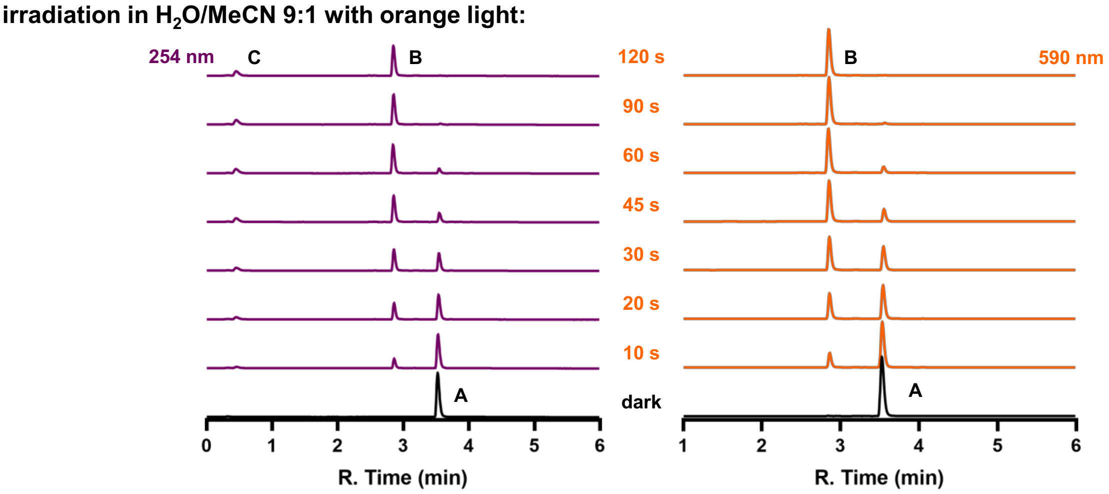

irradiation in H_2O/MeCN 9:1 with orange light (2D contour chromatograms):

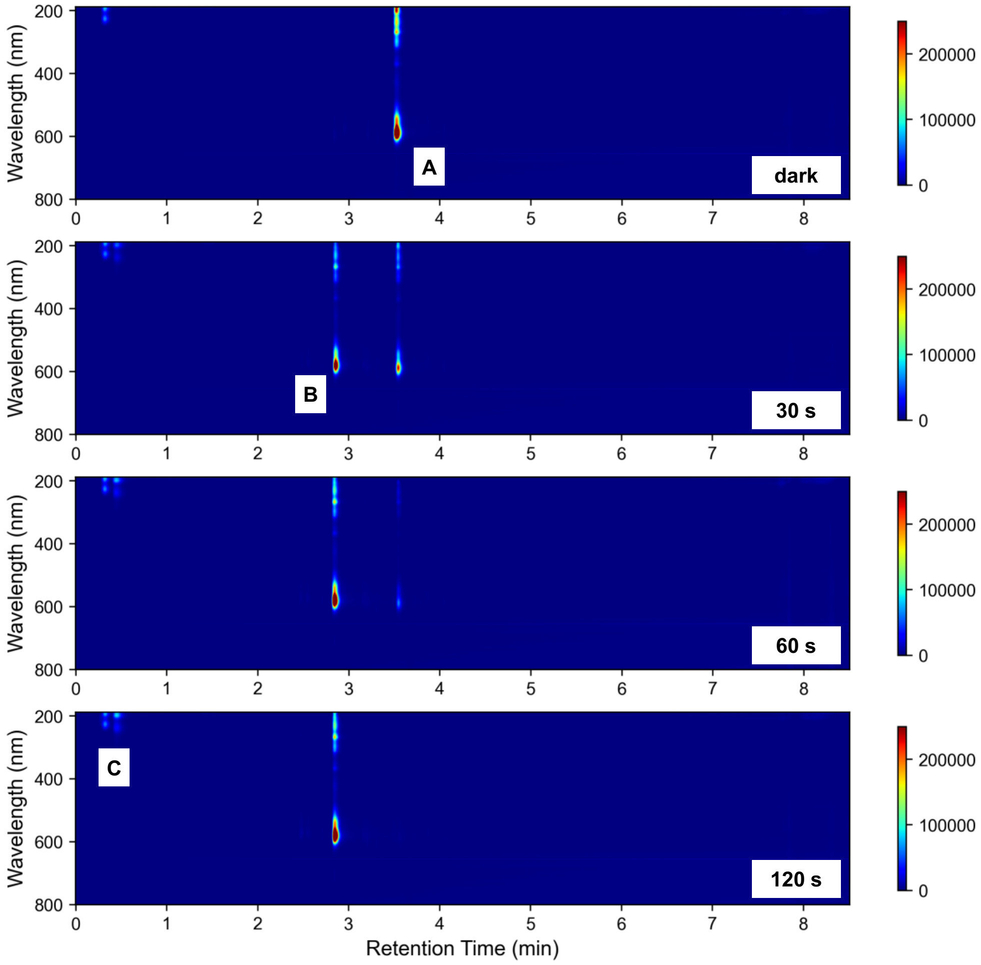

Figure S9. Chromatograms of the photolysis of ***X590-PP (iodide)*** *as followed by HPLC-UV/Vis-MS instrument.*

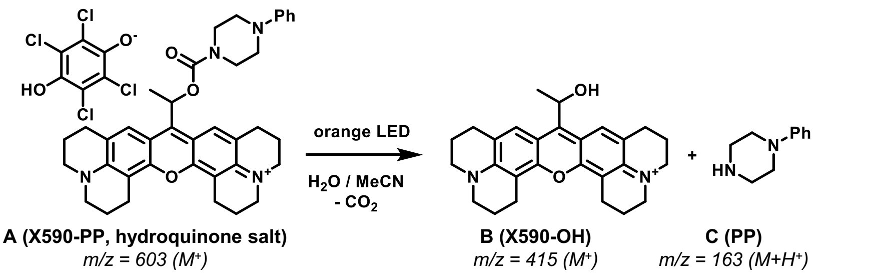

irradiation in H_2O/MeCN 9:1 with orange light:

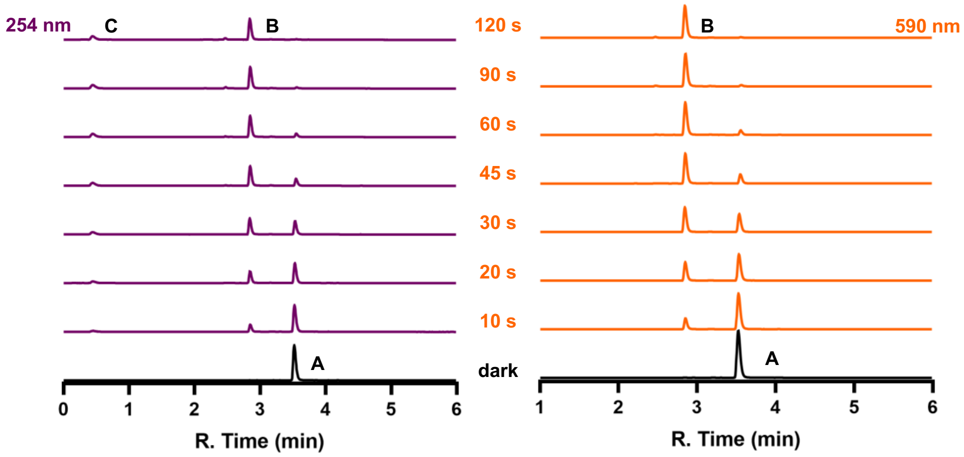

irradiation in H_2O/MeCN 9:1 with orange light (2D contour chromatograms):

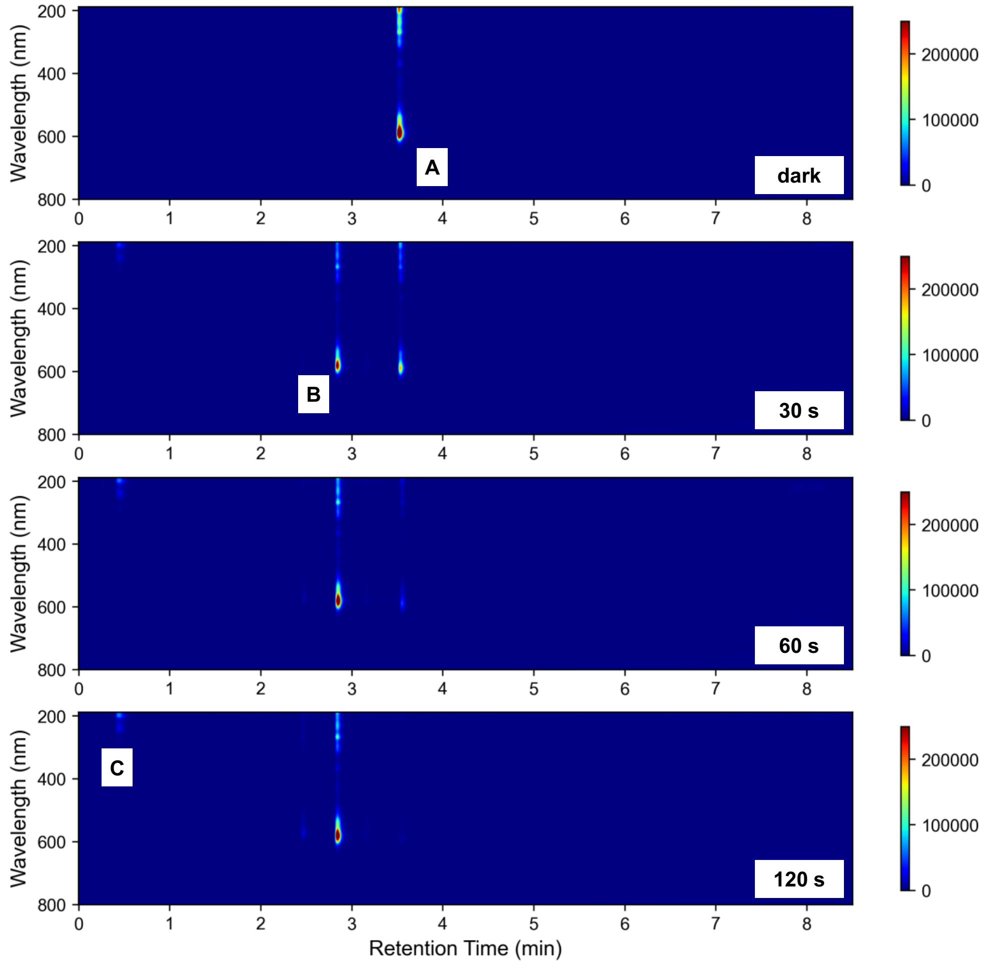

*Figure S10. Chromatograms of the photolysis of **X590-PP** (without ion exchange) as followed by HPLC-UV/Vis-MS instrument.*

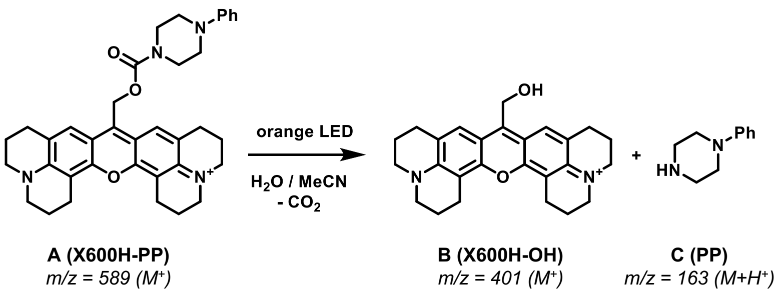

irradiation in H_2O/MeCN 9:1 with orange light:

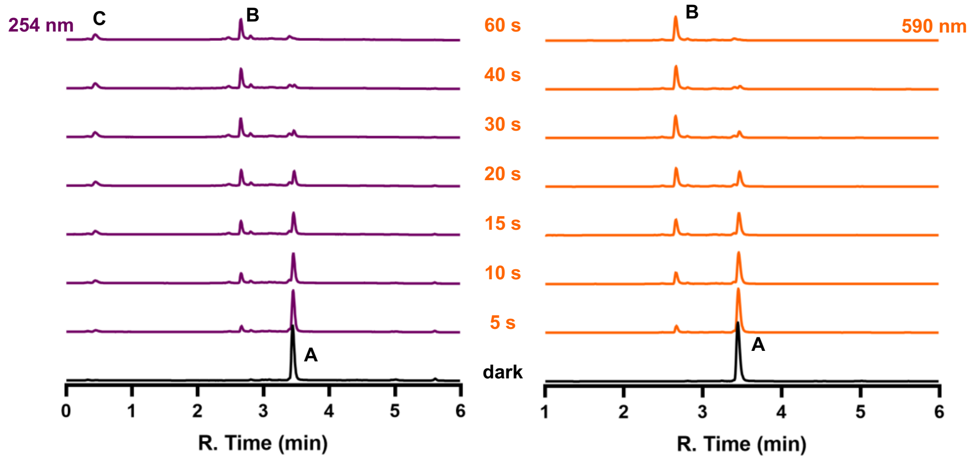

irradiation in H_2O/MeCN 9:1 with orange light (2D contour chromatograms):

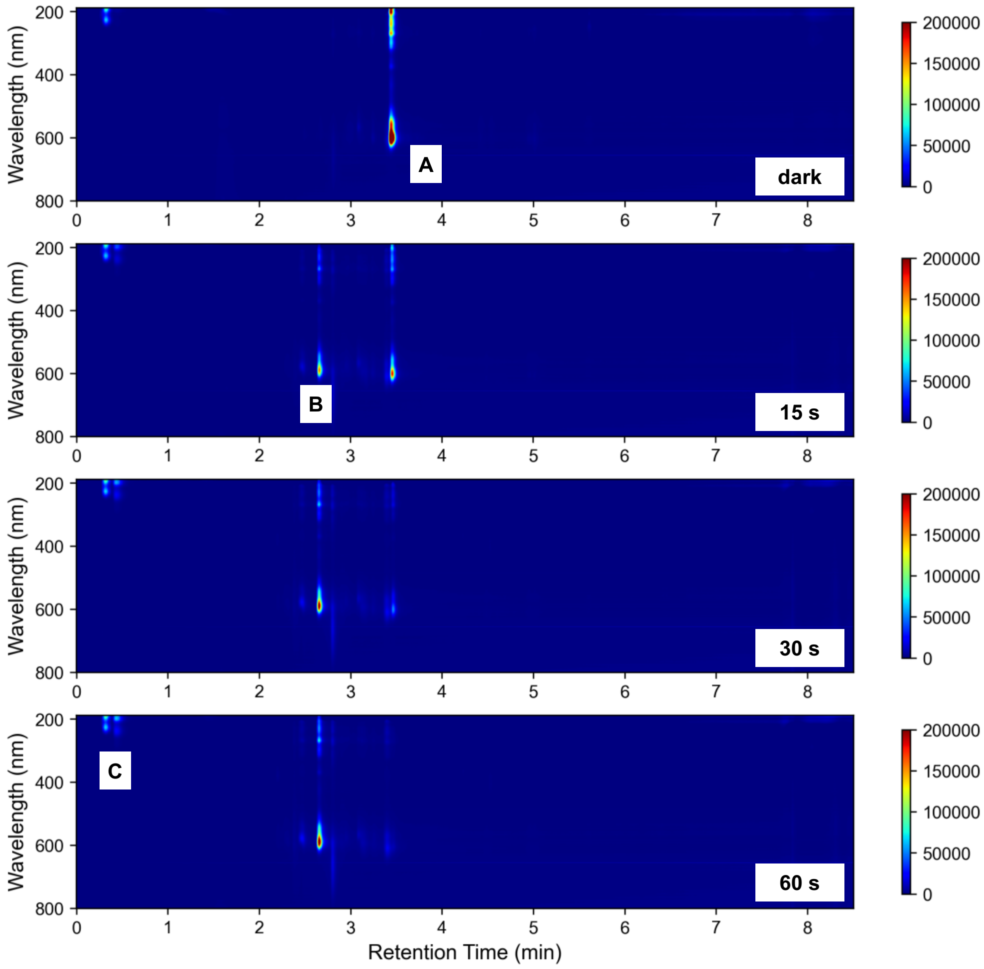

*Figure S11. Chromatograms of the photolysis of **X600H-PP** as followed by HPLC-UV/Vis-MS instrument.*

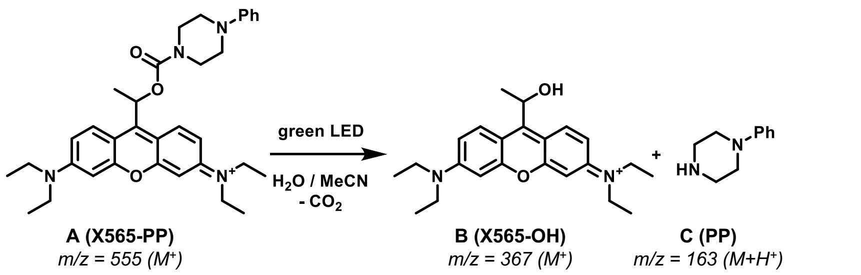

irradiation in H_2O/MeCN 9:1 with green light:

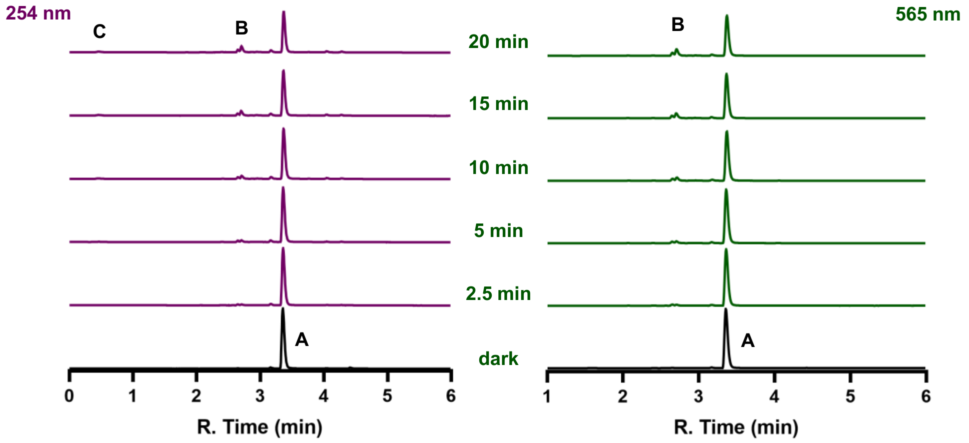

irradiation in H_2O/MeCN 9:1 with green light (2D contour chromatograms):

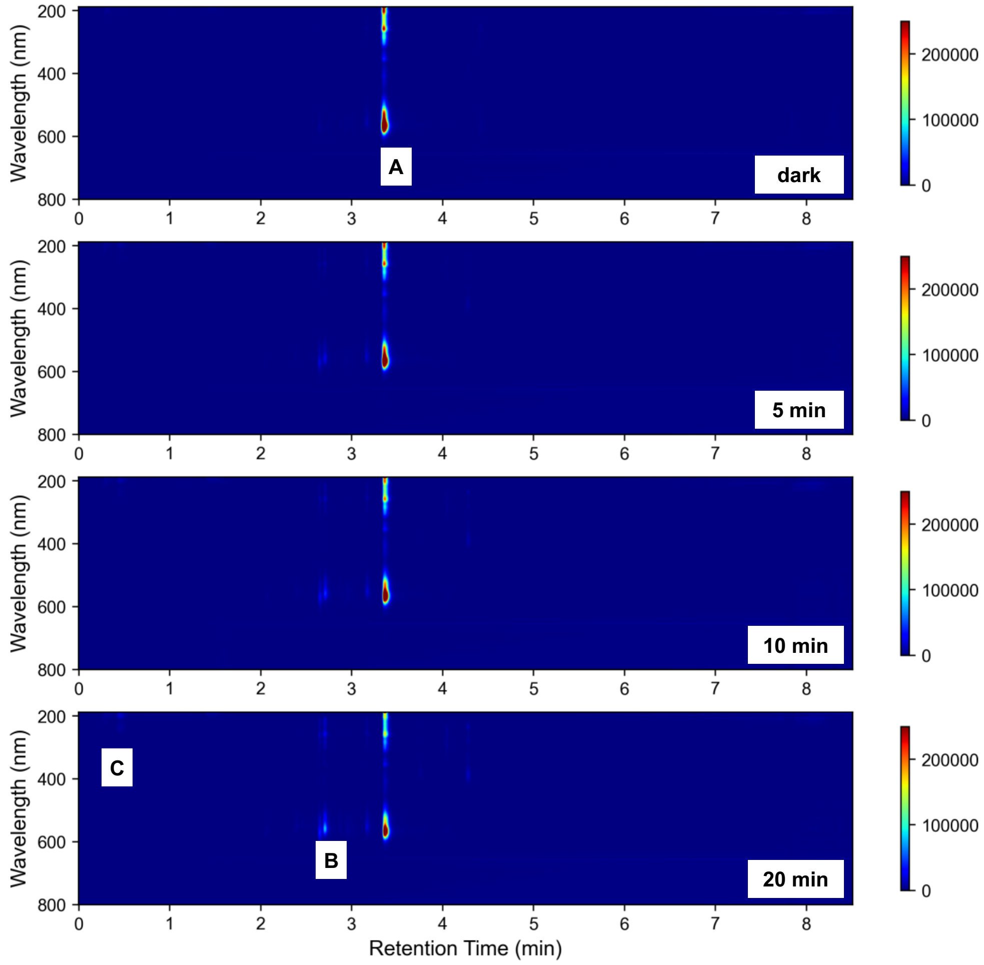

*Figure S12. Chromatograms of the photolysis of **X565-PP** as followed by HPLC-UV/Vis-MS instrument. Note that this experiment was performed using a green LED set at a lower intensity.*

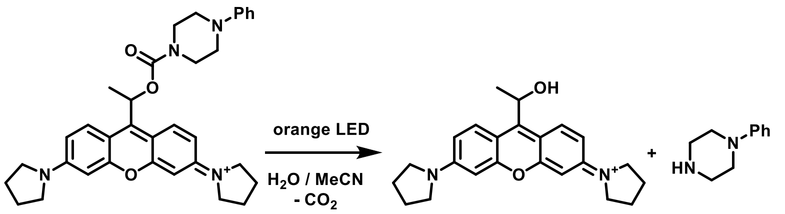

A (X570-PP) *m/z = 551 (M⁺)* **B (X570-OH)** *m/z = 363 (M⁺)* **C (PP)** *m/z = 163 (M+H⁺)*

irradiation in H_2O/MeCN 9:1 with orange light:

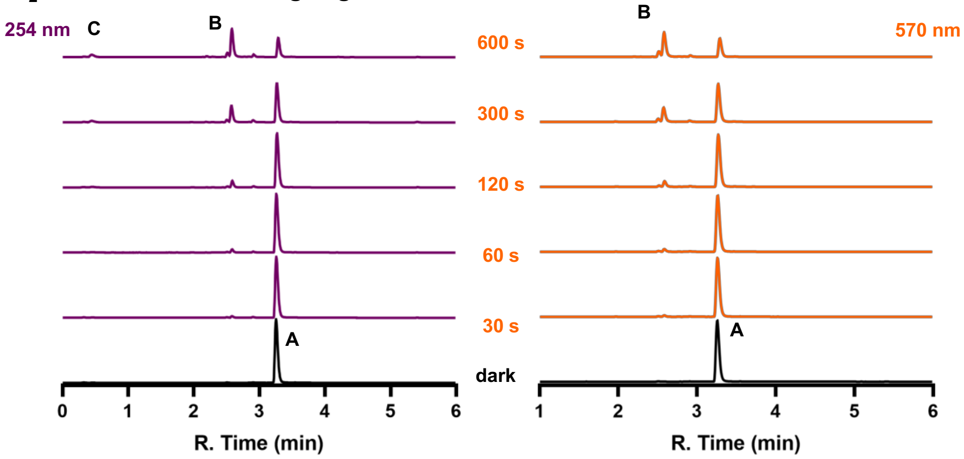

irradiation in H_2O/MeCN 9:1 with orange light (2D contour chromatograms):

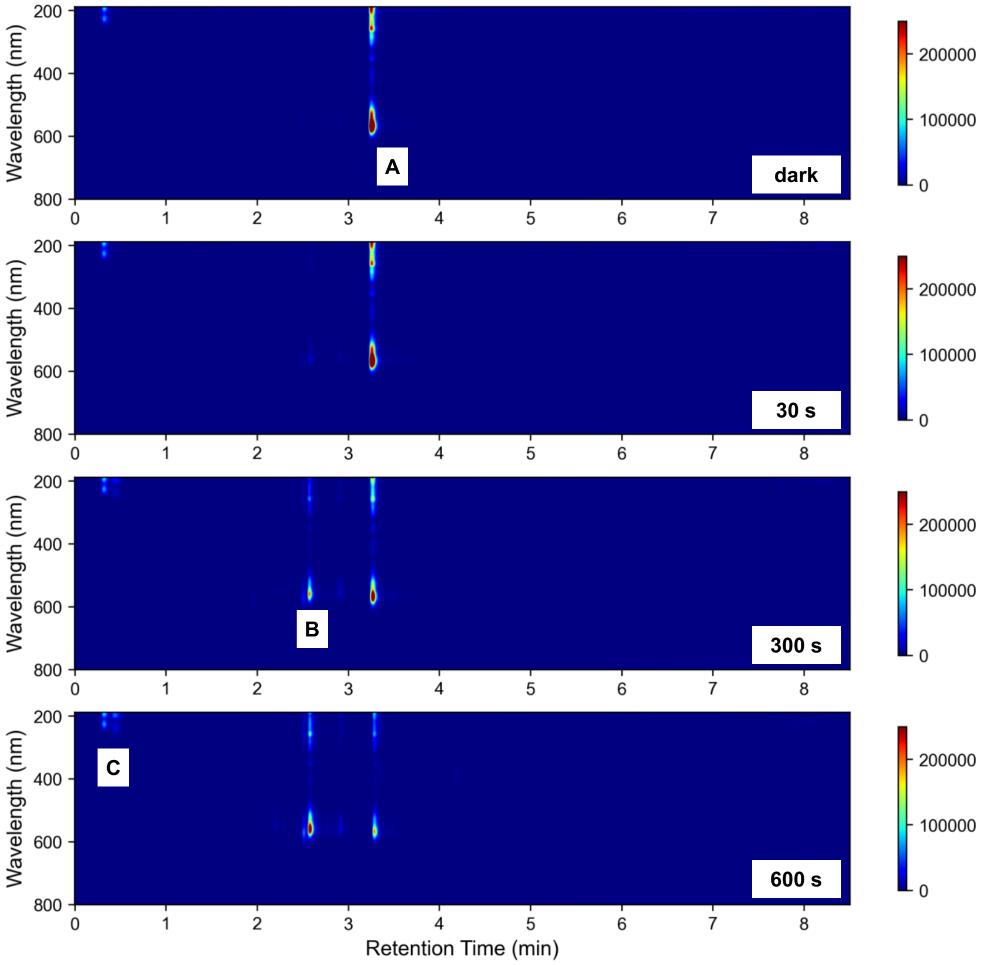

*Figure S13. Chromatograms of the photolysis of **X570-PP** as followed by HPLC-UV/Vis-MS instrument.*

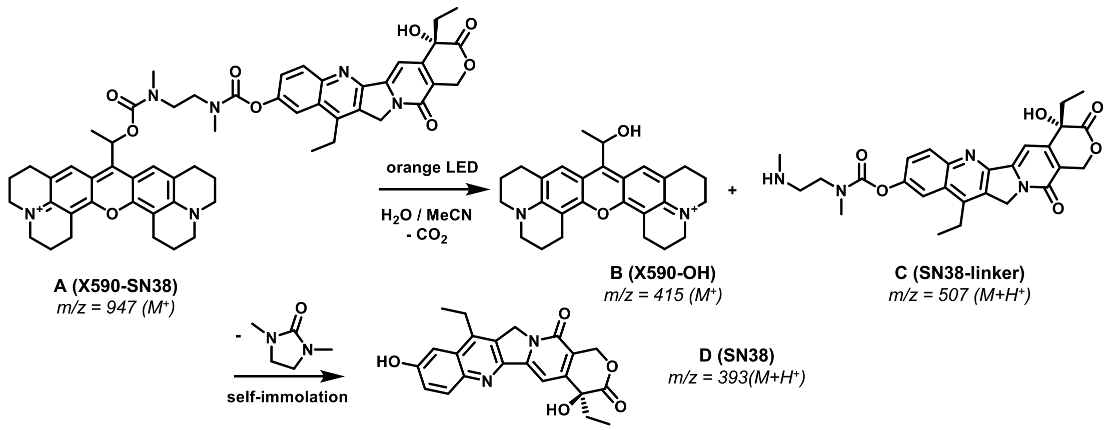

irradiation in H_2O/MeCN 9:1 with orange light:

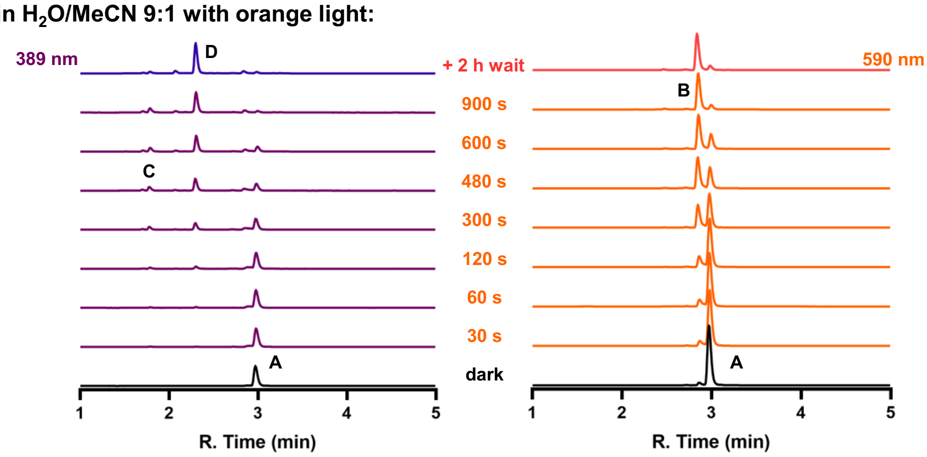

irradiation in H_2O/MeCN 9:1 with orange light (2D contour chromatograms):

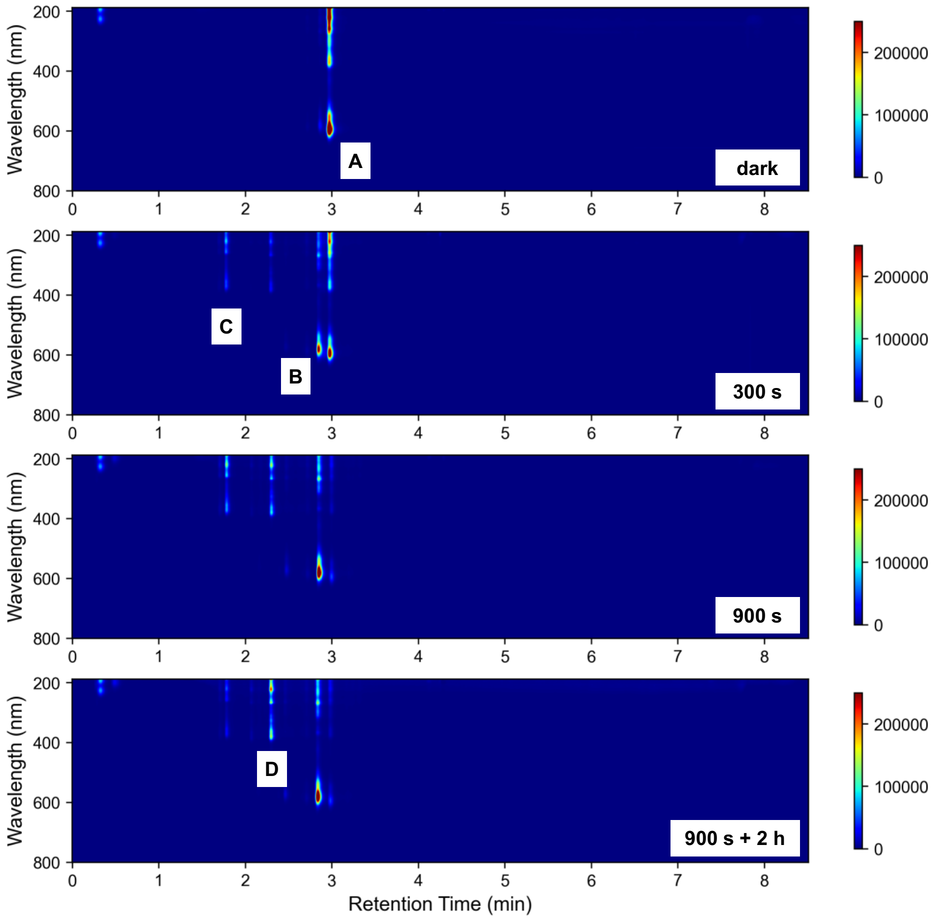

*Figure S14. Chromatograms of the photolysis of **X590-SN38** as followed by HPLC-UV/Vis-MS instrument.*

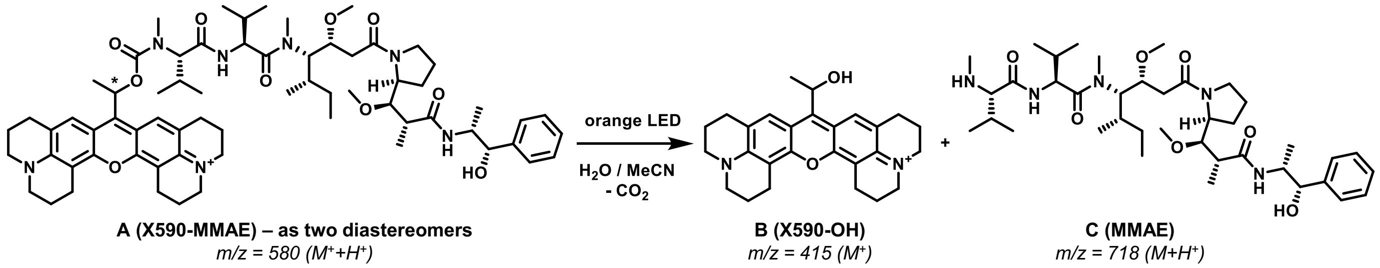

irradiation in H_2O/MeCN 9:1 with orange light:

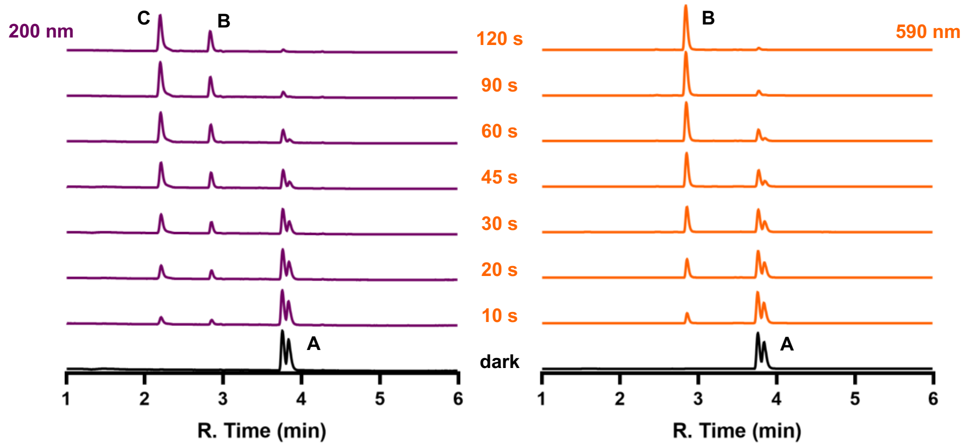

irradiation in H_2O/MeCN 9:1 with orange light (2D contour chromatograms):

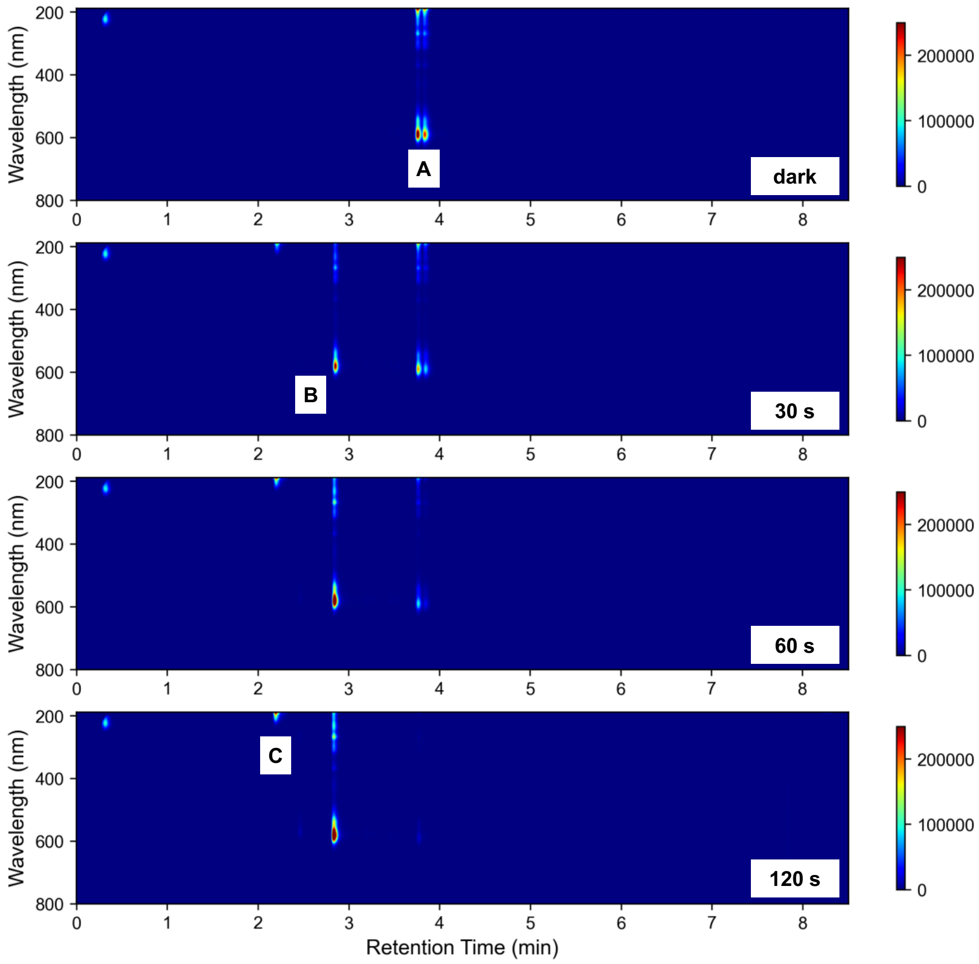

Figure S15. Chromatograms of the photolysis of ***X590-MMAE*** *as followed by HPLC-UV/Vis-MS instrument.*

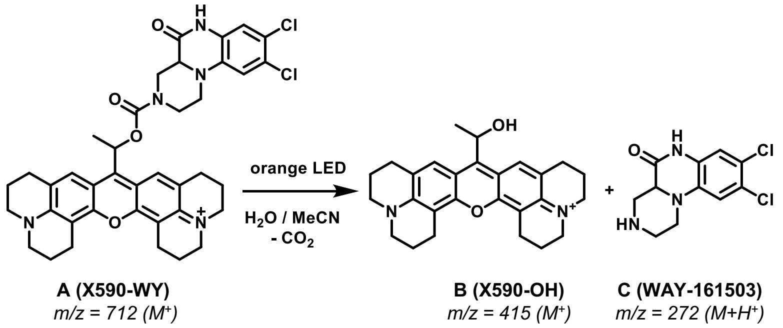

irradiation in H_2O/MeCN 9:1 with orange light:

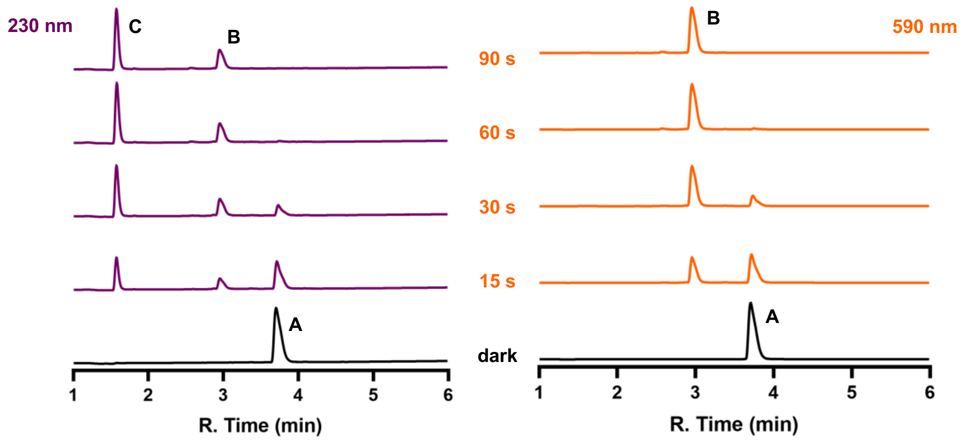

irradiation in H_2O/MeCN 9:1 with orange light (2D contour chromatograms):

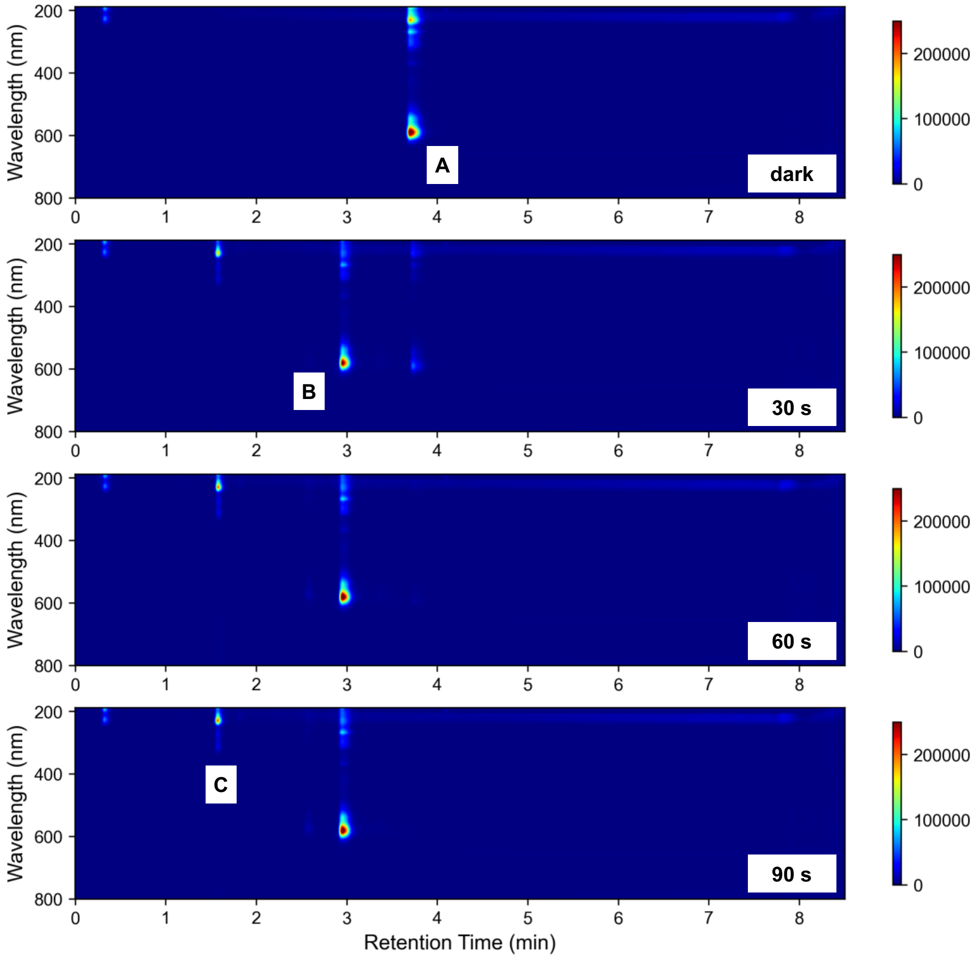

*Figure S16. Chromatograms of the photolysis of **X590-WY** as followed by HPLC-UV/Vis-MS instrument.*

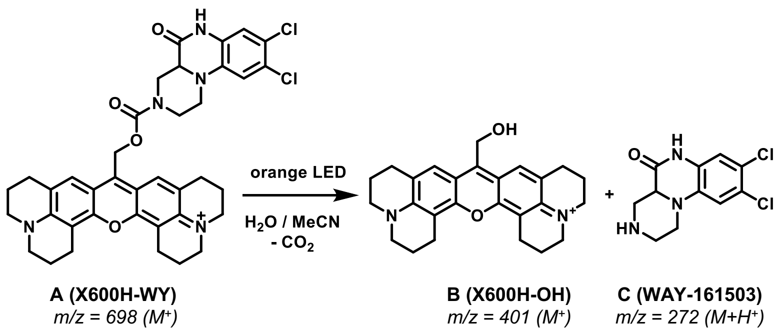

irradiation in H_2O/MeCN 9:1 with orange light:

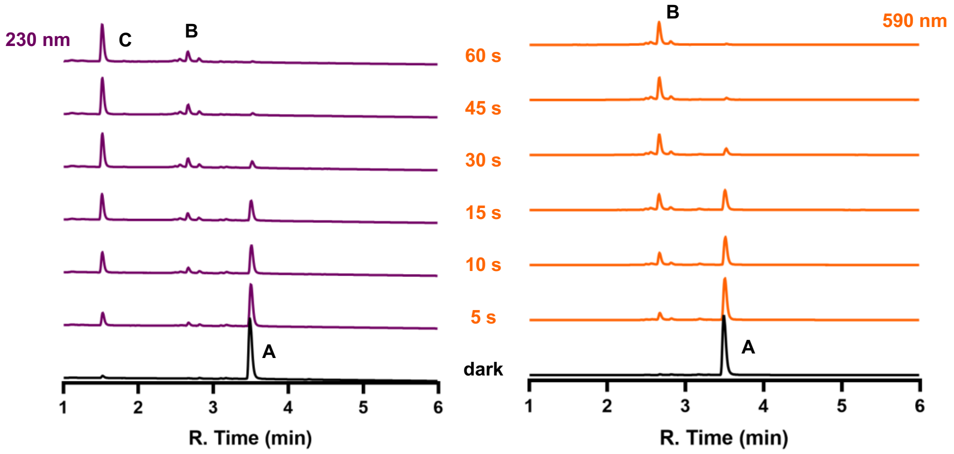

irradiation in H_2O/MeCN 9:1 with orange light (2D contour chromatograms):

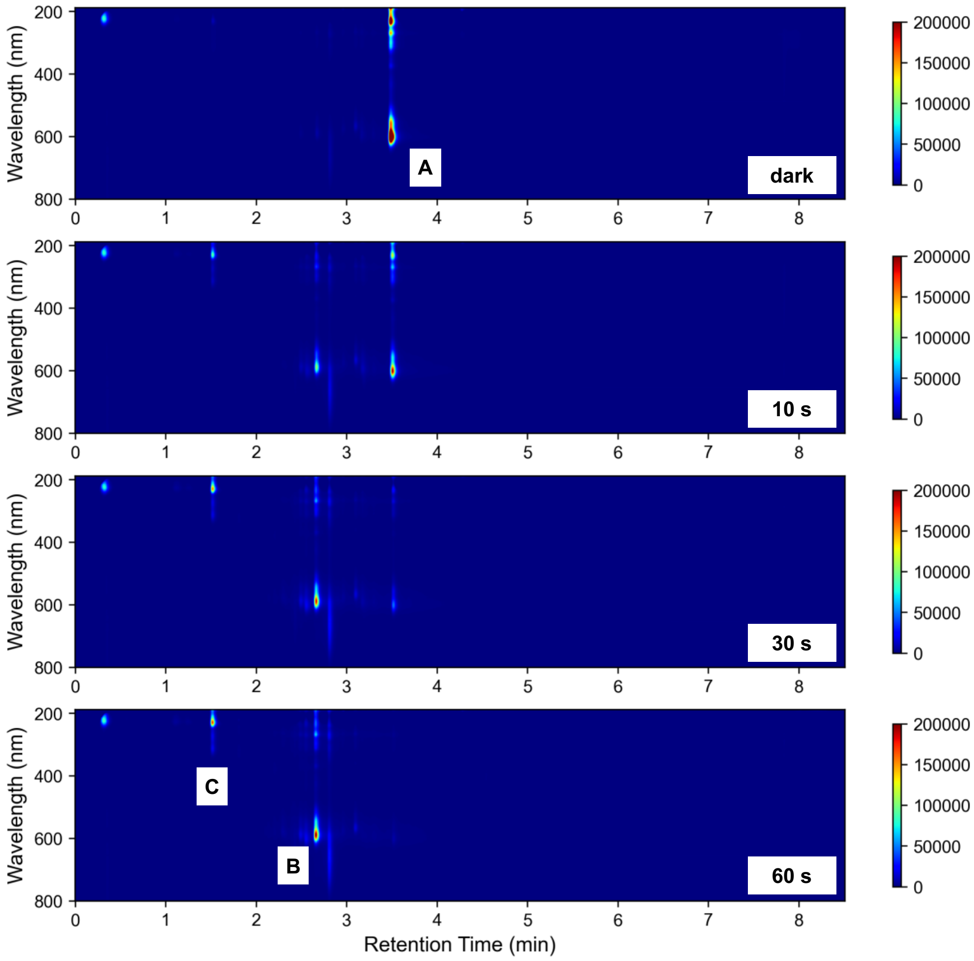

*Figure S17. Chromatograms of the photolysis of **X600H-WY** as followed by HPLC-UV/Vis-MS instrument.*

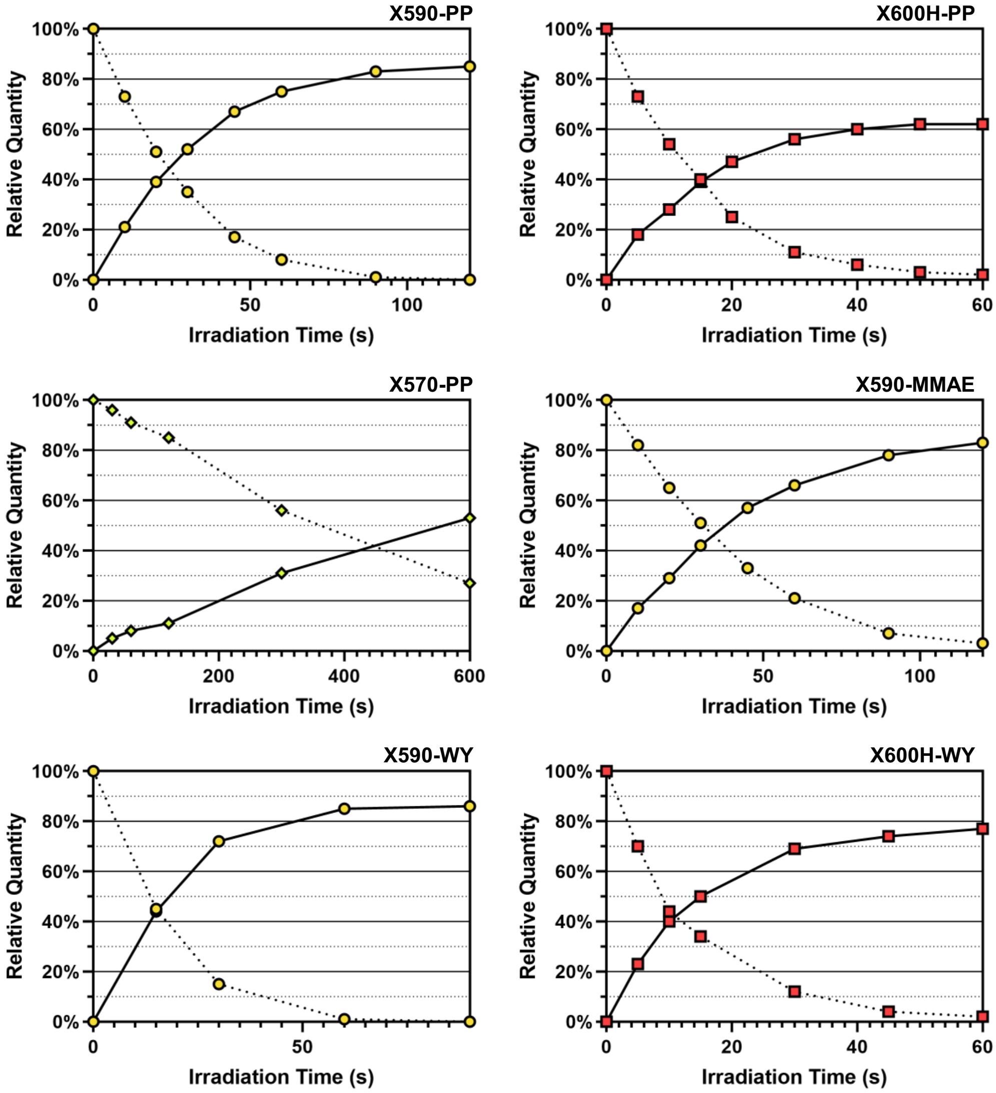

Figure S18. Representative uncaging curves showing the relative amounts of the starting material (dashed line) and released payloads (solid line) upon orange light irradiation based on the HPLC-UV/Vis integrals. Note the different time axis between the plots.

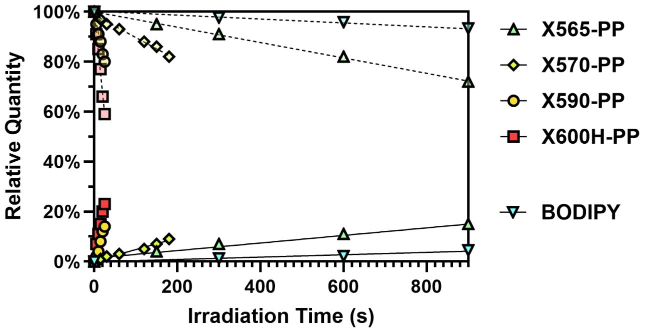

Figure S19. Representative initial uncaging curves showing the relative amounts of the starting material (dashed line) and released payloads (solid line) upon irradiation with green light (50% power setting). These data (in triplicates) were used for the quantum yield determination. See Section 5.5 for more information.

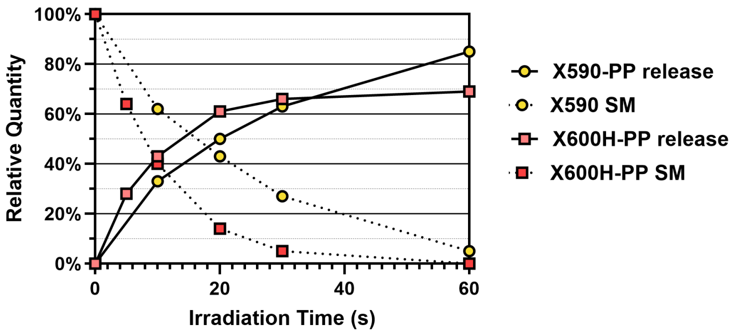

Figure S20. Uncaging curves showing the relative amounts of the starting material (dashed line) and released payloads (solid line) upon red (630 nm) light irradiation

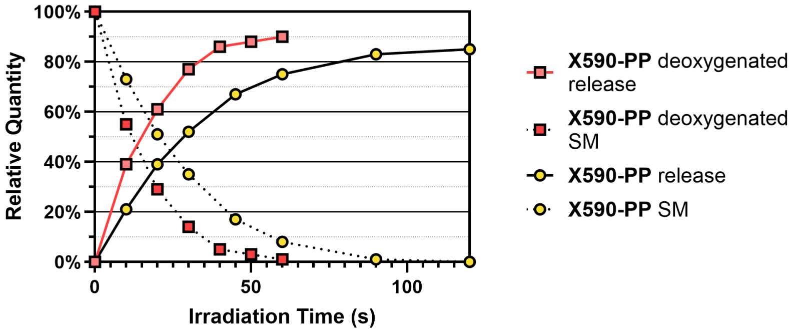

Figure S21. Uncaging curves showing the relative amounts of the starting material (dashed line) and released payloads (solid line) upon orange light irradiation of ***X590-PP*** *with the exclusion of oxygen*

5.3 Uncaging Experiments Followed by Optical Spectroscopy Methods

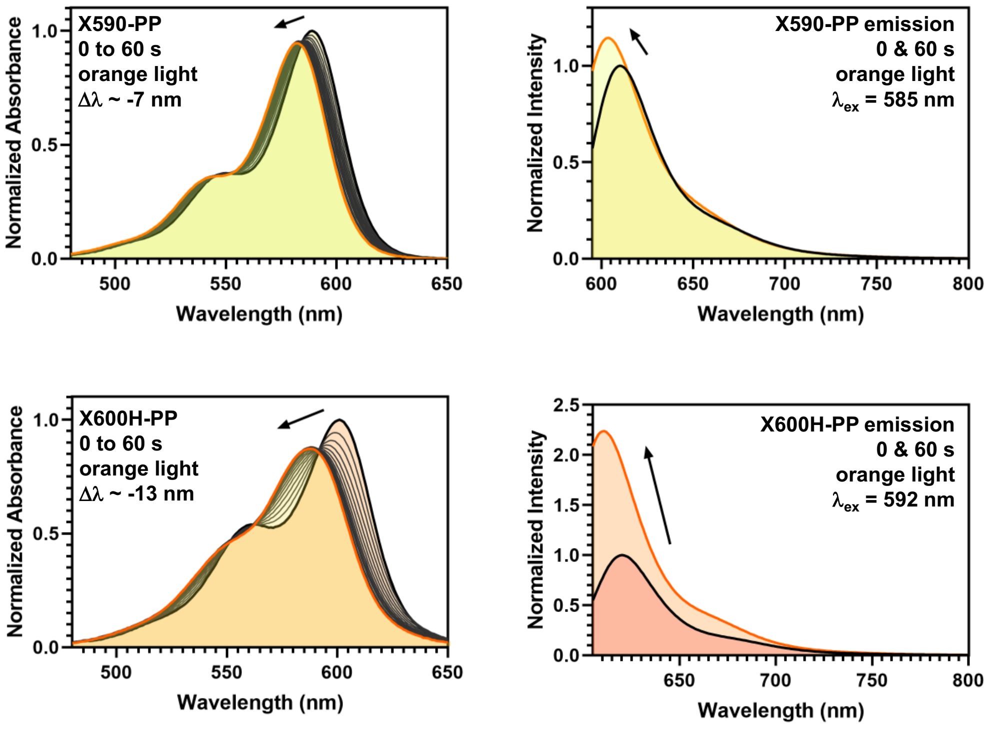

Figure S22. Normalized absorption and fluorescence spectra of ***X590-PP*** *and* ***X600H-PP*** *(~5 μM, >99% water) upon orange light irradiation (at 25% power setting compared to HPLC experiments, ~65 mW output power)*

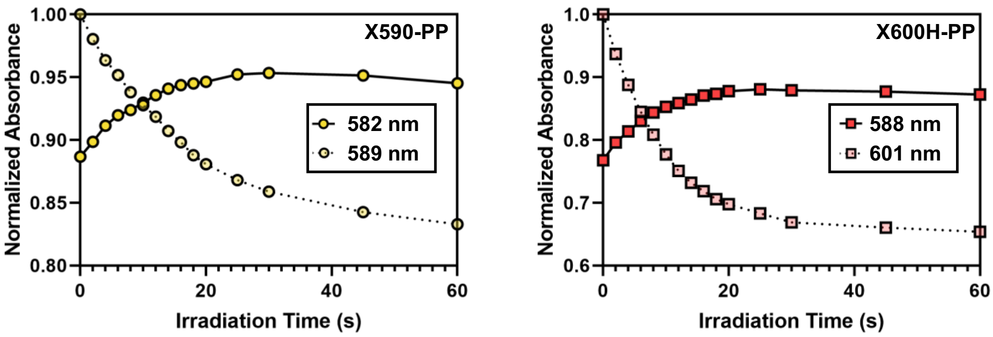

Figure S23. Normalized absorption values measured at the absorption maxima of respective species of ***X590-PP*** *and* ***X600H-PP*** *(~5 µM, >99% water) upon orange light irradiation and (at 25% power setting compared to HPLC experiments, ~65 mW output power).*

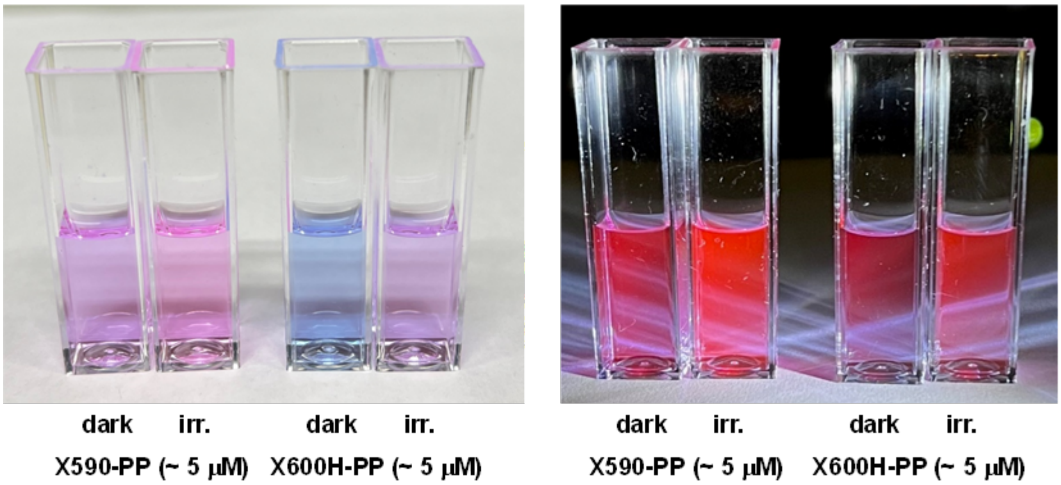

Figure S24. Photographs of the spectroscopy samples under ambient light and a white flashlight showing the fluorogenicity of ***X600H****.*

5.4 Dark Stability

5.4.1 Dark Stabilities Followed by HPLC-MS

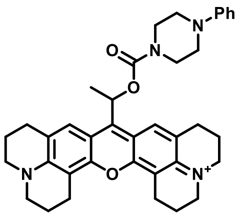

X590-PP in H_2O/MeCN 9:1 in the dark:

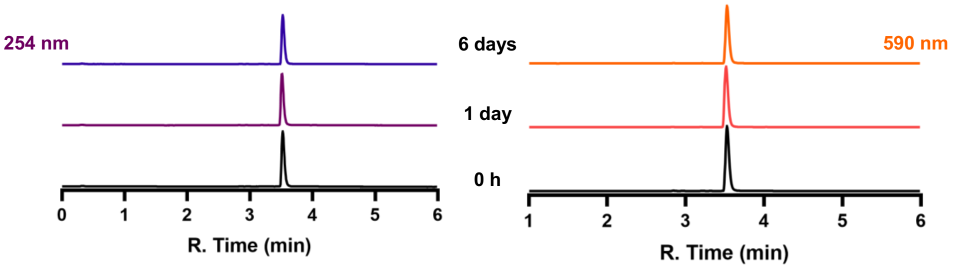

X590-PP in H_2O/MeCN 9:1 in the dark (2D contour chromatograms):

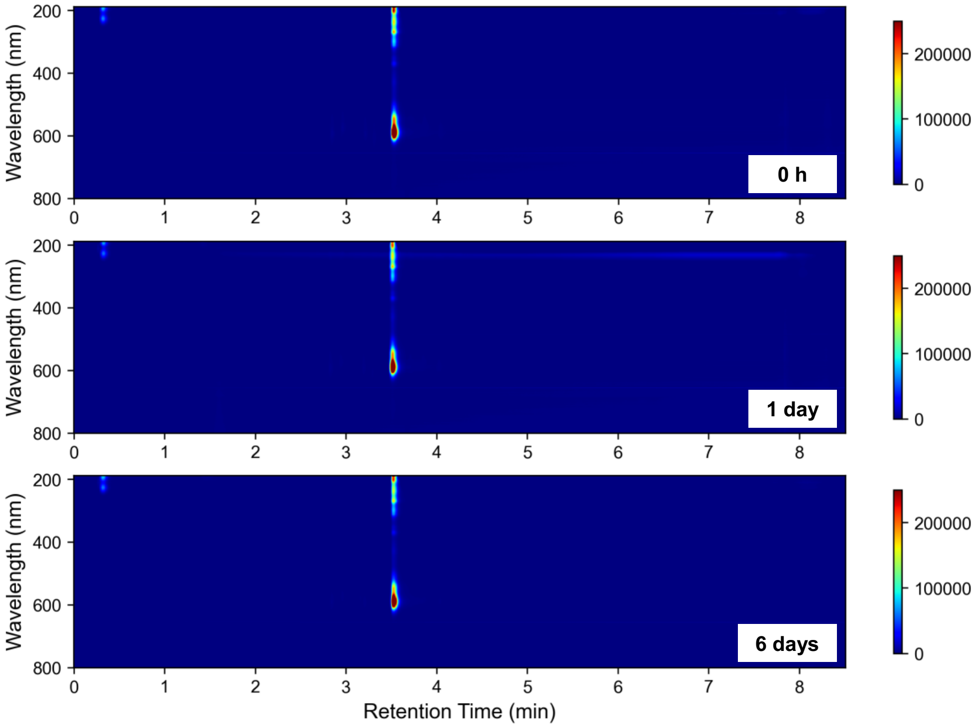

Figure S25. Chromatograms of the dark stability of ***X590-PP***

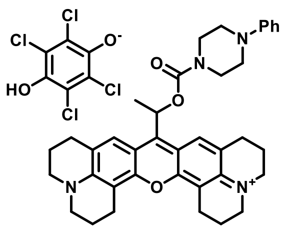

X590-PP (hydroquinone salt) in H_2O/MeCN 9:1 in the dark:

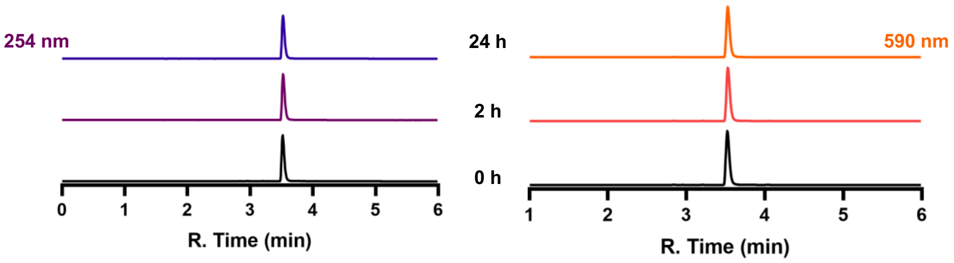

X590-PP in H_2O/MeCN 9:1 in the dark (2D contour chromatograms):

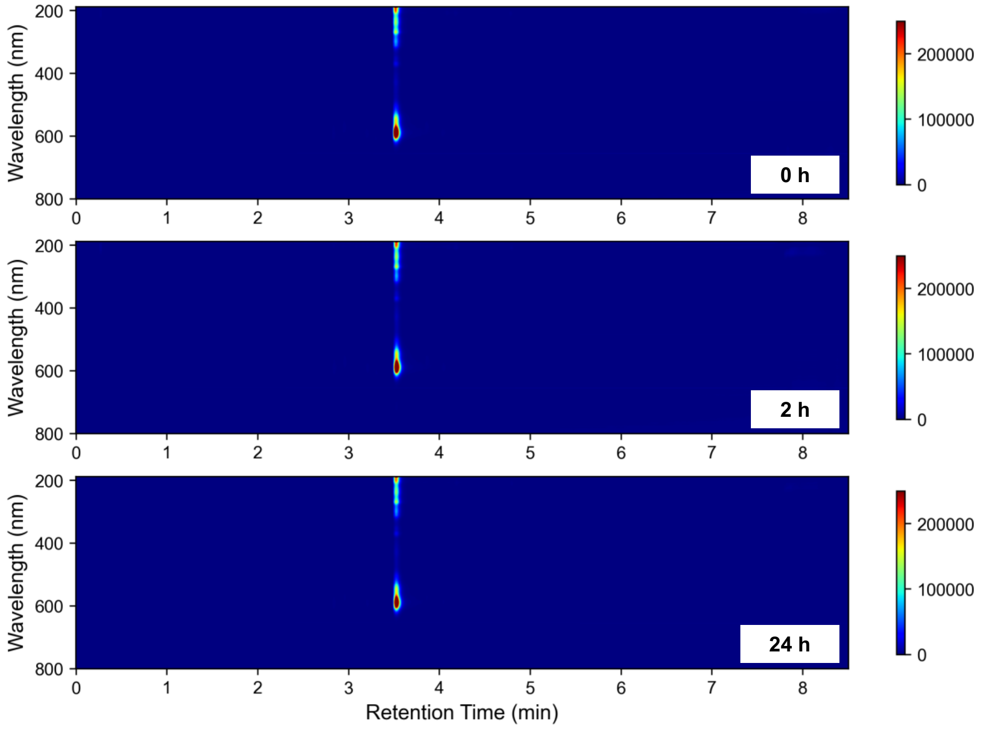

*Figure S26. Chromatograms of the dark stability of **X590-PP (hydroquinone salt)***

X600H-PP in H_2O/MeCN 9:1 in the dark:

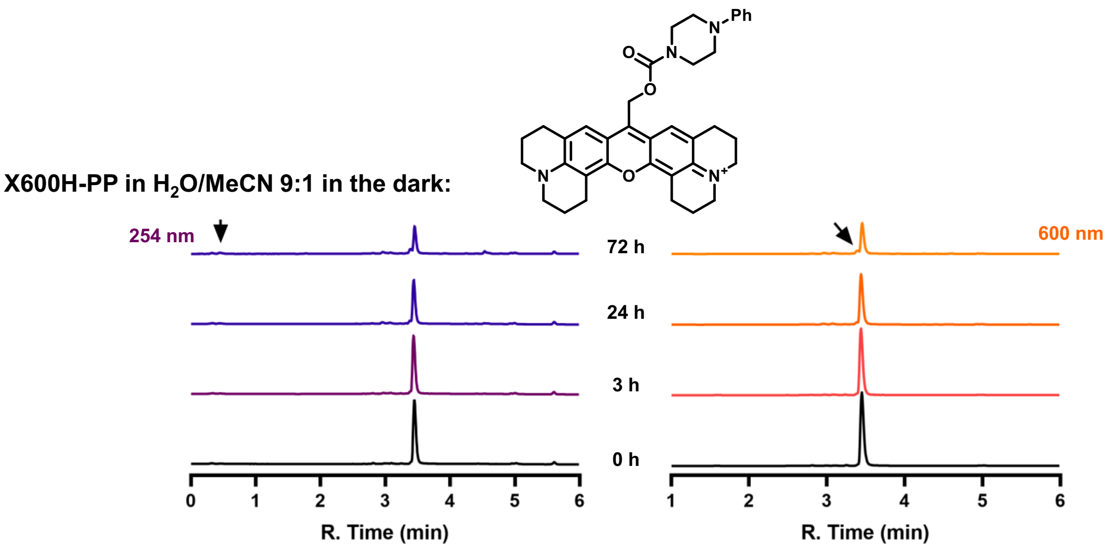

X600H-PP in H_2O/MeCN 9:1 in the dark (2D contour chromatograms):

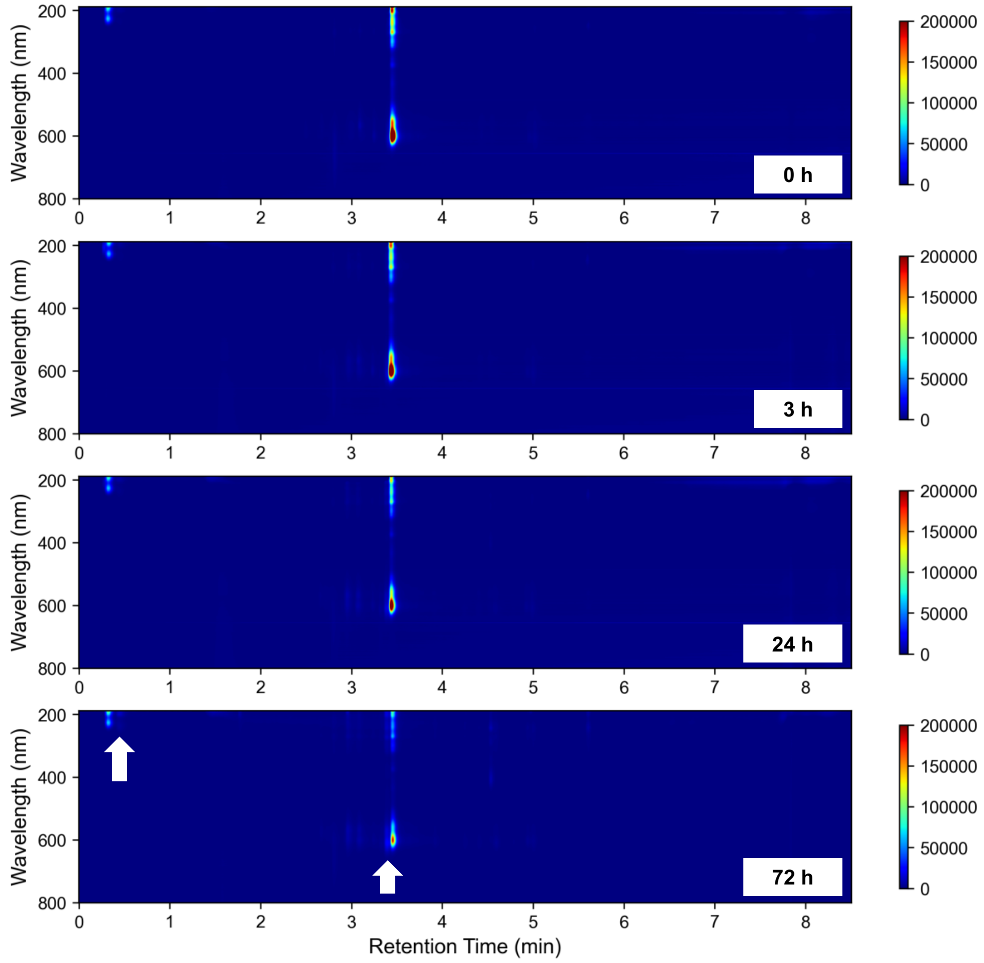

*Figure S27. Chromatograms of the dark stability of **X600H-PP**. The arrows indicate minor degradation products.*

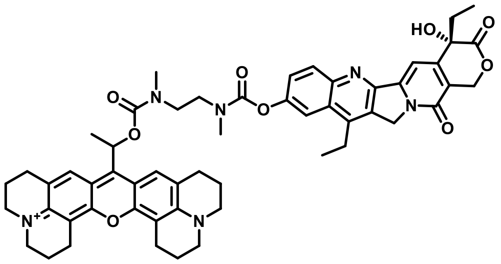

X590-SN38 in H_2O/MeCN 9:1 in the dark:

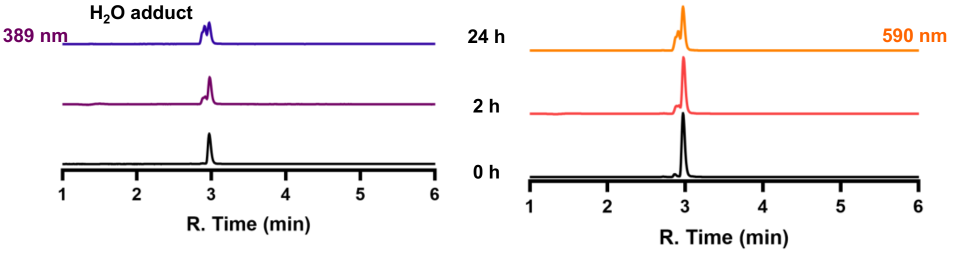

X590-SN38 in H_2O/MeCN 9:1 in the dark (2D contour chromatograms):

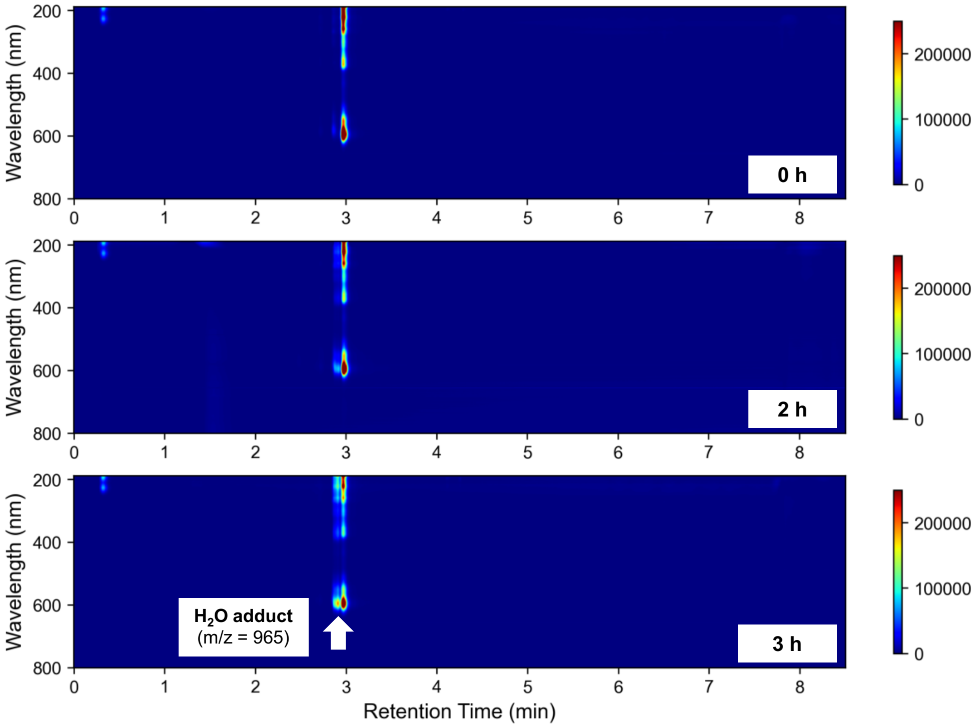

Figure S28. Chromatograms of the dark stability of ***X590-SN38.***

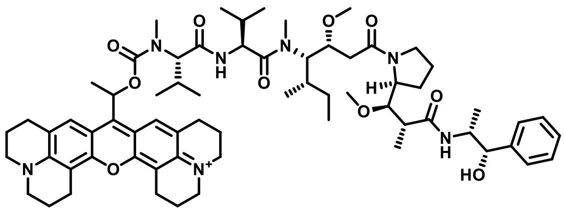

X590-MMAE in H_2O/MeCN 9:1 in the dark (note the two diastereomers):

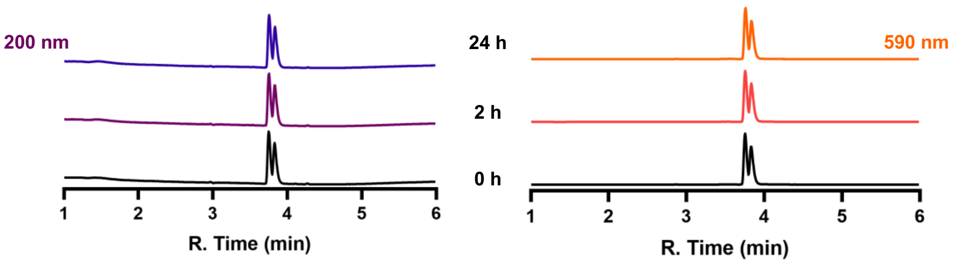

X590-MMAE in H_2O/MeCN 9:1 in the dark (2D contour chromatograms):

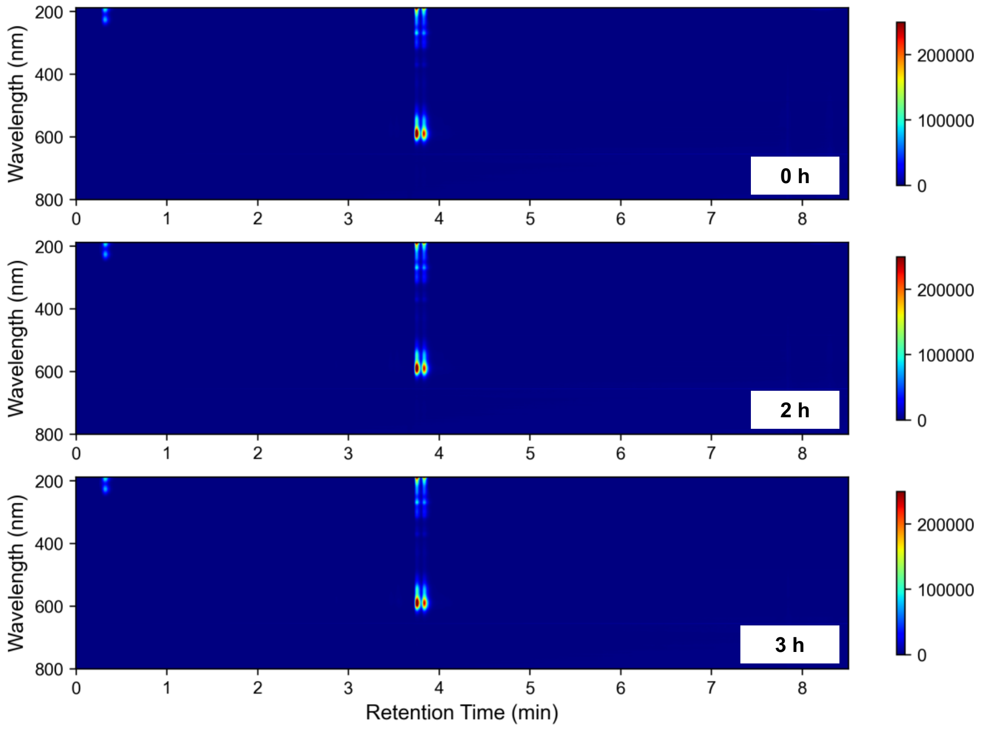

Figure S29. Chromatograms of the dark stability of ***X590-MMAE***

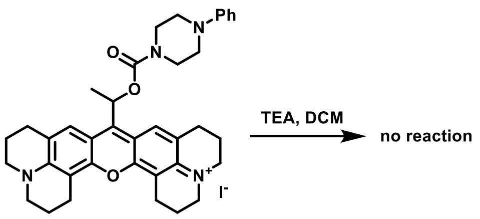

treatment with 10 equivalent triethylamine:

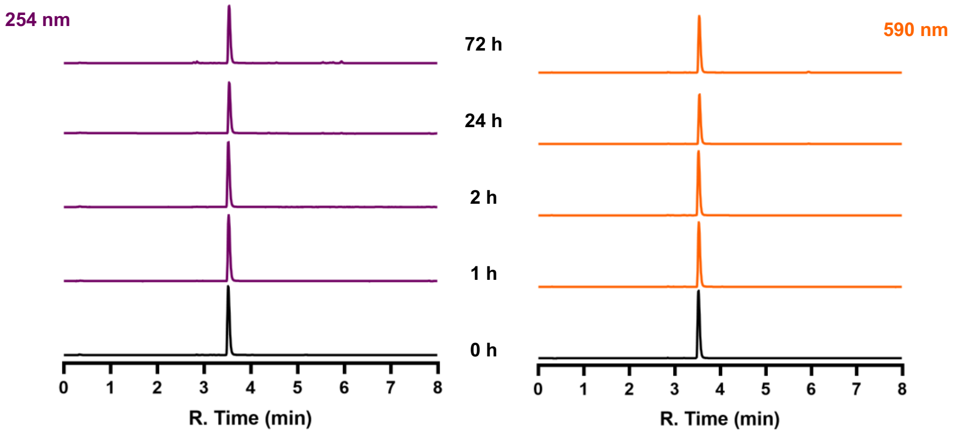

treatment with 10 equivalent triethylamine (2D contour chromatograms):

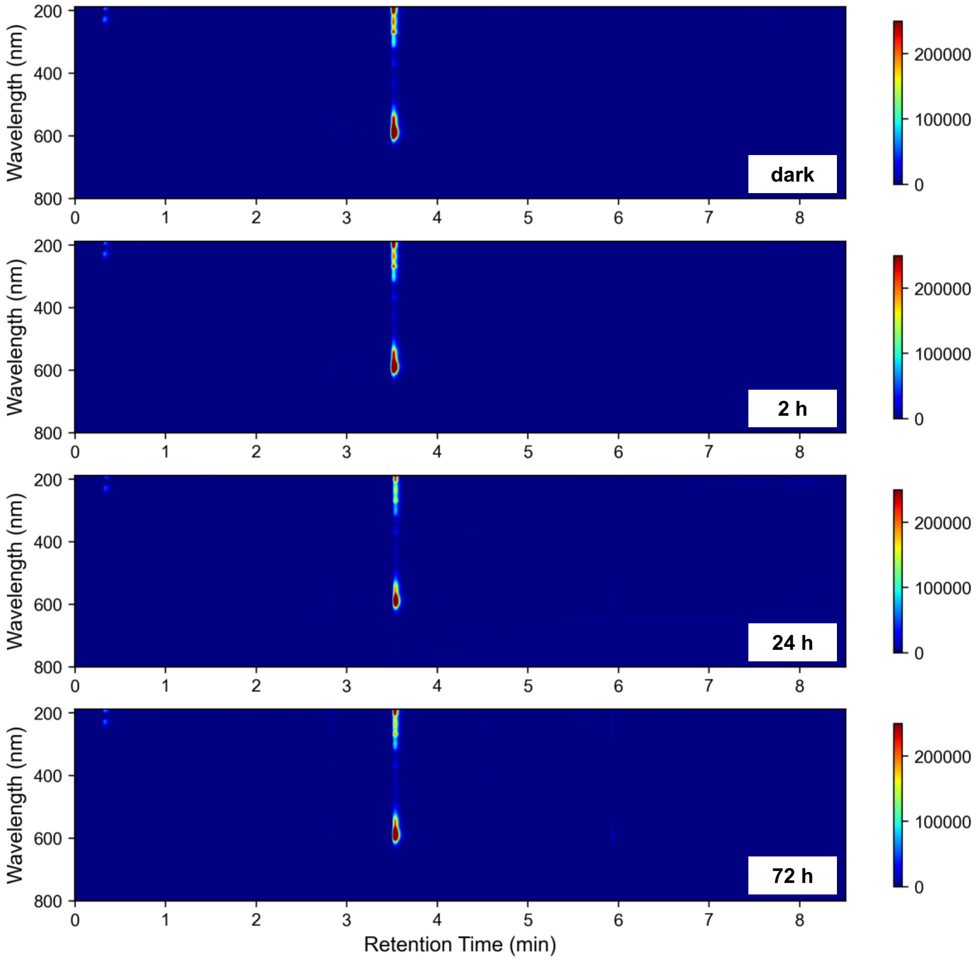

*Figure S30. Chromatograms of the treatment of **X590-PP** with triethylamine as followed by HPLC-MS. No exo form was observed.*

5.4.2 Dark Stabilities Followed by UV/VIS

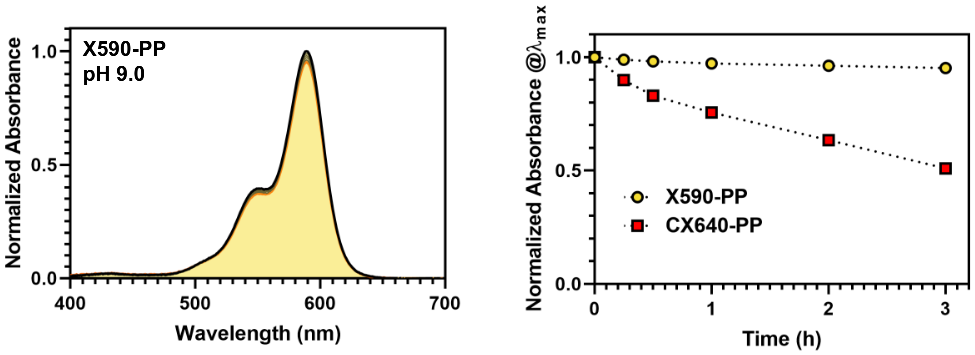

*Figure S31. Normalized absorption spectra of **X590-PP** (~3 μM, Britton-Robinson buffer, pH 9.0) recorded over time. Normalized absorbance values are plotted on the right.*

5.5 Photochemical and Singlet Oxygen Quantum Yield Determination

The relative photochemical quantum yields of the uncaging (payload release) together with the degradation quantum yields were determined as described in [2], from the results of the quantitative HPLC-UV experiments and shown in Table 1 in the main text. In all cases, the green LED (at 50% power setting) was used as all compounds absorbed in this range as well as the reference standard BODIPY from [9]. The experiments in case of the model conjugates were repeated 3 times. The photochemical quantum yields were determined using the following equation:

$$\Phi_u = \Phi_{u,ref} \cdot \frac{k \cdot c \cdot V \cdot a_{ref}}{k_{ref} \cdot c_{ref} \cdot V_{ref} \cdot a}$$

where

Φ_u	is the photochemical quantum yield of the sample
$\Phi_{u,ref}$	is the photochemical quantum yield of the reference
k	is the obtained reaction rate constant of the photoreaction of the sample
k_{ref}	is the obtained reaction rate constant of the photoreaction of the reference
c	is the concentration of the sample
c_{ref}	is the concentration of the reference
V	is the volume of the sample
V_{ref}	is the volume of the reference
a	is the absorption correction factor of the sample
a_{ref}	is the absorption correction factor of the reference

The absorption correction factors (a) were determined from the molar absorption coefficients and the green LED emission data obtained by a fluorimeter using the following equation:

$$a = \sum_{\lambda = \lambda_{min}}^{\lambda_{max}} \left[(1 - T(\lambda)) \frac{F(\lambda)}{\sum_{\lambda = \lambda_{min}}^{\lambda_{max}} [F(\lambda)]} \right]$$

where

$T(\lambda)$	is the transmittance at each wavelength (determined from the molar absorption coefficients)
$F(\lambda)$	is the LED emission at each wavelength
$\lambda_{min}, \lambda_{max}$	is the LED emission range

In brief, first we have calculated the transmittance at each wavelength (usual path length: 1.4 cm). Calculating 1-T at each wavelength results in the efficiency of photon absorption which was weighed at each wavelength with the emission of the LED with a normalized area under the curve of 1. Note that in the case of xanthenium photocages, complete absorption occurs.

The rate constants of the photoreactions were determined by considering the reactions monoexponential with an initial linear phase (up to 10-20% degradation). First, the concentration vs. HPLC-UV peak were determined for each leaving group and starting material. Then, in each case, a linear fitting of the time vs. released cargo concentrations was performed that resulted in the rate constant k as determined from the fitted linear equation. The rate constants were determined for both the reference (with known Φ_u) and the sample. The determination of the degradation quantum yields was similar; in these cases the linear fitting was performed on the starting material concentrations. For reference, a commonly known benchmark BODIPY derivative was used (in methanol, as reported) with phenylacetic acid as payload (Fig. S33, substrate **7** from [9]), Φ_u = 0.15%:

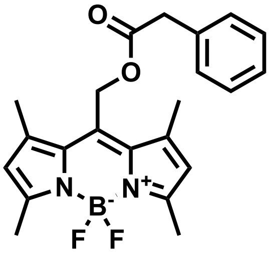

Figure S32. Structure of the reference BODIPY compound

The singlet oxygen quantum yields were determined by first performing reference measurement. A solution containing methylene blue (10 µM) and diphenyl-isobenzofuran (DPBF 50 µM) was prepared in water/MeCN 1:1 in a cuvette. The sample was irradiated with a green LED at 15% intensity in 0.5 s intervals. Subsequently, the photocaged compound was measured under identical conditions at a concentration of 10 µM, using the same irradiation protocol (i.e., cumulative irradiation in 0.5 s steps, e.g. 1 s = 2 × 0.5 s). The consumption of DPBF was monitored by following the decrease in absorbance at 411 nm.

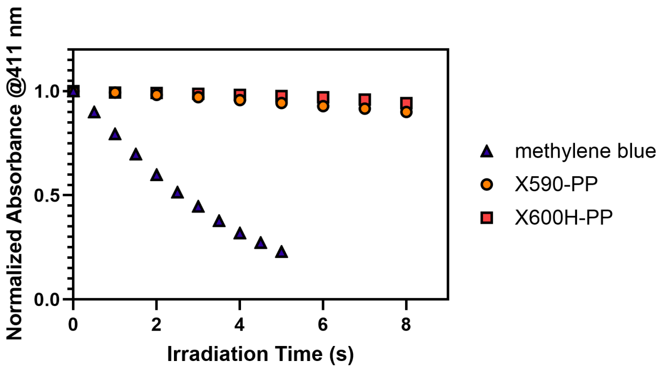

Figure S33. Normalized absorption of DPBF upon green light irradiation. LED power was reduced to 15%.

6. 2D Cell Culture for Photoactivated Chemotherapy Experiments

HeLa (human cervix adenocarcinoma, epithelial), U2OS (osteosarcoma, epithelial), and U87-MG (glioblastoma, epithelial) cells were maintained in Dulbecco's modified Eagle's medium (DMEM, Gibco 41965-039) supplemented with 10% FBS (Gibco A5256801), 1% penicillin-streptomycin (Gibco 15140-122), 1% sodium pyruvate (Gibco 11360-070) and 1% Glutamax (Gibco 35050-061). A549 (lung carcinoma, epithelial) and HCT-116 (colorectal carcinoma; epithelial) cells were maintained in RPMI 1640 medium (Gibco 11875093) supplemented with 10% FBS (Gibco A5256801), 1% penicillin-streptomycin (Gibco 15140-122). SK-OV-3 (ovarium, adenocarcinoma; epithelial) cells were maintained in McCoy 5A medium (Gibco 22330021) supplemented with 10% FBS (Gibco A5256801), 1% penicillin-streptomycin (Gibco 15140-122). Each cell line was cultured at 37°C in a 5% CO_2 atmosphere and passaged - using trypsin (0.05%; Gibco 25300-054) - every 3–4 days up to 20 passages.

6.1 Cell Viability Studies

6.1.1 Experimental Details for the Cell Viability Studies

Viability test was performed to evaluate the toxicity of MMAE and **X590-MMAE** (in the dark or after light irradiation) using the resazurin-resorufin assay. Cells were plated on 48-well plate (Nunc Biolite Thermo Fisher 130187) at the given cell concentration (cell number/well: A549: 6000; HeLa: 6000; HCT-116: 4500; SK-OV-3: 4500; U2OS: 6000; U87-MG: 4500) and cultured for 16-24 h at 37°C in a 5% CO_2 atmosphere before treatment. In case of U87 cells culture plate was treated with 20 ug/ml poly-D-lysine (Gibco, A3890401) for 4 hours before plating.

Compounds were applied at concentrations ranging from 10^{-12} to 10^{-5} M diluted in cell culture medium and incubated for 90 minutes (control treatment: 0.1% DMSO). Following treatment, cells were either exposed to light irradiation (605 nm LED) for 60 s (4 cycles of 15 s irradiation with 15 sec intermittent pause) or kept in the dark. Samples were incubated for 72 hours in the dark at 37°C in a 5% CO_2 atmosphere. Medium was replaced with 40 µM resazurin solution (in DMEM) and incubated for 2 hours. Fluorescence was measured at 530 nm excitation and 560 nm emission wavelength using a Biotek Synergy 2 Cytation 3 imaging plate reader with Gen5 software (version 3.11, Biotek, Winooski, VT, USA). Cell viability was expressed as the percentage of DMSO treated control cells (n = 3). IC_{50} values were calculated using GraphPad Prism software (ver. 8.0.1) applying non-linear regression viable slope (four parameters).

For irradiation time-dependent studies, light exposure was varied between 0-120 sec. To assess cytotoxicity and the contribution of PDT effect of the photocage, model compound **X590-PP** was applied in the concentration range of 10^{-12} to 10^{-5} M, similar as **X590-MMAE**. To evaluate the contribution of PDT effect to the cytotoxic effect of the photocage, **X590-PP** and **X590-MMAE** containing media were pre-irradiated and added to the cells (pre-irradiated on Fig. 3).

To assess cross-laboratory reproducibility between Budapest and Graz, 2D cellular IC_{50} measurements were reproduced at the Medical University of Graz (MUG). The human glioblastoma cell line U87, provided by MUG was used in the following experimental setups. U87 cells were selected due to their consistent growth behavior both in vitro and on CAM. Cells were cultured in Minimum Essential Medium Eagle (MEM Eagle, Sigma Aldrich) supplemented with 10 % fetal bovine serum (FBS, Gibco), 5 mL non-essential amino acids (NEAA, Gibco) and 5 mL L-Glutamine (Gibco). U87 cells were maintained at 37 °C at constant humidity and 5 % CO2. Adequate cell culturing required media exchange every 2-3 days, passaging upon exceeding 80% confluency with TrypLe (Thermo Fisher) and regular microscopic analysis to identify signs of contamination. In this case, the readout was performed 72 hours after treatment by examining the cell proliferation and metabolic activity using an MTS cell viability assay. The measured metabolic activity to determine the IC_{50}-curves of the U87 cell line treated with concentrations ranging from 10^{-12} to 10^{-5} M of MMAE, **X590-MMAE** (light), uncaged with Thorlabs lens system for 30 seconds, and **X590-MMAE** (dark), are shown in Figure S39. The determined IC_{50} values match well with the values measured in Budapest.

6.1.2 IC_{50} Curves

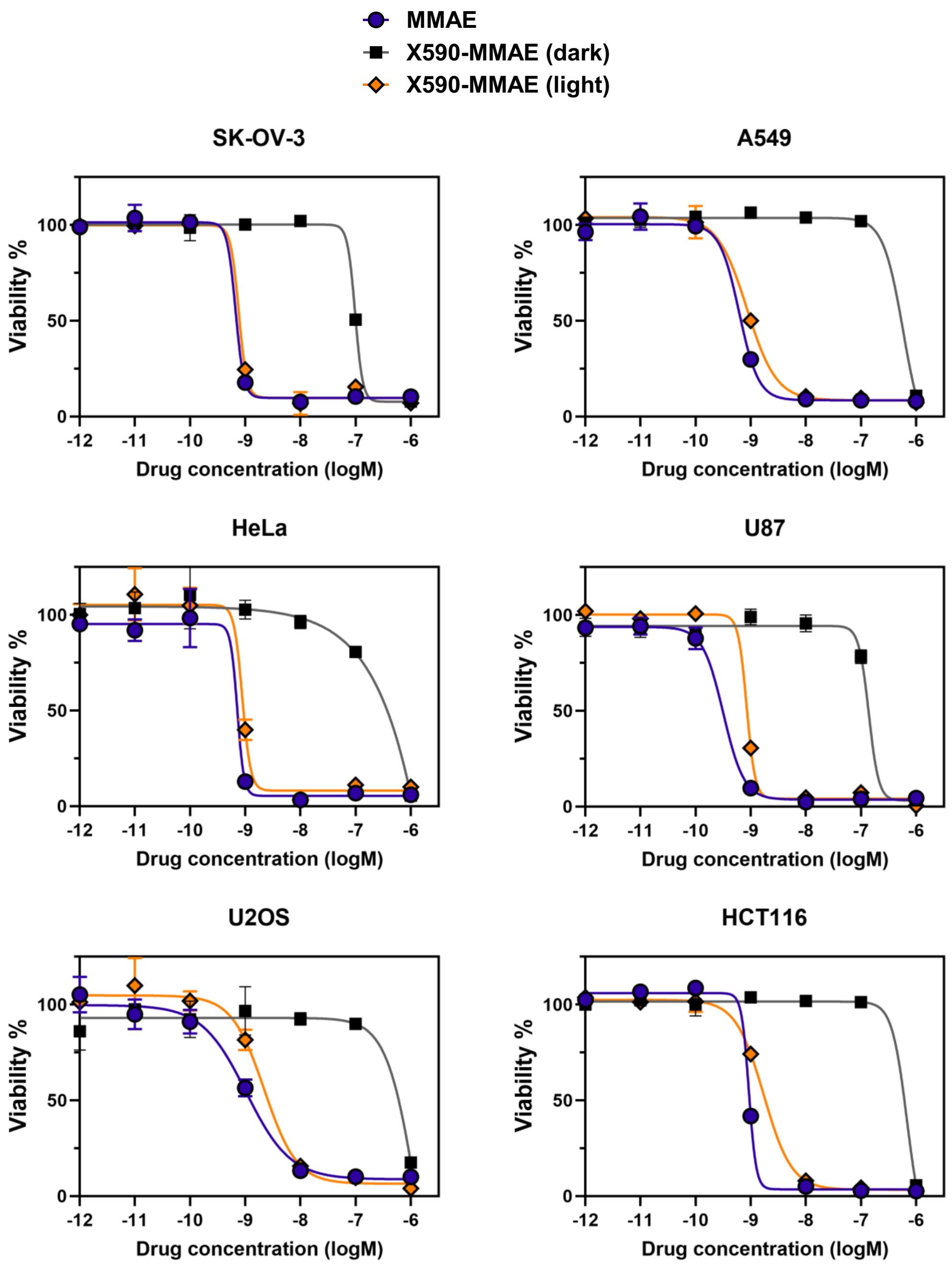

Figure S34. Concentration-dependent effects of MMAE and ***X590-MMAE*** *with or without 60 s orange light irradiation on cell viabilities of various 2D cancer cells together with their IC_{50} curves.*

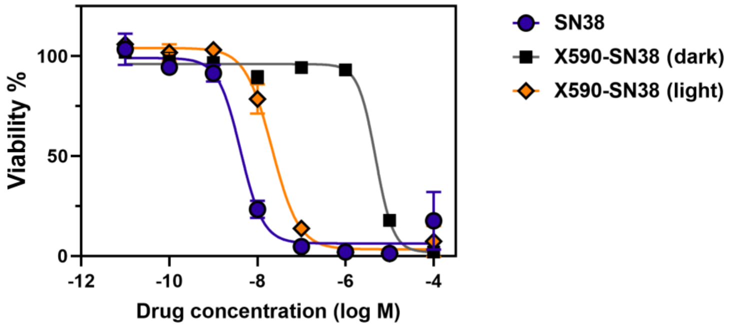

Figure S35. Concentration-dependent effects of SN38 and ***X590-SN38*** *with or without 60 s orange light irradiation on cell viabilities of various 2D cancer cells together with their IC_{50} curves.*

6.1.3 Irradiation Time-Dependent Viabilities

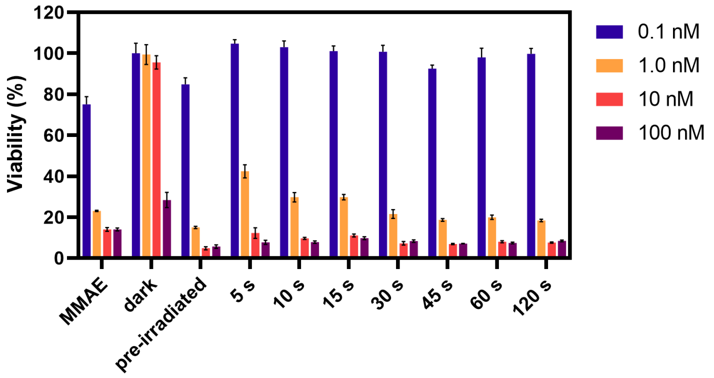

*Figure S36. Irradiation time-dependent cell viabilities of **X590-MMAE** on SK-OV-3 cells.*

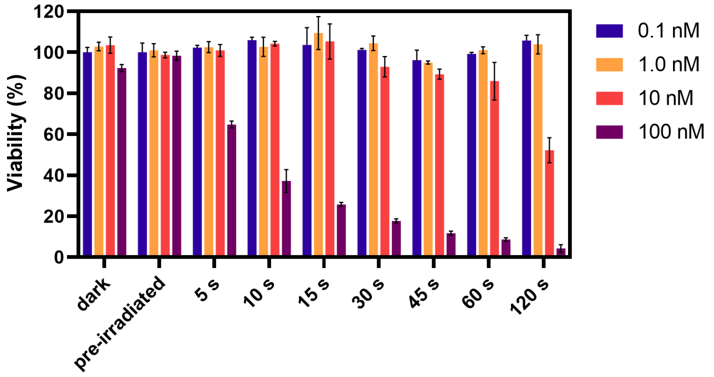

*Figure S37. Irradiation time-dependent cell viabilities of **X590-PP** on SK-OV-3 cells.*

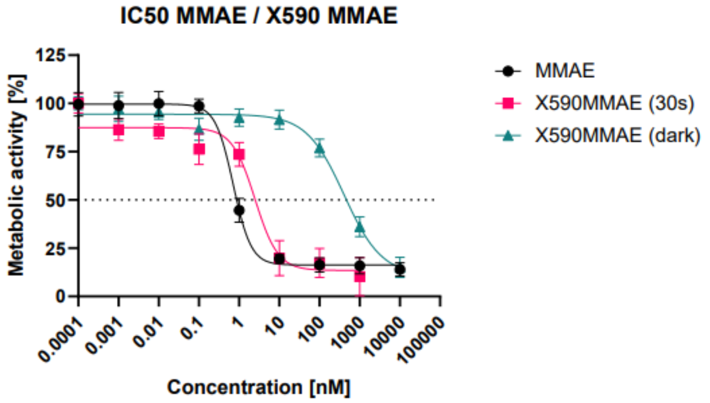

*Figure S38. Concentration-dependent effects of MMAE and **X590-MMAE** with or without 30 s orange light irradiation using the Thorlabs lens system on the viabilities of U87 cells together with their IC50 curves. Note that the experiments were performed at the Medical University of Graz as a benchmark for cross-laboratory reproducibility.*

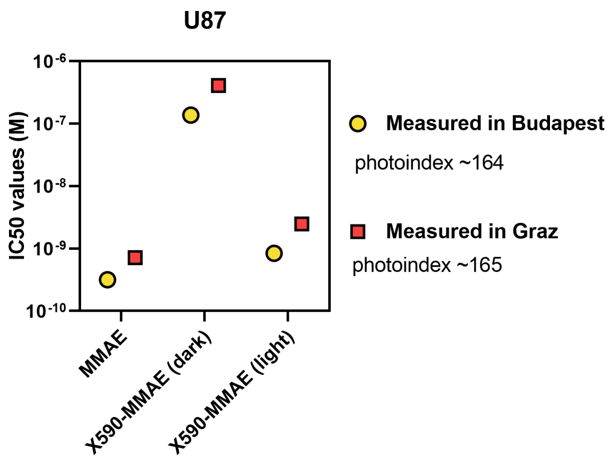

Figure S39. Comparison of the IC50 values determined on U87 cells in Budapest and Graz. Note that at MUG, MTS assay was used.

6.2 Microtubule Imaging

6.2.1 Experimental Details for the Microtubule Imaging

SK-OV-3 cells (8,000 cells/well) were transferred into 8-well chambered IBIDI µ-Slide coverslips (Ibidi 80827) and incubated for 40 h at 37 °C in a 5% CO_2 atmosphere. Live cells were kept at 37 °C continuously to avoid microtubule disruption due to low temperature. Cells were treated with either MMAE or **X590-MMAE** at a concentration of 10^{-8} to 10^{-6} M for 60 minutes (control treatment: 0.1% DMSO) and after the incubation period (with or without a washing step with medium) cells were irradiated with the 605 nm LED for 60 s (4 cycles of 15 s irradiation with 15 sec intermittent pause). To prove the local effect of the drug masked irradiation was carried out as well, samples were shielded with dark aluminum foil and were irradiated through a 1 mm pinhole. Samples were incubated for 24 hours in the dark at 37 °C in a 5% CO_2 atmosphere. Afterwards, cells were washed with PBS and fixed using 4% PFA + 0.1% TritonX-100 in PBS solution for 4 hours in the dark at 37 °C in a 5% CO_2 atmosphere. Samples were washed 4 times with PBS and immunolabeled (after an overnight blocking step (2 mg/ml BSA + 1% fish gelatin (Sigma G7765) + 0.1% Triton X-100 + 5% goat sera (Gibco, 16210-064) in PBS)) with primary antibody against tubulin (1/1000 dilution; 3h at RT; mouse monoclonal antibody; Sigma-Aldrich; T6199). After a careful washing procedure (4 times PBS for 5 min each) the secondary antibody (1/1000 dilution, 1.5 h at room temperature; Goat anti-Mouse IgG (H+L) Highly Cross-Adsorbed Secondary Antibody, Alexa Fluor™ 488; Invitrogen A-11029) was applied. Then, samples were washed 4 times with PBS and stored in 0.02% sodium azide containing PBS until subjected to confocal microscopy analyses.

Confocal images were acquired using a Leica TCS SP8 STED 3x microscope with a 488 nm excitation laser. Images were acquired with a Leica HC PL APO CS2 40x/1.30 oil immersion objective, and fluorescence was detected in the 500-800 nm range using HyD detector. Image processing was carried out with ImageJ software. Tile scan images were recorded with a Leica 10x/0.32 objective.

6.2.2 'No Wash' Microscopy Images

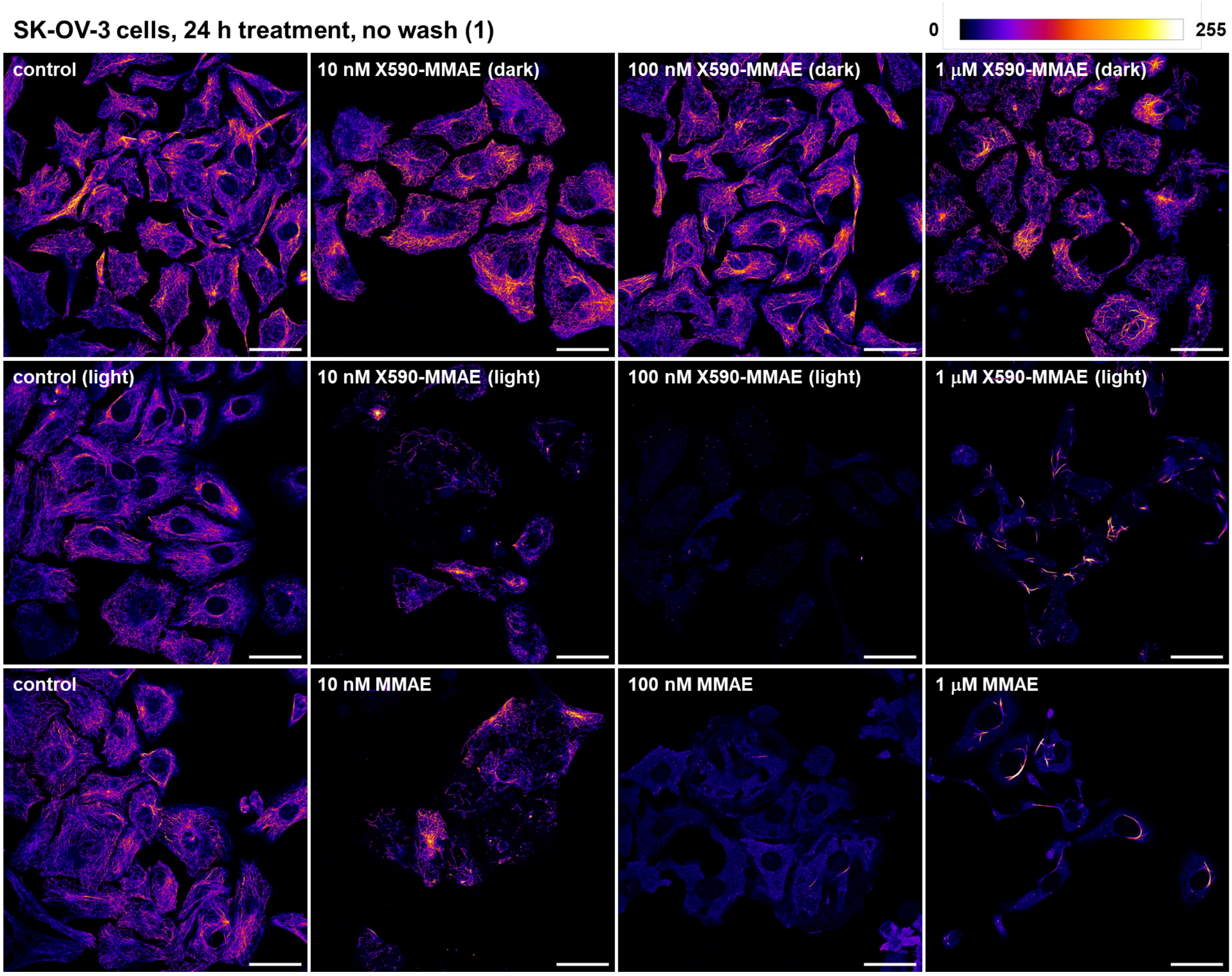

Figure S40. Confocal microscopy images of microtubule immunolabeling of SK-OV-3 cells under various treatment protocols (1st example). Scale bar: 50 µm.

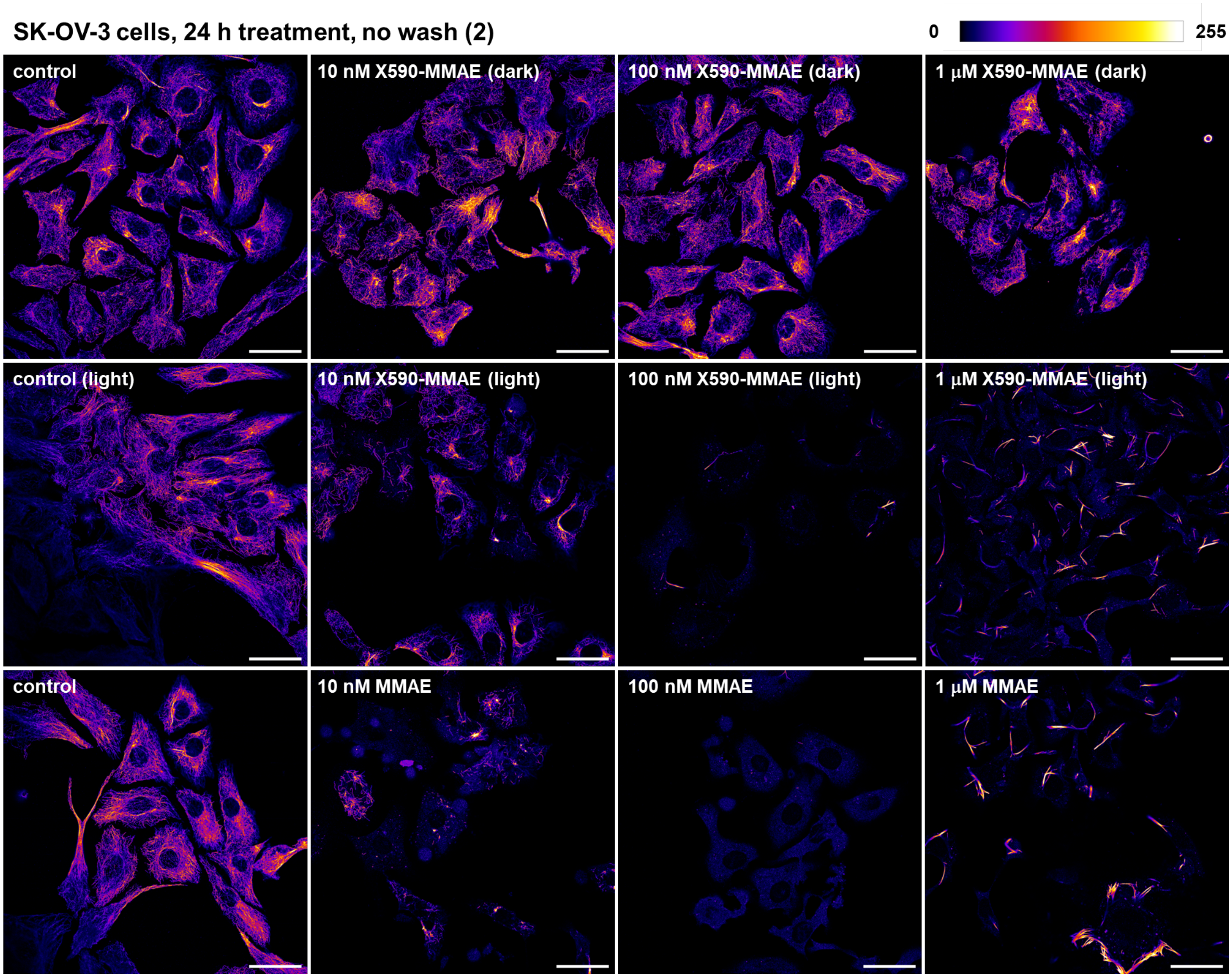

Figure S41. Confocal microscopy images of microtubule immunolabeling of SK-OV-3 cells under various treatment protocols (2nd example). Scale bar: 50 μm.

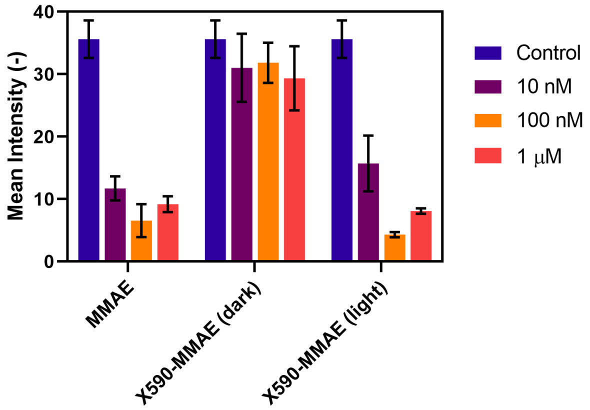

Figure S42. Mean intracellular fluorescence values of the microtubule immunolabeling experiment under various no wash treatment protocols.

7.2.2 'Wash' Microscopy Images

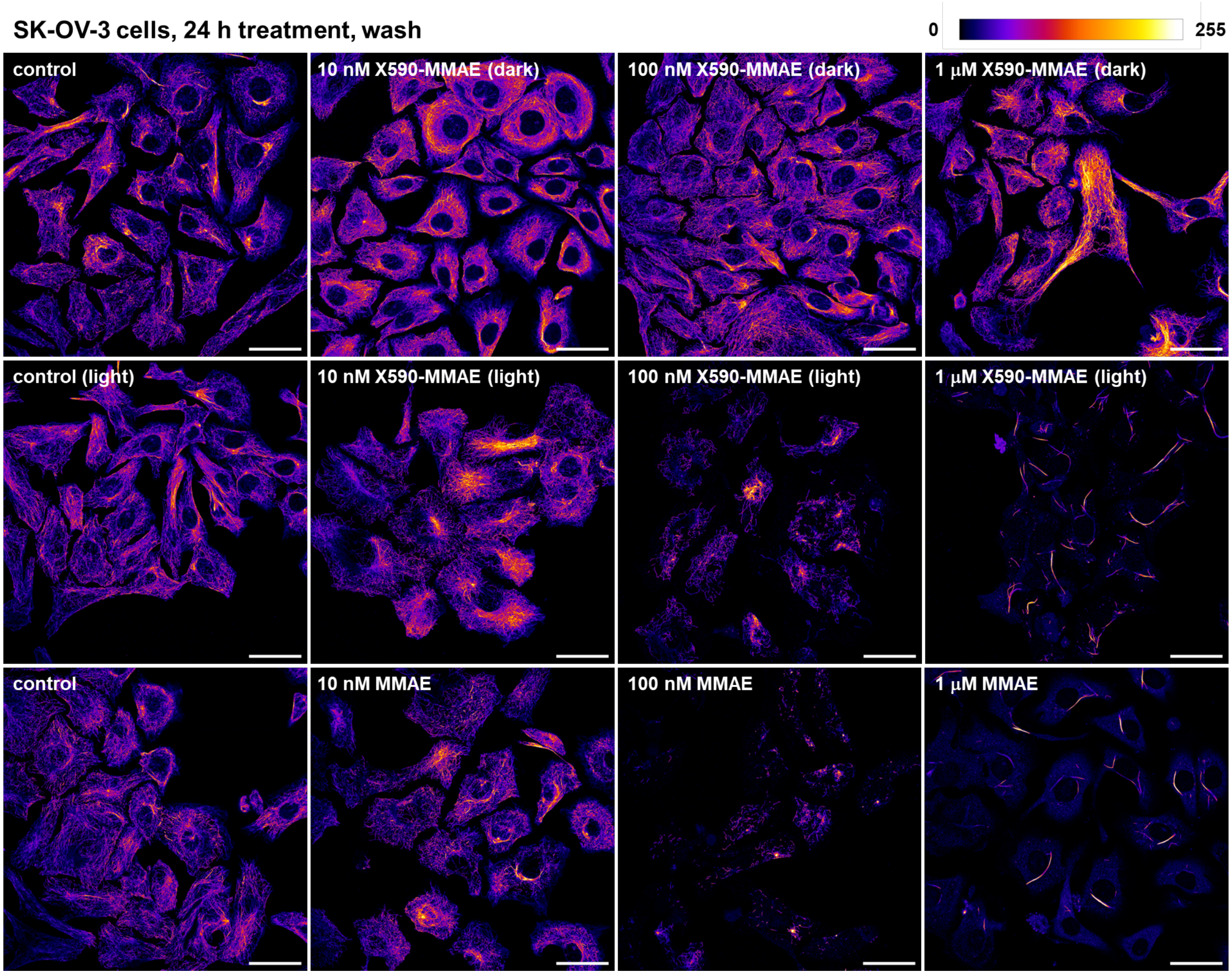

Figure S43. Confocal microscopy images of microtubule immunolabeling of SK-OV-3 cells under various treatment protocols (wash: media were replaced before irradiation). Scale bar: 50 µm.

7.2.3 Localized Irradiation Images

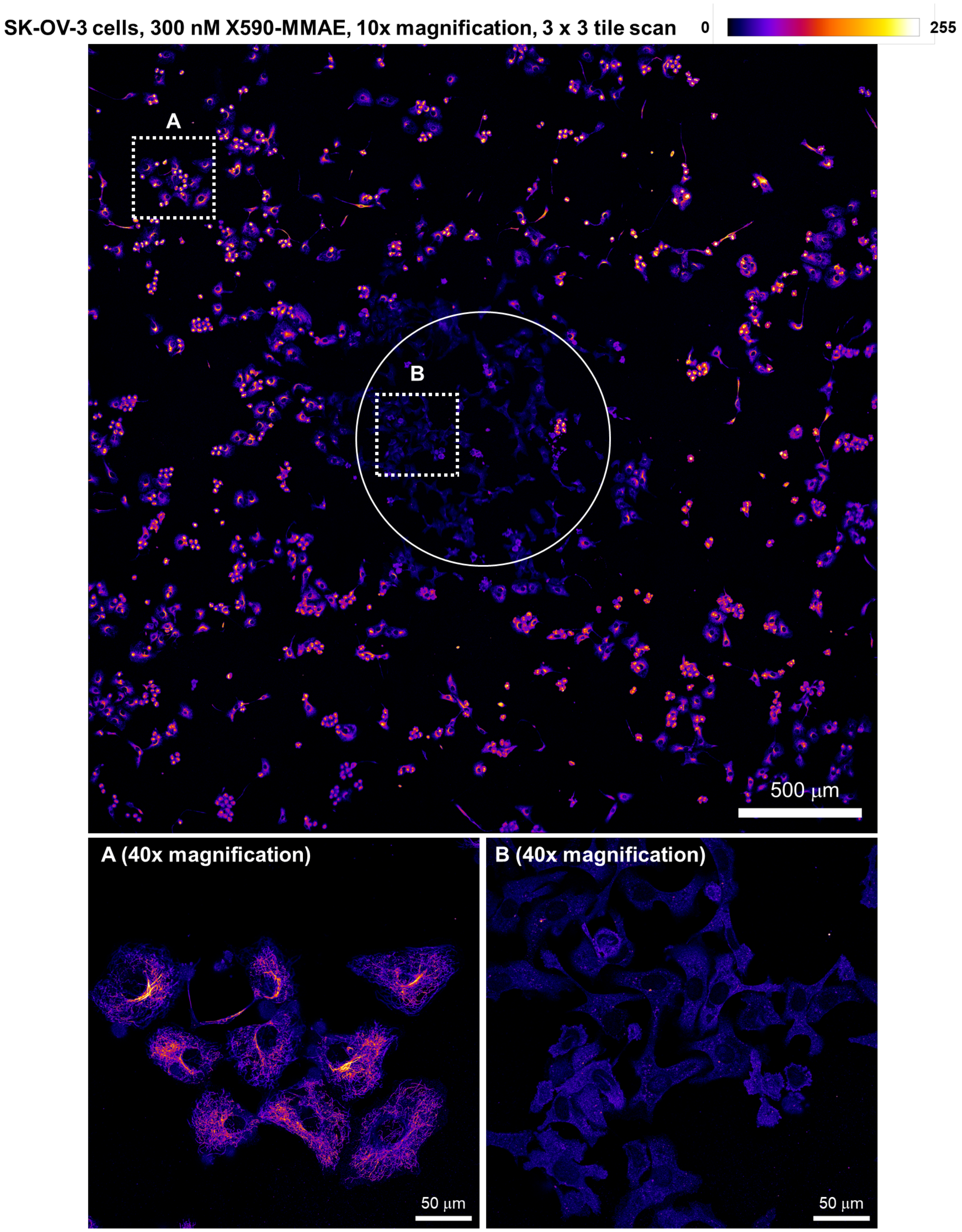

Figure S44. Confocal microscopy images of microtubule immunolabeling of SK-OV-3 cells treated with 300 nM ***X590-MMAE*** *and masked irradiation. The approximate border of the mask is shown in the tile scan image as a white circle (approx. radius ~0.5 mm). The zoomed-in images of the fixed cells were captured using different magnifications.*

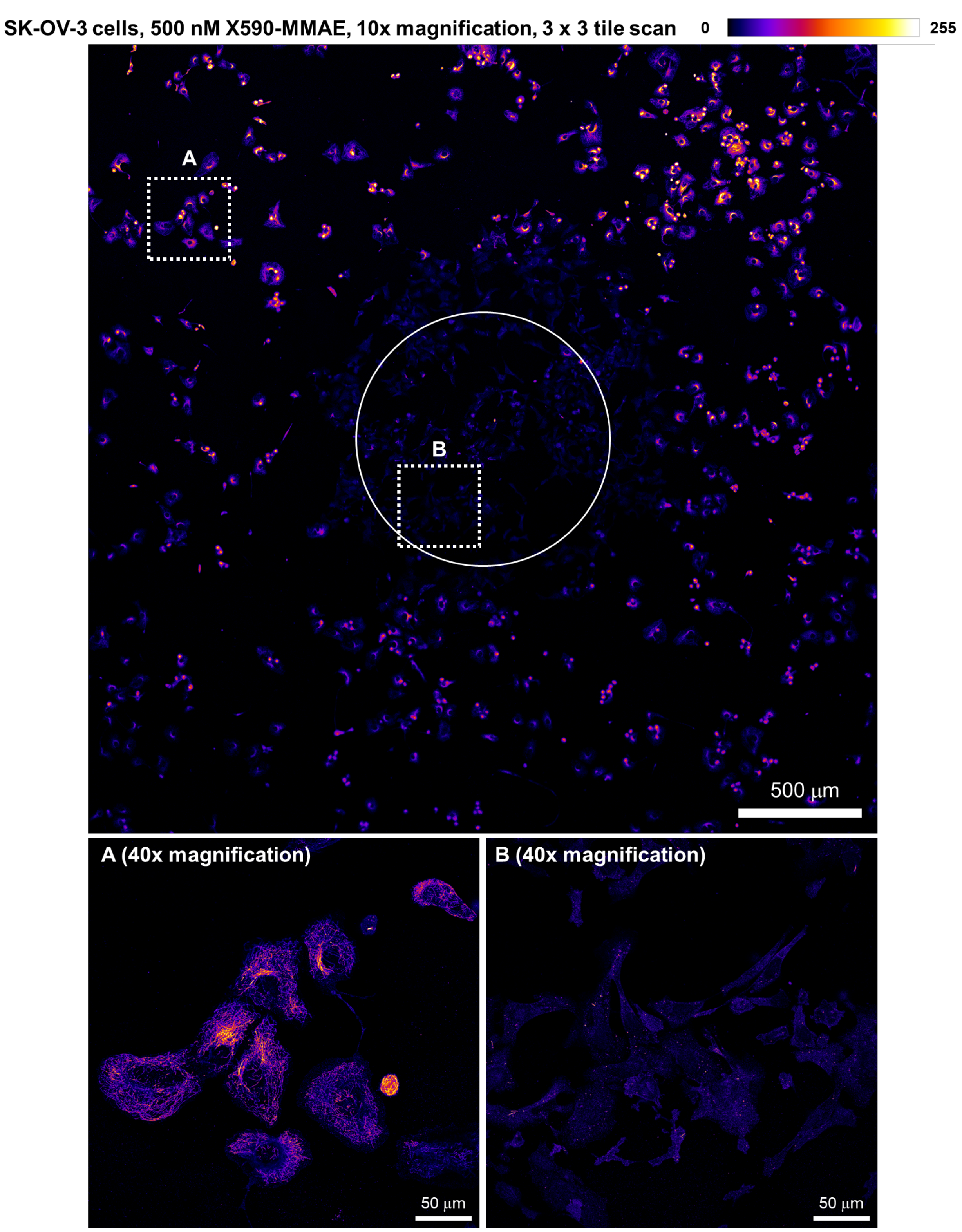

*Figure S45. Confocal microscopy images of microtubule immunolabeling of SK-OV-3 cells treated with 300 nM **X590-MMAE** and masked irradiation. The approximate border of the mask is shown in the tile scan image as a white circle (approx. radius ~0.5 mm). The zoomed-in images of the fixed cells were captured using different magnifications.*

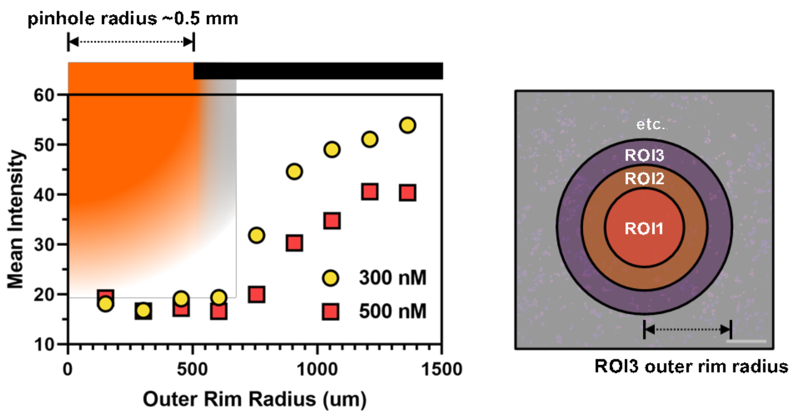

Figure S46. Intensity measurements of the masked irradiation experiments using annuli of increasing diameters. The intracellular intensity measurements were performed according to the figure on the right.

6.3 Colocalization Studies

SK-OV-3 cells (10,000 cells/well) were transferred into µ-Slide 8-well chambered coverslips (Ibidi 80827) and incubated for 40 h at 37 °C in a 5% CO_2 atmosphere. Cells were then treated with either 100 nM **X590-MMAE** (in medium) in combination with 5 nM LysoTracker Deep Red (Invitrogen L12492) or 5 nM MitoTracker Deep Red (Invitrogen M22426) for 90 min. Following treatment, cells were subjected to confocal microscopy with a Leica TCS SP8 STED 3x microscope. The images were acquired with a Leica HC PL APO CS2 40x/1.30 oil immersion objective using dual-channel detection with sequential imaging. Excitation/emission settings were as follows: green excitation channel (for **X590-MMAE**, yellow LUT), excitation at 552 nm and emission within the 562-599 nm range; red channel (for organelle trackers), excitation at 638 nm and emission acquired within the 700-800 nm range. Image processing was performed with ImageJ software.

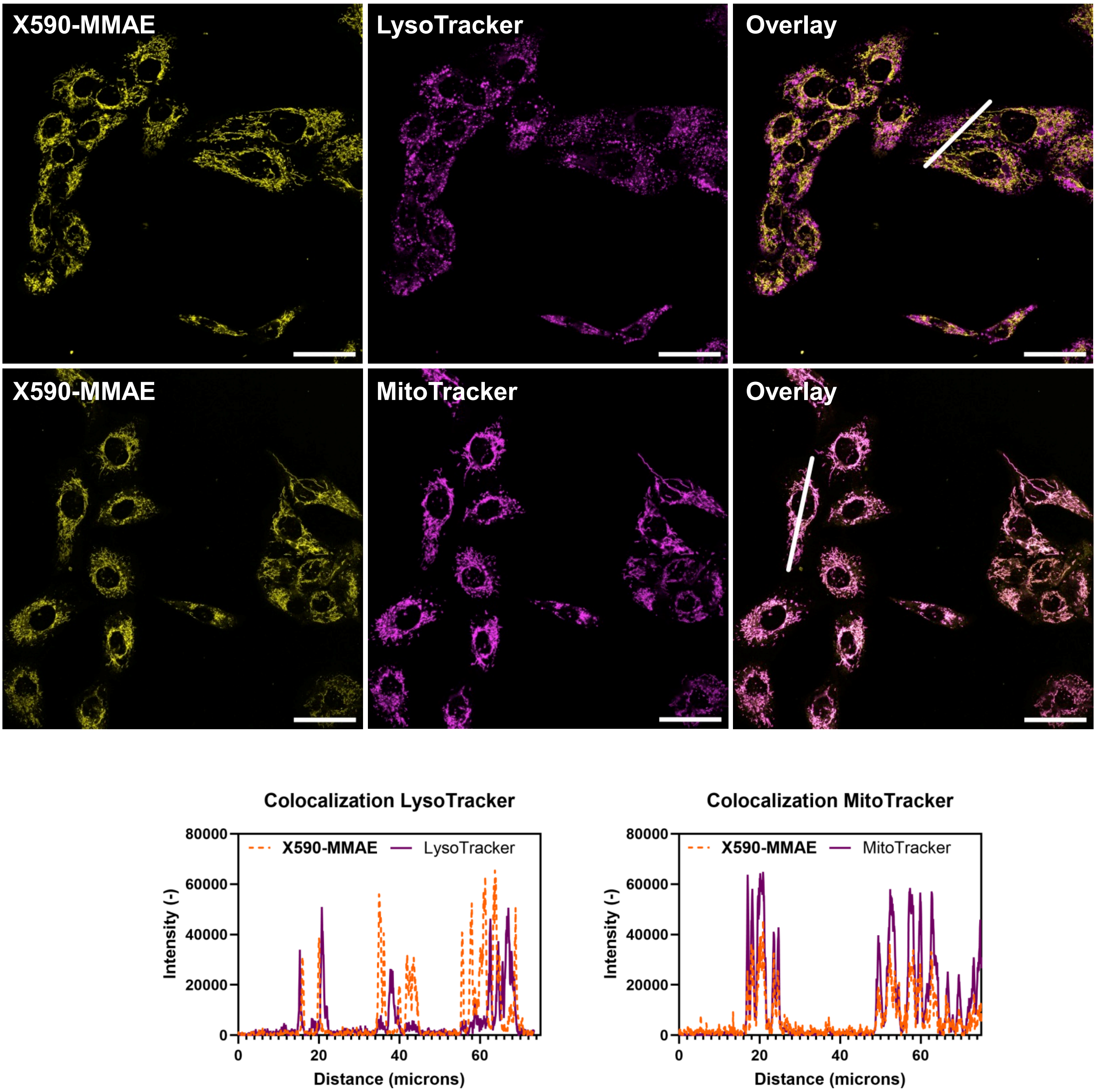

Figure S47. Confocal microscopy images of SK-OV-3 cells treated with 100 nM ***X590-MMAE*** *and 5 nM of either LysoTracker Deep Red or MitoTracker Deep Red. The white lines are plotted on the graphs to show the colocalization with the trackers. Scale bar: 50 µm.*

7. Spheroid Experiments

7.1 Experimental Details for the Spheroid Experiments

HeLa or HCT-116 cells were plated (5000 cells/well) on Nunclon™ Sphera™ 96-Well, Nunclon Sphera-Treated, U-Shaped-Bottom Microplate (174925) and incubated at 37°C in a 5% CO_2 atmosphere for two days to form compact spheroids. 3D spheroids were treated with MMAE or **X590-MMAE** at concentrations ranging from 10^{-12} to $2x10^{-5}$ M for 240 minutes (control treatment: 0.1% DMSO) before irradiation with 605 nm LED for 60 s (4 cycles of 15 s irradiation with 15 sec intermittent pause). Dark samples were kept in the dark during the whole procedure. Samples were incubated for 7 days in the dark at 37 °C in a 5% CO_2 atmosphere. Spheroid samples treated with high concentration (2x 10^{-5} M -10^{-6} M) of **X590-MMAE** were washed with medium three times to wash out the fluorescent background caused by the photocage interfering with resorufin fluorescence. Resazurin solution (40 µM) was added to the medium and incubated for 24 hours. Fluorescence was detected at 530 nm excitation and 560 nm emission wavelength using a Biotek Synergy 2 Cytation 3 imaging plate reader with Gen5 software (version 3.11, Biotek, Winooski, VT, USA). Cell viability was expressed as the percentage of DMSO treated control spheroids (n = 3). IC50 values were calculated using GraphPad Prism software (ver. 8.0.1) applying non-linear regression viable slope (four parameters). Spheroids were imaged with 4x magnification with Biotek Synergy 2 Cytation 3 imaging plate reader with Gen5 software (version 3.11, Biotek, Winooski, VT, USA).

7.2 Spheroid Images

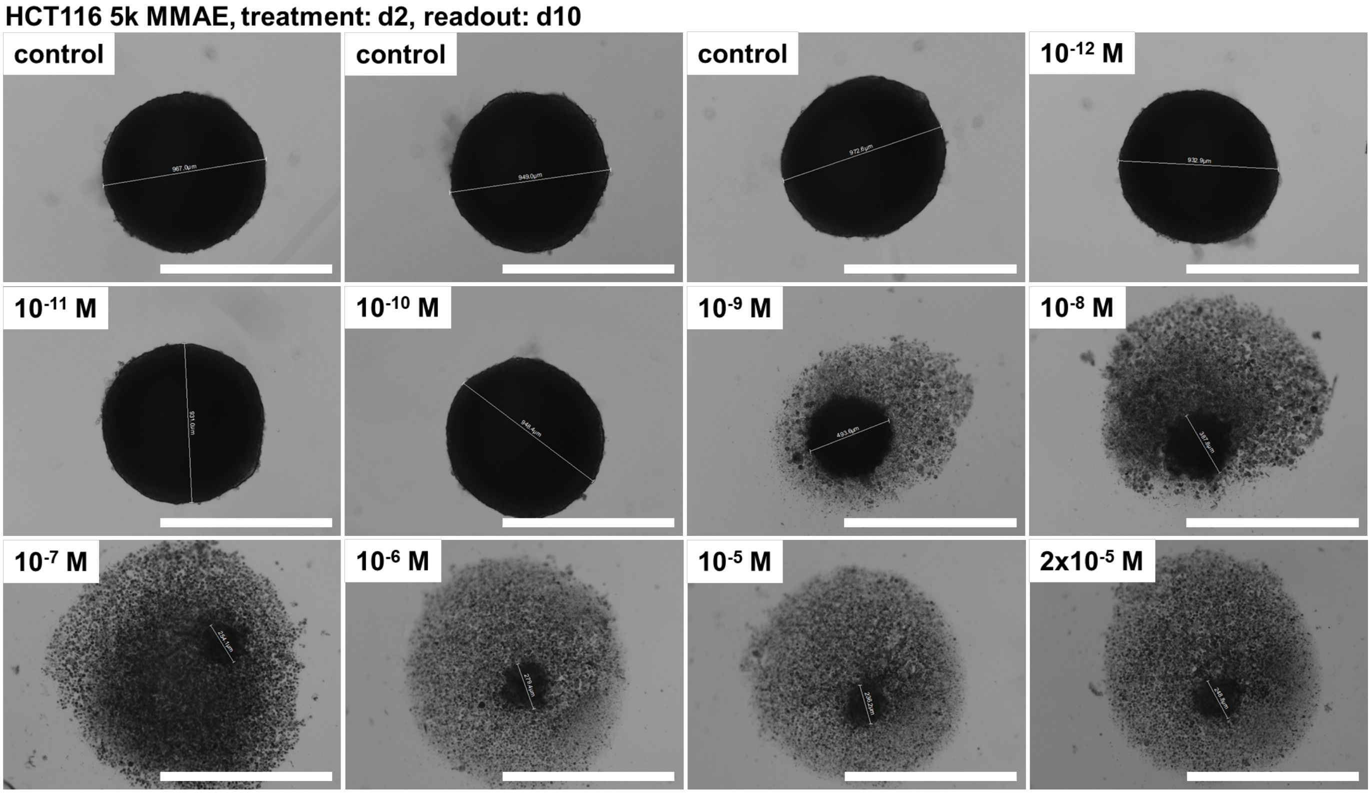

Figure S48. Microscopy images of HCT116 spheroids treated with MMAE. Scale bar: 1 mm.

HCT116 5k X590-MMAE (dark), treatment: d2, readout: d10

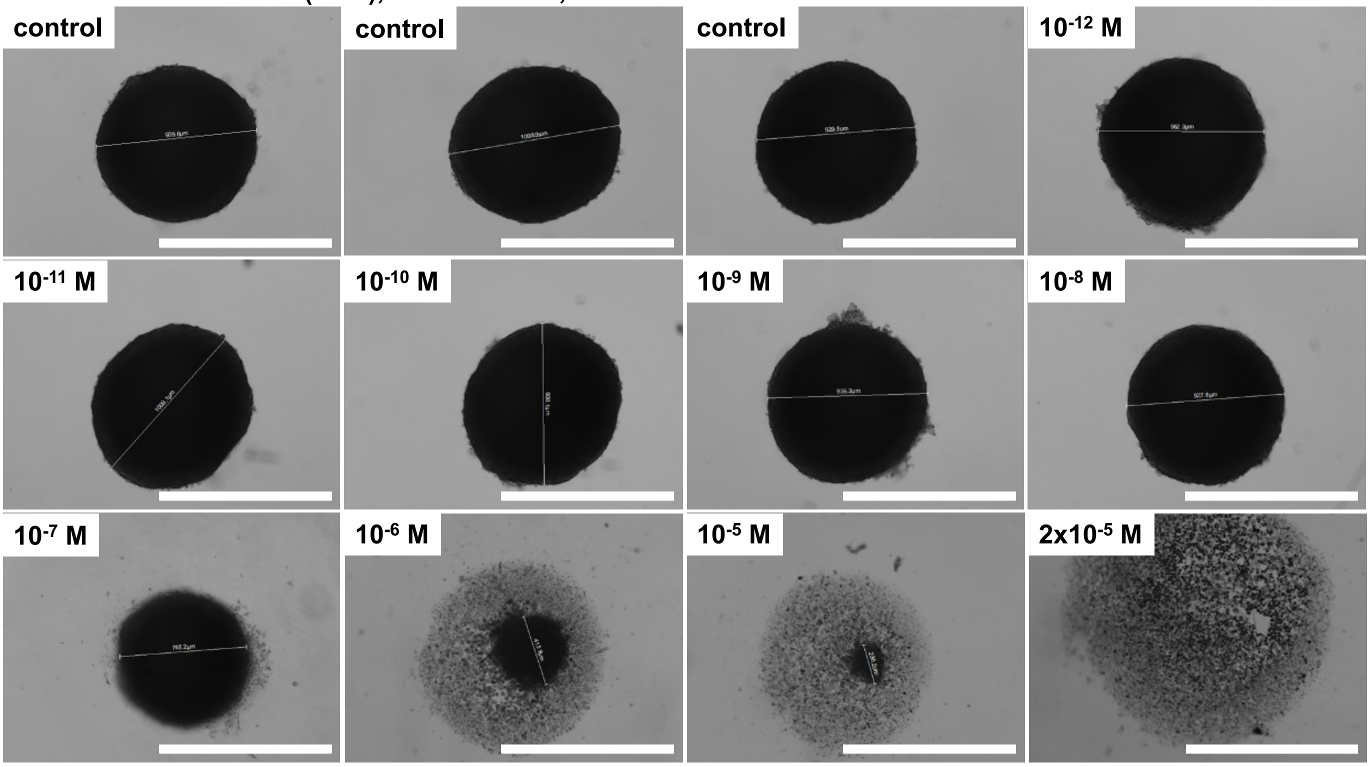

*Figure S49. Microscopy images of HCT116 spheroids treated with **X590-MMAE** in the dark. Scale bar: 1 mm.*

HCT116 5k X590-MMAE (60 s orange light), treatment: d2, readout: d10

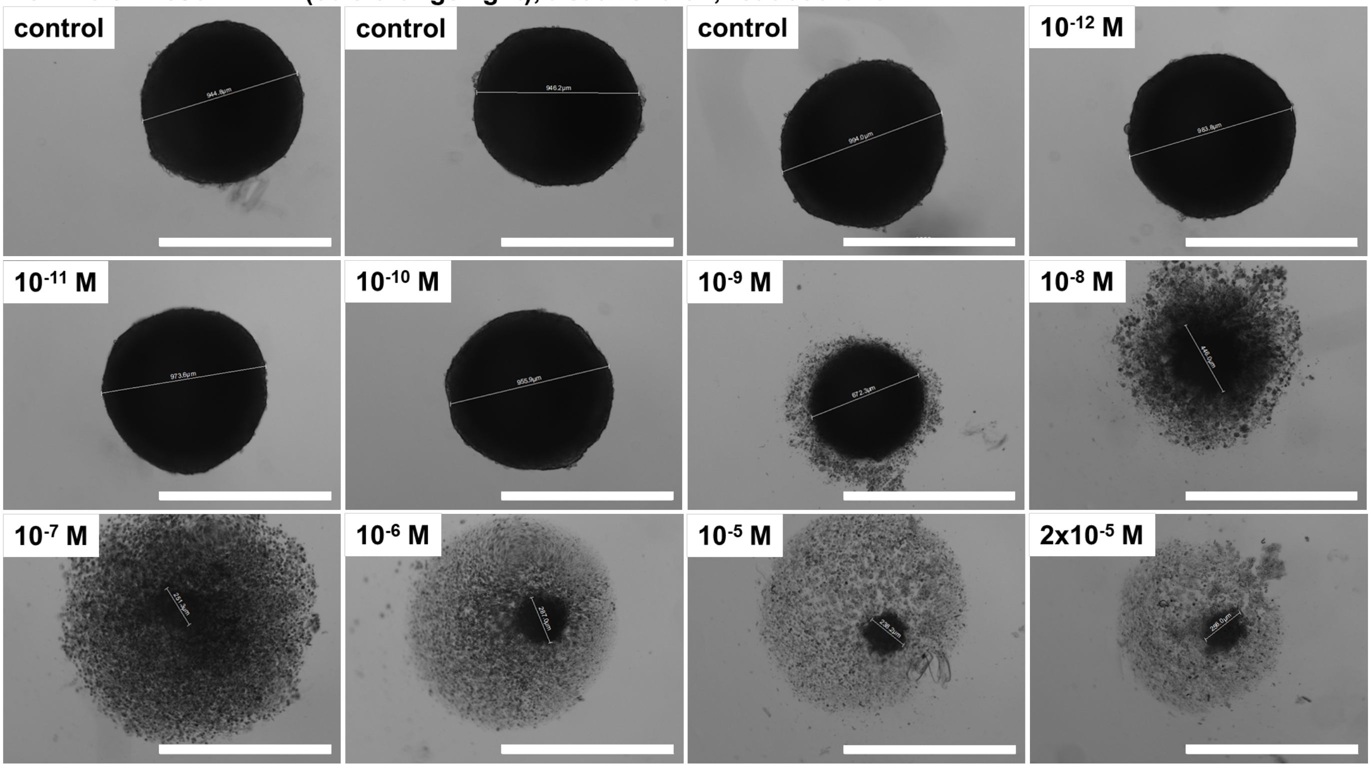

*Figure S50. Microscopy images of HCT116 spheroids treated with **X590-MMAE** and 60 s orange light. Scale bar: 1 mm.*

HeLa 5k MMAE, treatment: d2, readout: d10

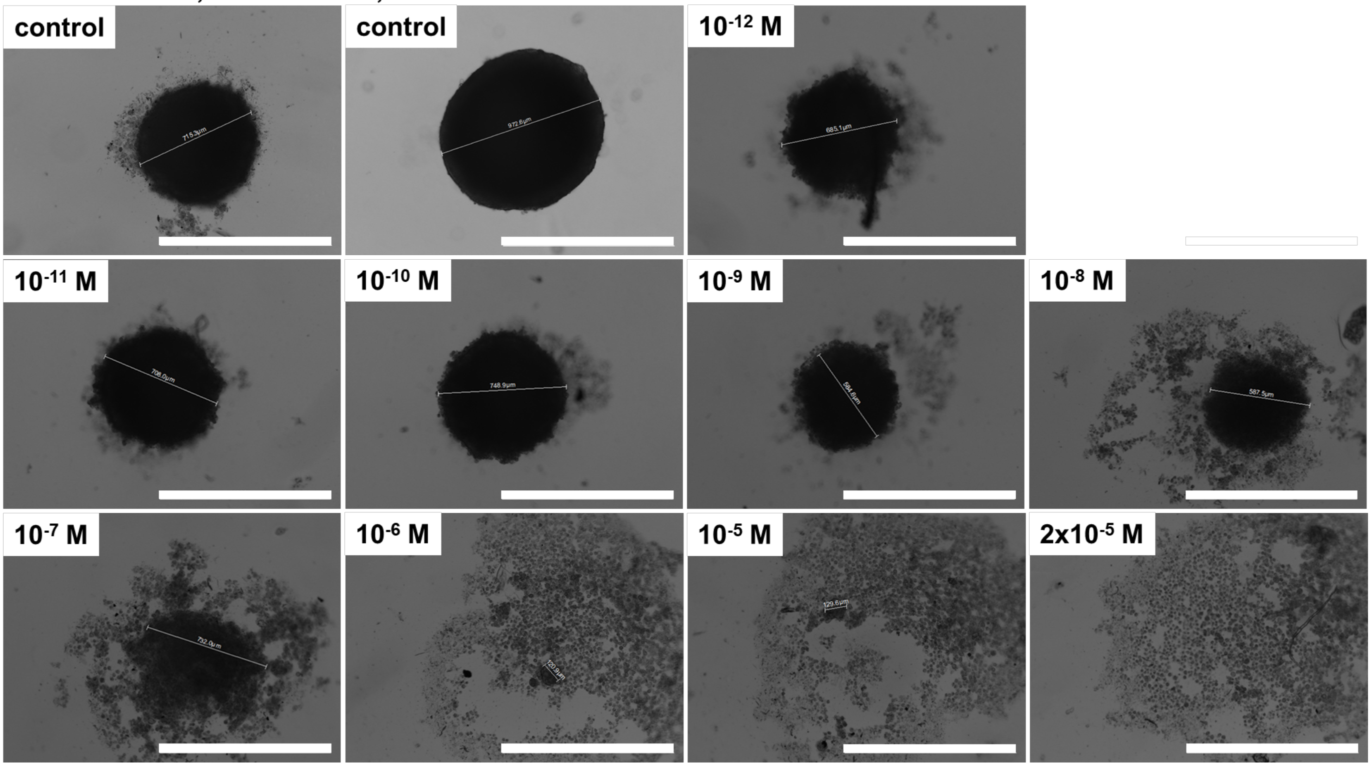

Figure S51. Microscopy images of HeLa spheroids treated with MMAE. Scale bar: 1 mm.

HeLa 5k X590-MMAE (dark), treatment: d2, readout: d10

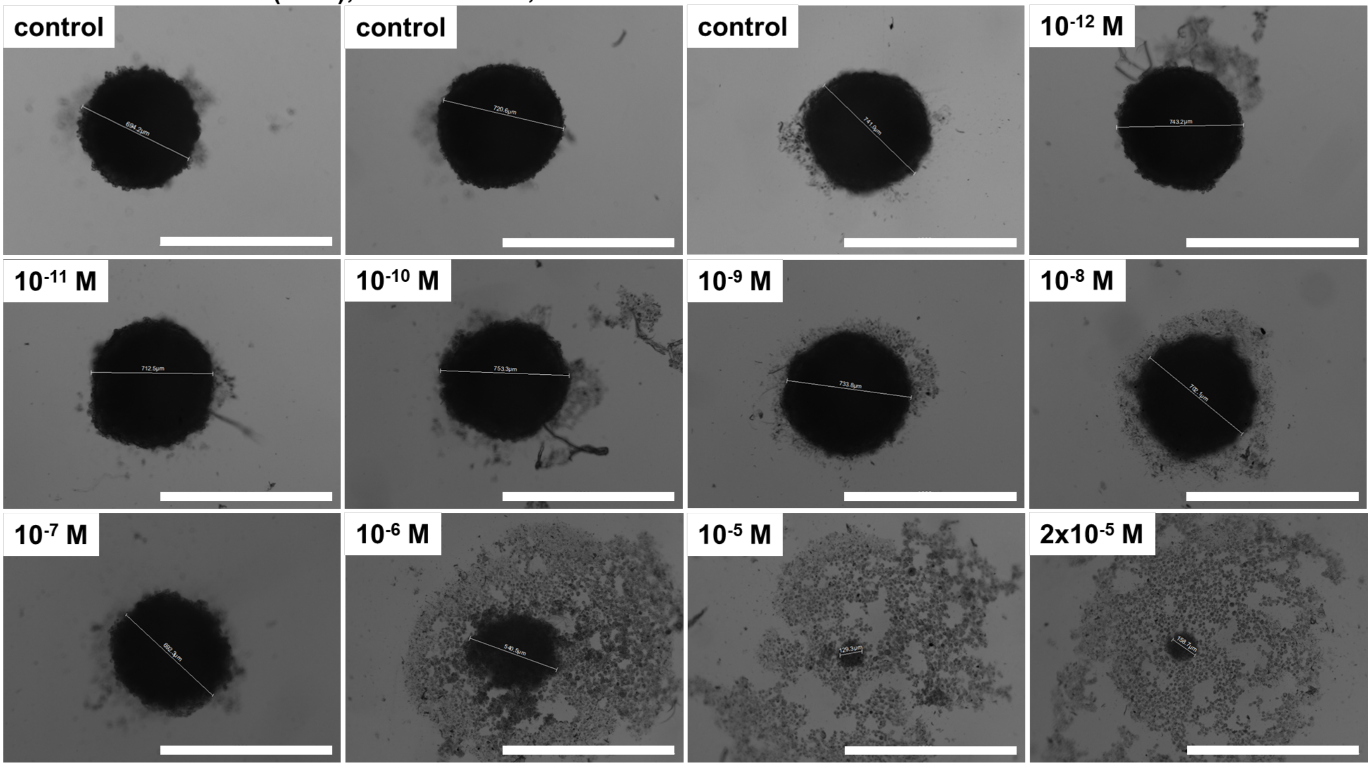

*Figure S52. Microscopy images of HeLa spheroids treated with **X590-MMAE** in the dark. Scale bar: 1 mm.*

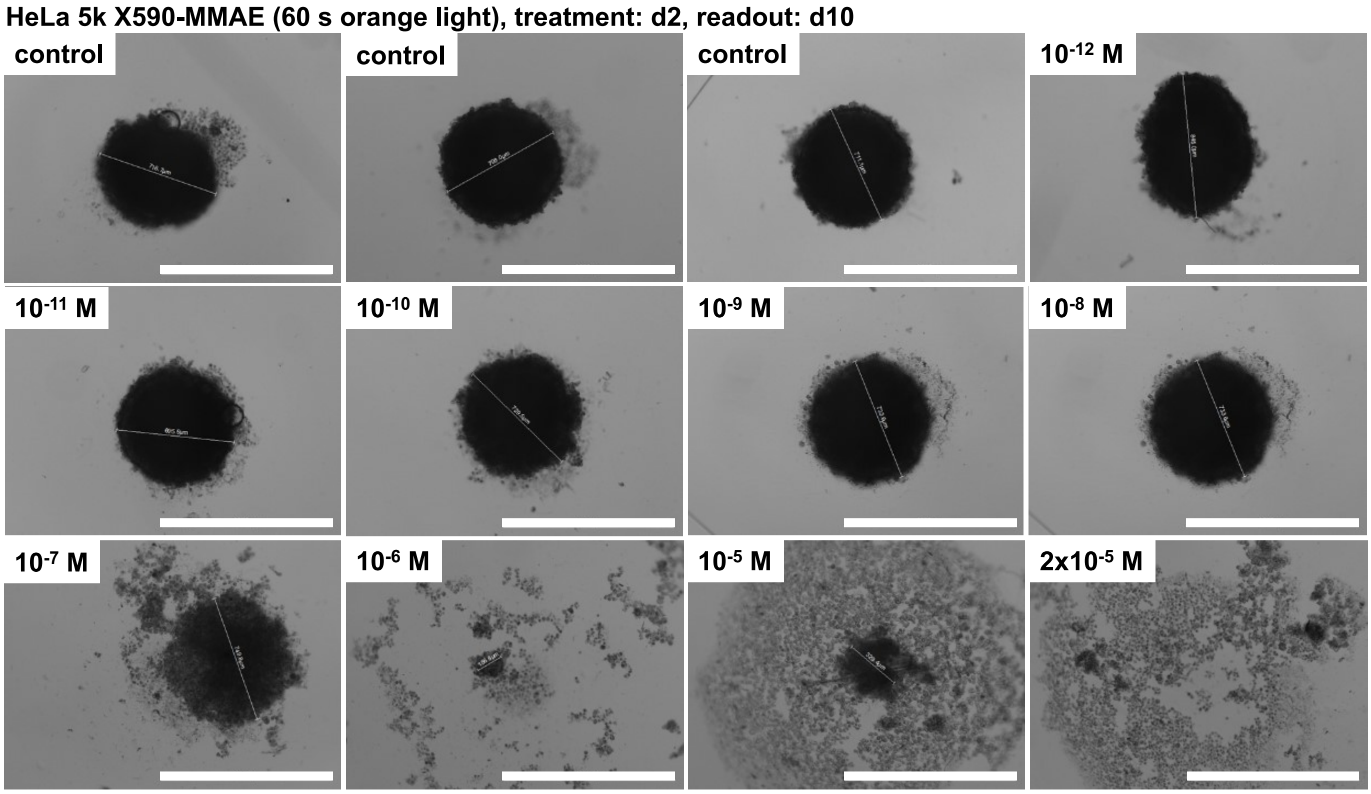

Figure S53. Microscopy images of HeLa spheroids treated with ***X590-MMAE*** *and 60 s orange light. Scale bar: 1 mm.*

8. CAM Assay

8.1 Light Source Developed for the CAM Assay

For the CAM experiments, a Thorlabs lens system was developed using an orange LED light source (M595L4, Thorlabs, Newton NJ, USA; wavelength range: 585 - 615 nm, 4.5 W input power, 120 mW output power). To focus the light beam emitted by M595L4, it was first collimated using an anti-reflection-coated aspheric condenser lens (ACL5040U-A, Thorlabs, NA = 0.6, focal length = 29 mm) and subsequently focused with a plano-convex lens (LA1131-ML, Thorlabs, backfocal length = 46.3 mm). It will be further referred to 'Thorlabs lens system' and is shown in Figure S54.

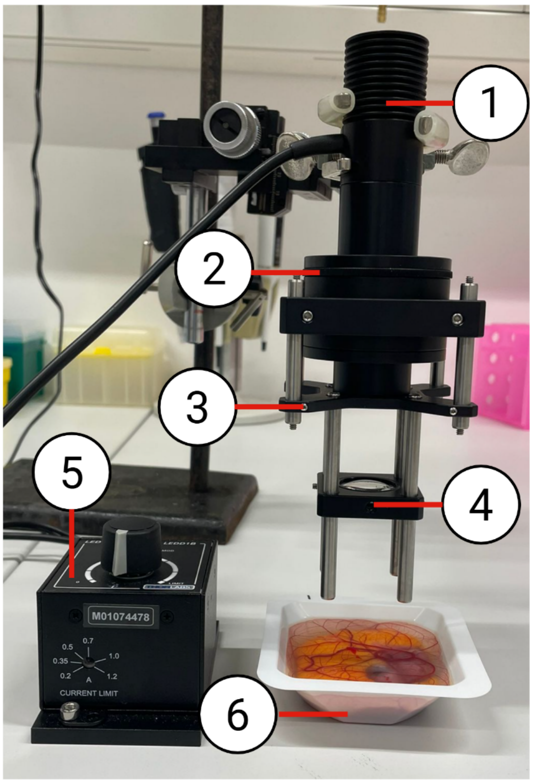

Figure S54. Photograph of the Thorlabs lens system. 1: High power LED M595L; 2: collimating lens ACL5040U-A in its associated lens tube; 3: caged system for lens system positioning; 4: adjustable plano convex lens LA1131-ML; 5: LED T-cube driver (LEDD1B, Thorlabs); 6: Petri dish with ex ovo CAM assay.

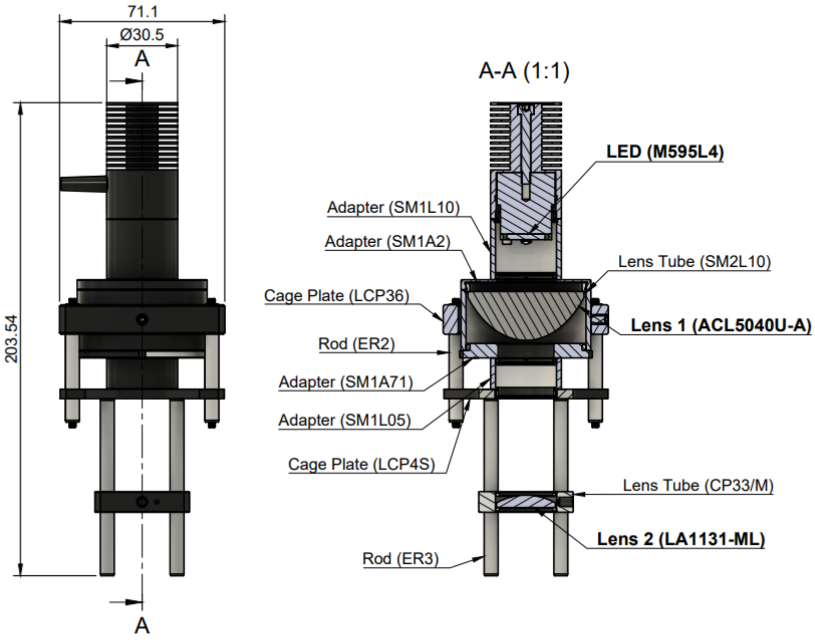

Figure S55. Schematic of the optical setup forming the Thorlabs lens system including true to scale dimensions in mm (left). Sectional view (right) includes all acquired parts for assembly. Lens 1 (ACL5040U-A) and Lens 2 (LA1131- ML) collect and direct the beam emitted by the LED (M595L4). Stability of the total setup is ensured by the combination of cage plates and rods.

8.2 Experimental Details for the CAM Assay

For the CAM assay the human neuronal glioblastoma cell line U-87 MG was provided by the Medical University of Graz. Cells were cultured in Eagle's Minimum Essential Medium (E-MEM) supplemented with 1 mM sodium pyruvate, 0.1 mM non-essential amino acids, 2 mM L-glutamine, 1% penicillin–streptomycin, and 10% FBS at 37 °C and 5% CO_2. For the *ex ovo* CAM assay, fertilized Lohmann white eggs were cleaned with tepid water and incubated for 72 h at 37.6 °C and 40–60% humidity. On embryonic development day (EDD) 3, eggs were disinfected with 75% ethanol and cracked into sterile Petri dishes using an electrical blade. Eggs were covered and incubated for another 7 days under standard conditions. Cell engraftment was performed on EDD 10. Cells were harvested using TrypLE (Thermo Fisher Scientific), centrifuged twice, resuspended in PBS, and counted using a CASY I cell counter. A master mix containing 1×10^6 cells in 15 µL PBS and 5 µL Geltrex® (Thermo Fisher Scientific) was prepared and kept on ice. Sterile silicone rings were placed on the CAM away from the embryo and major blood vessels, and 20 µL of the cell suspension was carefully pipetted into each ring. Embryos were then again incubated at 37.6 °C and 40–60% humidity. Treatment was performed by installing Thorlabs lens as shown above. For intravenous injections patch pipettes were pulled and connected to thin plastic tubing, which was attached to a 1 mL syringe for manual injection. After preparation of the injection solution, ensuring the absence of air bubbles, embryos were positioned under a stereomicroscope, and a suitable vessel was identified. Injections were performed exclusively into veins, which were distinguished by their lighter color due to oxygenated blood, ensuring that the injected solution flowed with the bloodstream. Arterial injections were avoided, as they can cause severe bleeding and embryo lethality. The patch pipette was aligned in the same focal plane as the target vein, carefully advanced, and used to penetrate the vessel wall. A total volume of 100 µL was slowly injected. Tubulin immunohistochemistry was performed on paraffin sections following deparaffinization in xylol and ethanol. Endogenous peroxidase activity was blocked with H_2O_2, followed by antigen retrieval using Sodium citrate buffer (pH 6.1) in a pressure cooker for 10 min at 95°C. Sections were blocked for 1 h (2 mg/mL BSA + 1 % fish gelatine (Sigma) + 0,1 % Triton X-100 + 5 % goat serum) and incubated overnight at 4 °C with anti-tubulin primary antibody (1:1000, Sigma). After washing, sections were incubated for 2 h at room temperature with Alexa Fluor 488 goat anti-mouse secondary antibody (1:1000), washed, and mounted with Vectashield (Vector Laboratories).

8.3 Survival Plot

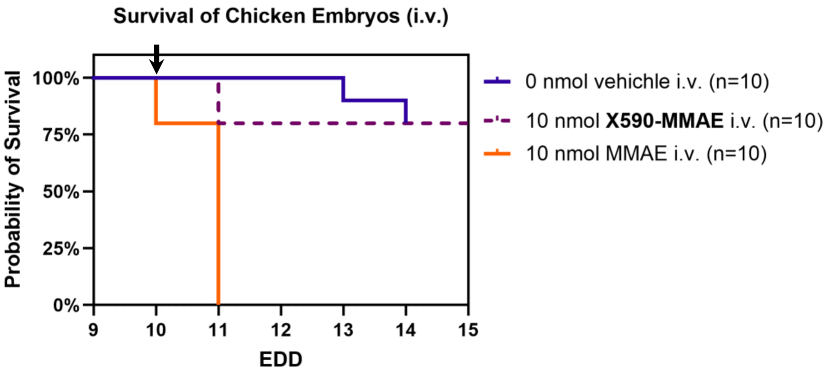

*Figure S56. Kaplan-Meier plot for chicken embryo survival upon different i.v. administration of PBS (vehicle), **X590-MMAE** (dark) and MMAE. Treatment was administered on EDD 10 (indicated by the arrow).*

9. GPCR Photoactivation

9.1 Molecular cloning

Plasmid allowing the mammalian expression of 5-HT_{2C} receptor (HTR2C) under the control of CMV promoter was constructed using polymerase chain reaction (PCR) and restricition cloning. All restriction endonucleases were purchased from NEB (New England Biolabs, MA, USA). HTR2C gene was amplified by PCR from HTR2C-Tango vector (Addgene #66411) using forward primer 5'-gcagagctctctggctaactagagaac-3' and reverse primer 5'-GTCACTCGAGatcactgatgatatccgc-3' with XhoI restriction site. pcDNA3.1-HTR2C was generated after insert PCR product and pcDNA3.1 (+) vector (Invitrogen) were digested using NotI-HF and XhoI restriction enzymes.

9.2 Cell culture

HEK293T (human embryotic kidney) cells were purchased from American Type Culture Collection (ATCC CRL-3216, Manassas, VA, USA). Cells were maintained in Dulbecco's modified Eagle's medium (Gibco Thermo Fisher #21063-029, Waltham, MA, USA) supplemented with 1% penicillin-streptomycin (Gibco #15140-122), 1% L-GlutaMAX (Gibco #35050-061), 1% sodium pyruvate (Gibco #11360-070), and 10% FBS (Gibco #10500-064). Cells were cultured at 37°C in a 5% CO_2 atmosphere and passaged using 0.05% trypsin-EDTA (Gibco #25300054) every 3–4 days up to 15-20 passages. Cells were plated in µ-slide 8-well plates (ibidi, Germany), previously coated using 0.01 mg/ml poly-D-lysine (Gibco #A3890401) for 2 hours followed by wash with PBS three times. Cells were seeded 20-24 h prior transfection (55,000 cell/well in 250 µl medium) resulting in 50-60% confluency at the time of transfection. Transfection was performed using JetPrime reagent (PeqLab, Fareham, UK) accoring to the manufacturer's recommendations. Cells were transfected with 0.5 µg pcDNA3.1-HTR2C per well. Medium was replaced 4-6 h after transfection to fresh medium. 24 h after cells were loaded with Fluo-4 AM (4 µM diluted from 2 mM DMSO stock, AAT Bioquest #20551, Pleasaton, CA, USA) in the presence of 0.02% pluronic F-124 (Invitrogen #P6867), 2.5 mM probenecid (Invitrogen #P36400) and 0.05% bovine serum albumin (Sigma Aldrich #A4503, Merck, Darmstadt, Germany) in Krebs-Ringer HEPES buffer (120 mM NaCl, 4.7 mM KCl, 2.2 mM $CaCl_2$, 10 mM HEPES, 1.2 mM KH_2PO_4, 1.2 mM $MgSO_4$, 1.8 g/l D-glucose, pH 7.4) for 1 h at 37°C in a 5% CO_2 atmosphere. Cells were washed twice with prewarmed Krebs-Ringer HEPES buffer and subjected to confocal imaging immediately.

9.3 Confocal imaging and photoactivation

Photocaged compounds **X590-WY** and **X600H-WY** were added to cells at 5 µM concentration from 1 mM DMSO stock in Krebs-Ringer HEPES buffer. DMSO stocks were freshly prepared, stored at -20 °C and used within 1 month. Cells were imaged and photoactivated using a Leica TCS SP8 STED 3x microscope. Fluo-4 fluorescence was acquired with a Leica HC PL APO CS2 40x/1.30 oil immersion objective using a 488 nm laser, and fluorescence was detected in the 500-540 nm range using HyD detector. Photoactivation was carried out using the FRAP module in bleach point mode, defining two point-ROIs near the target cells allowing enough distance to avoid cross-activation between the two lasers. Before photoactivation, 25-50 frames were recorded to define baseline fluorescence and detect abberant photoactivation (1 frame: 0.86 s). Uncaging was performed sequentially using 552 nm and then 638 nm laser at 100% laser power for 500 ms each. Fluorescence was recorded for an additional 150-300 frame. Image processing was carried out with ImageJ software. For fluorescence intensity measurements, target cells were gated individually, and mean fluorescence intensity values were extracted over time. Calcium response ($\Delta F/F_0$) was defined as changes in the fluorescence on each frame (F) from which averaged basal fluorescence signal (F_0) (first 25-50 frames, before uncaging) was subtracted (ΔF), which value was normalized by basal signal (F_0).

9.4 Exemplary Workflow for the Analysis

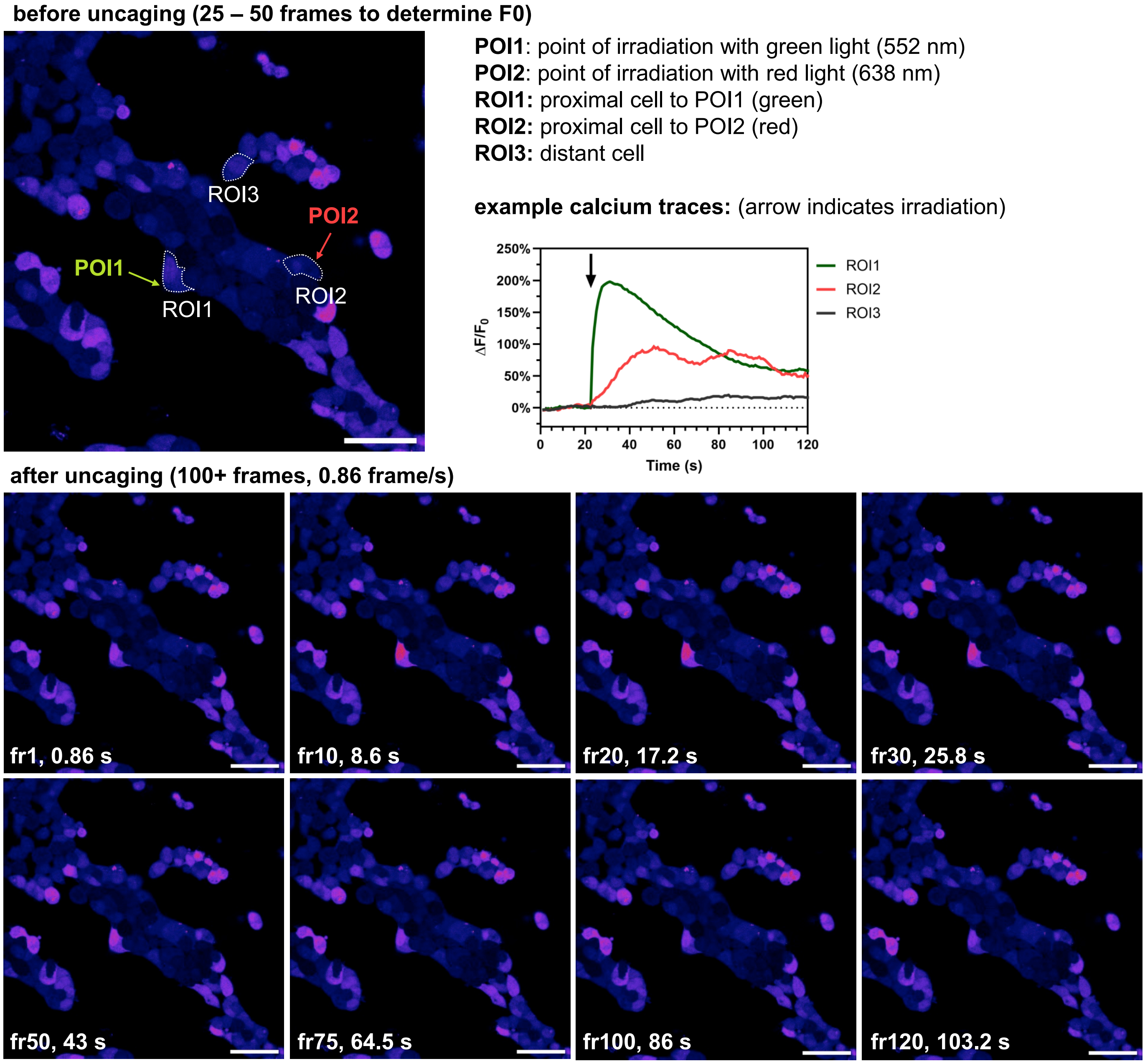

*Figure S57. Exemplary workflow for the analysis of the calcium response after **X590-WY** uncaging, one experiment shown with example images. Frame numbers (fr) indicate the frames recorded after uncaging. Scale bar: 50 μm.*

9.5 Calcium Imaging of Uncaging

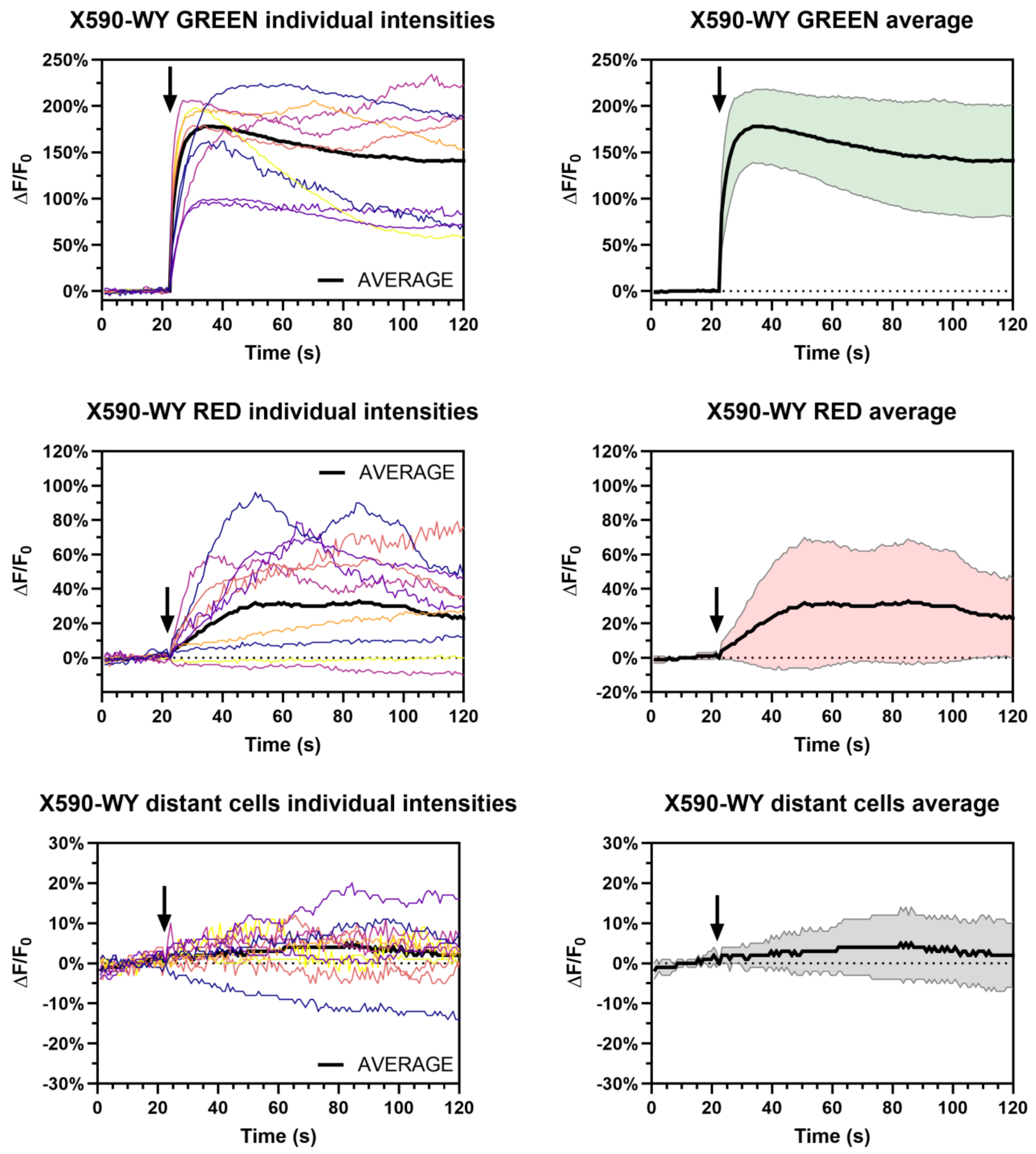

*Figure S58. Analysis of the calcium traces measured within the proximal and distant cells to the point of irradiation before and after uncaging of **X590-WY**. The arrows indicate the irradiation time points (0.5 s for each laser line). Black lines represent average calcium traces of nine experiments with colored standard deviation after 552 nm uncaging (green), 638 nm uncaging (red) and distant cells (grey).*

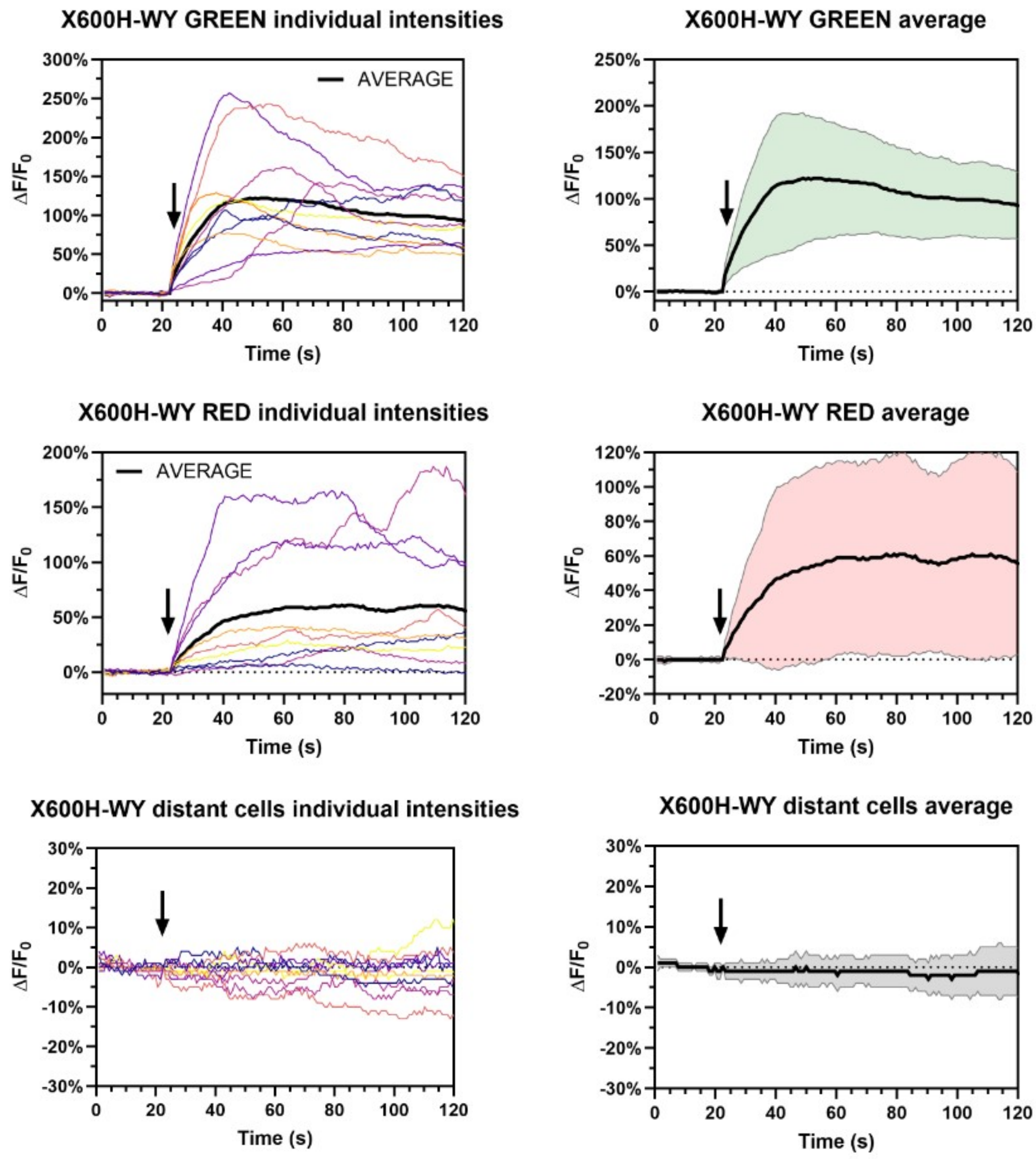

*Figure S59. Analysis of the calcium traces measured within the proximal and distant cells to the point of irradiation before and after uncaging of **X600H-WY**. The arrows indicate the irradiation time points (0.5 s for each laser line). Black lines represent average calcium traces of nine experiments with colored standard deviation after 552 nm uncaging (green), 638 nm uncaging (red) and distant cells (grey).*

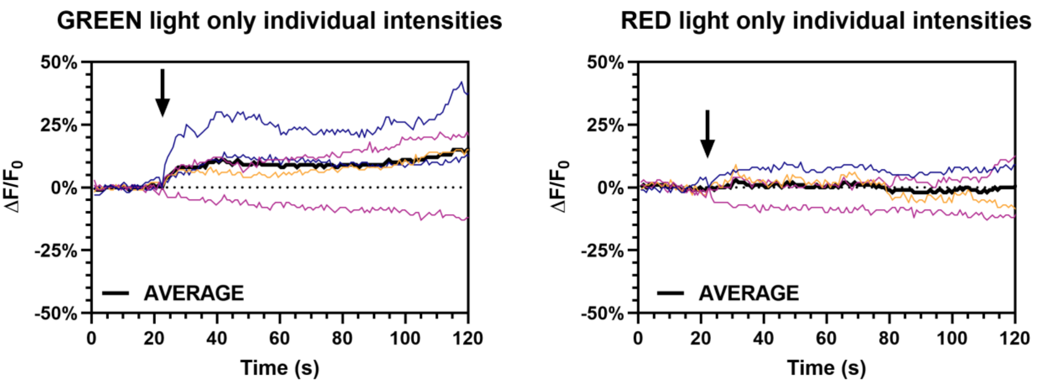

*Figure S60. Analysis of the calcium traces within the proximal cells to the point of irradiation in the absence of photocaged **X590-WY** or **X600H-WY**. The arrows indicate the irradiation time points (0.5 s for each laser line). Black lines represent average calcium traces of five experiments with colored standard deviation after 552 nm uncaging (green), 638 nm uncaging (red).*

10. References

[1] Rurack, K.; Spieles, M. Fluorescence Quantum Yields of a Series of Red and Near-Infrared Dyes Emitting at 600−1000 Nm. *Anal. Chem.* **2011**, *83* (4), 1232–1242. https://doi.org/10.1021/ac101329h.

[2] Egyed, A.; Németh, K.; Molnár, T. Á.; Kállay, M.; Kele, P.; Bojtár, M. Turning Red without Feeling Embarrassed — Xanthenium-Based Photocages for Red-Light-Activated Phototherapeutics. *J. Am. Chem. Soc.* **2023**, *145* (7), 4026–4034. https://doi.org/10.1021/jacs.2c11499

[3]: Butkevich, A. N.; Sednev, M. V.; Shojaei, H.; Belov, V. N.; Hell, S. W. PONy Dyes: Direct Addition of P(III) Nucleophiles to Organic Fluorophores. *Org. Lett.* **2018**, *20* (4), 1261–1264. https://doi.org/10.1021/acs.orglett.8b00270.

[4]: Wei, L.; Chen, Z.; Shi, L.; Long, R.; Anzalone, A. V.; Zhang, L.; Hu, F.; Yuste, R.; Cornish, V. W.; Min, W. Super-Multiplex Vibrational Imaging. *Nature* **2017**, *544* (7651), 465–470. https://doi.org/10.1038/nature22051.

[5]: Bachman, J. L.; Escamilla, P. R.; Boley, A. J.; Pavlich, C. I.; Anslyn, E. V. Improved Xanthone Synthesis, Stepwise Chemical Redox Cycling. *Org. Lett.* **2019**, *21* (1), 206–209. https://doi.org/10.1021/acs.orglett.8b03661.

[6]: Ghosh, A. K.; Duong, T. T.; McKee, S. P. Di(2-Pyridyl) Carbonate Promoted Alkoxycarbonylation of Amines: A Convenient Synthesis of Functionalized Carbamates. *Tetrahedron Lett.* **1991**, *32* (34), 4251–4254. https://doi.org/10.1016/S0040-4039(00)92141-5.

[7]: Zhang, H.; Liu, J.; Sun, Y.-Q.; Huo, Y.; Li, Y.; Liu, W.; Wu, X.; Zhu, N.; Shi, Y.; Guo, W. A Mitochondria-Targetable Fluorescent Probe for Peroxynitrite: Fast Response and High Selectivity. *Chem. Commun.* **2015**, *51* (13), 2721–2724. https://doi.org/10.1039/C4CC09122A.

[8]: Rosenzweig-Lipson, S.; Sabb, A.; Welmaker, G.; Nelson, J. *2,3,4,4a-Tetrahydro-1H-pyrazino[1,2-a]quinoxalin-5-(6H)-one Derivatives*. U.S. Patent Application US 2001/0051622 A1, published Dec 13, 2001; American Home Products Corp/Wyeth LLC.

[9]: Slanina, T.; Shrestha, P.; Palao, E.; Kand, D.; Peterson, J. A.; Dutton, A. S.; Rubinstein, N.; Weinstain, R.; Winter, A. H.; Klán, P. In Search of the Perfect Photocage: Structure–Reactivity Relationships in Meso -Methyl BODIPY Photoremovable Protecting Groups. *J. Am. Chem. Soc.* **2017**, *139* (42), 15168–15175. https://doi.org/10.1021/jacs.7b08532.
